# Complete biosynthesis of the clinical agent escin Ia

**DOI:** 10.1038/s41467-026-76851-3

**Published:** 2026-08-18

**Authors:** Huihua Wan, Mofan Zhang, Ranran Gao, Xuehua Xie, Xiangxiao Meng, Songchun Guan, Weiqiang Chen, Xue Cao, Wei Yang, Hui Yao, Chen Qin, YinDi Zhu, Yi Zhang, Zhaohua Shi, Xiang Chen, Lichun Ye, Huiyuan Gao, Shilin Chen, Zhichao Xu, Wei Sun

**Affiliations:** 1 State Key Laboratory for Quality Ensurance and Sustainable Use of Dao-di Herbs, Key Laboratory of Beijing for Identification and Safety Evaluation of Chinese Medicine, Institute of Chinese Materia Medica, China Academy of Chinese Medical Sciences, Beijing, China; 2 Key Laboratory of National Forestry and Grassland Administration on Chinese Herbal Medicine, College of Life Science, Northeast Forestry University, Harbin, China; 3 State Key Laboratory of Bioactive Substance and Function of Natural Medicines, Institute of Medicinal Plant Development, Chinese Academy of Medical Sciences & Peking Union Medical College, Beijing, China; 4 School of Chinese Medicine, Wenzhou Medical University, Wenzhou, China; 5 Tianjin Key Laboratory of TCM Chemistry and Analysis, Tianjin University of Traditional Chinese Medicine, Tianjin, China; 6 Key Laboratory of Chinese Medicine Resource and Compound Prescription, Ministry of Education, Hubei University of Chinese-Medicine, Wuhan, China; 7 Hubei-Shizhen Laboratory, Wuhan, China; 8 School of Pharmaceutical Sciences, Sun Yat-sen University, Guangzhou, China; 9 College of Traditional Chinese Medicine, Hubei University of Chinese Medicine, Wuhan, China; 10 School of Traditional Chinese Materia Medica, Shenyang Pharmaceutical University, Shenyang, China; 11 Institute of Herbgenomics, Chengdu University of Traditional Chinese Medicine, Chengdu, China

**Keywords:** Secondary metabolism, Biosynthesis, Plant biotechnology

## Abstract

Escin Ia is a barrigenol-type triterpenoid specific to *Aesculus* seeds and is widely utilized in clinical practice for the treatment of cerebral edema, swelling, and venous insufficiency. Here, we fully elucidated its biosynthetic pathway by identifying ten previously uncharacterized enzymes from *Aesculus chinensis*. AcCYP716BX1/2 catalyze the C-22α and C-28 hydroxylation of the β-amyrin scaffold, and AcCYP714E67 mediates the C-24 hydroxylation to produce protoaescigenin. Sequential glycosylation at the 2’-*O* and 4’-*O* positions is catalyzed by AcUGT94AK1/3/6 and AcUGT87AZ3, respectively, forming aesculuside B. AcCCL3 catalyzes the formation of tigloyl-CoA, and AcBAHD13/17 catalyze the C-21β tigloylation of aesculuside B to yield desacetylescin Ia. These enzymes significantly broaden the catalytic versatility of their respective gene families. Finally, we successfully reconstructed the complete pathway in *Nicotiana benthamiana*, enabling the heterologous production of escin Ia. This work fully elucidates the biosynthesis of escin Ia and establishes a foundation for the green production of barrigenol-type triterpenoid saponins.

## Abstract (web summary)

Escin Ia is a clinically important triterpenoid saponin from *Aesculus chinensis*. Here the authors identify ten biosynthetic enzymes and reconstitute the Escin 1a production in *Nicotiana benthamiana*.

## Introduction

Triterpene glycosides are naturally occurring organic compounds ubiquitously distributed in plants^1^. These triterpenoids have achieved extensive prevalence in both food commodities and clinical pharmaceuticals. Notable examples include QS-21 derived from the soapbark tree^2^, glycyrrhizic acid extracted from licorice^3^, escin Ia (**1**) isolated from the horse chestnut (*Aesculus* spp.)^4^, and ginsenoside Rh_1_ purified from Chinese ginseng^5,6^. Of these, aescins represent a distinct group of barrigenol-type triterpenoids, characterized by multiple hydroxyl groups at C-3β, C-15α, C-16α, C-21β, C-22α, C-24, and C-28, and a branched trisaccharide chain (β-D-glucopyranosyl-(1 → 4)-[β-D-glucopyranosyl-(1 → 2)]-β-D-glucuronopyranosyl acid) attached at C-3β, and acyl modifications at C-21β and C-22α^7,8^. More than 100 different types of aescins have been identified^8,9^. Chief among these, escin Ia (**1**) is the dominant bioactive constituent, which exerts therapeutic effects against cerebral edema and chronic venous insufficiency through its anti‑edematous, anti-inflammatory and vascular-stabilizing properties^10,11^. As a result, escin Ia (**1**) has been developed into oral tablets, topical gels and liniments, and intravenous injections, which are widely applied in clinical practice. In addition to its significant pharmacological effects, escin Ia (**1**) also exhibits a mildly soapy texture and potent antioxidant activity, making it a promising ingredient for cosmetic skincare formulations^12^. However, escin Ia (**1**) can only be isolated from the seeds of the *Aesculus* genus, and its total chemical synthesis has not been achieved. Furthermore, the long juvenile phase of *Aesculus*—from seedling establishment to seed production—together with irregular fruiting in wild stands and urban trees, creates a bottleneck for a consistent raw material supply. Therefore, elucidating the biosynthetic pathway and developing heterologous production platforms for escin Ia (**1**) is crucial to secure a sustainable supply of this compound.

Triterpenoids are derived from the mevalonate (MVA) pathway, followed by cyclization via oxidosqualene cyclases (OSCs) and decoration via cytochrome P450 monooxygenases (P450s), UDP-glycosyltransferases (UGTs), and BAHD acyltransferases^13–15^, which together contribute to the structural diversity of these compounds. CYP716 is an ancient P450 family demonstrated to be a major contributor to the diversification of eudicot triterpenoid biosynthesis^16–19^. In soapbark, CYP716A224 (C-28 oxidation), CYP716A297 (C-16α hydroxylation), and CYP714E52 (C-23 oxidation) drive the formation of quillaic acid^20^. In contrast, the P450s involved in quillaic acid biosynthesis differ in *Saponaria officinalis*, with CYP716A379 exhibiting bifunctional oxidation at the C-28 and C-16α positions, and CYP72A984 oxidizing the C-23 position^21^. Regarding UGTs, members from the UGT73 family of group D are extensively proven to function as 2’-*O*-glycosyltransferase and 3’-*O*-glycosyltransferase of oleanane-type pentacyclic triterpene 3-*O*-β-D-glucopyranosiduronic acid^20–22^. Similarly, both UGT73P12 from *Glycyrrhiza uralensis* and UGT73CB3 from *Aralia elata* catalyze the 2’-*O*-glycosylation of oleanane-type pentacyclic triterpene 3-*O*-β-D-glucopyranosiduronic acid^23^. In addition, PgUGT94Q2 of the A group has been proven to transfer a glucose moiety to the C2’-OH position of the first sugar moiety on ginsenoside Rg_3_ and Ginsenoside Rd^24^. However, the enzymes involved in the multi-site hydroxylation, tiglation, and 4’-*O*-glycosylation of triterpenoids remain unknown. Therefore, elucidating the biosynthetic pathway of escin Ia (**1**) is imperative to broaden our understanding of the molecular mechanisms underlying triterpenoid biosynthesis.

Escin Ia (**1**) is structurally composed of a polyhydroxy triterpene aglycone protoaescigenin (**2)**, which bears a branched trisaccharide chain at the C-3β position and is further functionalized with an acetyl group at C-22α and a tigloyl group at C-21β^25^. Our prior genomic analysis of *A. chinensis* revealed a species-specific whole-genome duplication (WGD) event driving the duplication of triterpenoid biosynthetic gene clusters (BGCs). Within this genomic context, we previously characterized five key enzymes responsible for escin Ia (**1**) biosynthesis: AcOSC6 (β-amyrin (**3**) synthase), AcCYP716A278 (C-21β hydroxylation), AcCYP716A275 (C-16α hydroxylation), AcCSL1 (C-3β glucuronidation), and AcBAHD6 (C-22α acetylation)^26^. Mai et al., (2025) discovered a conserved saponin BGC in Sapindaceae species, which consisted of BAS (β-amyrin (**3**) synthase), CYP716A, UGT87A and CSL^27^. CSL proteins have been demonstrated to catalyze 3-*O*-glucuronosylation of triterpenoid aglycones in several plant species, including licorice^28^, spinach^29^, and horse chestnut^26^. Although we have previously characterized five enzymatic steps in the biosynthetic pathway of escin Ia (**1**), these represent discontinuous segments. Therefore, it is essential to identify the remaining biosynthetic enzymes to completely elucidate the pathway of escin Ia (**1**).

Here, we report the complete elucidation of the biosynthetic pathway of escin Ia (**1**) through the systematic characterization of the remaining critical enzymes. We identified three P450-mediated hydroxylations associated with the formation of protoaescigenin (**2**), in which both AcCYP716BX1 and AcCYP716BX2 catalyze C-22α and C-28 oxidations, while AcCYP714E67 specifically modifies the C-24 position. The glucuronopyranosyl moiety undergoes sequential glycosylation, with modifications at the 2’-*O* position mediated by AcUGT94AK1, AcUGT94AK3 or AcUGT94AK6, followed by glucosylation at the 4’-*O* position executed by AcUGT87AZ3. Subsequent to scaffold hydroxylation and glycosylation, the specialized CoA ligase (AcCCL3) activates tiglic acid (**5**) to form tigloyl-CoA (**6**), which serves as the donor substrate for C-21β tigloylation reactions catalyzed by the BAHD acyltransferase family members AcBAHD13 and AcBAHD17. Finally, heterologous reconstitution of the complete pathway in *Nicotiana benthamiana* successfully produced escin Ia (**1**).

## Results

### Bioinformatic analysis

**Fig. 1:**
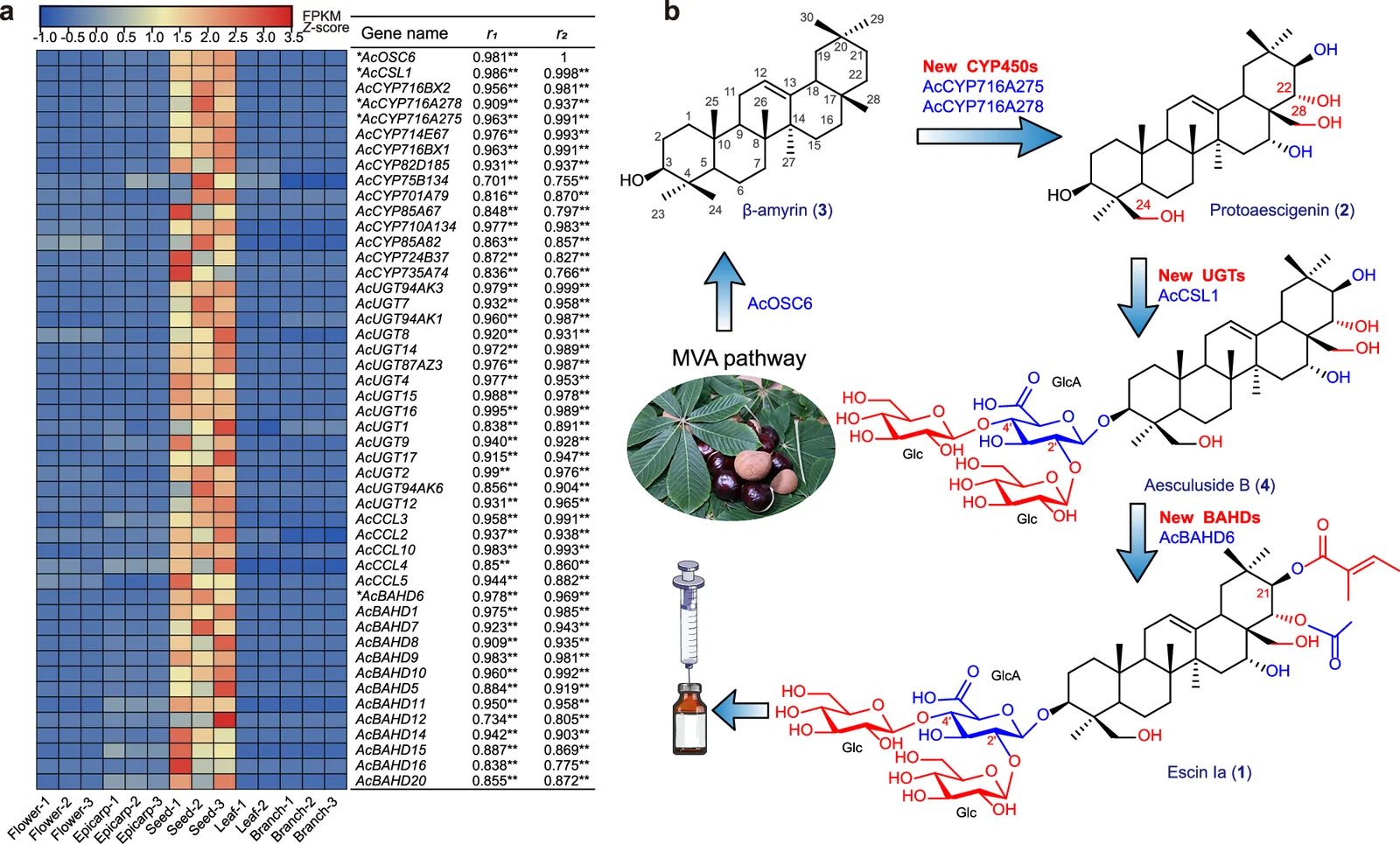
Transcriptomic analysis of candidate genes and the proposed escin Ia (1) biosynthetic pathway in *A. chinensis.* **a** Expression levels of candidate genes from the seed-specific ‘turquoise’ module. The color scale indicates relative gene expression levels based on Z-score normalized FPKM values, ranging from blue (low expression) to red (high expression). Genes marked with asterisks have been validated to be involved in escin Ia (**1**) biosynthesis. Pearson correlation coefficients (*r*_*1*_ and *r*_*2*_) indicate the correlation of gene FPKM values with escin Ia (**1**) content and *AcOSC6* expression, respectively. Statistical significance for Pearson correlation coefficients was determined using a two-tailed test, with significance defined as *p* < 0.01. **b** The proposed biosynthesis pathway of escin Ia (**1**) in *A. chinensis*. Enzymes and catalytic steps shown in blue have been previously validated^26^, while those highlighted in red represent enzymes and catalytic steps to be investigated in this study. The syringe and injectable solution icons were adapted from Servier Smart Medical Art (https://smart.servier.com/), licensed under a Creative Commons Attribution 3.0 Unported License (https://creativecommons.org/licenses/by/3.0/).

Horse chestnut accumulates escin Ia (**1**) as a major bioactive compound, with significantly higher concentrations found in seeds compared to leaves and other tissues^26^. To investigate the genetic basis of escin Ia (**1**) biosynthesis, transcriptomic analysis and weighted gene co-expression network analysis (WGCNA) were performed across five tissues: flower, epicarp, seed, leaf, and branch. A seed-specific ‘turquoise’ module was identified as the primary candidate for escin Ia (**1**) biosynthesis, as its expression profile most closely mirrored the accumulation of **1** in seeds. Conversely, other identified modules showed peak expression in non-seed tissues and lacked significant correlation with the biosynthetic pathway. The ‘turquoise’ module includes AcOSC6, AcCSL1, 14 P450s, 14 UGTs, 5 CCLs, and 13 BAHDs. Among these, AcOSC6, AcCSL1, AcCYP716A278, AcCYP716A275, and AcBAHD6 have been functionally validated to participate in escin Ia biosynthesis^26^. Co-expression analysis revealed that the Pearson correlation coefficients between the FPKM values of candidate genes in the ‘turquoise’ module and both escin Ia (**1**) content and *AcOSC6* expression were greater than 0.7 (*p* < 0.01), suggesting a strong association with escin Ia production (Fig. 1a, Supplementary Table 1).

Based on the intermediate metabolites purified from seeds of the *Aesculus* genus^30,31^, we propose that β-amyrin (**3**) is hydroxylated by P450s (AcCYP716A278, AcCYP716A275, and other unidentified P450s) at the C-21β, C-16α, C-22α, C-24, and C-28 positions to form protoaescigenin (**2)**. Subsequently, **2** is converted to 3β-*O*-{β-D-glucopyranosyl-(1 → 4)-[β-D-glucopyranosyl-(1 → 2)]-β-D-glucuronopyranosyl acid}-protoaescigenin (aesculuside B, **4**) through the assembly of the trisaccharide moiety at the C-3β position. This transformation is mediated by AcCSL1^26^ and some unknown UGTs. The terminal biosynthetic phase features acyl modifications through the concerted action of AcBAHD6 and additional BAHD acyltransferases, facilitating both acetylation and tigloylation reactions to produce **1** (Fig. 1b).

### Biosynthesis of the protoaescigenin (2) scaffold

**Fig. 2:**
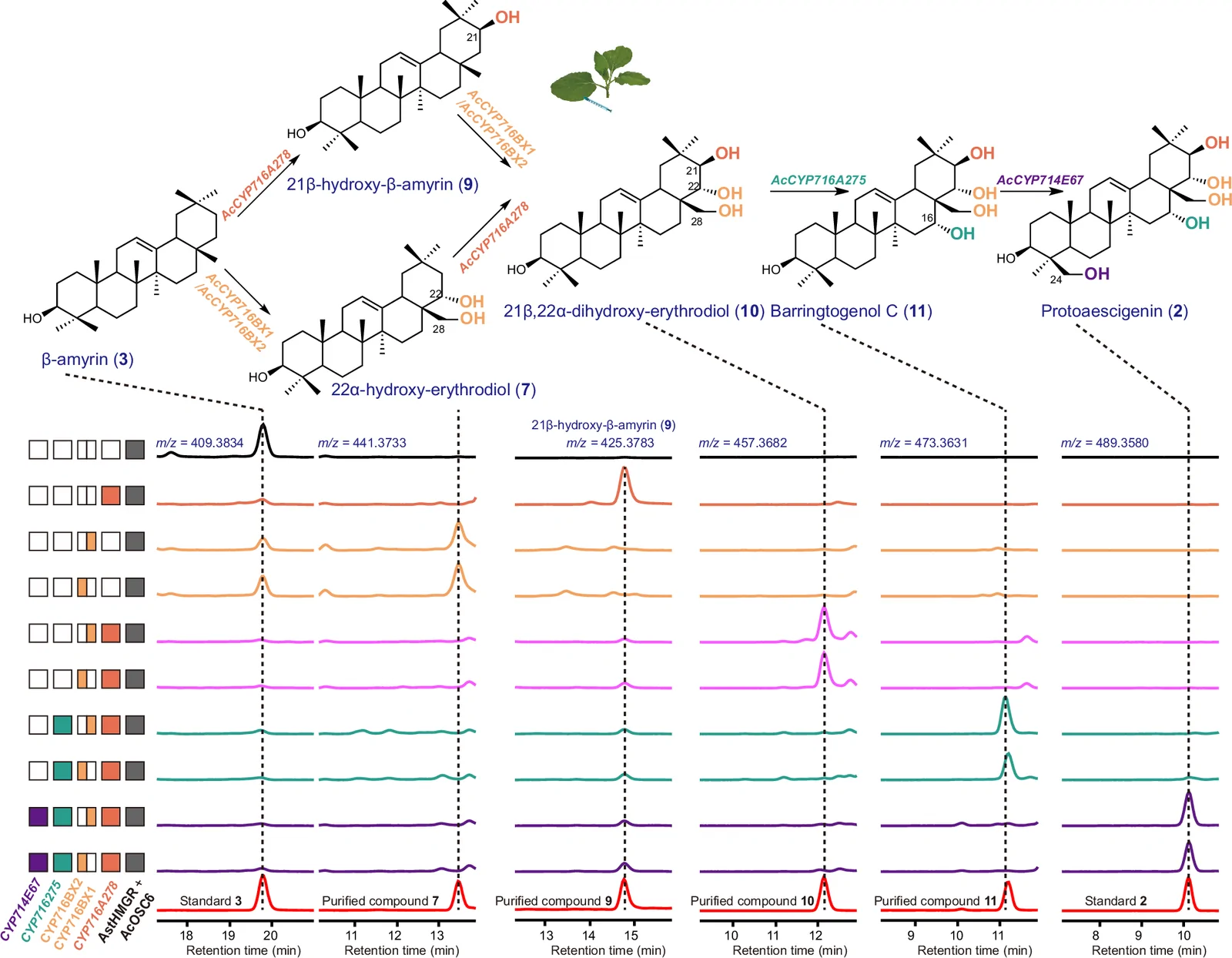
Progressive elucidation of protoaescigenin (2) biosynthetic pathway. Extracted ion chromatograms (EICs) for each compound (with corresponding *m/z* values) were obtained from *N. benthamiana* transiently co-expressing candidate biosynthetic genes from *A. chinensis*. All compounds were tested through LC-QTOF-MS. Assays for the biosynthesis of **2** are representative of *n* = 3 independent biological replicates with similar results. The syringe schematic was created by Luz Eugenia Velasquez and obtained from Vecteezy (Vector Art ID: 14175787, Vecteezy Free License, https://www.vecteezy.com/vector-art/14175787-syringe-medical-drug).

To elucidate the unresolved enzymatic steps governing the C-22α, C-24, and C-28 hydroxylations in **2** biosynthesis, we systematically characterized candidate P450s encoded within the previously identified BGCs of (AcCluster1 and AcCluster2) in the *A. chinensis* genome^26^. Among the homologous gene pairs, *AcCYP716BX1* and *AcCYP716BX2*, sharing 89.80% amino acid sequence identity and localizing in AcCluster1 and AcCluster2, respectively, displayed a seed-predominant expression pattern and were therefore initially selected for catalytic analysis (Supplementary Tables 1–2). Transient co-expression assays in *N. benthamiana* revealed that the combinatorial expression of *AcCYP716BX1* with β-amyrin (**3**) biosynthetic genes (*AstHMGR* + *AcOSC6*) yielded two new products (*m/z* 441.3733 at 13.4 min and *m/z* 455.3525 at 12.9 min) (Fig. 2 and Supplementary Figs. 1–3). These products were purified and structurally identified as 22α-hydroxy-erythrodiol (**7**) and 22α-hydroxy-oleanic acid (**8**) via NMR and LC-MS analysis (Supplementary Figs. 4–16 and Supplementary Table 3). These results suggest a sequential oxidation pathway: C-22 and C-28 hydroxylation of β-amyrin to yield compound **7**, followed by C-28 carboxylation to produce compound **8**. Notably, *AcCYP716BX2* exhibited identical catalytic activity to *AcCYP716BX1*, implying a functional redundancy likely resulting from the whole-genome duplication^26^. Given that compound **8** is not an intermediate in **1** biosynthesis, our combined functional and structural data demonstrate that either AcCYP716BX1 or AcCYP716BX2 mediates the C-22α and C-28 hydroxylations in the biosynthetic pathway of **1**. Extended pathway reconstitution incorporating AcCYP716A278 (C-21β hydroxylase, which catalyzes the formation of 21β-hydroxy-β-amyrin, **9**) enabled the biosynthesis of 21β,22α-dihydroxy-erythrodiol (**10,** *m/z* 457.3682 at 12.2 min), structurally validated through NMR analysis of compounds purified from large-scale *N. benthamiana* infiltrations (Fig. 2, Supplementary Figs. 17–24 and Supplementary Table 4). Full pathway integration (*AstHMGR*, *AcOSC6*, *AcCYP716A278*, *AcCYP716A275*, and either *AcCYP716BX1* or *AcCYP716BX2*) successfully generated barringtogenol C (**11**), as evidenced by LC-MS alignment of retention times and mass fragmentation patterns with those of an in-house authenticated reference standard^31^ (Fig. 2 and Supplementary Fig. 25).

To resolve the terminal C-24 hydroxylation step, each of the nine additional P450s from the seed-specific ‘turquoise’ module was co-expressed with the gene combination producing **11** in *N. benthamiana*. LC-MS profiling identified AcCYP714E67 as the C-24 hydroxylase generating **2** (Fig. 2 and Supplementary Figs. 26–27). Enzymatic validation through the co-infiltration of the standard **11** with *AcCYP714E67* confirmed this function (Supplementary Fig. 28).

To further decipher the cooperative biosynthetic cascade from β-amyrin (**3**) to protoaescigenin (**2**), we performed multiplexed transient expression matrices using various gene combinations in *N. benthamiana*. LC-MS analysis revealed no detectable 24-hydroxy-β-amyrin, suggesting that *AcCYP714E67* lacks hydroxylation activity toward β-amyrin at the C-24 position (Supplementary Fig. 29). Additionally, it also failed to catalyze the C-24 hydroxylation of 16α-hydroxy-β-amyrin (**23**). However, with the exception of compounds **3** and **23**, AcCYP714E67 was capable of hydroxylating other tested intermediates at the C-24 position. In contrast, AcCYP716A275, AcCYP716A278, and AcCYP716BX1/2 displayed broader substrate promiscuity, as they were capable of catalyzing regioselective hydroxylations on all tested β-amyrin derivatives with varying degrees of prior hydroxylation (Supplementary Fig. 30). Various gene combinations yielded seven new intermediate products (**16**-**22**). Based on the mass spectrometric patterns and diagnostic fragments, the chemical structures of **16**-**22** were putatively identified (Supplementary Figs. 31–37). Collectively, the substrate promiscuity of AcCYP716A275, AcCYP716A278, AcCYP716BX1/2 and AcCYP714E67, results in a complex, grid-like metabolic network for the biosynthetic pathway from β-amyrin (**3**) to protoaescigenin (**2**).

To unambiguously eliminate the possibility of functional redundancy contributed by endogenous *N. benthamiana* enzymes, we performed stringent ‘leave-one-out’ combinatorial omission controls in *N. benthamiana* leaves. LC-MS analysis indicated that **2** was exclusively produced in the two gene pools containing *AstHMGR*, *AcOSC6*, *AcCYP716A278*, *AcCYP716BX1*/*2*, *AcCYP716A275*, and *AcCYP714E67*. Notably, the omission of any single *P450* gene from either pool completely abrogated the production of **2**, confirming that no host-plant P450s can complement or bypass the required steps (Supplementary Fig. 38). Furthermore, the independent functions of the newly identified enzymes (AcCYP716BX1, AcCYP716BX2, and AcCYP714E67) were robustly validated using in vitro *Saccharomyces cerevisiae* microsomal assays, yielding results consistent with those observed in *N. benthamiana* (Supplementary Figs. 39–40). Collectively, transient co-expression of AcCYP716BX1 or AcCYP716BX2 with associated gene modules in *N. benthamiana* leaves produced **2** at a yield of 1294.73 µg/g (dry weight, DW) and 844.02 µg/g (DW), respectively (Supplementary Fig. 41).

### Addition of the C-3β sugar chain

Following the establishment of the enzymatic framework for **2** biosynthesis, subsequent steps involving stereospecific C-3β glycosylation were systematically decoded. The pathway requires the sequential installation of a glucuronopyranosyl moiety at the C-3β position of **2**, followed by two glucopyranosyl units at the 2’-*O* and 4’-*O* positions of this initial glucuronopyranosyl moiety. While AcCSL1-mediated 3β-*O*-glucuronidation of **2** was previously characterized^26^, the enzymes responsible for the subsequent sugar chain elongation remain undefined. Genomic interrogation of seed-expressed ‘turquoise’ module transcripts identified 15 UGT candidates, of which eight genes were successfully cloned and functionally expressed.

**Fig. 3:**
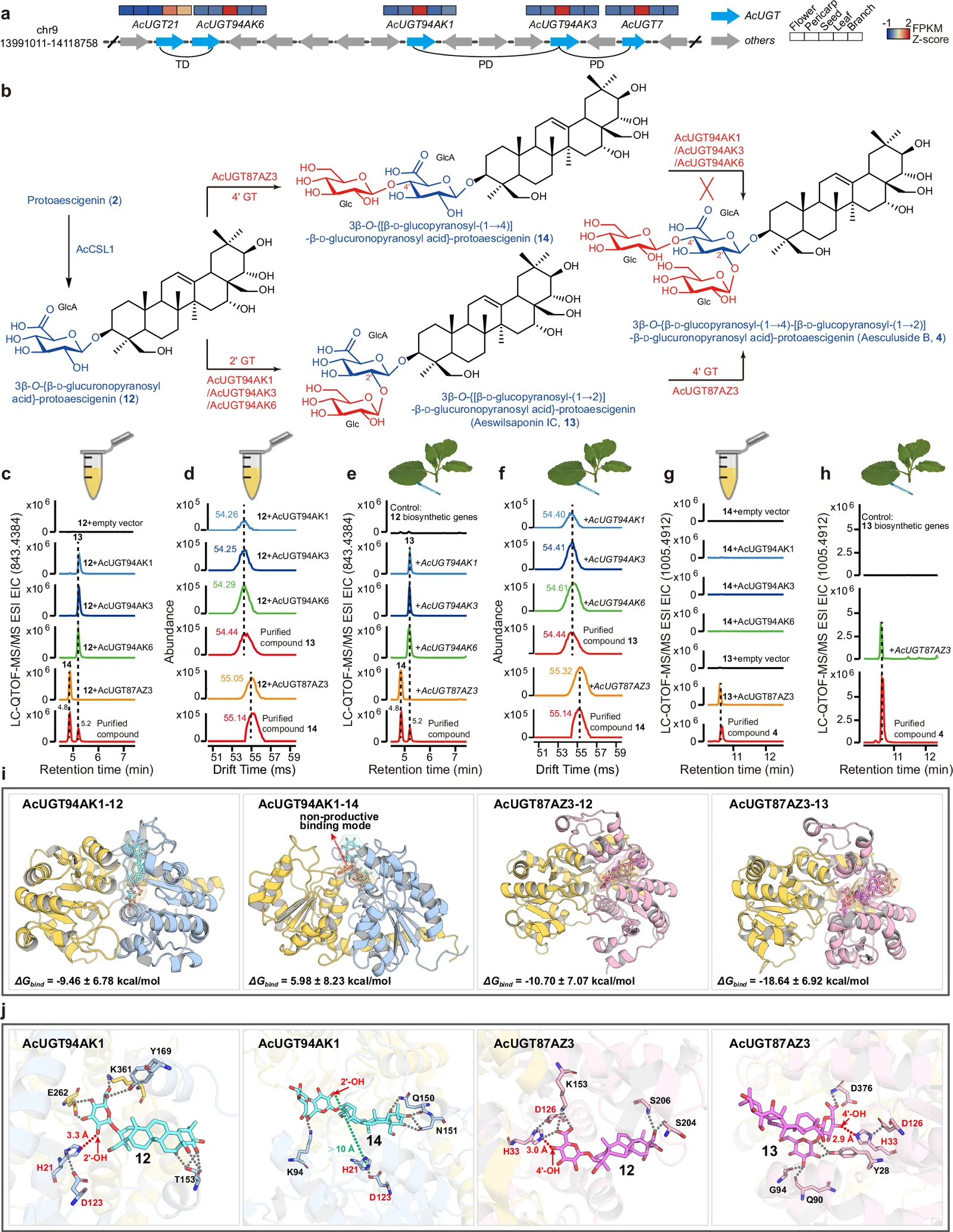
Genetic, biochemical, and structural basis of the branched C-3 sugar chain assembly in escin Ia (1) biosynthesis. **a** Genomic localization and expression profile of *AcUGTs*. *AcUGT94AK1*, *AcUGT94AK3*, *AcUGT94AK6*, and two other *AcUGTs* form a cluster on chromosome 9 and exhibit seed-specific expression. TD: tandem duplication; PD: proximal duplication. The color scale indicates relative expression levels of *AcUGTs* based on Z-score normalized FPKM values, ranging from blue (low expression) to red (high expression). **b** Biosynthetic pathway from **2** to **4**. The “×“ symbol indicates that no catalytic activity was detected. **c** In vitro assays showed that **12** was catalyzed by AcUGT94AK1/3/6 and AcUGT87AZ3 to generate a new product, which was identified as **13** (*m/z* 843.4384) and **14** (*m/z* 843.4384), respectively. **d** Isomers **13** and **14** were further distinguished by DTIM-MS analysis. This analysis confirmed that, in vitro, AcUGT94AK1/3/6 catalyzed the glycosylation of **12** at the C2’-OH position, while AcUGT87AZ3 catalyzed the glycosylation of **12** at the C4’-OH position. **e**,**f** In infiltrated *N. benthamiana*, co-expression of genes involved in **12** biosynthesis (*AstHMGR, AcOSC6, AcCYP716A278, AcCYP716BX1, AcCYP716A275, AcCYP714E67, AcCSL1*) with either *AcUGT94AK1*, *AcUGT94AK3* or *AcUGT94AK6* resulted in the production of **13**, while co-expression of these seven genes with *AcUGT87AZ3* led to the production of **14**. **g** In vitro assays revealed that AcUGT94AK1, AcUGT94AK3 or AcUGT94AK6 did not catalyze **14** to produce **4**, while AcUGT87AZ3 catalyzed **13** to generate **4**. **h** Co-expression of eight genes involved in compound **13** biosynthesis (*AstHMGR, AcOSC6, AcCYP716A278, AcCYP716BX1, AcCYP716A275, AcCYP714E67, AcCSL1, AcUGT94AK1*) with *AcUGT87AZ3* resulted in the production of **4** in infiltrated *N. benthamiana*. **i** Overlay of the initial docking poses and MD-equilibrated conformations for the AcUGT94AK1-**12**, AcUGT94AK1-**14**, AcUGT87AZ3-**12** and AcUGT87AZ3-**13** complexes with calculated binding energies. In AcUGT94AK1 and AcUGT87AZ3, the C-terminal and N-terminal domains are colored yellow and blue/pink, respectively. *∆G*_*bind*_ values were calculated using the MM/GBSA method based on 200 ns MD trajectories. **j** Close-up views of the catalytic center. Red and green dotted lines indicate the distances between the acceptor hydroxyl group and the catalytic histidine residue. Assays for the biosynthesis of **4** are representative of *n* = 3 independent biological replicates with similar results, both in vitro and in vivo. The centrifuge tube icon was created by Helicase 11 (CC-BY 4.0, https://creativecommons.org/licenses/by/4.0/); and the syringe schematic was by Luz Eugenia Velasquez via Vecteezy (Vector Art ID: 14175787, Vecteezy Free License, https://www.vecteezy.com/vector-art/14175787-syringe-medical-drug).

Using 3β-*O*-{β-D-glucuronopyranosyl acid}-protoaescigenin (**12**) as the substrate, enzymatic assays revealed that four of the eight tested AcUGTs generated a new peak with an identical *m/z* value of 843.4384 and retention time of 8.2 min, matching those of the two isomers: 3β-*O*-{[β-D-glucopyranosyl-(1 → 2)]-β-D-glucuronopyranosyl acid}-protoaescigenin (aeswilsaponin IC, **13**) and 3β-*O*-{β-D-glucopyranosyl-(1 → 4)-β-D-glucuronopyranosyl acid}-protoaescigenin (**14**) (Supplementary Fig. 42). While initial attempts to separate these isomers using an extended 35 min elution gradient were unsuccessful (Supplementary Fig. 43), they were unambiguously differentiated by MS/MS fragmentation stability, a third optimized elution gradient along with Drift Tube Ion Mobility Mass Spectrometry (DTIM-MS). Specifically, **13** showed a shorter drift time than **14** (by about 0.7 ms). In terms of fragmentation stability, **13** required higher collision energy (CE) than **14**; at CE = 60, the relative intensity of the precursor ion [M-H]^⁻^ for **13** remained higher than that of its product ion [M-H-Glc]^⁻^. In contrast, for **14**, the precursor ion intensity was already lower than the product ion even at a lower CE of 40. Furthermore, the retention times of **13** and **14** were completely resolved under the third elution gradient. Based on these distinct differences in drift time, fragmentation, and retention time, we confirmed that AcUGT94AK1, AcUGT94AK3, and AcUGT94AK6 catalyzed the regioselective 2’-*O*-glucosylation to form **13**, whereas AcUGT87AZ3 mediated 4’-*O*-glucosylation to yield **14** (Fig. 3c-d and Supplementary Figs. 44–45). Functional validation through *N. benthamiana* transient co-expression assays (*AstHMGR*, *AcOSC6*, *AcCYP716A278*/*275*/*BX1*, *AcCYP714E67, AcCSL1*) with individual AcUGTs confirmed this regioselective glycosylation activity in vivo (Fig. 3e–f and Supplementary Figs. 45–46).

Sequential glycosylation cascades were successfully reconstituted by incubating **13** with AcUGT87AZ3, which afforded the trisaccharide conjugate **4** (*m/z* 1005.4912 at 10.6 min). The identity of this product was unambiguously validated via LC-MS analysis and comparison with an authenticated reference standard (Fig. 3g and Supplementary Figs. 47–48). Conversely, no product was detected from the reaction of compound **14** with AcUGT94AK1, AcUGT94AK3, or AcUGT94AK6 (Fig. 3g and Supplementary Fig. 49). Furthermore, co-infiltration of *AcUGT87AZ3* with genes responsible for the biosynthesis of **13** successfully reconstructed the production of **4** in *N. benthamiana* (Fig. 3h and Supplementary Fig. 48). Convergent evidence from both in vivo and in vitro assays establishes that AcUGT94AK1/3/6 and AcUGT87AZ3 are 2’-*O* and 4’-*O* glucosyltransferases, respectively. Crucially, our results demonstrate that 2’-*O*-glucosylation is a prerequisite for subsequent 4’-*O*-glucosylation during the enzymatic assembly of the C-3β trisaccharide moiety of **1**. Based on the UGT classification criteria^32^, phylogenetic analysis classified the three 2’-*O*-glucosyltransferase (AcUGT94AK1, AcUGT94AK3 and AcUGT94AK6) within subgroup A of the glycosyltransferase GT1 family (Supplementary Fig. 50). This subgroup encompasses functionally characterized enzymes such as PgUGT94Q2 from *Panax ginseng* and Pq-O-UGT2 from *P. quinquefolius*, both demonstrated to mediate the specific 2’-*O*-glucosylation of dammarane-type triterpenoids^24,33^. In contrast, the 4’-*O*-glucosyltransferase AcUGT87AZ3 belonged to subgroup J, a phylogenetically distinct clade that remained functionally underexplored in triterpenoid glycosylation research.

To elucidate the molecular mechanism underlying the glycosylation sequence, molecular dynamics (MD) simulations were conducted to compare the catalytic reactivity of candidate AcUGTs. Plant UGTs typically utilize S_N_2-like direct displacement to catalyze glycosyl transfer^34^. A histidine (His) and an aspartic acid (Asp) near the acceptor binding pocket at the N-terminal domain of UGT have been proven to play crucial roles in the recognition and specificity of the substrate^34,35^. Given that AcUGT94AK1, AcUGT94AK3, and AcUGT94AK6 share over 90% sequence similarity (Supplementary Table 5), AcUGT94AK1 was selected as a representative model for the MD simulations. Structural models of AcUGT94AK1 and AcUGT87AZ3 were generated using AlphaFold3, and sequence alignment identified H21/D123 and H33/D126 as their respective catalytic dyads (Supplementary Fig. 51). Complexes of AcUGT94AK1 with **12**/**14** and AcUGT87AZ3 with **12**/**13** were subjected to docking followed by MD simulations. Although classical MD simulations cannot directly estimate reaction barriers as QM/MM methods do, productive binding conformations defined by the proximity and stability between the acceptor hydroxyl group and the catalytic His residue are widely used in parallel to evaluate UGT catalytic competence and regioselectivity^36–40^. Notably, in the AcUGT94AK1-**14** complex, ligand **14** exhibited pronounced fluctuations and progressively drifted toward the solvent-exposed region, indicating failure to maintain a productive binding mode within the acceptor pocket, consistent with an unfavorable binding free energy (*ΔG*_*bind*_ = +5.98 ± 8.23 kcal/mol) (Fig. 3i-j and Supplementary Fig. 52). Consequently, the O–N distance between the C2′-OH oxygen of **14** and the His ε-nitrogen in the AcUGT94AK1-**14** complex remained above 10 Å throughout the simulation (Supplementary Fig. 53). These MD simulation results are consistent with the in vitro validations, collectively demonstrating that AcUGT94AK1 cannot catalyze the 2’-*O*-glucosylation of **14**. In contrast, the AcUGT94AK1-**12** complex adopted a stable binding state with favorable affinity (*ΔG*_*bind*_ = −9.46 ± 6.78 kcal/mol), in which the O-N distance between the C2′-OH oxygen and the catalytic His ε-nitrogen rapidly shortened and stabilized at less than 3.5 Å. Similarly, both AcUGT87AZ3-**12** and AcUGT87AZ3-**13** complexes maintained stable conformations with catalytic O-N distances consistently below 3.5 Å, indicating productive catalytic geometries (Fig. 3i, j and Supplementary Figs. 52, 53). Together, these MD simulations reveal that AcUGT94AK1 exhibits more stringent substrate specificity than AcUGT87AZ3, selectively catalyzing the 2′-O-glycosylation of **12**, but not **14**. Therefore, this difference in substrate specificity elucidates why 2′-O-glycosylation precedes 4′-O-glycosylation in the biosynthesis of **4**.

### Formation of tigloyl-CoA (6)

Through systematic investigation, we comprehensively elucidated the biosynthetic pathway governing the formation of **2** and its C-3β trisaccharide side chain (Figs. 2 and 3). Having previously identified the acetyltransferase^26^, we subsequently focused on identifying the enzymes responsible for the formation of the tigloyl group at the C-21β-OH position of **1**. However, due to constraints imposed by the limited availability and prohibitive cost of **6**, we prioritized elucidating the biosynthetic origin of this critical CoA donor.

**Fig. 4:**
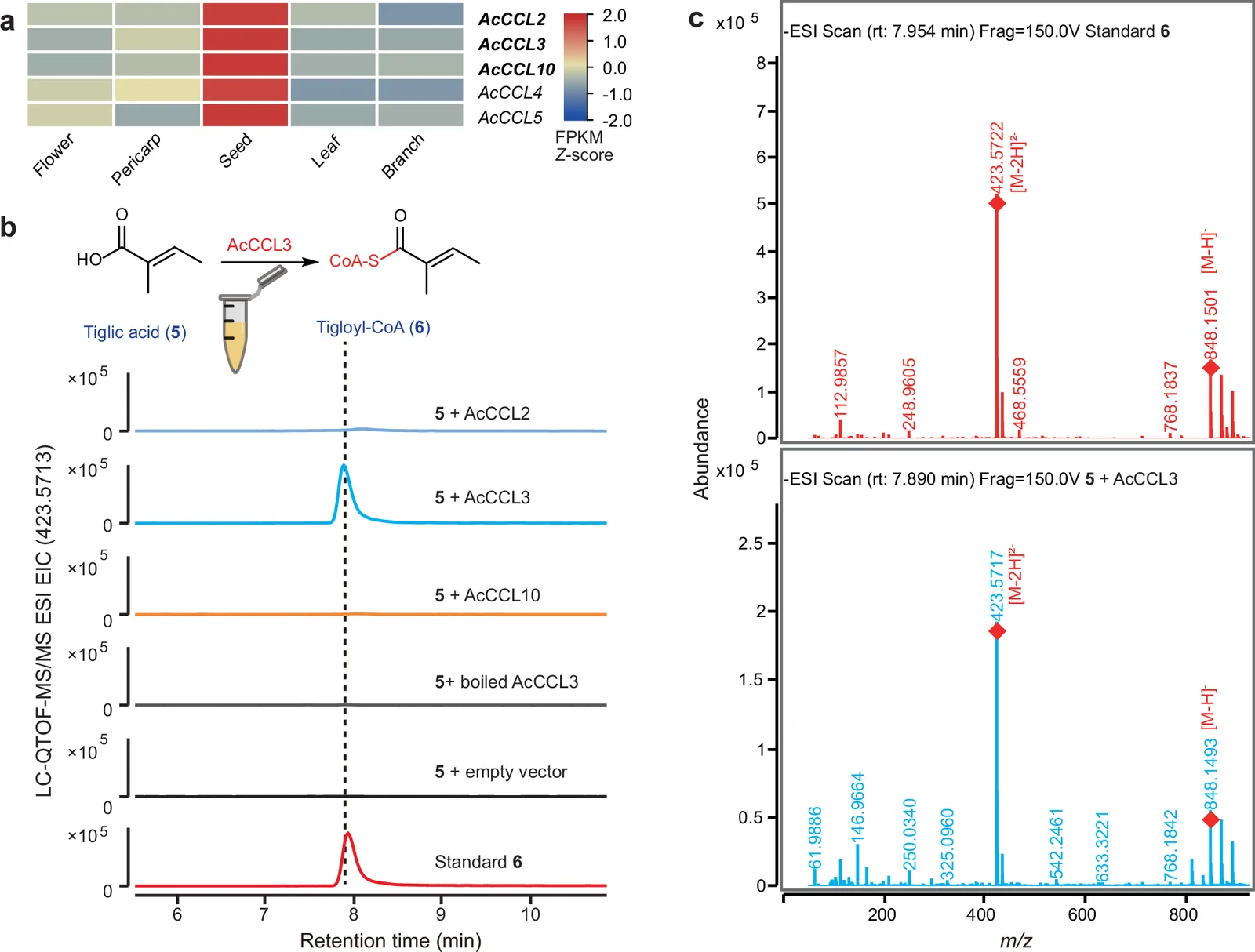
Functional characterization and biochemical validation of AcCCL3 involved in tigloyl-CoA (6) biosynthesis. **a** Candidate *AcCCL* genes exhibited seed-specific expression. The color scale indicates relative expression levels of *AcCCLs* based on Z-score normalized FPKM values, ranging from blue (low expression) to red (high expression). AcCCLs that were successfully expressed as proteins were marked in bold font. **b** In vitro enzymatic assay showing that AcCCL3 catalyzed the conversion of **5** to **6**. **c** The mass spectra of the standard **6** and the corresponding product generated in vitro by AcCCL3 using **5** as the substrate. Assays for the biosynthesis of **6** are representative of *n* = 3 independent biological replicates with similar results. The centrifuge tube icon was created by Helicase 11 (CC-BY 4.0, https://creativecommons.org/licenses/by/4.0/).

Isotopic labeling studies demonstrate that **5** is biosynthetically derived from the catabolism of the branched-chain amino acid *L*-isoleucine, followed by CoA activation mediated by CCLs to generate **6**^41,42^. Co-expression network analysis identified five *AcCCL* genes within the ‘turquoise’ module, three of which, including *AcCCL2*, *AcCCL3* and *AcCCL10* were cloned and functionally characterized (Fig. 4a). AcCCL3 functioned as a tiglic acid-activating enzyme that catalyzed the ligation of **5** with CoA to form **6** ([M-2H]^2-^, *m/z* 423.5713 at 7.9 min). This activity was validated through LC-MS metabolic profiling by comparison of a commercial standard (Fig. 4b, c). Following the CCL classification criteria from Martin^43^, phylogenetic analysis demonstrated that AcCCL3 belonged to the group IV (Supplementary Fig. 54). In this group, HlCCL4 from *Humulus lupulus* was proven to ligate a CoA group onto 2-methylbutyric acid^44^. Thus, AcCCL3 was selected for further studies investigating the roles of BAHD acyltransferases in related biochemical pathways.

### Addition of C-21β tigloyl and reconstruction of escin Ia (1)

**Fig. 5:**
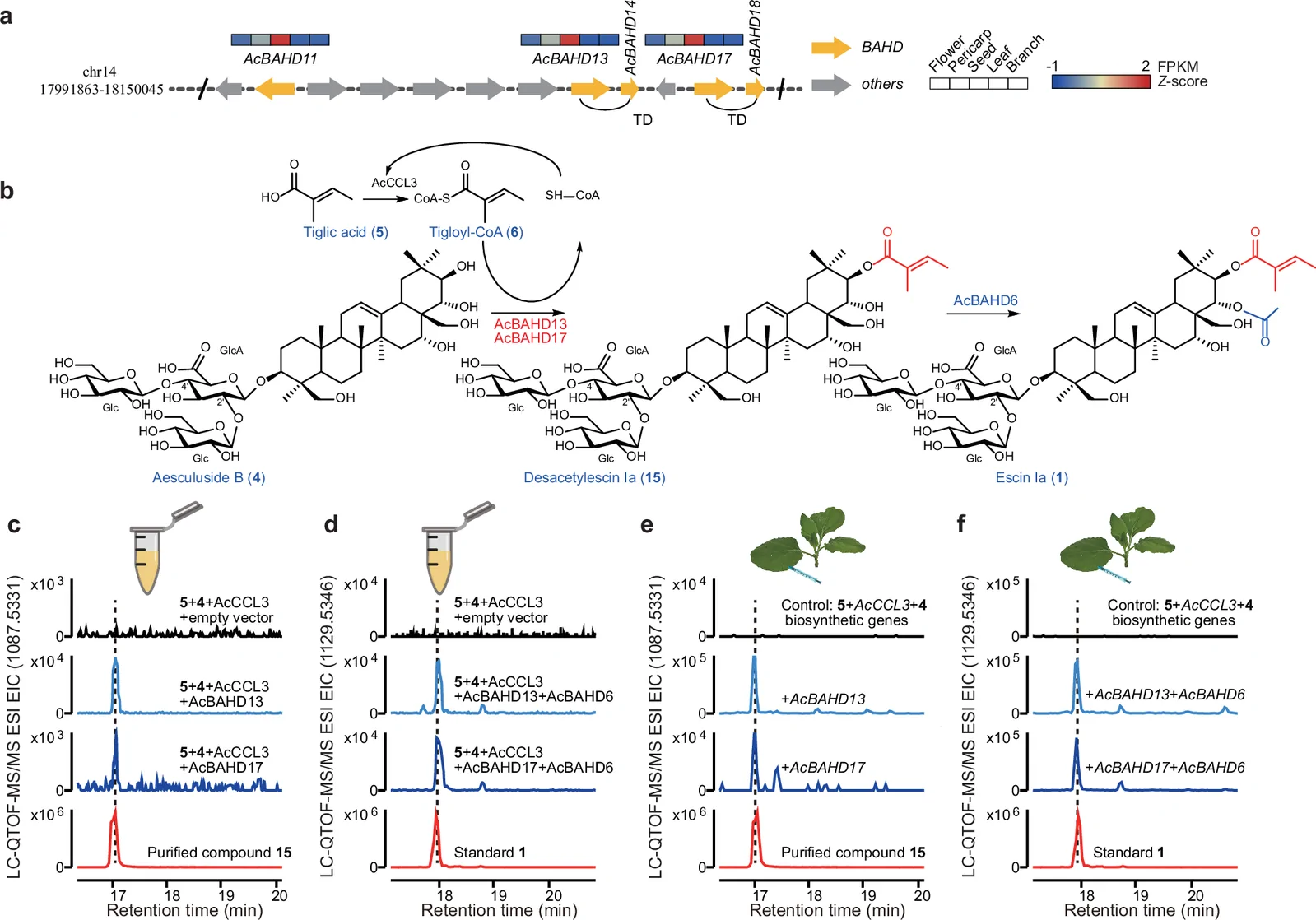
Genetic architecture and the enzymatic mechanisms of acyl side chain assembly in escin Ia (1) biosynthesis. **a** Genomic organization of AcBAHDs on chromosome 14. *AcBAHD13* and *AcBAHD17*, along with three other *AcBAHDs*, form a biosynthetic gene cluster. TD: tandem duplication. The color scale indicates relative expression levels of *AcBAHDs* based on Z-score normalized FPKM values, ranging from blue (low expression) to red (high expression). **b** Biosynthetic pathway from **4** to **1**. **c** In vitro assays showed that AcBAHD13 or AcBAHD17, in the presence of AcCCL3 and tiglic acid (**5**), catalyzed the tigloylation of **4** to form **15**. **d** Further in vitro reconstitution showing that the addition of AcBAHD6 to the reaction mixture (AcBAHD13/17, AcCCL3, and **5**) enables the conversion of **4** to **1**. **e** Co-expression of either *AcBAHD13* or *AcBAHD17* with *AcCCL3*, tiglic acid (**5**) and nine genes involved in aesculuside B (**4**) biosynthesis (*AstHMGR, AcOSC6, AcCYP716A278, AcCYP716BX1, AcCYP716A275, AcCYP714E67, AcCSL1, AcUGT94AK1*, and *AcUGT87AZ3*) generated **15**. **f** Co-expression of *AcBAHD6* with the system of (**e**) led to the production of **1** in infiltrated *N. benthamiana*. Assays for the biosynthesis of **1** are representative of *n* = 3 independent biological replicates with similar results. The centrifuge tube icon was created by Helicase 11 (CC-BY 4.0, https://creativecommons.org/licenses/by/4.0/); and the syringe schematic was by Luz Eugenia Velasquez via Vecteezy (Vector Art ID: 14175787, Vecteezy Free License, https://www.vecteezy.com/vector-art/14175787-syringe-medical-drug).

To identify the enzyme responsible for transferring the tigloyl group to the C-21β hydroxyl group of **4**, genomic analyses revealed a BAHD gene cluster comprising five members (AcBAHD11, 13, 14, 17, and 18). DupGen_finder analysis demonstrated distinct evolutionary origins: AcBAHD13/14 and AcBAHD17/18 emerged via tandem duplication events, while AcBAHD11 was identified as a paralog of AcBAHD6 (previously characterized as the C-22α acetyltransferase) arising from a WGD event (Fig. 5a). Among these, only AcBAHD11, 13, and 17 retained complete BAHD domains and displayed seed-specific expression, establishing them as primary candidates. Further, AcBAHD13 and 17 were successfully cloned for functional validation.

In vitro enzymatic assays using recombinant proteins demonstrated that AcBAHD13 and AcBAHD17 catalyzed the formation of a new product when incubated with **4,** **5** and AcCCL3. LC-MS analysis identified the catalytic product as desacetylescin Ia (**15**) (*m/z* 1087.5331 at 17.0 min), which was unequivocally confirmed by comparison with authentic standards (Fig. 5b–c and Supplementary Fig. 55). Furthermore, the above reaction system was incubated with AcBAHD6, an enzyme previously characterized as a C-22α-acetyltransferase^26^, leading to the production of **1** (*m/z* 1129.5436 at 17.9 min, Fig. 5d and Supplementary Fig. 56). Phylogenetic analysis revealed that AcBAHD6, 13 and 17 belonged to clade 3 (Supplementary Fig. 57). This clade contains several BAHDs known to recognize short-chain acyl-CoA thioesters, such as isobutyryl-CoA, isovaleryl-CoA, 2-methylbutyryl-CoA, and acetyl-CoA^45–47^.

Transient co-expression assays in *N. benthamiana* further corroborated the enzymatic activity. The co-expression of *AstHMGR*, *AcOSC6*, *AcCYP716A278*/*275*/*BX1*, *AcCYP714E67*, *AcCSL1*, *AcUGT94AK1*, *AcUGT87AZ3* and *AcCCL3* with either *AcBAHD13* or *17*, supplemented with **5**, consistently yielded **15** (*m/z* 1087.5331 at 17.0 min; Fig. 5e). Convergent evidence from both in vitro and in vivo assays robustly established that AcBAHD13 and AcBAHD17 are genuinely responsible for the C-21β tigloylation of **1**. Notably, the incorporation of AcBAHD6 into the same system (containing either AcBAHD13 or AcBAHD17) resulted in the production of **1** (Fig. 5f, Supplementary Figs. 56, 58–59). Furthermore, we evaluated the levels of **1** and key intermediate metabolites across various gene combinations in *N. benthamiana*. Initially, the content of **12** was higher when expressing *AcCYP716BX1* compared to *AcCYP716BX2* (2,487.37 vs. 2,140.72 µg/g, respectively). Using the *AcCYP716BX1*-based combination to further stack *AcUGT*s, we found that the yield of **4** peaked at 6,123.06 µg/g in the group IV (harboring *AcUGT94AK3*), which was significantly higher than that in group V (*AcUGT94AK6*) and III (*AcUGT94AK1*) (*P* < 0.05). Among the **1**-producing groups, the accumulation of **1** reached a maximum of 122.55 µg/g in group X (containing *AcUGT94AK3* and *AcBAHD17*), significantly outperforming all other combinations (*P* < 0.05). This was followed by groups VII, XI, IX, VI, and VIII, yielding 67.10, 64.31, 35.28, 32.57, and 31.64 µg/g, respectively (Supplementary Fig. 59).

## Discussion

Advancements in sequencing technologies, improved assembly techniques, and reduced sequencing costs have significantly facilitated the elucidation of enzymatic activities involved in triterpenoid biosynthesis^48–50^. Co-expression analyses, coupled with the identification of BGCs, enable the rapid discovery of enzyme-encoding genes associated with these pathways^51,52^. Notably, we identified a WGD event unique to *A. chinensis*, which contributed to the lineage-specific accumulation of aescins^26^. Within these clusters, *AcCYP716BX1* and its paralog *AcCYP716BX2*, both originating from a WGD event, function as isoenzymes that catalyze the oxidation of β-amyrin at the C-22 and C-28 positions to yield 22α-hydroxy-erythrodiol or 22α-hydroxy-oleanolic acid. Their functional redundancy supports the hypothesis that the *Aesculus*-specific WGD was a pivotal driver for the high-level accumulation of **1** in seeds. Several studies have demonstrated that members from the CYP716A subfamily can catalyze the sequential three-step oxidation at the C-28 position of the β-amyrin skeleton. Previously, *Arabidopsis* CYP716A2 has been demonstrated to catalyze C-22α or C-28 monohydroxylation of α-amyrin and β-amyrin^53^, while CYP72A61v2^54^ and CYP72A566^55^ are known to catalyze the C-22β hydroxylation of the β-amyrin skeleton. However, no P450s capable of dual C-22α and C-28 hydroxylation of the β-amyrin skeleton to form a dihydroxylated product have been reported until now. Thus, the characterization of AcCYP716BX1 and AcCYP716BX2 uncovers a distinct evolutionary and catalytic trajectory for multi-position tailoring in triterpenoid pathways.

Previous studies have shown that CYP93E1/2/9 from leguminous plants^54,56,57^, CYP749A63 from *Crataegus pinnatifida*^58^, and CYP93A220 from *Ilex asprella*^59^ can catalyze the C-24 hydroxylation of the oleanane skeleton. This study is the first to report that a member of the CYP714E subfamily functions as a C-24 hydroxylase of the oleanane skeleton. As reported, CYP714E19 from *Centella asiatica* catalyzed the C-23 hydroxylation of oleanolic acid and ursolic acid^60^, and CYP714E52 from *Quillaja saponaria* was involved in the C-23 carbonylation of echinocystic acid^20^. Consequently, these findings not only advance our understanding of oleanane C-24 hydroxylases but also elucidate the functional diversity of the CYP714E subfamily.

In recent years, there has been an increasing number of complete elucidations of the metabolic pathways of plant-derived natural products with clinical applications^61,62^. A growing body of research has discovered that target products often do not arise through a simple linear metabolic process^63^. This study provides a comprehensive framework for elucidating the complex polyhydroxylation mechanisms of barrigenol-type triterpenoids. Notably, based on the profiles of the isolated intermediates (such as **7,** **8** and **11**) and the results of multiplexed transient expression assays, the core biosynthetic steps from β-amyrin (**3**) to protoaescigenin (**2**) do not strictly follow a single, channeled pathway but instead operate as a highly plastic metabolic grid. Driven by the promiscuous nature of the AcCYP716 and AcCYP714 families, the sequential hydroxylations at C-16α, C-21β, C-22α, C-24, and C-28 proceed via complex, interconnected grid routes. Evolving such non-linear, resilient catalytic networks for specialized metabolism represents a classic metabolic hedging strategy in plants. The core ecological significance of this architecture lies in maximizing chemical diversity and metabolic robustness, thereby enhancing the evolutionary fitness and environmental adaptation of the species^64,65^.

Glycosylation is a crucial modification that enhances the stability, water solubility, and pharmacological activities of triterpenoids^66,67^. A defining feature of **1** is its branched trisaccharide chain, composed of a glucuronic acid core with glucose units attached at the 2’-*O* and 4’-*O* positions. Elucidating the enzymes involved in this modification is of significant interest. Previously, our lab identified AcCSL1 as a 3-*O*-glucuronosyltransferase^26^. In this study, we verified that AcUGT94AK1/3/6 from group A functions as the 2’-*O*-glucosyltransferase in the biosynthesis of **1**. Interestingly, our findings demonstrate that UGTs from different groups (UGT94: group A; UGT73: group D) can exhibit convergent catalytic activities. Phylogenetic analysis suggests a common ancestry between group A and D, supporting our hypothesis that 2’-*O*-glycosyltransferase activity was inherited from a shared ancestor and subsequently underwent independent evolution across species, resulting in significant sequence divergence while retaining ancestral function. While many UGTs have been demonstrated to catalyze triterpene glycosylation at various positions^66^, 4’-*O-glucosylation* remains poorly understood. To date, only AsTG1 has been reported to catalyze this specific reaction, which is a glycosyl hydrolase (GH) rather than a UGT^68^. Thus, no UGTs have been proven to catalyze the 4’-*O*-glucosylation of triterpenoids until now. Although members of the UGT87A subfamily in *Sapindus mukorossi* were recently identified in a BGC and displayed activity toward oleanolic acid, their specific regioselectivity remained elusive^27^. Here, we report for the first time that AcUGT87AZ3 catalyzes the addition of the final 1,4-D-glucose moiety to the glucuronic acid linked at the 3β-*O* position of the barrigenol-type triterpenoid. This identifies AcUGT87AZ3 as only the second functionally characterized member of the UGT87 family in secondary metabolism, following UGT87Y1, which catalyzes the glucosylation of mandelonitrile to form prunasin^69^. These findings expand the known catalytic functions of the UGT87 family and even the J group to which it belongs. Furthermore, MD simulations and biochemical assays provided critical insights into the assembly order of the branched trisaccharide chain, 2’-*O*-glycosylation precedes 4’-*O*-glycosylation. AcUGT94AK1/3/6 exhibited higher substrate specificity, exclusively catalyzing the 2’-*O*-glucosylation of the monoglycoside. In contrast, AcUGT87AZ3 displayed broader flexibility, catalyzing 4’-*O*-glucosylation on both the monoglycoside and the diglycoside intermediates. Collectively, this study provides the first experimental evidence that AcUGT94AK1/3/6 and AcUGT87AZ3 are involved in forming the branched (1 → 2 and 1 → 4) trisaccharide chain of **1**, offering valuable insights into the complex glycosylation patterns of triterpenoids.

The pharmacological activities of **1** are closely related to the acyl groups at C-21β and C-22α^70^. Specifically, 21β-tigloylation is not only the characteristic feature of **1**, but also a common structural motif in 23 of the 98 aescins reviewed by Zhang et al. (2010)^8^. However, the enzyme responsible for the 21β-*O*-tigloylation of aescins remained unknown for decades. Here, we provide the first in vivo and in vitro evidence that BAHD13 and BAHD17 are isoenzymes involved in the 21β-*O*-tigloylation of **1**. Phylogenetic analysis shows that AcBAHD13 and AcBAHD17 clustered with AcBAHD6, a previously characterized acetyltransferase of **1**, within clade 3. This clade contains enzymes involved in the acylation of aliphatic alcohols, terpenes, anthocyanin glycosides, aliphatic amines, and alkaloids. For example, a BAHD from this clade catalyzes the reaction between 3β-tropanol and tigloyl-CoA to form 3β-tigloyloxytropane in *Atropa belladonna*. These findings suggest that clade 3 of BAHDs plays vital roles in the tigloylation of secondary metabolites. Finally, we successfully reconstituted the biosynthesis of escin Ia (**1**) in *N. benthamiana* by co-infiltrating the functionally characterized genes identified in this study and previously^26^.

The production of **1** (122.55 μg/g DW) achieved in this study is competitive compared to the heterologous synthesis of other triterpenoids in *N. benthamiana*, such as QS-7 (7.9 mg/g DW)^20^, QS-21 (8.6 μg/g DW)^43^, and asiaticoside (97.4 μg/g DW)^71^,. However, metabolic flux analysis reveals a significant bottleneck at the terminal acylation steps. Indeed, the levels of the aglycone and various glycosylated intermediates, including **2,** **4**, and **12**, consistently reached mg/g scales. Particularly, intermediate **4** accumulated to a maximum of 6,123.06 μg/g. In comparision, the yield of the final product (**1**) remained relatively low. Overcoming this disparity will require multi-faceted metabolic engineering strategies. At the enzymatic level, this includes increasing the supply of acetyl-CoA and tigloyl-CoA donors, enhancing the catalytic efficiency of BAHDs through rational design or directed evolution. At the system level, pathway optimization, flux rewiring, transcriptional regulation, and spatial organization hold great promise for further elevating the production of compound 1, as evidenced by several successful precedents. For instance, systematic optimization of the *N. benthamiana* expression system enhanced rotenoid content by 5.0-38.6-fold, reaching 0.2–0.6 mg/g DW^72^. Similarly, “rewiring” diterpene biosynthesis from the plastidial MEP pathway to the high-flux cytosolic MVA pathway increased the content of forskolin and related compounds approximately 10-fold^73^. Furthermore, through the systematic reconstruction of the upstream isoflavone pathway, coupled with the utilization of transcription factors to upregulate target genes while suppressing competing branches, researchers achieved yields of 7.04 g/kg DW for daidzein and 11.85 g/kg DW for genistein^74^. Regarding spatial organization, MCD1 (miR1919-targeted cell death-factor-1) from Solanaceae plants was recently shown to act as a metabolic organizer, assembling three sequential enzymes into a complex to promote the biosynthesis of debneyol^75^. Combining these sophisticated strategies in our established *N. benthamiana* platform will pave the way for the scalable production of **1**.

Beyond their well-characterized pharmacological activities, triterpenoids serve as indispensable defensive metabolites evolved by plants to counteract pathogens, herbivores, and phytophagous insects^76,77^. The biosynthesis of **1** represents a substantial metabolic investment, requiring significant carbon flux through at least 15 specialized catalytic steps. This metabolic commitment underscores the vital contribution of **1** to the ecological fitness of *Aesculus* species. For instance, aescins exhibit potent antifungal activity against *Leptosphaeria maculans*, *Microdochium nivale* and five other important fungal pathogens via a synergistic dual-mode mechanism: directly disrupting fungal membrane sterols and priming the plant immune system via salicylic acid signaling and oxidative bursts^78^. Similarly, the localized accumulation of aescin in *A. pavia* leaves provides a robust barrier against leafminer infestation^79^. Furthermore, the distinct bitterness of **1** in seeds functions as a formidable deterrent to both generalist herbivores and insect pests, illustrating a specialized chemical strategy to safeguard reproductive tissues^76,80^. Taken together, we propose that **1** and its metabolic intermediates orchestrate a multi-layered chemical defense system, conferring comprehensive protection upon *Aesculus* species against a diverse array of biotic stressors.

Collectively, this study presents a comprehensive elucidation of the biosynthetic pathway for **1**, achieved through an integrated strategy of genomic mining, phylogenetics, in vitro biochemistry, and heterologous reconstitution in *N. benthamiana*. By identifying and functionally characterizing ten previously uncharacterized enzymes—including three P450s (AcCYP716BX1/2, and AcCYP714E67), four UGTs (AcUGT94AK1/3/6 and AcUGT87AZ3), a specialized CoA ligase (AcCCL3), and two BAHDs (AcBAHD13/17)—this work fills the remaining gaps in the conversion of compound **3** to **1**. Furthermore, these ten enzymes not only provide key insights into the polyhydroxylation, branched trisaccharide chain formation, and tigloylation of triterpenoids, but also broaden our understanding of the functional diversity within their respective gene families. The establishment of a *N. benthamiana* production platform validates the functional assignment of pathway components under near-native cellular conditions, paving the way for future scalable biosynthesis and downstream diversification of clinically relevant saponins. Building upon these findings, future research will focus on enhancing the biosynthetic efficiency of **1** through metabolic engineering, establishing a scaling production platform by transferring this pathway into microbial chassis such as *S. cerevisiae*, and utilizing this newfound enzymatic repertoire to create next-generation analogs of **1** via combinatorial biosynthesis.

## Methods

### Plant materials and chemicals

This study utilized *A. chinensis* tree cultivated at the Institute of Medicinal Plant Development (IMPLAD), Chinese Academy of Medical Sciences. Reference standards of escin Ia (**1**), protoaescigenin (**2**), and β-amyrin (**3**) were procured from Chenguang Biotechnology Inc. (Xi’an, China). Tiglic acid (**5**) was sourced from Yuanye Biotechnology Co., Ltd. (Shanghai, China). Enzymatic cofactors, including UDP-glucose (UDPG) and acetyl-CoA trisodium salt, were acquired from Sigma-Aldrich (St. Louis, MO, USA), while tigloyl-CoA (**6**) was purchased from Shanghai Kaisen Biotechnology Co., Ltd. (Shanghai, China). HPLC-grade reagents, such as methanol, acetonitrile, and formic acid, were obtained from Thermo Fisher Scientific (Waltham, MA, USA). The compounds aesculuside B (**4**), 21β-hydroxy-β-amyrin (**9**), barringtogenol C (**11**), 3β-*O*-{β-D-glucuronopyranosyl acid}-protoaescigenin (**12**), aeswilsaponin IC (**13**), 3β-*O*-{[β-D-glucopyranosyl-(1 → 4)]-β-D-glucuronopyranosyl acid}-protoaescigenin (**14**) and desacetylescin Ia (**15**) were previously isolated and purified by our collaborators, as detailed in our prior work^30,31^.

### Genome mining, phylogenetic analysis, gene expression analysis and identification of gene duplications

The genome assembly, RNA-sequencing and WGCNA analysis of *A. chinensis* were previously reported^26^. To better screen candidate genes, we further updated the annotation of the *A. chinensis* genome by integrating both short-read and long-read transcriptomic data. Specifically, short-read RNA-seq data were aligned, assembled, and merged using HISAT2 (v2.1.0)^81^ and StringTie (v2.0), while full-length transcriptome data were aligned with the isoseq3 (v3.4.0) package^82^. Subsequently, the structural annotation files were refined and merged using gffcompare (v0.12.6)^83^. Based on this newly updated genome annotation, the full-length protein sequences of target genes were identified using EggNOG and Pfam annotations (P450: PF00067; UDP-glucuronosyl/glucosyltransferase: PF00201; Carboxyl-CoA Ligase: PF00501; BAHD: PF02458)^84^. Multiple sequence alignment of amino acid sequences was conducted using MAFFT (version 7.526) with default parameters. Poorly aligned regions were trimmed using trimAI (version 1.5.0), retaining only the evolutionarily conserved regions for subsequent analysis. Phylogenetic trees were constructed with IQ-TREE (version 2.3.6) using the maximum likelihood method, with the ultrafast bootstrap approximation set to 1000 replicates. The best-fit amino acid substitution models were automatically selected by ModelFinder based on the Bayesian Information Criterion (BIC), resulting in the use of Q.plant+R7 for UGTs, LG + R6 for CCLs, and Q.plant+R5 for BAHDs. The resulting trees were visualized and annotated using ITOL v7.

To identify candidate genes implicated in escin Ia (**1**) biosynthesis, co-expression analysis was performed using R software (version 4.3.1) to calculate pairwise Pearson correlation coefficients. Specifically, correlations were evaluated between the FPKM values of candidate genes within the ‘turquoise’ module and both the accumulation of escin Ia (1) and the FPKM values of AcOSC6. Gene sequences were validated by aligning them to third-generation transcriptome data using TBtools-II v2.331^85^. Gene duplication modes were determined through the DupGen_finder pipeline^86^ (https://github.com/qiao-xin/DupGen_finder). Gene expression heatmaps were generated using TBtools-II v2.331^85^.

### Gene cloning and vector construction

Total RNA was isolated from nine-month-old *A. chinensis* seeds utilizing the EasyPure® Plant RNA Kit (TransGen, Beijing, China). First-strand complementary DNA (cDNA) was synthesized from purified RNA using the TransScript® One-Step gDNA Removal and cDNA Synthesis SuperMix kit (TransGen, Beijing, China), following the manufacturer’s instructions. The full-length open reading frames (ORFs) of candidate genes—P450s, UGTs, CCLs, and BAHD acyltransferases—were amplified via PCR with KOD-Plus-Neo DNA polymerase (TOYOBO Bio, Japan). All target genes were cloned into pEAQ-HT, pESC-HIS, or pET-series vectors (pET-32a/28a) using In-Fusion cloning. Recombinant pEAQ-HT constructs were introduced into *Agrobacterium tumefaciens* strain GV3101 for heterologous expression in *N. benthamiana*. The pESC-HIS constructs were introduced into *Saccharomyces cerevisiae* WAT11 strain, while pET-series plasmids were transformed into *Escherichia coli* BL21(DE3) for recombinant protein expression and in vitro enzymatic assays. Detailed primer sequences and vector information are provided in Supplementary Tables 6–9.

### Heterologous expression of candidate genes in *N. benthamiana* via agroinfiltration

For transient expression, the transformed *A. tumefaciens* strains were cultured on LB agar plates containing 50 µg/mL kanamycin and 50 µg/mL gentamicin at 28 °C for 2-3 days. Single colonies were selected, sequenced, and re-streaked on a fresh plate for 2 days. Cells were subsequently harvested and resuspended in 1 mL of MMA buffer (10 mM MES pH 5.6, 10 mM MgCl_2_, and 100 μM acetosyringone). Different *A. tumefaciens* strains harboring the target genes were combined based on the desired catalytic outcome, with the OD_600_ of each strain adjusted to 0.2 using MMA buffer. The pooled *A. tumefaciens* strains were incubated at room temperature in the dark for 3 h. Each combinatorial infiltration included the HMGR gene (tHMGR) from *Avena strigosa* to enhance triterpene production^87^. For the biosynthesis of tigloyl-CoA (**6**), tobacco leaves were infiltrated with tiglic acid (**5**) at a concentration of 1 mM two days after initial infiltration. For small-scale testing, manual agroinfiltration was conducted using a 1 mL needleless syringe on three to five *N. benthamiana* leaves per construct. For large-scale agroinfiltration, 100-200 *N. benthamiana* plants were treated using vacuum infiltration techniques. Leaves were harvested after one day in the darkness followed by four days in the light, then freeze-dried, ground into a powder, and immediately extracted.

Lyophilized leaf powder (20-30 mg dry weight, DW) was extracted with 1 mL of 80% methanol in an ultrasonic bath at room temperature for 30 min. The mixtures were then shaken at 28 °C and 200 rpm for 2 h. The extracts were centrifuged at 12000 g for 10 min. A volume of 700 µL of supernatant was collected and washed twice with 700 µL of pre-cooled *n*-hexane. The resulting mixture was evaporated to dryness using an Eppendorf Concentrator plus, and the residue was reconstituted in 200 μL of methanol for LC-MS analysis.

### Expression of candidate P450s in *S. cerevisiae* and in vitro enzymatic activity assay

Positive yeast colonies were selected and cultivated in synthetic complete (SC) medium lacking histidine and containing 2% (w/v) glucose. Yeast with candidate genes was grown in 500 mL of YPDA medium containing 1% glucose and 2% galactose until the glucose was consumed completely. After 16 h, the yeast cells were then lysed with an ultra-high pressure cell crusher, and the resulting supernatant was collected by sequential centrifugation at 10,000× g for 10 min at 4 °C, followed by centrifugation at 120,000× g for 90 min at 4 °C. The pellet containing microsomes was resuspended in 1.5 mL of buffer. The enzymatic assays were performed at 30 °C for 2 h in a reaction system containing 200 μL of microsomes, 2 μL NADPH (50 mM), and 2 μL substrate (1 mg/mL). The reaction was quenched by adding 200 μL of methanol, and the resulting supernatant was dried at 45 °C, resuspended in 200 μL of methanol to be analyzed by LC-MS.

### Expression of candidate genes in *E. coli*

Single colonies were cultured in LB medium supplemented with 50 μg/mL ampicillin for pET-32a(+) vectors or 50 μg/mL kanamycin for pET-28a (+) vectors at 37 °C until reaching an OD₆₀₀ of 0.6. Protein expression was then induced with 0.3 mM IPTG (final concentration) at 16 °C for 20 h. For protein purification, bacterial cells were harvested by centrifugation (6,000× g, 5 min, 4 °C) and resuspended in 3 mL of lysis buffer (50 mM Tris-HCl, pH 7.5). Cells were disrupted by ultrasonication on ice for 10 min, followed by centrifugation at 12,000× g for 30 min. The supernatant was purified using a nickel affinity column (BeyoGold™ His-tag Purification Resin, Beyotime) and concentrated using an Amicon Ultra-15 centrifugal filter unit (30 kDa molecular weight cutoff, Merck Millipore).

### In vitro enzyme assays of candidate AcUGTs, AcCCLs and AcBAHDs

The AcUGT catalytic reactions were performed in a mixture containing 1 μL UDP-Glu (100 mM), 2 μL substrate (1 mg/mL), 50 μL purified protein, and 1×PBS to yield a total volume of 200 μL. The AcUGT reactions were terminated with an equal volume of methanol after incubation at 30 °C for 2 h. The AcCCL reactions were carried out in 1 mL systems containing 50 mM Tris-HCl, 5 mM MgCl₂, 2.5 mM ATP, 0.2 mM CoA, 1 mM tiglic acid, and 10 μg of purified AcCCL protein. These reactions were terminated by boiling for 5 min after incubation at 25 °C for 16 h. For the sequential catalytic reaction of AcCCL3 and AcBAHDs, the reactions catalyzed by AcCCL3 were first performed at 25 °C for 4 h as described above. Subsequently, 10 μg of AcBAHD13/17 protein and 0.2 mM aesculuside B were supplemented; if AcBAHD6 was also included, 0.2 mM acetyl-CoA was added, followed by continued incubation for a total of 16 h. The reaction was quenched by adding an equal volume of ethyl acetate and sonicating for 30 min. The organic phase was evaporated to dryness and reconstituted in 200 μL of methanol. All the in vitro reaction mixtures were analyzed by LC-MS to determine whether any products were generated.

### Qualification and quantification analysis of products by LC-MS

The liquid chromatography-mass spectrometry (LC-MS) analysis for catalytic product identification was performed utilizing an LC-QTOF mass spectrometer (1290-6546, Agilent Technologies Co., Ltd., USA). For AcP450-derived products, analyses were conducted via an atmospheric pressure chemical ionization (APCI) source in positive ion mode (*m/z* 50–500), whereas a dual Agilent Jet Stream electrospray ionization (Dual AJS ESI) source in negative ion mode (*m/z* 50–500) was employed for detecting products of AcUGTs, AcBAHDs and AcCCLs. The APCI ionization parameters were configured as follows: gas temperature at 350 °C, drying gas flow rate of 10 L/min, nebulizer pressure of 35 psi, VCap voltage of 3500 V, fragmentor voltage of 150 V, skimmer voltage of 65 V, and octupole 1 RF Vpp of 750 V. The Dual AJS ESI source was optimized under identical conditions, with the exception of the ionization mode. Operational parameters, including flow rate (0.3 mL/min), injection volume (3 μL), and column temperature (40 °C), were maintained consistently across analyses. DTIM-MS was carried out on 6560 ion mobility LC-QTOF (Agilent Technologies Co., Ltd., USA). For the DTIM-MS conditions, the trap fill time was 10000 μs, the trap release time was set to 150 μs, and the drift tube entrance voltage was 1200 V.

Quantitative assessment of products from transiently expressed candidate genes in *N. benthamiana* leaves was performed on an LC-QQQ mass spectrometer (1290-6470, Agilent Technologies Co., Ltd., USA) equipped with an APCI source for **2**, and a Dual AJS ESI source for **12,** **4**, and **1**. The quantitation analysis was conducted by external calibration of the corresponding standards, and the Multiple Reaction Monitoring (MRM) parameters, the linear ranges, calibration curves, and correlation coefficients (R^2^) for **2,** **12,** **4**, and **1** are listed in Supplementary Tables 10–11. The flow rate, injection volume, and column temperature were identical to those employed in the LC-QTOF analyses as detailed above. Chromatographic column specifications, mobile phase compositions, and gradient elution profiles are provided in Supplementary Table 12. The ppm of key compounds was listed in Supplementary Table 13. Data processing was performed using Agilent MassHunter Qualitative Analysis 10.0 software.

### Metabolite purification of transgenic *N. benthamiana* leaves

Large-scale leaf samples from 50 to 100 *N. benthamiana* plants were used for metabolite purification, which were lyophilized to complete dryness and ground to a powder by a knife mill. The leaf powder was then placed in a 4 L Erlenmeyer flask to remove lipids by adding *n*-hexane (at a solid-to-liquid ratio of 1:10, w/v), and shaking at 37 °C overnight. The mixture was allowed to stand to precipitate, and the supernatant was discarded. This defatting step was repeated by shaking with *n*-hexane (at a solid-to-liquid ratio of 1:5, w/v) at 37 °C for 2 h. After discarding the supernatant, the remaining residue was extracted three times with ethyl acetate (solid-to-liquid ratio of 1:10, w/v) by heated reflux for 2 h. The three batches of extracts were combined and filtered using vacuum filtration, and concentrated to dryness using a rotary evaporator. The ethyl acetate extract was then adsorbed onto silica gel in a ratio of 1:2 (extract:silica gel, w/w) and dried under reduced pressure, before loading onto a column for separation through flash column chromatography.

Metabolite purification from transgenic *N. benthamiana* leaves was performed using a flash chromatography system (SepaBean^TM^ machine T, Santai Technologies, Changzhou, China) equipped with a silica column (S-8101-0025, 40–63 μm, 60 Å, 25 g, Santai Technologies, Changzhou, China). The mobile phases consisted of *n*-hexane (A) and ethyl acetate (B) with a constant flow rate of 35 mL/min. A gradient of solvent B was applied as follows: 0–6% for 4 column volumes (CV), 6–20% for 12 CV, 20–30% for 20 CV, 30–40% for 30 CV, 40–50% for 35 CV, 50–60% for 40 CV, 60–100% for 41 CV, and 100% for 60 CV. Fractions (17 mL per tube) containing the target compounds, as verified by LC-QTOF-MS, were combined and concentrated using a rotary evaporator.

The fractions containing the target products were further separated using an Agilent 1100 HPLC system (Agilent Technologies Co., Ltd., Santa Clara, CA, USA) equipped with a COSMOSIL 5C_18_ -MS-Ⅱ column (10 mm I.D.×250 mm). The mobile phases consisted of water (A) and methanol (B) at a flow rate of 2.5 mL/min. The elution gradients of B were as follows: 0-60 min, 75%; 61–70 min, 100%; 71–90 min, 75%. The injection volume and column temperature were set at 100 µL and 40 °C, respectively. The elution fractions containing the target compounds were combined, dried to completion using a rotary evaporator, and lyophilized for NMR analysis.

### NMR analysis of purified compounds

The purified compounds were all dissolved in pyridine-*d*_5_. NMR spectra were conducted on Bruker 400 or 600 MHz NMR spectrometers at a temperature of 298 K. All NMR data were evaluated employing Bruker TopSpin 4.4.0 software, using deuterated solvents as external standards, and are presented as δ in ppm and J in Hz. The multiplicity of signals was indicated as s (singlet), d (doublet), t (triplet), q (quartet), dd (double doublet), and m (multiplet), among others. Signal assignments were determined through an extensive analysis of ^1^H, ^13^C, DEPT-135, DEPT-edited HSQC, HMBC, and 2D NOESY or ROESY experiments.

### Molecular docking and molecular dynamics simulations

The three-dimensional structures of AcUGT94AK1, AcUGT94AK3, AcUGT94AK6, and AcUGT87AZ3 were predicted using AlphaFold3^88^. The top-ranked models were selected based on their average pLDDT scores. Molecular docking was performed using AutoDock4.2.6^89^. A cubic grid box with dimensions of 100 × 70 × 60 Å was defined to encompass the N-terminal substrate-binding pocket. The grid center was positioned to cover the key catalytic residues with the x, y, and z coordinates of (3.437, 6.962, 3.030). Docking calculations were carried out using the Lamarckian Genetic Algorithm with 200 independent runs. The most favorable binding poses were selected based on a combination of binding free energy and the orientation of the ligand relative to key catalytic residues, and these poses were then used as starting structures for molecular dynamics simulations.

The complexes were subjected to molecular dynamics simulations using the AMBER22^90^ software package. The ff14SB^89^ force field was applied to proteins, while ligand parameters were described using the GAFF2^91^ force field. Each system was solvated in an explicit TIP3P^92^ water box with a buffer distance of at least 10 Å, and counterions were added to neutralize the system. Energy minimization was performed for 5000 steps to relieve any steric clashes. This was followed by a short NVT heating simulation for 0.1 ns using a 1 fs time step, and then a 1 ns NPT equilibration with a 2 fs time step. Production MD simulations were conducted with a 2 fs time step for 200 ns. Trajectory analyses were performed using the CPPTRAJ module in AMBER. The binding free energies were estimated using the MM/GBSA method based on snapshots extracted from the trajectories.

### Image credits

All schematic figures in this study were originally designed and created by the authors. To enhance visual clarity and representation, four specific vector icons were adapted from public online repositories under their respective licensing terms: the syringe and injectable solution icons by Servier Smart Medical Art (https://smart.servier.com/), licensed under CC-BY 3.0 Unported (https://creativecommons.org/licenses/by/3.0/); the centrifuge tube icon by Helicase 11 (CC-BY 4.0, https://creativecommons.org/licenses/by/4.0/); and the syringe schematic by Luz Eugenia Velasquez via Vecteezy (Vector Art ID: 14175787, Vecteezy Free License).

### Reporting summary

Further information on research design is available in the Nature Portfolio Reporting Summary linked to this article.

## Supplementary information

Supplementary Information

Reporting Summary

Transparent Peer Review file

## Source data

Source Data

## Data Availability

The nucleotide sequences of the functionally characterized genes in this study have been deposited in the GenBase database at Beijing Institute of Genomics, Chinese Academy of Sciences (https://ngdc.cncb.ac.cn/genbase), under the following accession codes: *AcCYP716BX1* (C_AA118697.1), *AcCYP716BX2* (C_AA118699.1), *AcCYP714E67* (C_AA118693.1), *AcUGT94AK1* (C_AA118701.1), *AcUGT94AK3* (C_AA118702.1), *AcUGT94AK6* (C_AA118700.1), *AcUGT87AZ3* (C_AA118703.1), *AcCCL3* (C_AA118698.1), *AcBAHD13* (C_AA118694.1), and *AcBAHD17* (C_AA118695.1). The updated gene annotation of *A. chinensis* has been deposited in the Figshare database (https://doi.org/10.6084/m9.figshare.21350865.v2). The raw analytical data, including all LC-MS datasets and NMR spectra, have been deposited in the Figshare database (https://doi.org/10.6084/m9.figshare.32613411). Both datasets are publicly and fully accessible. Source data are provided with this paper.
