# Supplementary material for: Complete biosynthesis of the clinical agent escin Ia
Source: Nat Commun. 2026 Aug 18;17:9891. doi: 10.1038/s41467-026-76851-3 (PMC13582942; doi:10.1038/s41467-026-76851-3)
Supplement: Supplementary file 1 [file 41467_2026_76851_MOESM1_ESM.pdf]

Complete Biosynthesis of the Clinical Agent Escin Ia

Huihua Wan[1#], *Mofan Zhang*[1#], *Ranran Gao*[1,2#], *Xuehua Xie*[1#], *Xiangxiao Meng*[1], *Songchun Guan*[1], *Weiqiang Chen*[1], *Xue Cao*[1], *Wei Yang*[1], *Hui Yao*[3], *Chen Qin*[1], *YinDi Zhu*[4], *Yi Zhang*[5], *Zhaohua Shi*[6,7], *Xiang Chen*[8], *Lichun Ye*[7,9], *Huiyuan Gao*[10], *Shilin Chen*[11*], *Zhichao Xu*[2*], *Wei Sun*[1*]

1 State Key Laboratory for Quality Ensurance and Sustainable Use of Dao-di Herbs, Key Laboratory of Beijing for Identification and Safety Evaluation of Chinese Medicine, Institute of Chinese Materia Medica, China Academy of Chinese Medical Sciences, Beijing 100700, China

2 Key Laboratory of National Forestry and Grassland Administration on Chinese Herbal Medicine, College of Life Science, Northeast Forestry University, Harbin 150040, China

3 State Key Laboratory of Bioactive Substance and Function of Natural Medicines, Institute of Medicinal Plant Development, Chinese Academy of Medical Sciences & Peking Union Medical College, Beijing 100193, China

4 School of Chinese Medicine, Wenzhou Medical University, Wenzhou 325035, China

5 Tianjin Key Laboratory of TCM Chemistry and Analysis, Tianjin University of Traditional Chinese Medicine, Tianjin 301617, China

6 Key Laboratory of Chinese Medicine Resource and Compound Prescription, Ministry of Education, Hubei University of Chinese-Medicine, Wuhan 430065, China

7 Hubei-Shizhen Laboratory, Wuhan, Hubei, 430065, China

8 School of Pharmaceutical Sciences, Sun Yat-sen University, Guangzhou 510006, China

9 College of Traditional Chinese Medicine, Hubei University of Chinese Medicine, Wuhan, Hubei, 430065, China

10 School of Traditional Chinese Materia Medica, Shenyang Pharmaceutical University, Shenyang 110016, China.

11 Institute of Herbgenomics, Chengdu University of Traditional Chinese Medicine, Chengdu 611137, China

# These authors contributed equally: *Huihua Wan, Mofan Zhang, Ranran Gao, Xuehua Xie*.

* Corresponding authors. E-mail address: wsun@icmm.ac.cn; slchen@icmm.ac.cn; zcxu@nefu.edu.cn

This Supplementary Information contains:

Supplementary Figures 1-59

Supplementary Tables 1-13

Supplementary Figures 1 to 59:

Supplementary Figure 1. The MS^1 and MS^2 spectra of 22α-hydroxy-erythrodiol (**7**). 5

Supplementary Figure 2. Overlay of LC-QTOF-MS/MS extracted ion chromatograms for 22α-hydroxy-oleanic acid (**8**, *m/z* 455.3525) produced in infiltrated *N. benthamiana*. 6

Supplementary Figure 3. The MS^1 and MS^2 spectra of 22α-hydroxy-oleanic acid (**8**). 7

Supplementary Figure 4. Key COSY and HMBC correlations of **7**–**8**. 8

Supplementary Figure 5. ^{1}H NMR spectrum of **7** in pyridine-d_5. 9

Supplementary Figure 6. ^{13}C APT spectrum of **7** in pyridine-d_5. 10

Supplementary Figure 7. HSQC spectrum of **7** in pyridine-d_5. 11

Supplementary Figure 8. HMBC spectrum of **7** in pyridine-d_5. 12

Supplementary Figure 9. COSY spectrum of **7** in pyridine-d_5. 13

Supplementary Figure 10. NOESY Spectrum of **7** in pyridine-d_5. 14

Supplementary Figure 11. ^{1}H NMR spectrum of **8** in pyridine-d_5. 15

Supplementary Figure 12. ^{13}C APT spectrum of **8** in pyridine-d_5. 16

Supplementary Figure 13. HSQC spectrum of **8** in pyridine-d_5. 17

Supplementary Figure 14. HMBC spectrum of **8** in pyridine-d_5. 18

Supplementary Figure 15. COSY spectrum of **8** in pyridine-d_5. 19

Supplementary Figure 16. ROESY spectrum of **8** in pyridine-d_5. 20

Supplementary Figure 17. The MS^1 and MS^2 spectra of 21β,22α-dihydroxy-erythrodiol (**10**). 21

Supplementary Figure 18. Key COSY and HMBC correlations of **10**. 22

Supplementary Figure 19. ^{1}H NMR spectrum of **10** in pyridine-d_5. 23

Supplementary Figure 20. ^{13}C NMR spectrum of **10** in pyridine-d_5. 24

Supplementary Figure 21. HSQC Spectrum of **10** in pyridine-d_5. 25

Supplementary Figure 22. HMBC Spectrum of **10** in pyridine-d_5. 26

Supplementary Figure 23. COSY Spectrum of **10** in pyridine-d_5. 27

Supplementary Figure 24. NOESY Spectrum of **10** in pyridine-d_5. 28

Supplementary Figure 25. The MS^1 and MS^2 spectra of barringtogenol C (**11**). 29

Supplementary Figure 26. Identification of *P450s* involved in the C24 hydroxylation of barringtogenol C (**11**) to generate protoaescigenin (**2**) in infiltrated *N. benthamiana*. 30

Supplementary Figure 27. The MS^1 and MS^2 spectra of protoaescigenin (**2**). 31

Supplementary Figure 28. Co-infiltration of **11** and *AcCYP714E67* into *N. benthamiana*. 32

Supplementary Figure 29. Co-infiltration of *AstHMGR*, *AcOSC6* and *AcCYP714E67* into *N. benthamiana*. 33

Supplementary Figure 30. Complex metabolic network for the biosynthesis of **2** from **3** 34

Supplementary Figure 31. Mass spectra (A) and proposed fragmentation pathways (B) of 21β,24-dihydroxy-β-amyrin (**16**) produced in infiltrated *N. benthamiana*. 35

Supplementary Figure 32. Mass spectra (A) and proposed fragmentation pathways (B) of 16α,21β-dihydroxy-β-amyrin

(**17**) produced in infiltrated *N. benthamiana*. 36

Supplementary Figure 33. Mass spectra (A) and proposed fragmentation pathways (B) of 21β,22α,24-trihydroxy-erythrodiol (**18**) produced in infiltrated *N. benthamiana*. 37

Supplementary Figure 34. Mass spectra (A) and proposed fragmentation pathways (B) of 16α,21β,24-trihydroxy-β-amyrin (**19**) produced in infiltrated *N. benthamiana*. 38

Supplementary Figure 35. Mass spectra (A) and proposed fragmentation pathways (B) of 16α,22α-dihydroxy-erythrodiol (**20**) produced in infiltrated *N. benthamiana*. 39

Supplementary Figure 36. Mass spectra (A) and proposed fragmentation pathways (B) of 22α,24-dihydroxy-erythrodiol (**21**) produced in infiltrated *N. benthamiana*. 40

Supplementary Figure 37. Mass spectra (A) and proposed fragmentation pathways (B) of 16α,22α,24-trihydroxy-erythrodiol (**22**) produced in infiltrated *N. benthamiana*. 41

Supplementary Figure 38. Elimination of interference from endogenous *N. benthamiana* P450s via co-expression minus-one control experiments. 42

Supplementary Figure 39. Functional validation of AcCYP716BX1 and AcCYP716BX2 via yeast microsomal assays. 43

Supplementary Figure 40. Functional validation of AcCYP714E67 via yeast microsomal assays. 44

Supplementary Figure 41. Protoaescigenin (**2**) content in infiltrated *N. benthamiana*. 45

Supplementary Figure 42. *In vitro* functional analysis of candidate AcUGTs using **12** as substrate detected by the 15 min elution gradient. 46

Supplementary Figure 43. Functional characterization of candidate AcUGTs using **12** as substrate detected by the 35 min elution gradient. 47

Supplementary Figure 44. The MS^1 and MS^2 spectra of 3β-*O*-{[β-D-glucopyranosyl-(1→2)]-β-D-glucuronopyranosyl acid}-protoaescigenin (Aeswilsaponin IC, **13**) generated *in vitro*. 48

Supplementary Figure 45. The MS^1 and MS^2 spectra of 3β-*O*-{[β-D-glucopyranosyl-(1→4)]-β-D-glucuronopyranosyl acid}-protoaescigenin (**14**). 49

Supplementary Figure 46. The MS^1 and MS^2 spectra of **13** generated in infiltrated *N. benthamiana*. 50

Supplementary Figure 47. *In vitro* functional analysis of candidate AcUGTs using **13** as substrate detected by the 15 min elution gradient. 51

Supplementary Figure 48. The MS^1 and MS^2 spectra of 3β-*O*-{β-D-glucopyranosyl-(1→4)-[β-D-glucopyranosyl-(1→2)]-β-D-glucuronopyranosyl acid}-protoaescigenin (Aesculuside B, **4**). 52

Supplementary Figure 49. *In vitro* functional analysis of candidate AcUGTs using **14** as substrate detected by the 15 min elution gradient. 53

Supplementary Figure 50. Phylogenetic analysis of the AcUGT gene family. 54

Supplementary Figure 51. Sequence alignment among AcUGT94AK1, AcUGT87AZ3 and UGT91H_A1. 55

Supplementary Figure 52. Root-mean-square deviation (RMSD) profiles of AcUGT94AK1 and AcUGT87AZ3 in the complexes with various ligands during 200 ns MD simulations. 56

Supplementary Figure 53. The measured O–N distance between the oxygen atom of the acceptor molecule and the ε-nitrogen of histidine. 57

Supplementary Figure 54. Phylogenetic analysis of the AcCCL gene family. 58

Supplementary Figure 55. The mass spectra of Desacetylescin Ia (**15**). 59

Supplementary Figure 56. The mass spectra of escin Ia (**1**). 60

Supplementary Figure 57. Phylogenetic analysis of the AcBAHD6, AcBAHD11, AcBAHD13, and AcBAHD17 with known BAHDs. 61

Supplementary Figure 58. Elucidation and reconstruction of escin Ia biosynthesis in *N. benthamiana.* 62

Supplementary Figure 59. Quantification of **12**, **4**, and **1** in infiltrated *N. benthamiana*. 63

Supplementary Tables 1 to 13

Supplementary Table 1. The expression patterns of candidate AcP450s, AcUGTs and AcCCLs, which were identified in the 'turquoise' module of WGCNA 64

Supplementary Table 2. Candidate P450s identified in biosynthetic genomic clusters (BGCs) and their expression patterns in different tissues. 66

Supplementary Table 3. ^{1}H (600 MHz) and ^{13}C NMR (150 MHz) data for **7** and **8** in pyridine-d_5. 67

Supplementary Table 4. ^{1}H (600 MHz) and ^{13}C NMR (150 MHz) data for **10** in pyridine-d_5. 69

Supplementary Table 5. Sequence similarity of four AcUGT enzymes 71

Supplementary Table 6. Designed primers for cloning *AcP450s* into the pEAQ-HT vector. 72

Supplementary Table 7. Designed primers for cloning *AcP450s* into the pESC-HIS vector. 73

Supplementary Table 8. Designed primers for cloning *AcUGTs*, *AcCCLs* and *AcBAHDs* into pET-series vectors. 74

Supplementary Table 9. Designed primers for cloning *AcCSL1*, *AcUGTs*, *AcCCL3* and *AcBAHDs* into pEAQ-HT vector. 76

Supplementary Table 10. Parameters of the Multiple Reaction Monitoring (MRM) for **2**, **12**, **4**, and **1**. 77

Supplementary Table 11. Linear ranges, calibration curves and correlation coefficients (R^2) for **2**, **12**, **4**, and **1** obtained by LC-QQQ-MS. 78

Supplementary Table 12. Specific parameters of LC-MS/MS analysis of enzymatic products. 79

Supplementary Table 13. The ppm of key compounds. 80

References 81

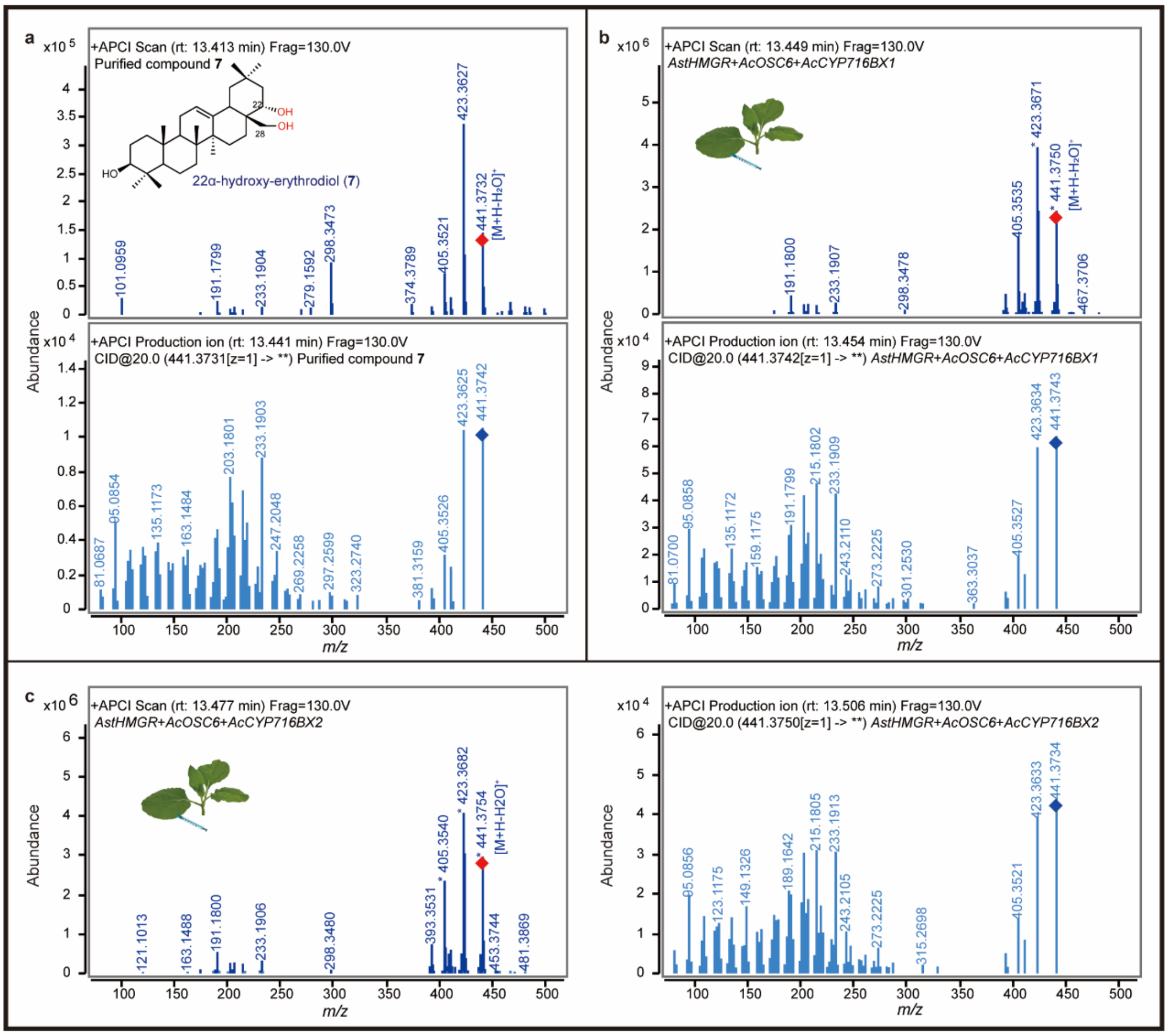

Supplementary Figure 1. The MS^1 and MS^2 spectra of 22α-hydroxy-erythrodiol (**7**). (**a**) The purified compound **7**. (**b**, **c**) The corresponding compound generated by *AstHMGR+AcOSC6+AcCYP716BX1* and *AstHMGR+AcOSC6+AcCYP716BX2* in infiltrated *Nicotiana benthamiana*. The syringe schematic was created by Luz Eugenia Velasquez and obtained from Vecteezy (Vector Art ID: 14175787, Vecteezy Free License, https://www.vecteezy.com/vector-art/14175787-syringe-medical-drug).

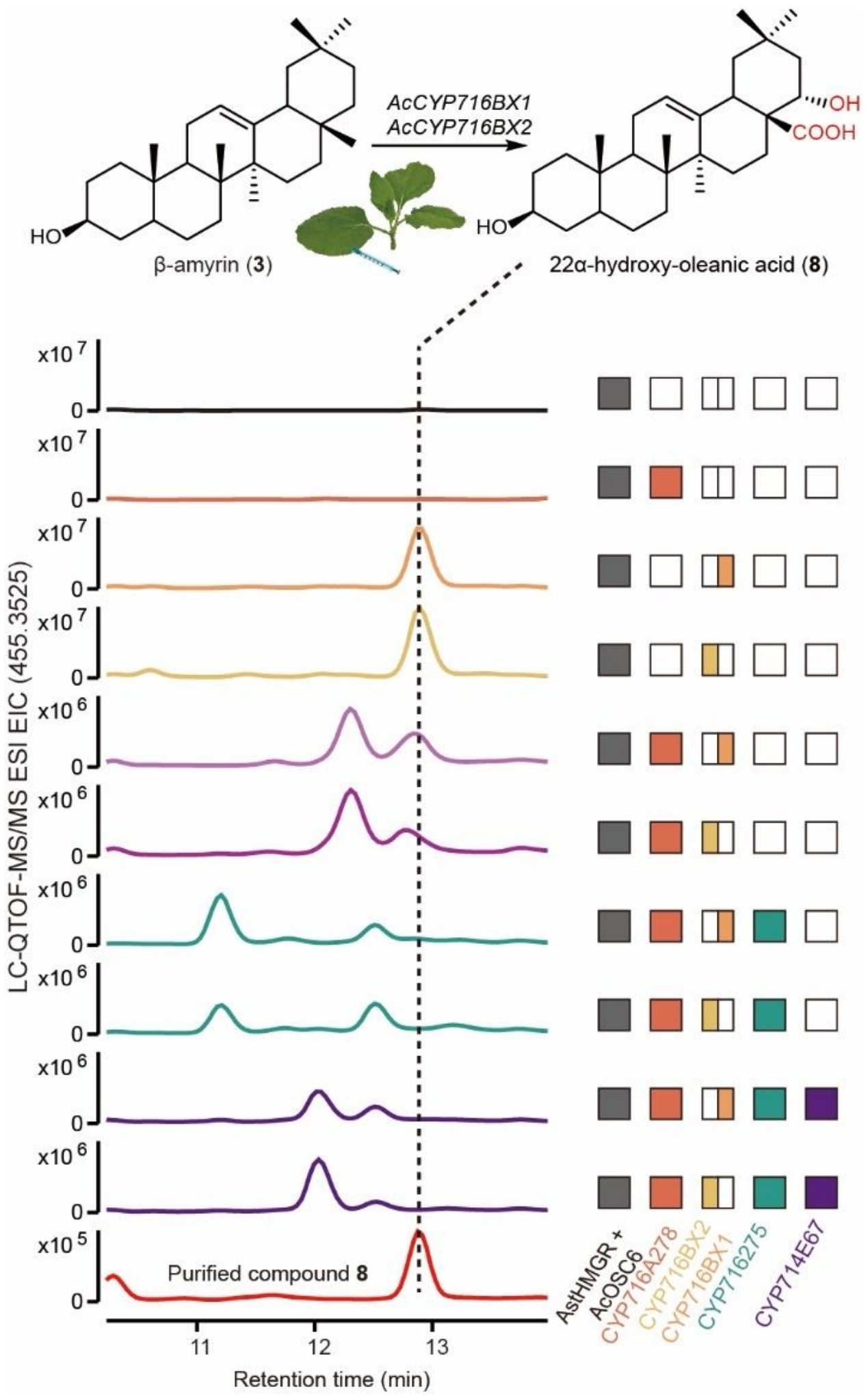

Supplementary Figure 2. Overlay of LC-QTOF-MS/MS extracted ion chromatograms for 22α-hydroxy-oleanic acid (**8**, *m/z* 455.3525) produced in infiltrated *N. benthamiana*. The syringe schematic was created by Luz Eugenia Velasquez and obtained from Vecteezy (Vector Art ID: 14175787, Vecteezy Free License, https://www.vecteezy.com/vector-art/14175787-syringe-medical-drug).

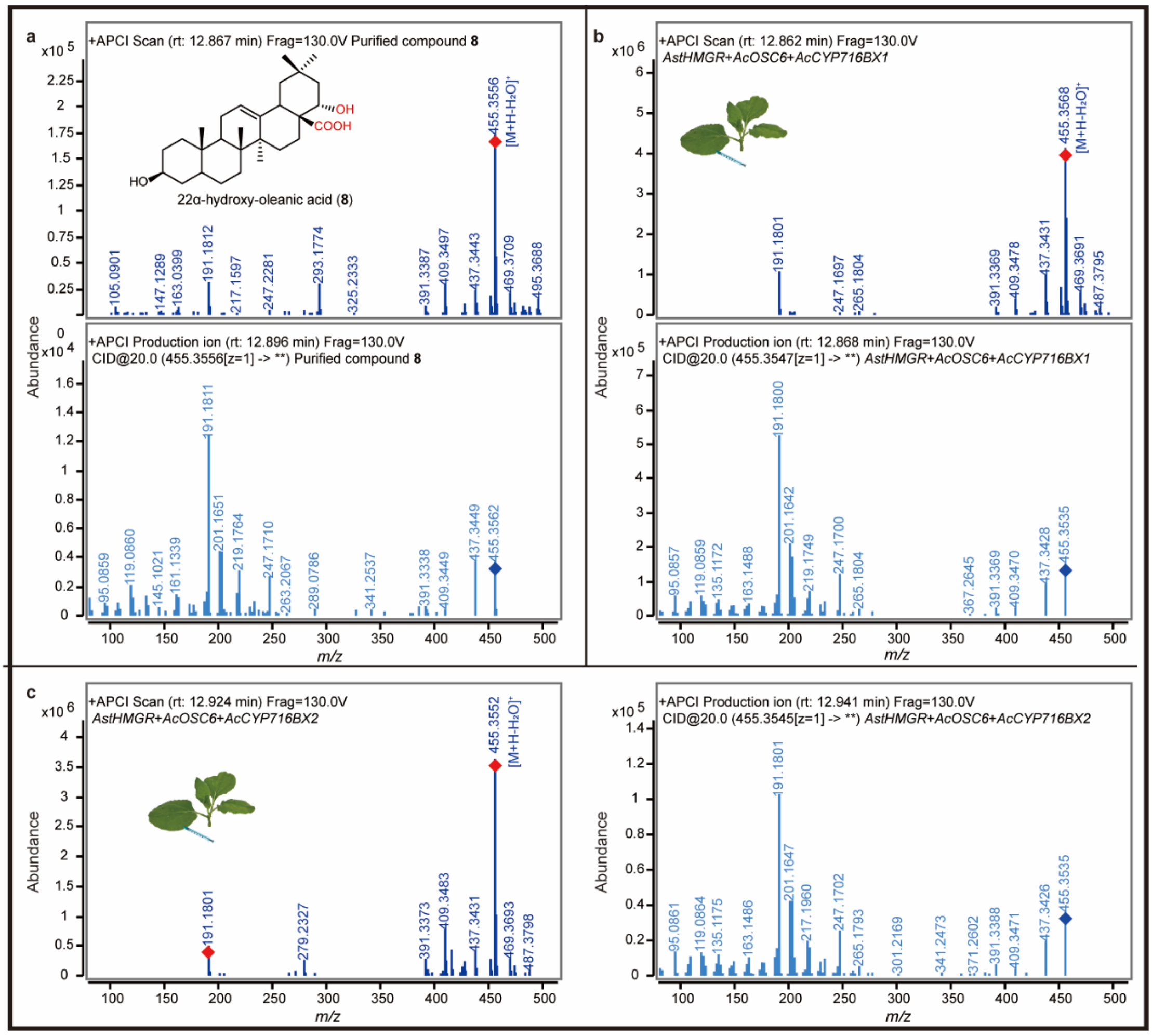

Supplementary Figure 3. The MS[1] and MS[2] spectra of 22α-hydroxy-oleanic acid (**8**). (**a**) The purified compound **8**. (**b**, **c**) The corresponding product generated by *AstHMGR+AcOSC6+AcCYP716BX1* and *AstHMGR+AcOSC6+AcCYP716BX2* in infiltrated *N. benthamiana*. The syringe schematic was created by Luz Eugenia Velasquez and obtained from Vecteezy (Vector Art ID: 14175787, Vecteezy Free License, https://www.vecteezy.com/vector-art/14175787-syringe-medical-drug).

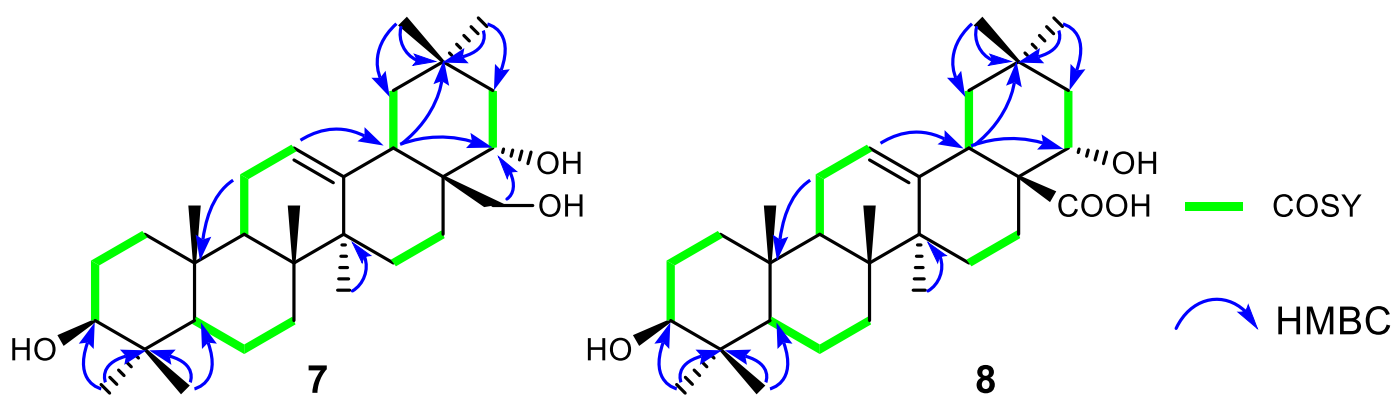

Supplementary Figure 4. Key COSY and HMBC correlations of **7–8**.

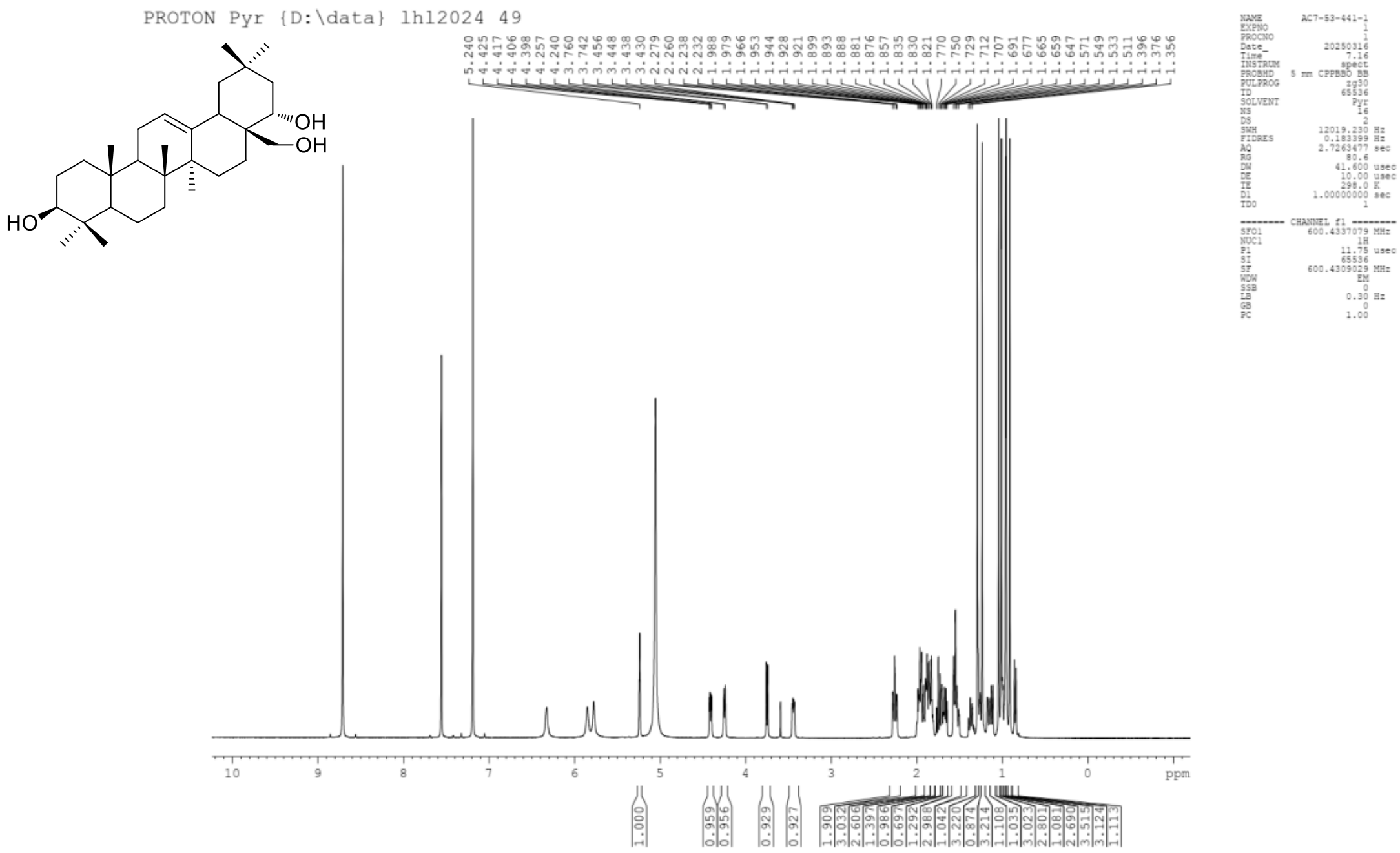

Supplementary Figure 5. ^{1}H NMR spectrum of **7** in pyridine-d_5.

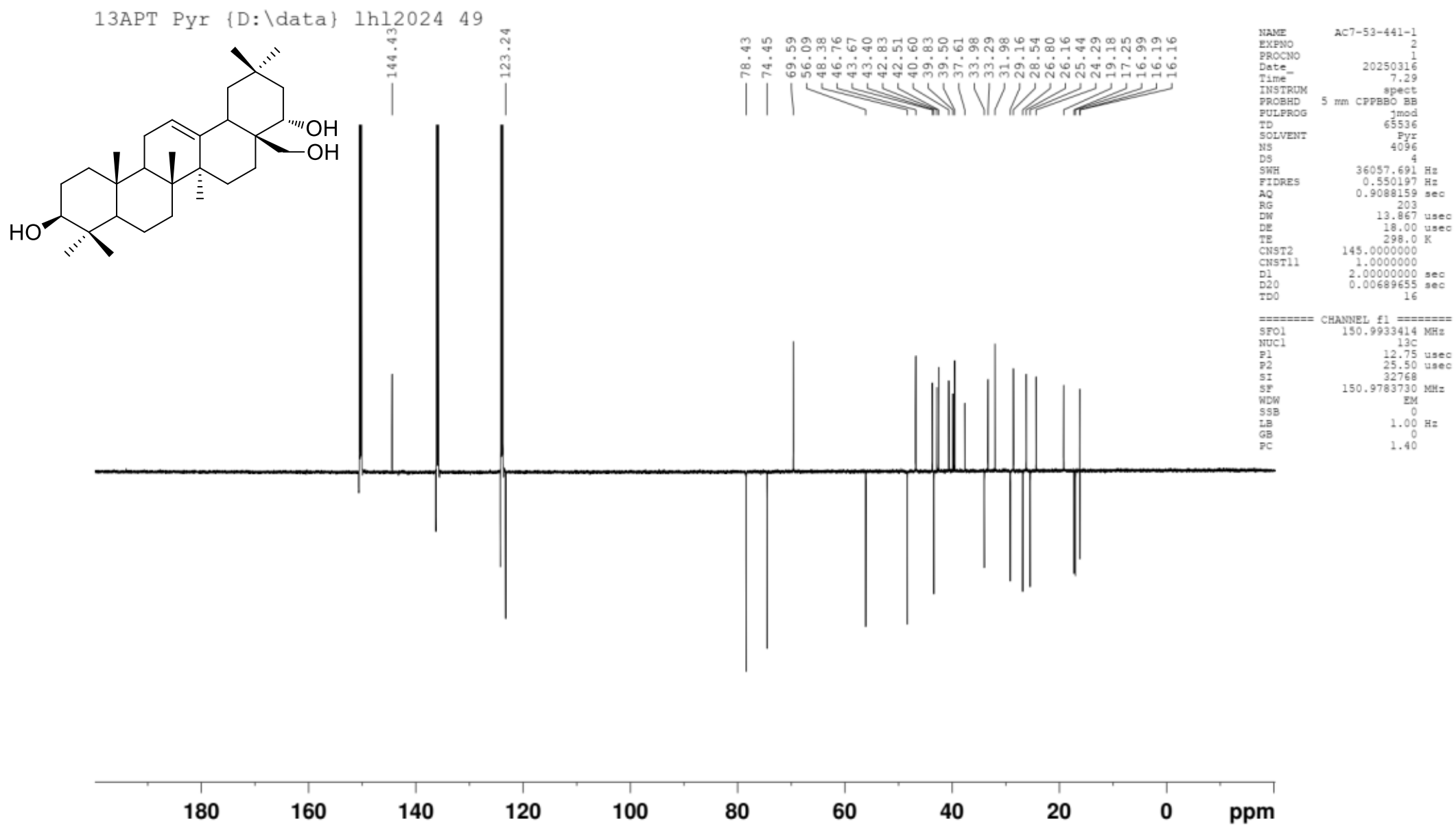

Supplementary Figure 6. ^{13}C APT spectrum of **7** in pyridine-d_5.

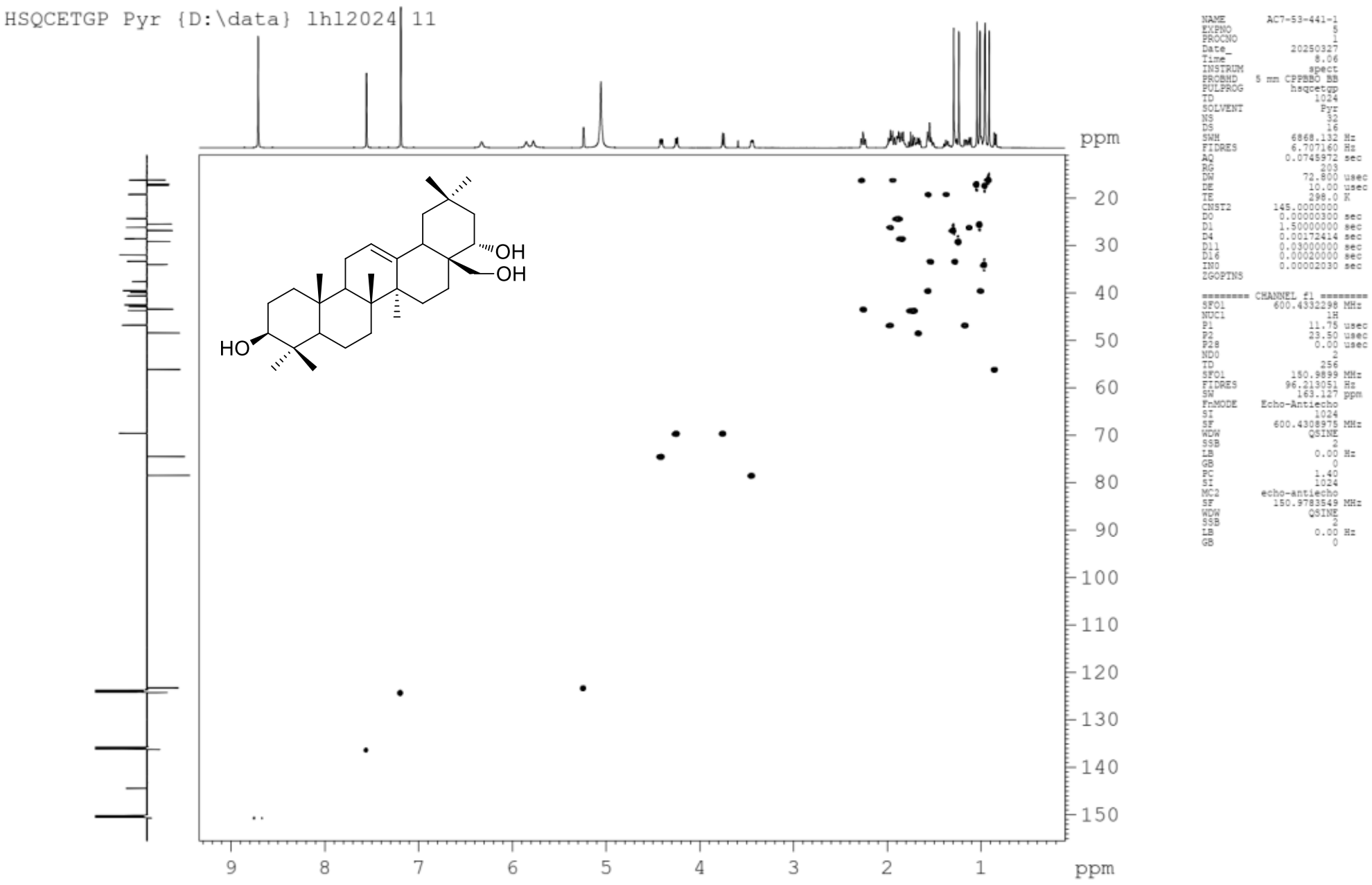

Supplementary Figure 7. HSQC spectrum of **7** in pyridine-d_5.

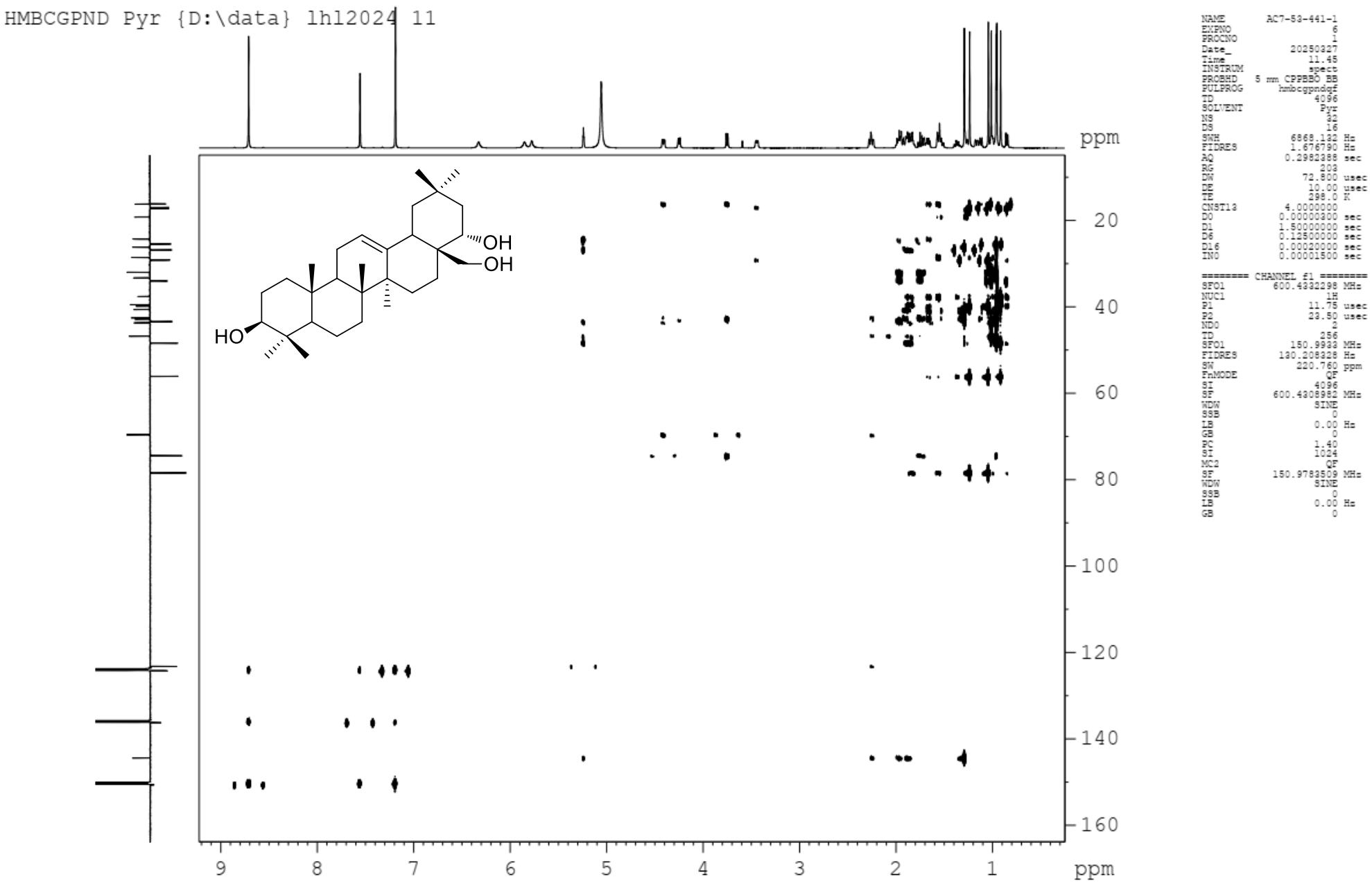

Supplementary Figure 8. HMBC spectrum of **7** in pyridine-d_5.

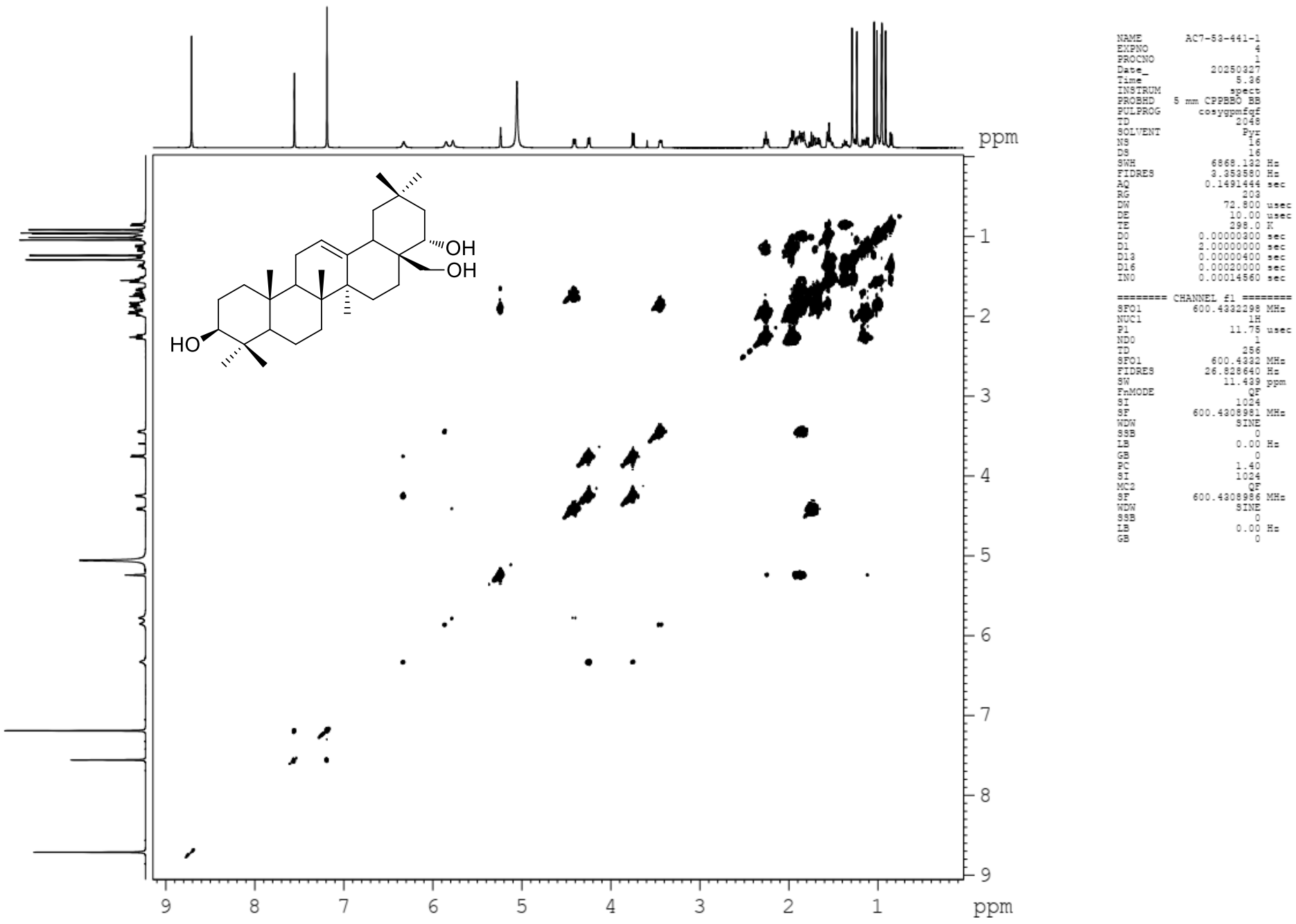

Supplementary Figure 9. COSY spectrum of **7** in pyridine-d_5.

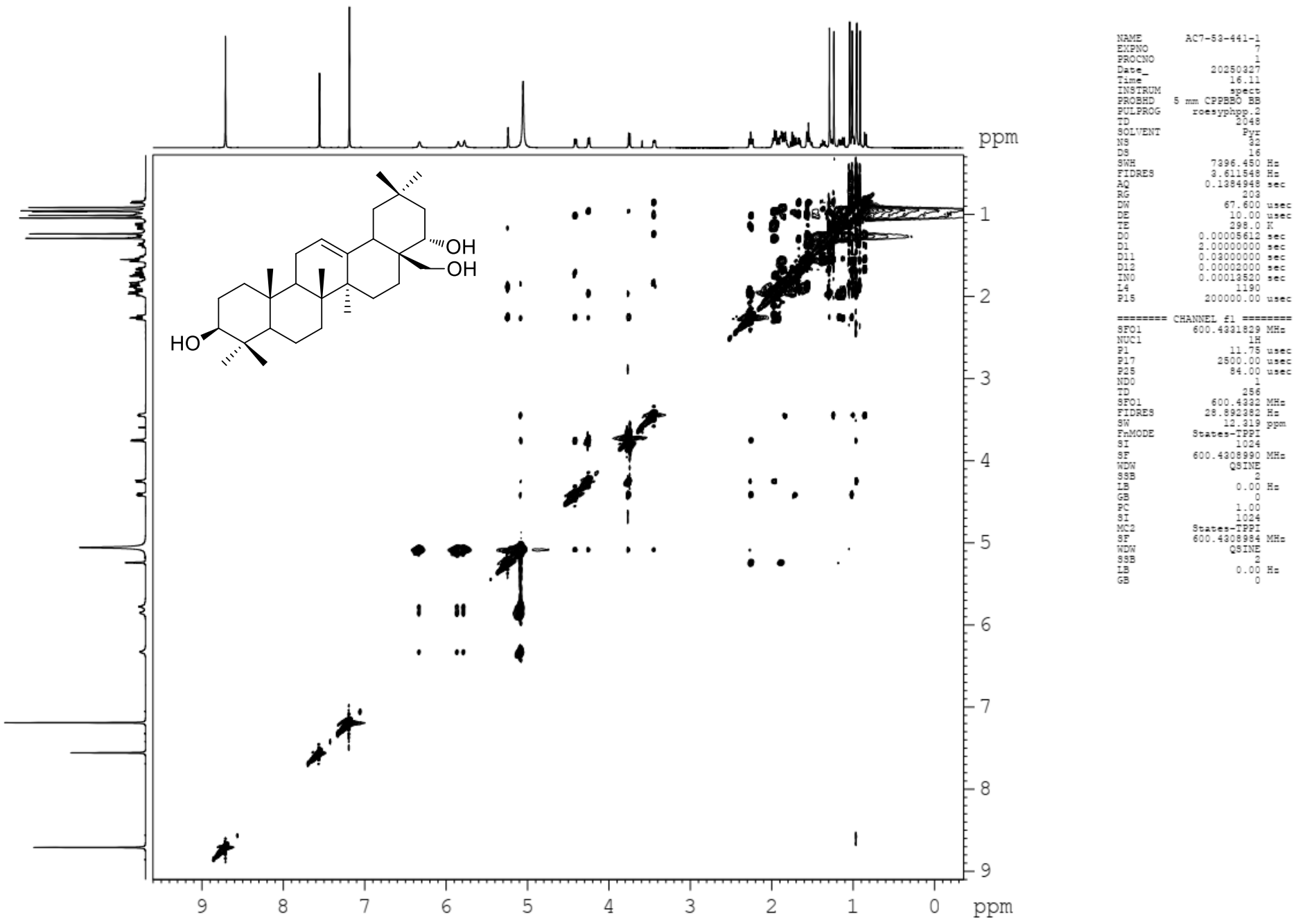

Supplementary Figure 10. NOESY Spectrum of **7** in pyridine-d_5.

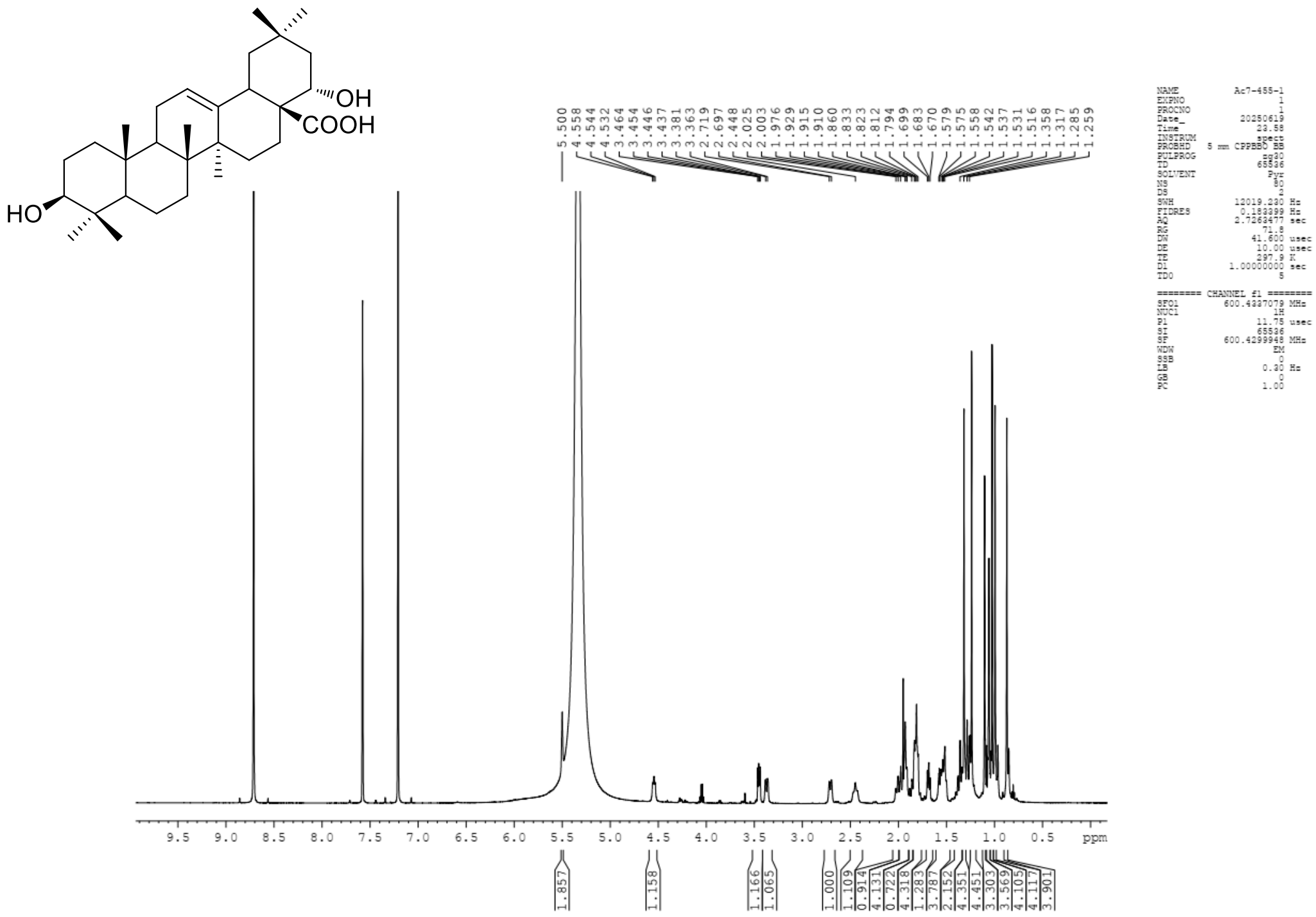

Supplementary Figure 11. ^{1}H NMR spectrum of **8** in pyridine-d_5.

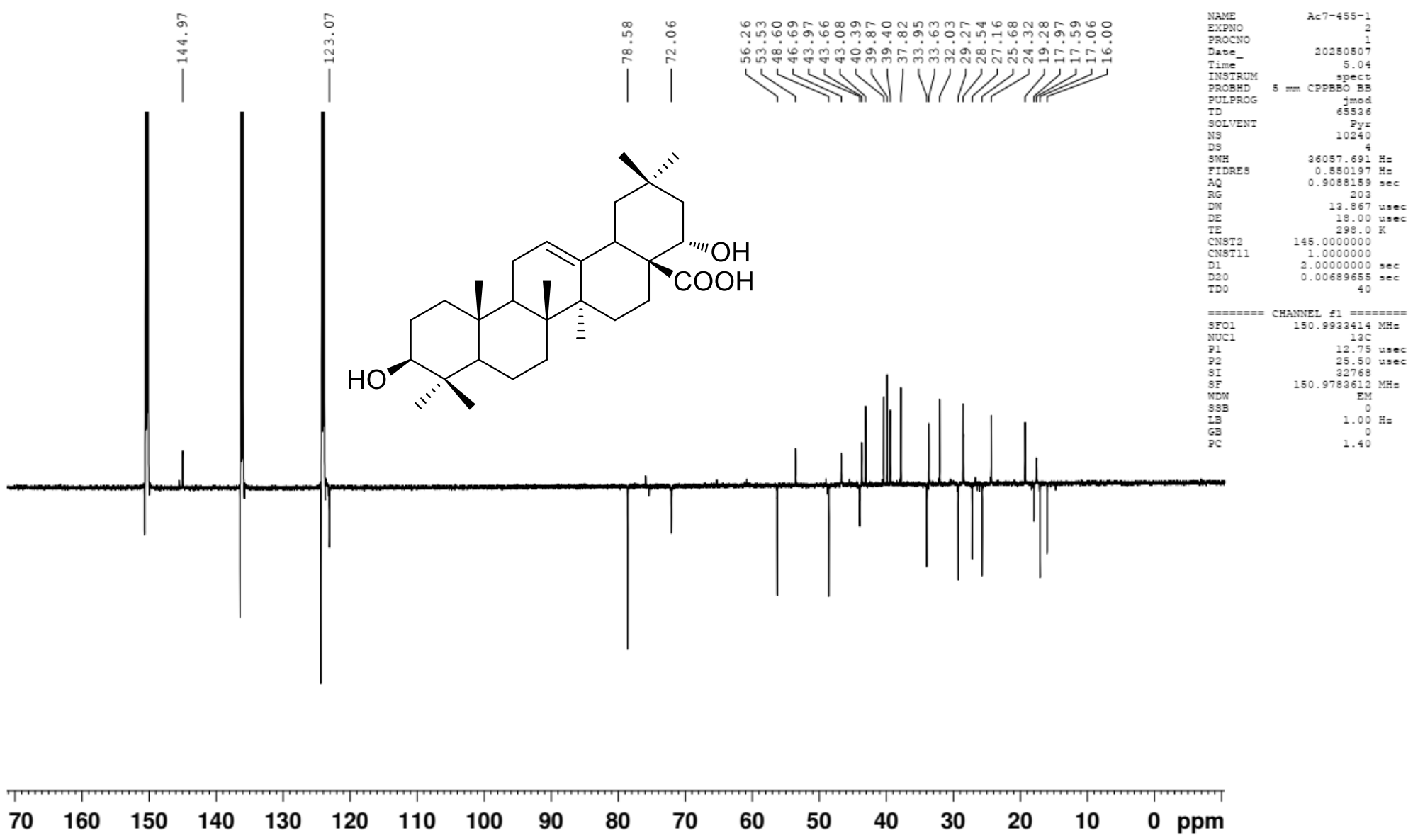

Supplementary Figure 12. ^{13}C APT spectrum of **8** in pyridine-d_5.

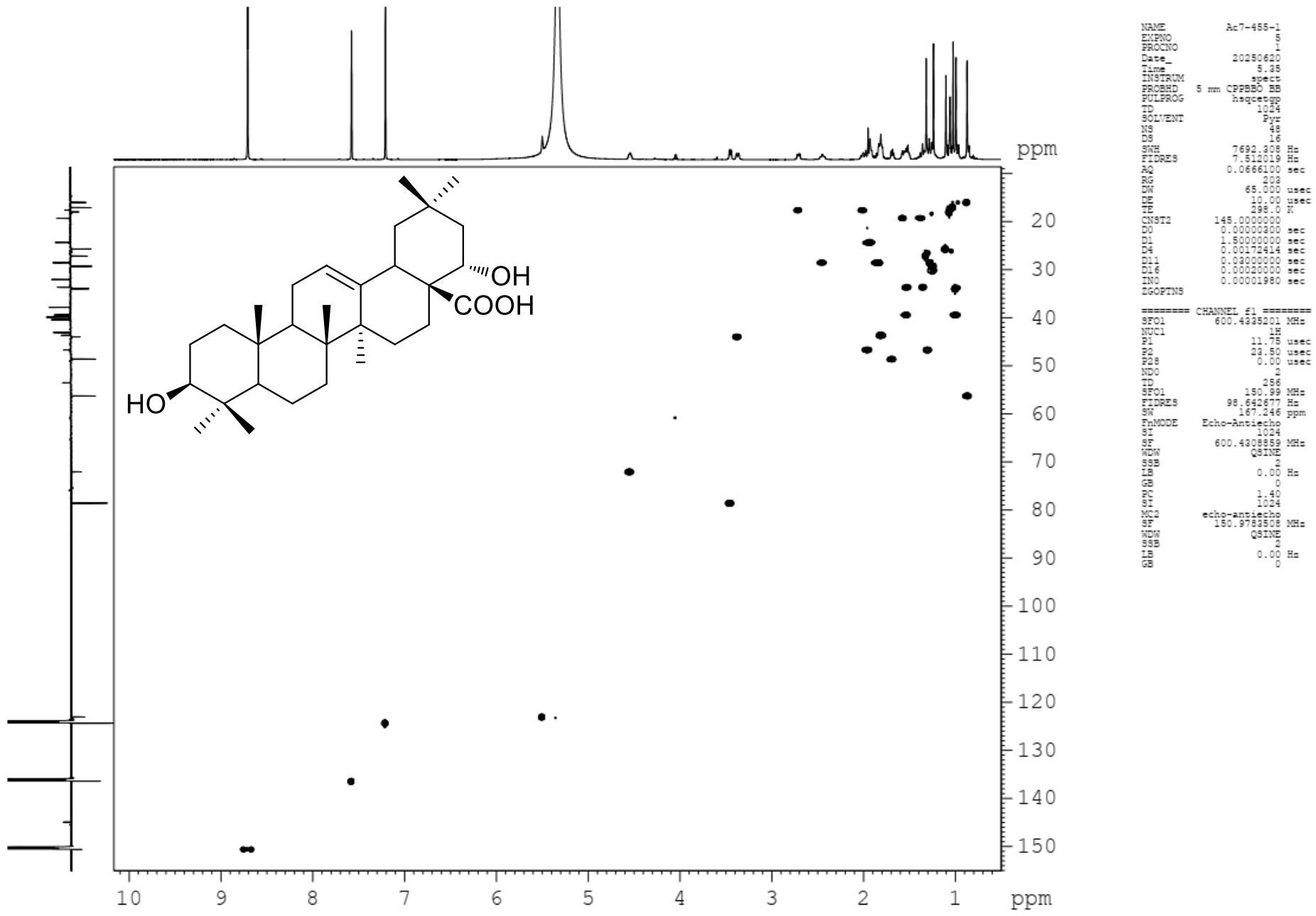

Supplementary Figure 13. HSQC spectrum of **8** in pyridine-d_5.

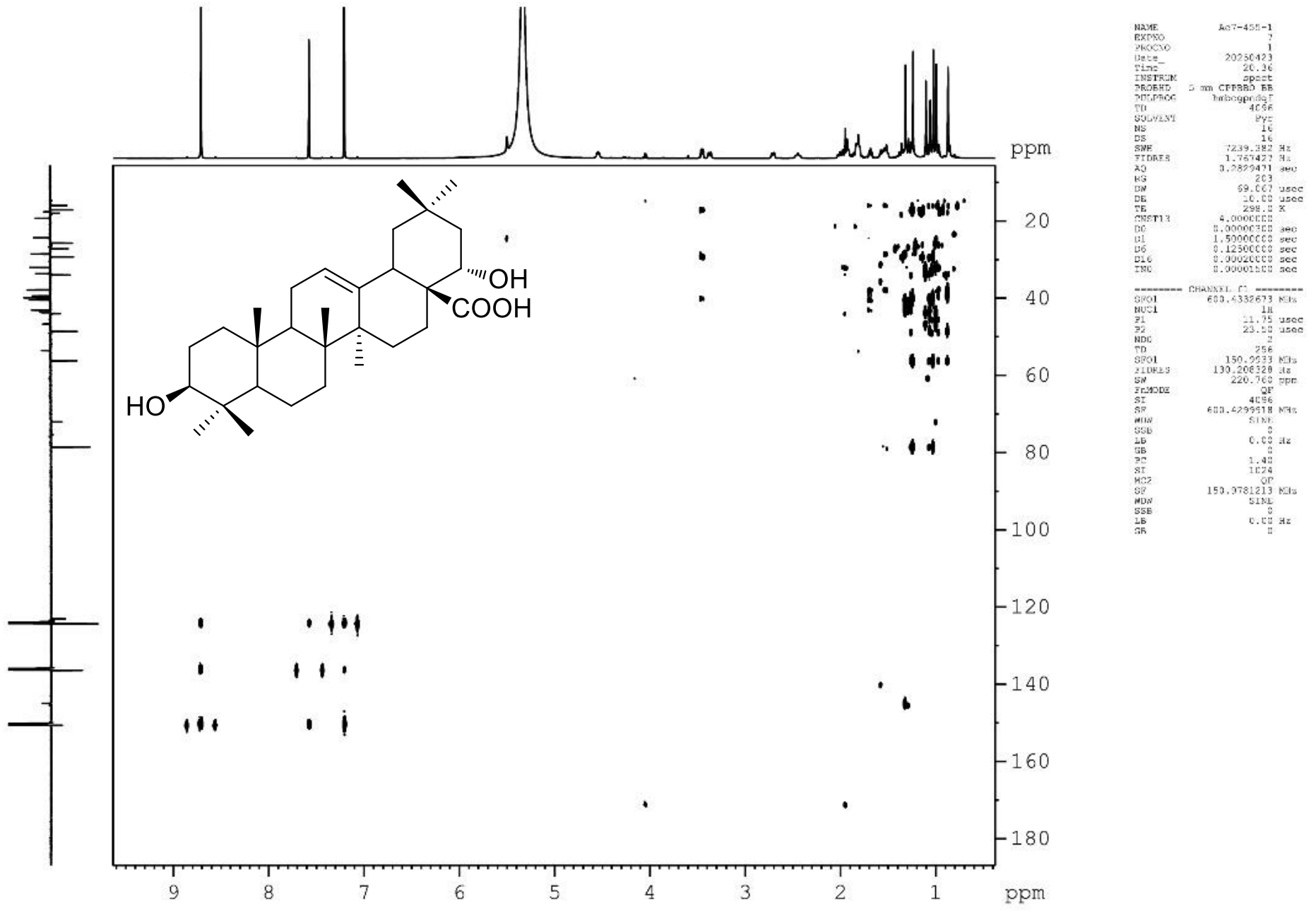

Supplementary Figure 14. HMBC spectrum of **8** in pyridine-d_5.

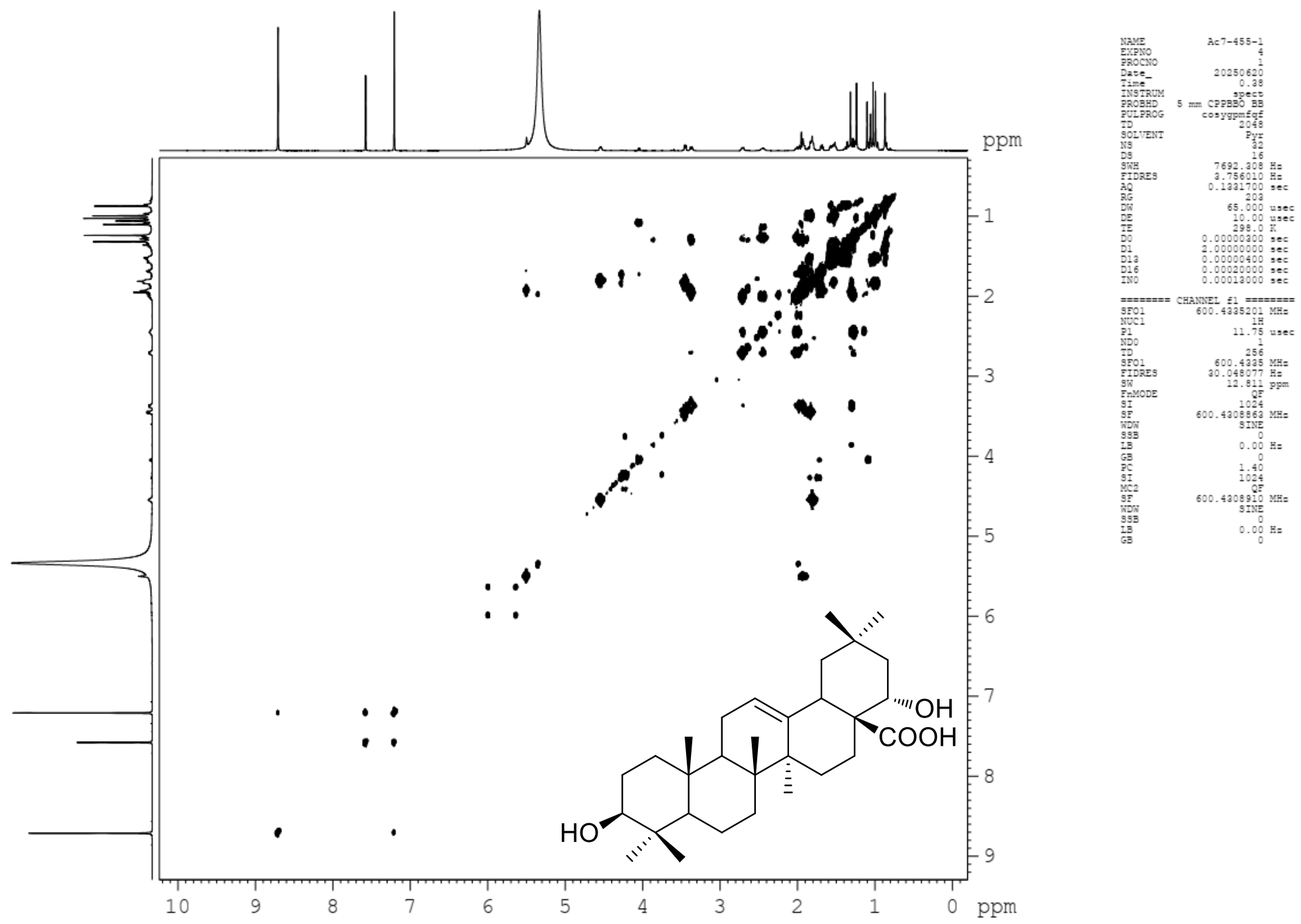

Supplementary Figure 15. COSY spectrum of **8** in pyridine-d_5.

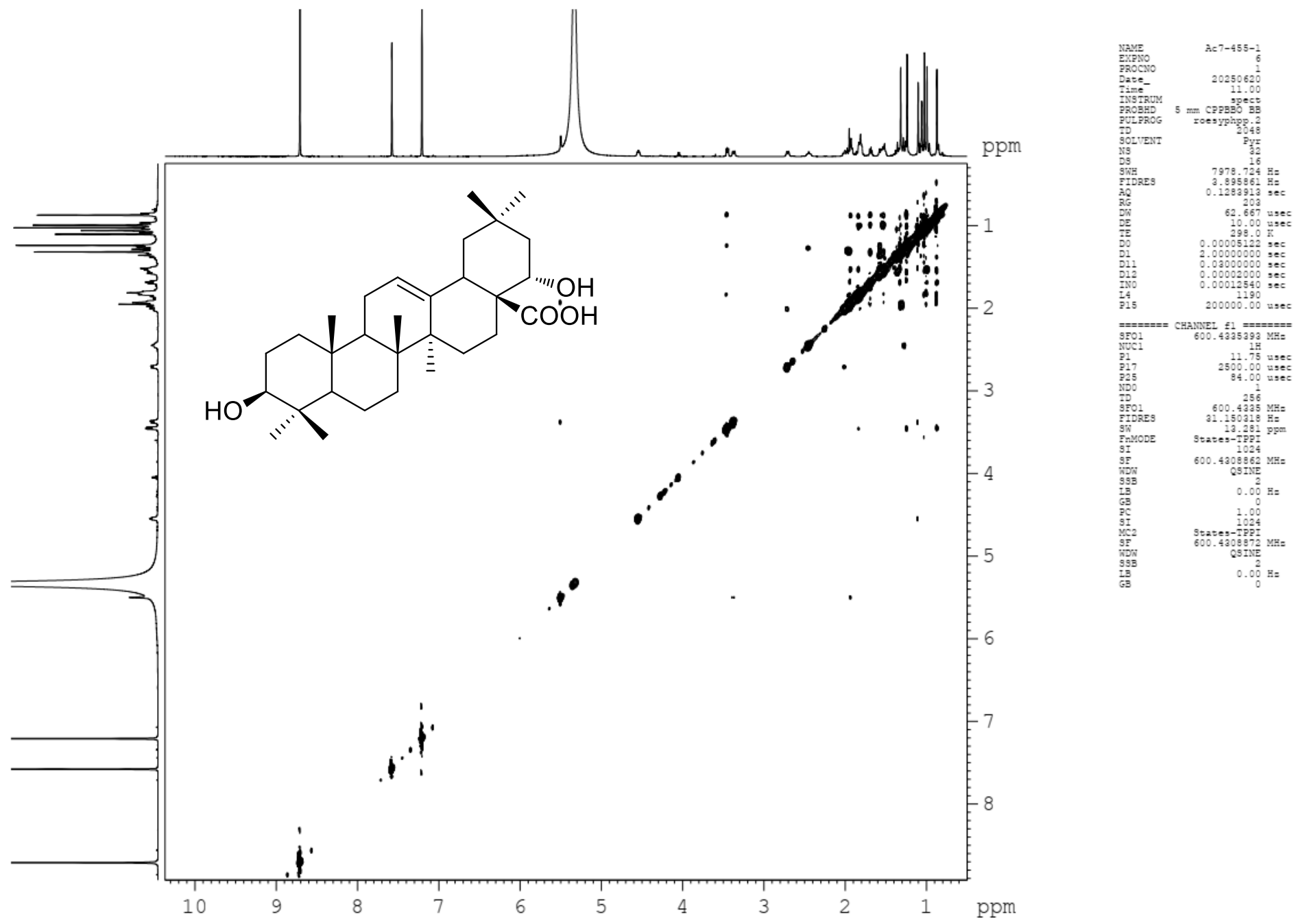

Supplementary Figure 16. ROESY spectrum of **8** in pyridine-d_5.

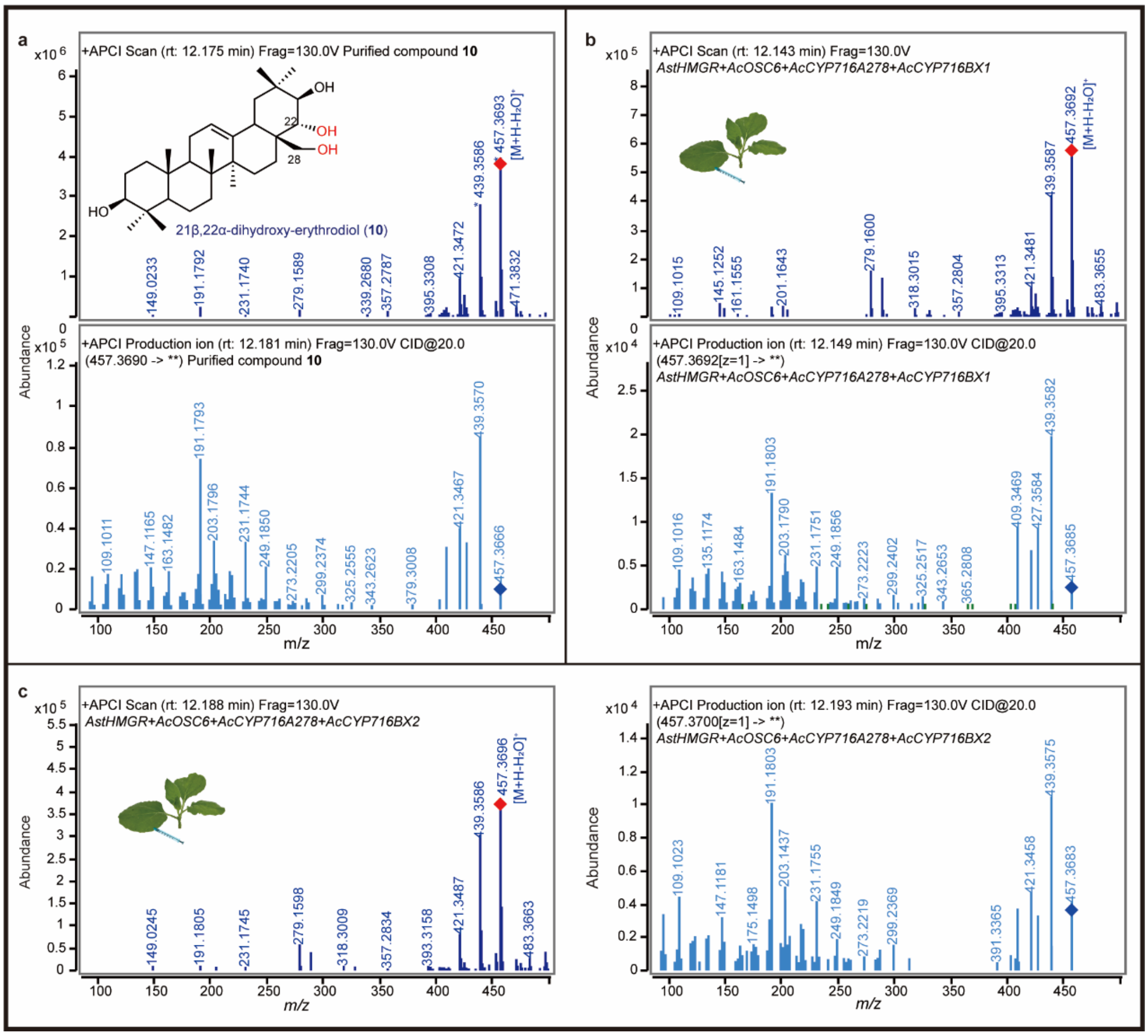

Supplementary Figure 17. The MS[1] and MS[2] spectra of 21β,22α-dihydroxy-erythrodiol (**10**). (**a**) The purified compound **10**. (**b**, **c**) The corresponding compound generated by *AstHMGR+AcOSC6+ AcCYP716A278+AcCYP716BX1* and *AstHMGR+AcOSC6+AcCYP716A278+AcCYP716BX2* in infiltrated *N. benthamiana*. The syringe schematic was created by Luz Eugenia Velasquez and obtained from Vecteezy (Vector Art ID: 14175787, Vecteezy Free License, https://www.vecteezy.com/vector-art/14175787-syringe-medical-drug).

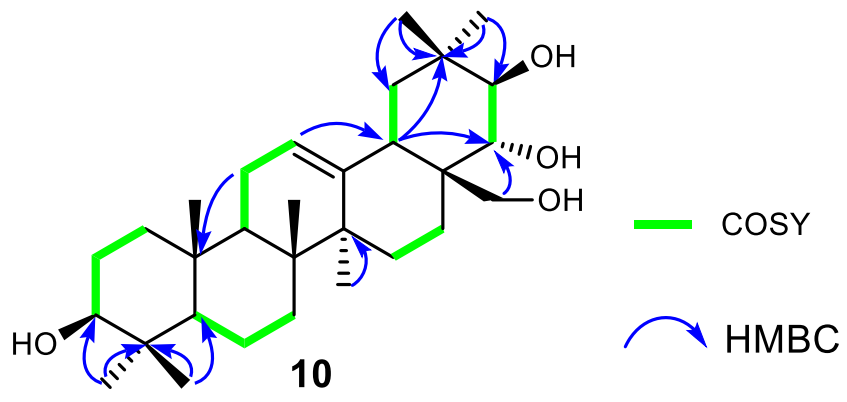

Supplementary Figure 18. Key COSY and HMBC correlations of **10**.

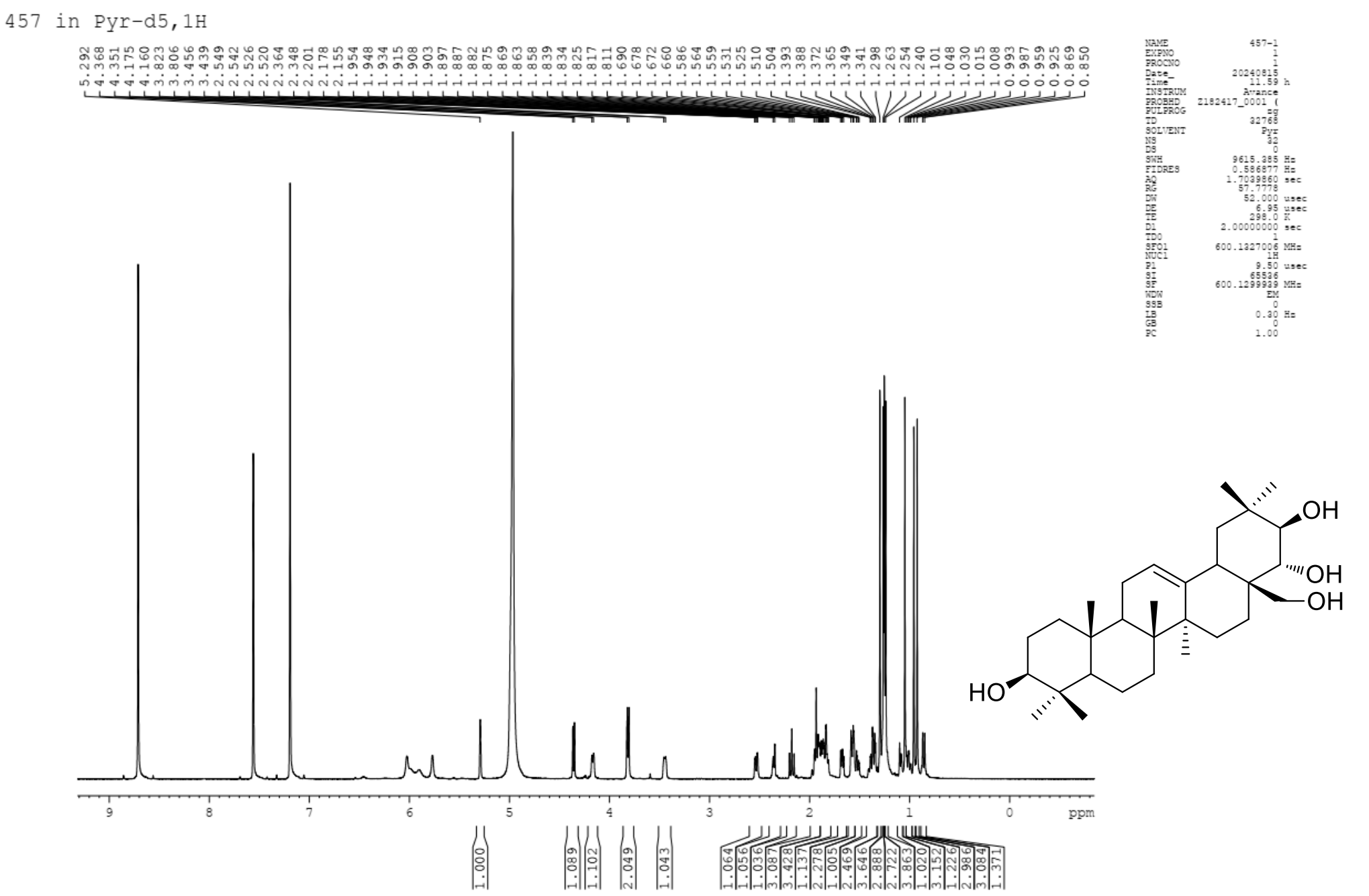

Supplementary Figure 19. ^{1}H NMR spectrum of **10** in pyridine-d_5.

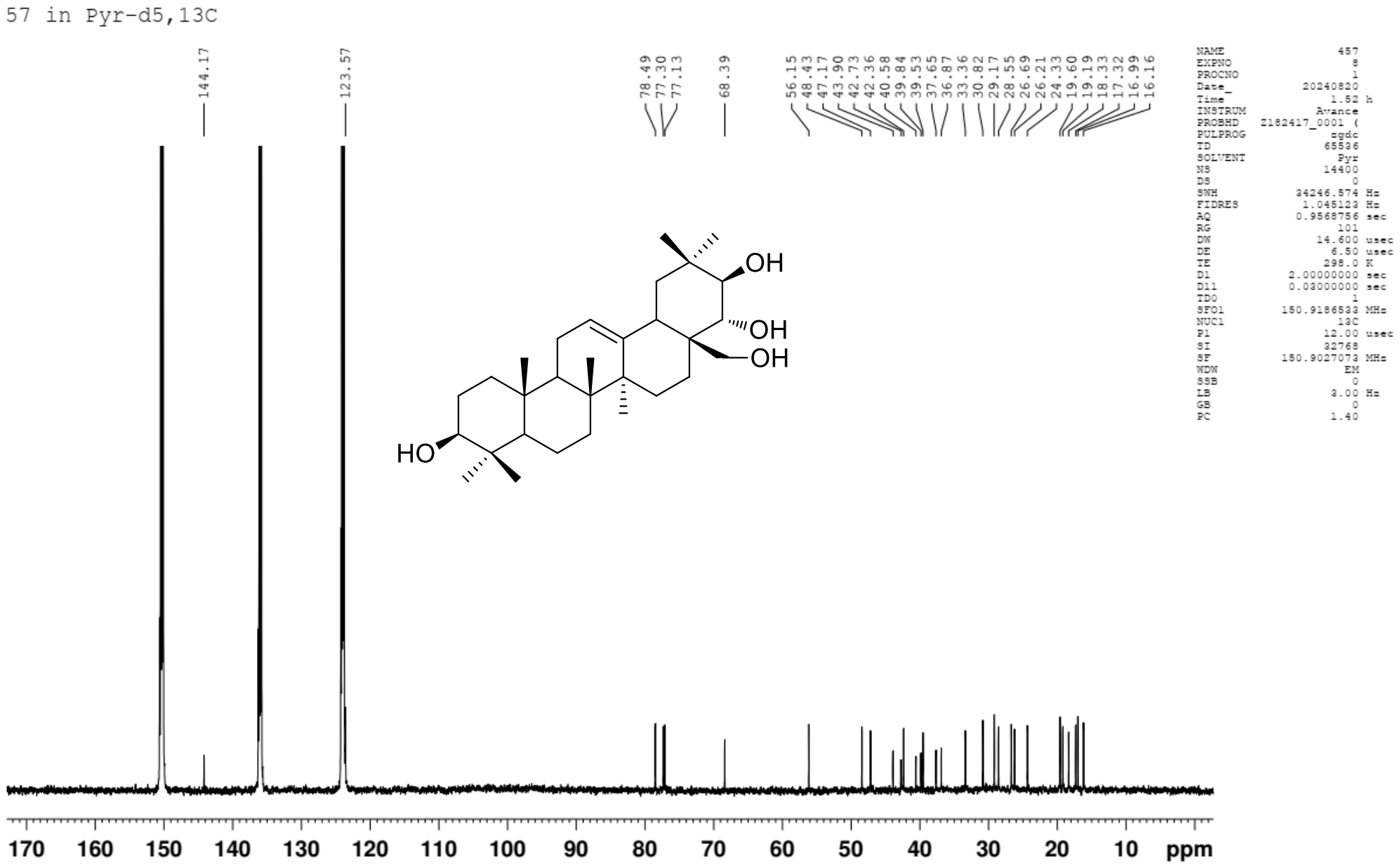

Supplementary Figure 20. ^{13}C NMR spectrum of **10** in pyridine-d_5.

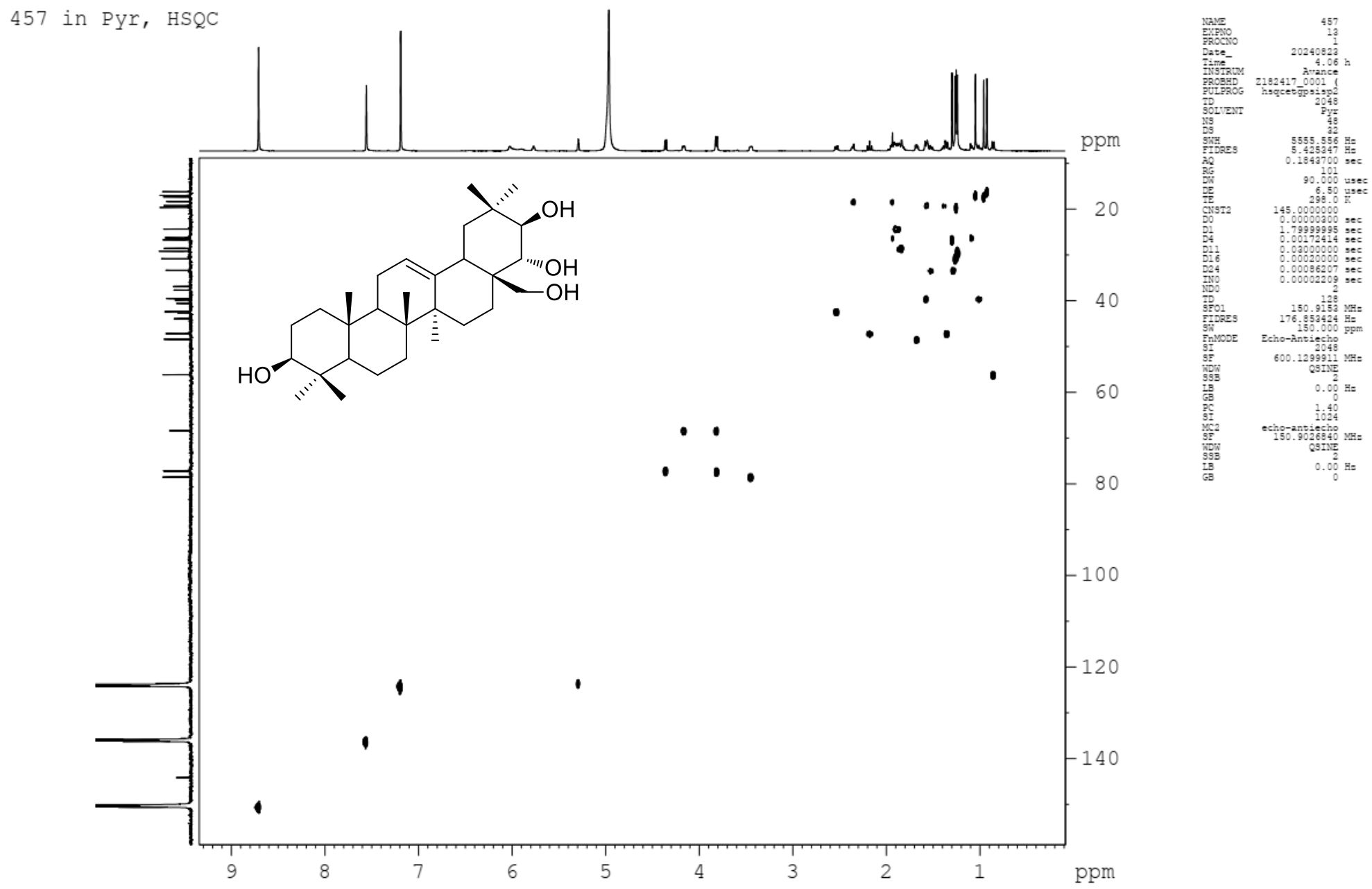

Supplementary Figure 21. HSQC Spectrum of **10** in pyridine-d_5.

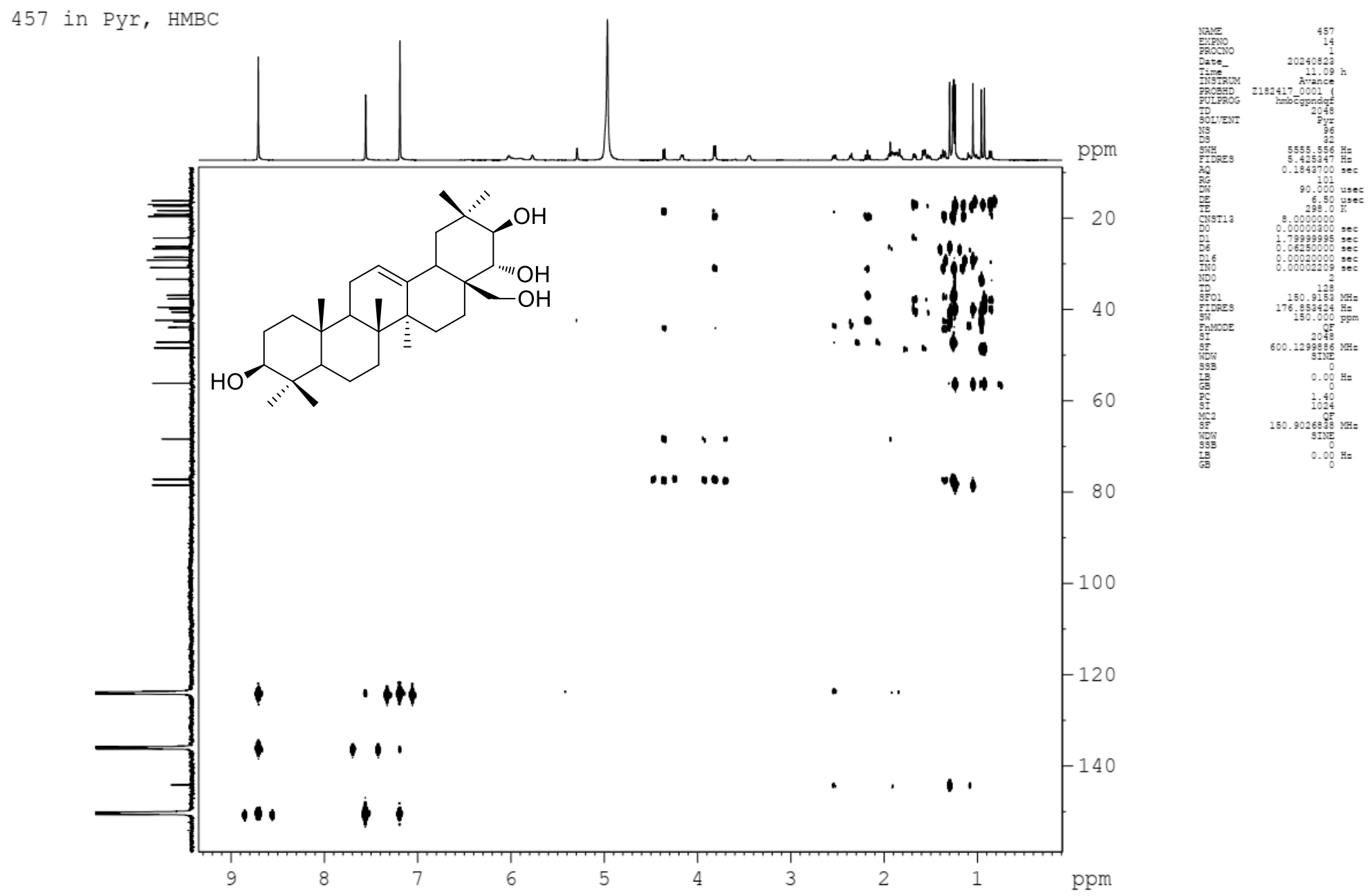

Supplementary Figure 22. HMBC Spectrum of **10** in pyridine-d_5.

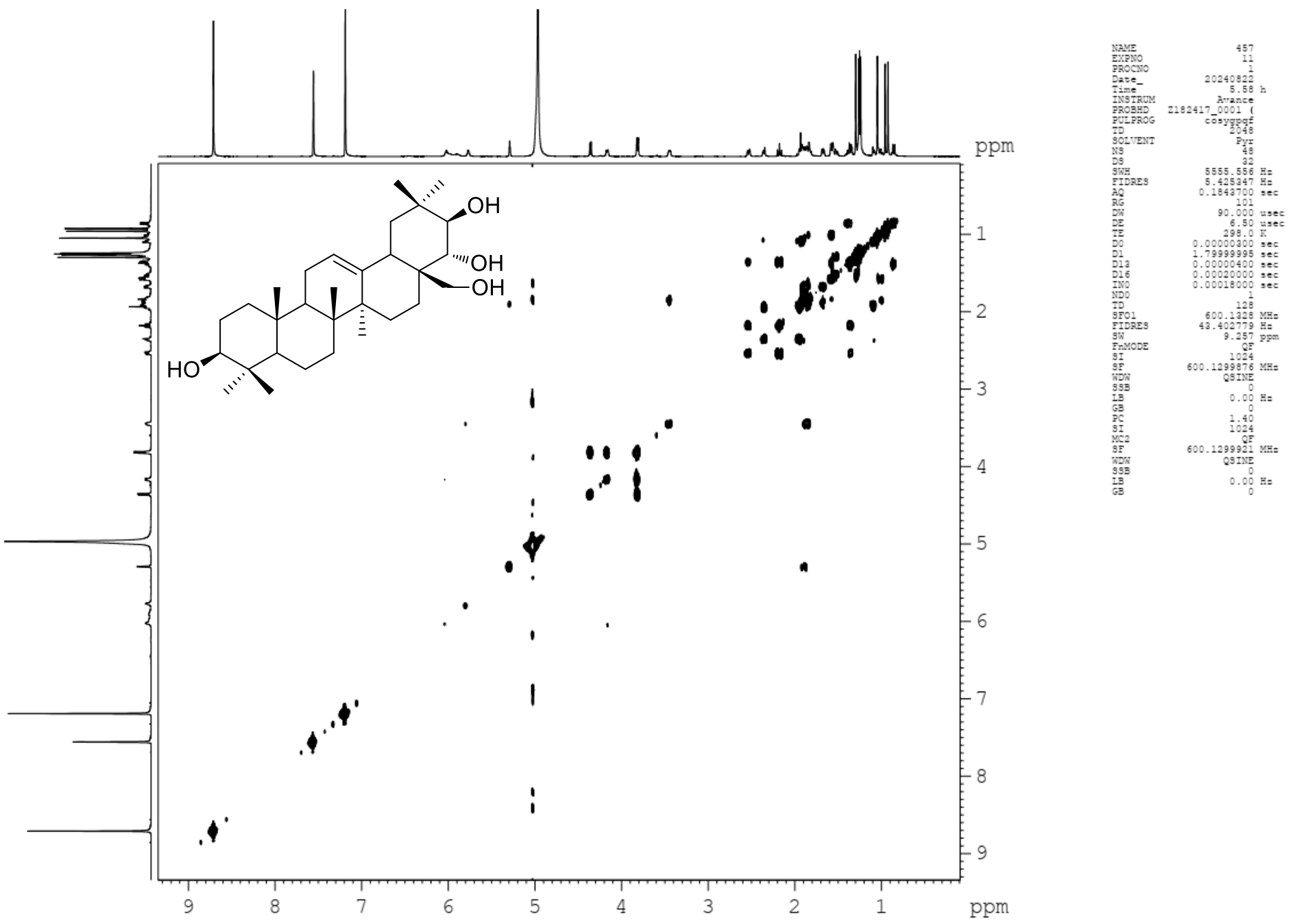

Supplementary Figure 23. COSY Spectrum of **10** in pyridine-d_5.

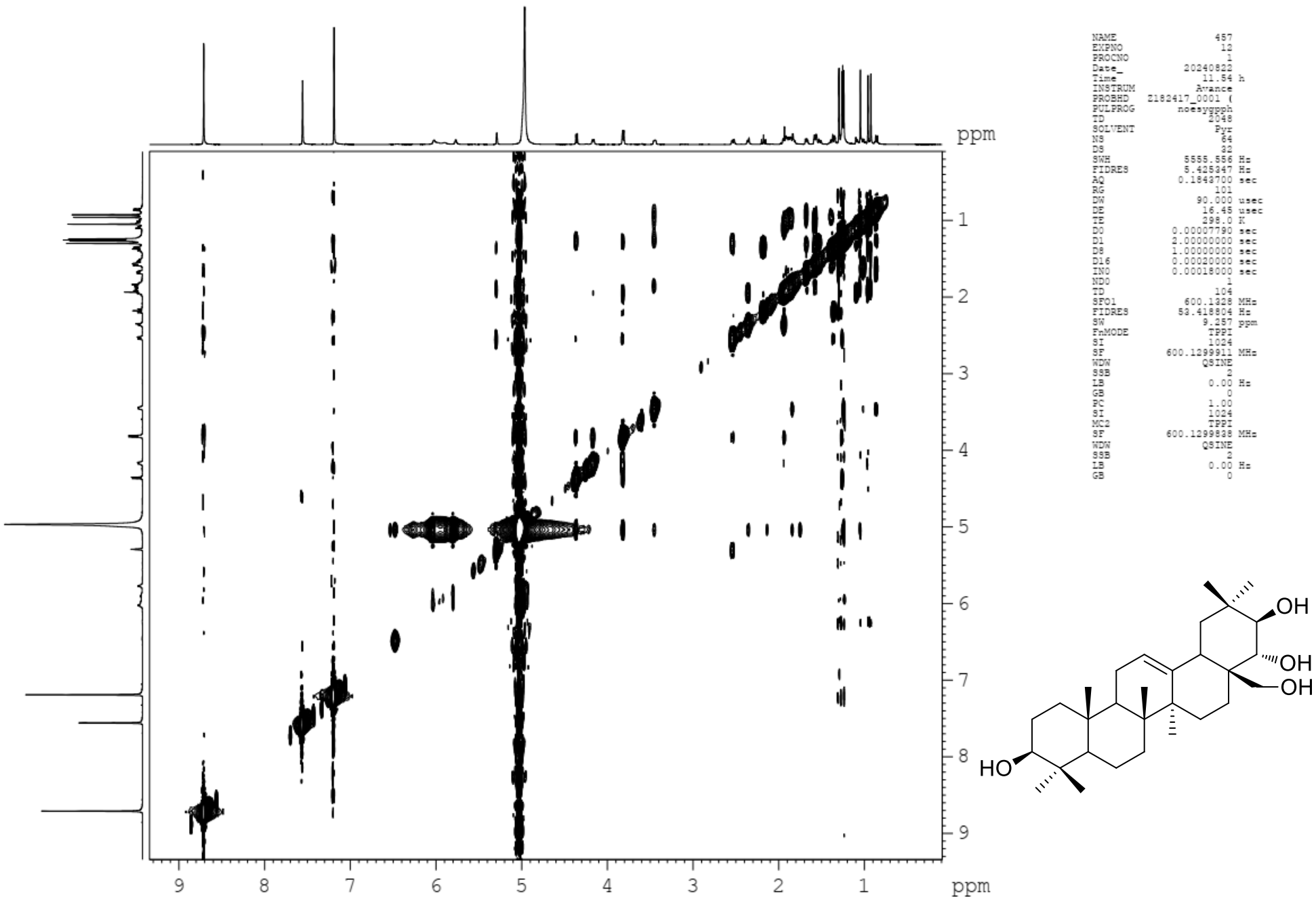

Supplementary Figure 24. NOESY Spectrum of **10** in pyridine-d_5.

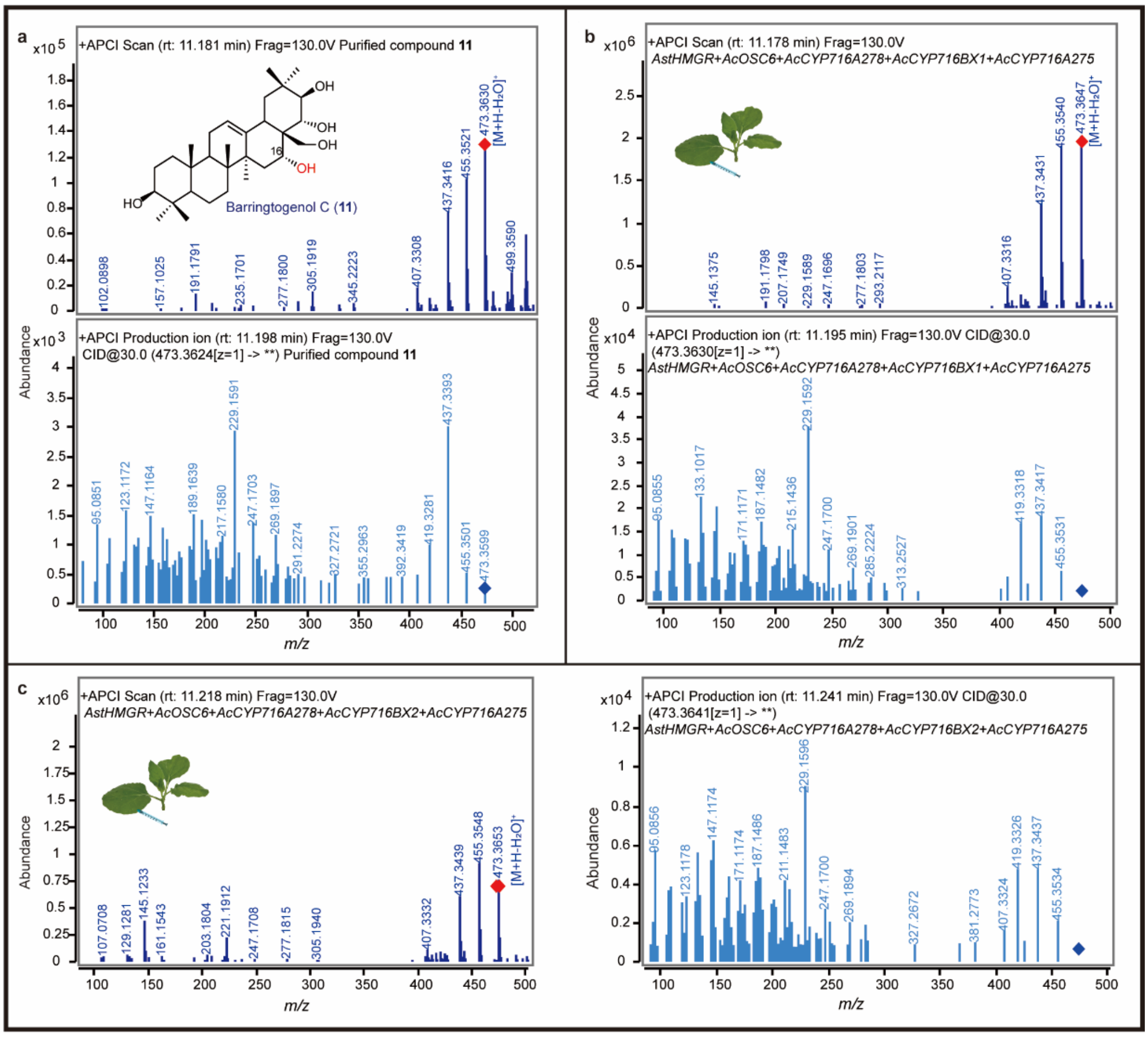

Supplementary Figure 25. The MS^1 and MS^2 spectra of barringtogenol C (**11**). (**a**) The purified compound **11**. (**b**, **c**) The corresponding compound generated by *AstHMGR+AcOSC6+AcCYP716A278+ AcCYP716BX1+AcCYP716A275* and *AstHMGR+AcOSC6+AcCYP716A278+AcCYP716BX2+ AcCYP716A275* in infiltrated *N. benthamiana*. The syringe schematic was created by Luz Eugenia Velasquez and obtained from Vecteezy (Vector Art ID: 14175787, Vecteezy Free License, https://www.vecteezy.com/vector-art/14175787-syringe-medical-drug).

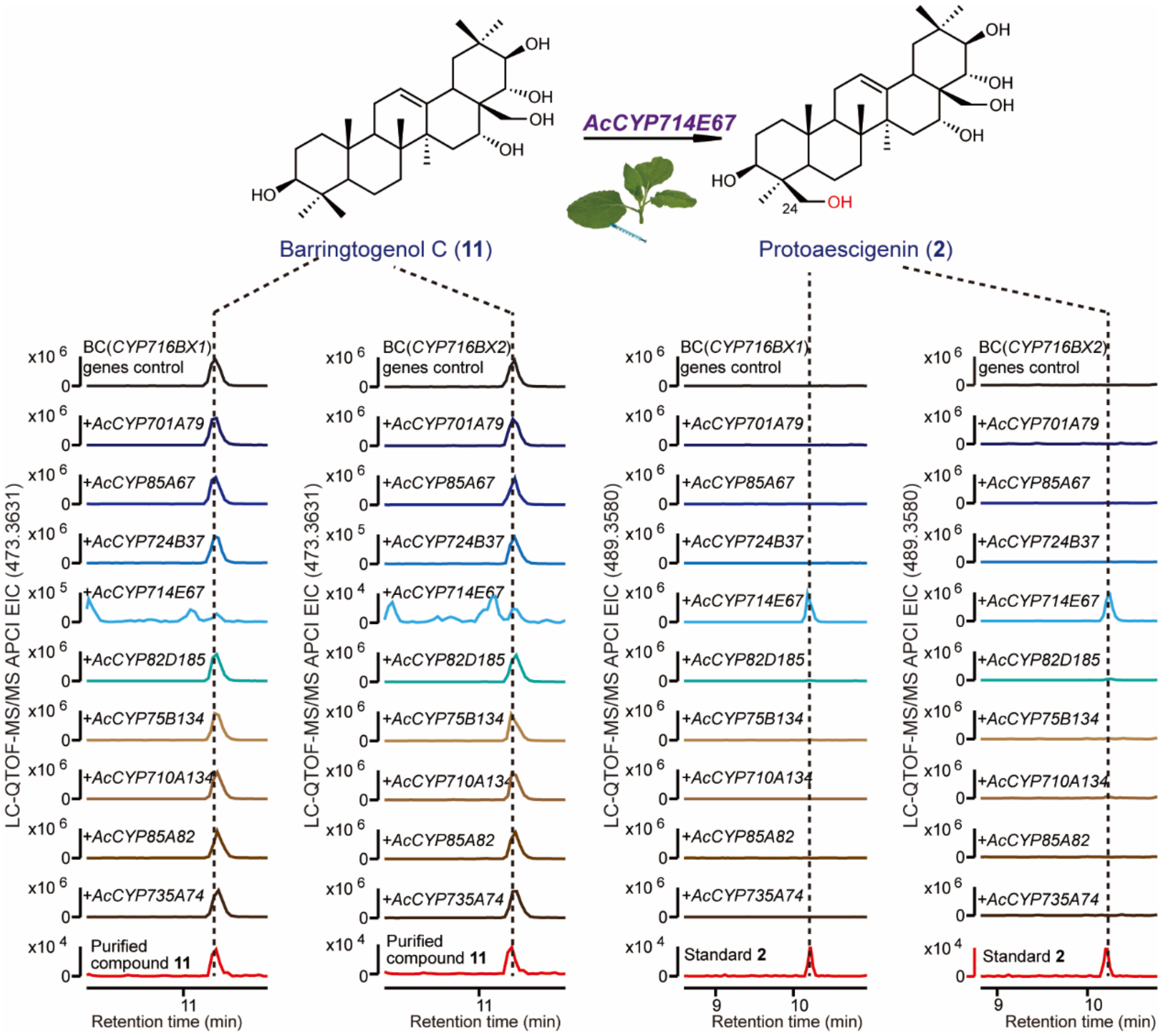

Supplementary Figure 26. Identification of *P450* genes involved in the C24 hydroxylation of barringtogenol C (**11**) to generate protoaescigenin in infiltrated *N. benthamiana*. Candidate *P450s* were respectively co-expressed with barringtogenol C pathway genes (*AstHMGR+AcOSC6+AcCYP716A278+AcCYP716BX1+AcCYP716A275* or *AstHMGR+AcOSC6+AcCYP716A278+AcCYP716BX2+ AcCYP716A275*). Overlay of EIC results showed that only co-expression of *AcCYP714E67* with BC genes can produce protoaescigenin (**2**, *m/z* 489.3580). The syringe schematic was created by Luz Eugenia Velasquez and obtained from Vecteezy (Vector Art ID: 14175787, Vecteezy Free License, https://www.vecteezy.com/vector-art/14175787-syringe-medical-drug).

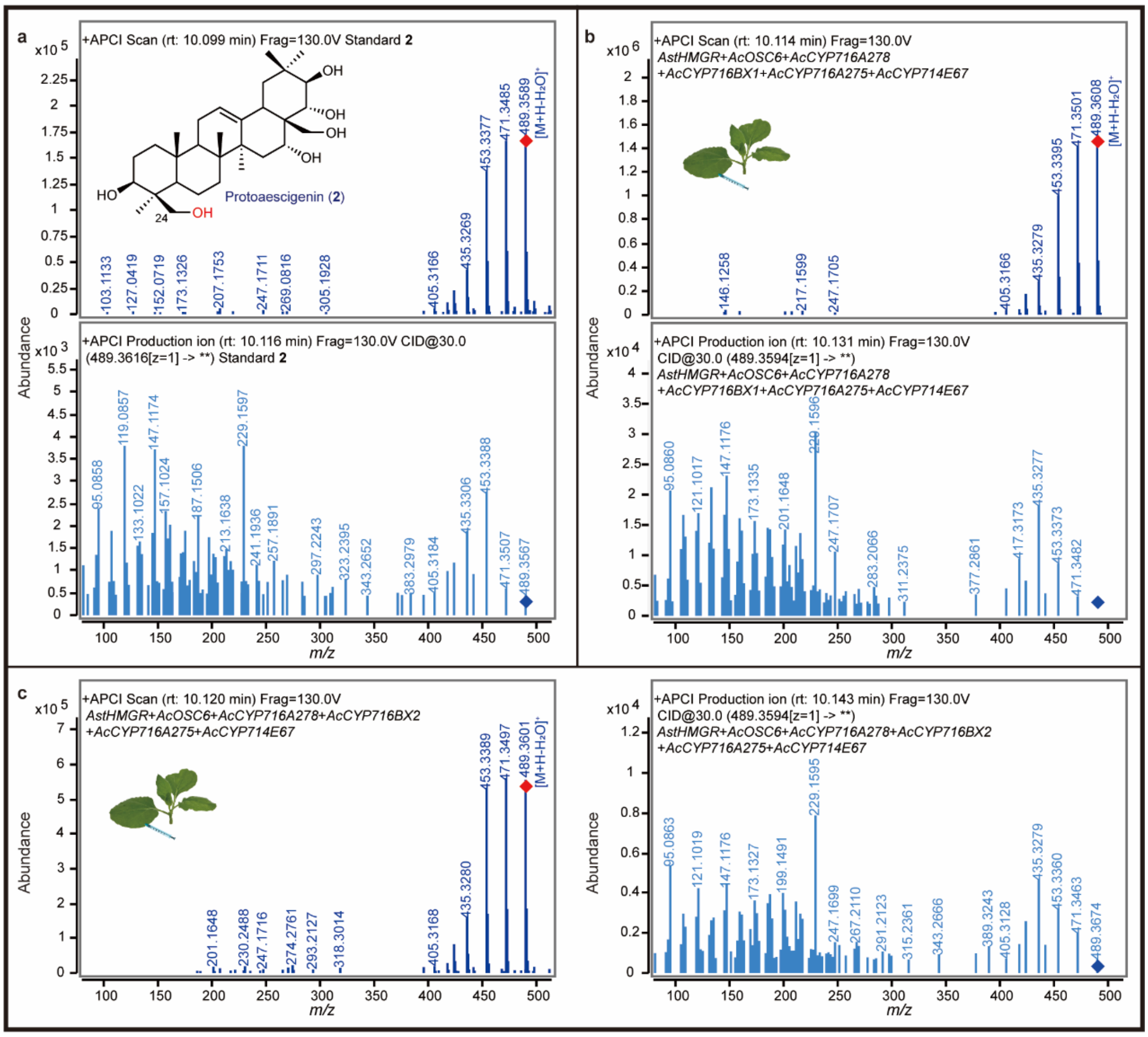

Supplementary Figure 27. The MS[1] and MS[2] spectra of protoaescigenin (**2**). (**a**) The standard **2**. (**b**, **c**) The corresponding product generated by *AstHMGR+AcOSC6+AcCYP716A278+AcCYP716BX1+AcCYP716A275+AcCYP714E67* and *AstHMGR+AcOSC6+AcCYP716A278+AcCYP716BX2+AcCYP716A275+AcCYP714E67* in infiltrated *N. benthamiana*. The syringe schematic was created by Luz Eugenia Velasquez and obtained from Vecteezy (Vector Art ID: 14175787, Vecteezy Free License, https://www.vecteezy.com/vector-art/14175787-syringe-medical-drug).

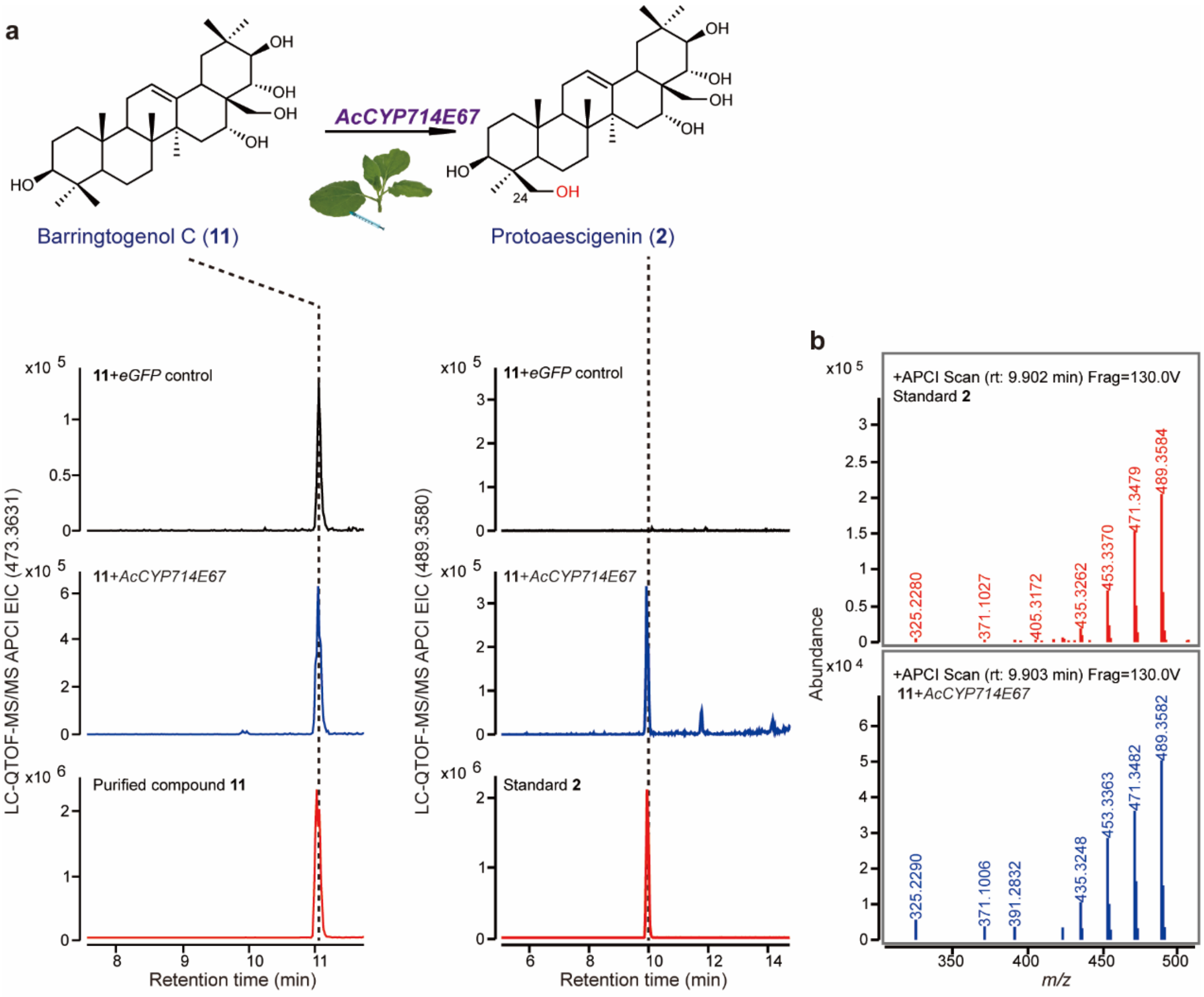

Supplementary Figure 28. Co-infiltration of compound **11** and *AcCYP714E67* into *N. benthamiana*. (**a**) Overlay of LC-QTOF-MS/MS extracted ion chromatograms for **11** and **2**. (**b**) Mass spectra of **2**. The syringe schematic was created by Luz Eugenia Velasquez and obtained from Vecteezy (Vector Art ID: 14175787, Vecteezy Free License, https://www.vecteezy.com/vector-art/14175787-syringe-medical-drug).

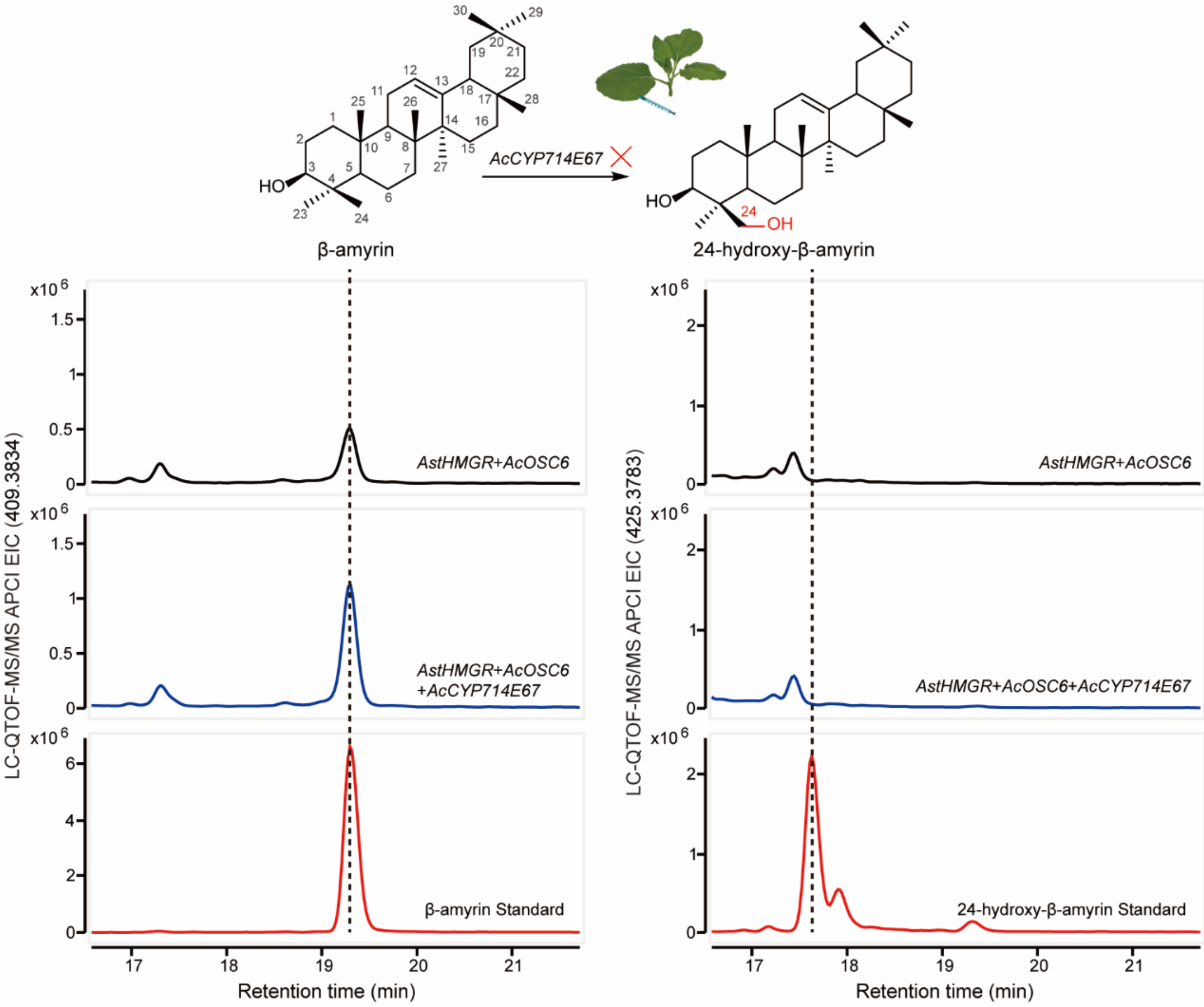

Supplementary Figure 29. Co-infiltration of *AstHMGR*, *AcOSC6* and *AcCYP714E67* into *N. benthamiana*. This co-infiltration only yielded β-amyrin and no 24-hydroxy-β-amyrin was detected. These results indicate *AcCYP714E67* cannot catalyze the hydroxylation of β-amyrin at the C-24 position. The syringe schematic was created by Luz Eugenia Velasquez and obtained from Vecteezy (Vector Art ID: 14175787, Vecteezy Free License, https://www.vecteezy.com/vector-art/14175787-syringe-medical-drug).

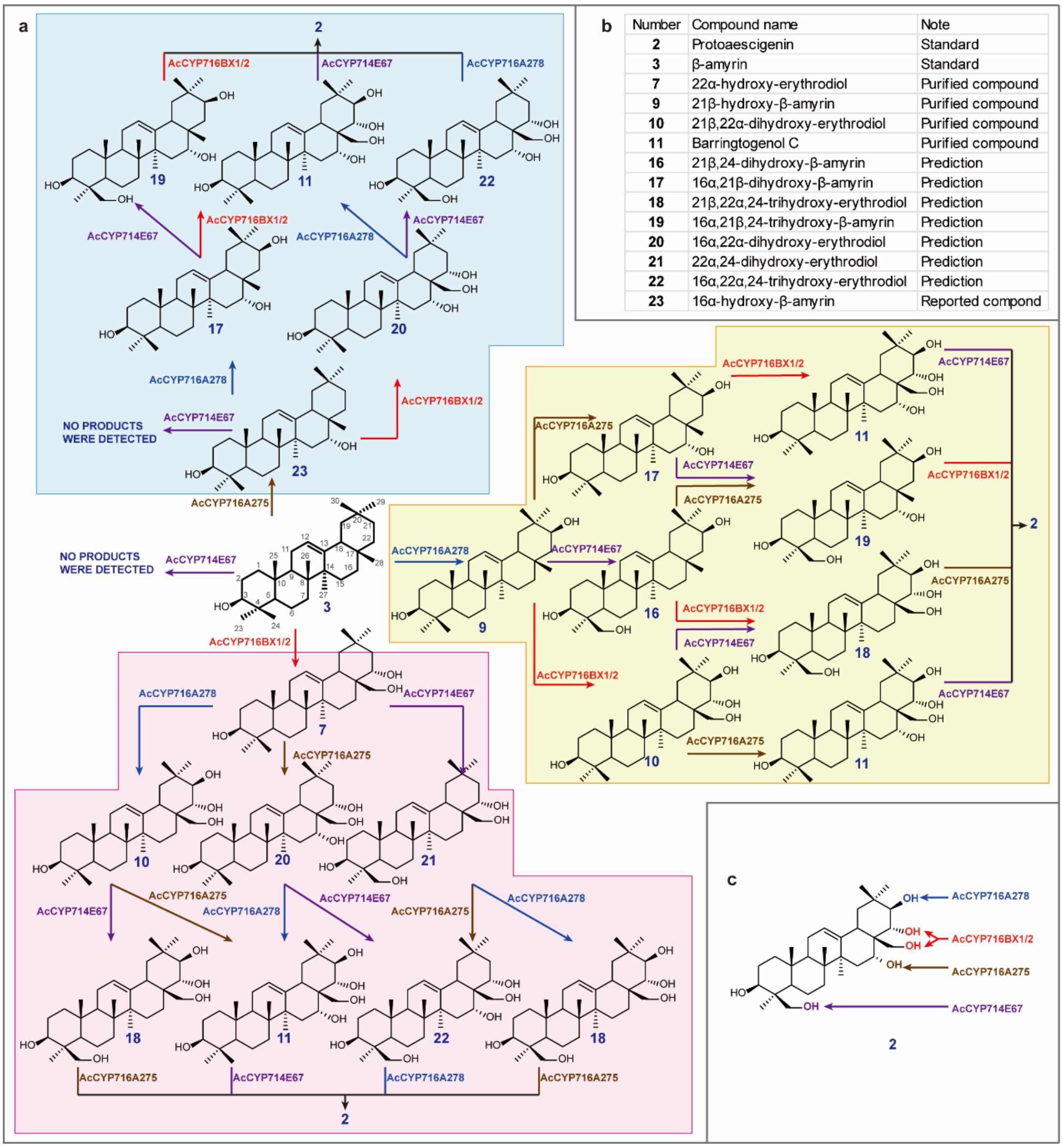

Number	Compound name	Note
2	Protoaescigenin	Standard
3	β-amyrin	Standard
7	22α-hydroxy-erythrodiol	Purified compound
9	21β-hydroxy-β-amyrin	Purified compound
10	21β,22α-dihydroxy-erythrodiol	Purified compound
11	Barringtogenol C	Purified compound
16	21β,24-dihydroxy-β-amyrin	Prediction
17	16α,21β-dihydroxy-β-amyrin	Prediction
18	21β,22α,24-trihydroxy-erythrodiol	Prediction
19	16α,21β,24-trihydroxy-β-amyrin	Prediction
20	16α,22α-dihydroxy-erythrodiol	Prediction
21	22α,24-dihydroxy-erythrodiol	Prediction
22	16α,22α,24-trihydroxy-erythrodiol	Prediction
23	16α-hydroxy-β-amyrin	Reported compond

Supplementary Figure 30. Complex metabolic network for the biosynthesis of protoaescigenin (**2**) from β-amyrin (**3**). (**a**) Schematic diagram of the metabolic network; (**b**) Related intermediates; (**c**) Schematic diagram of hydroxylation sites catalyzed by P450s.

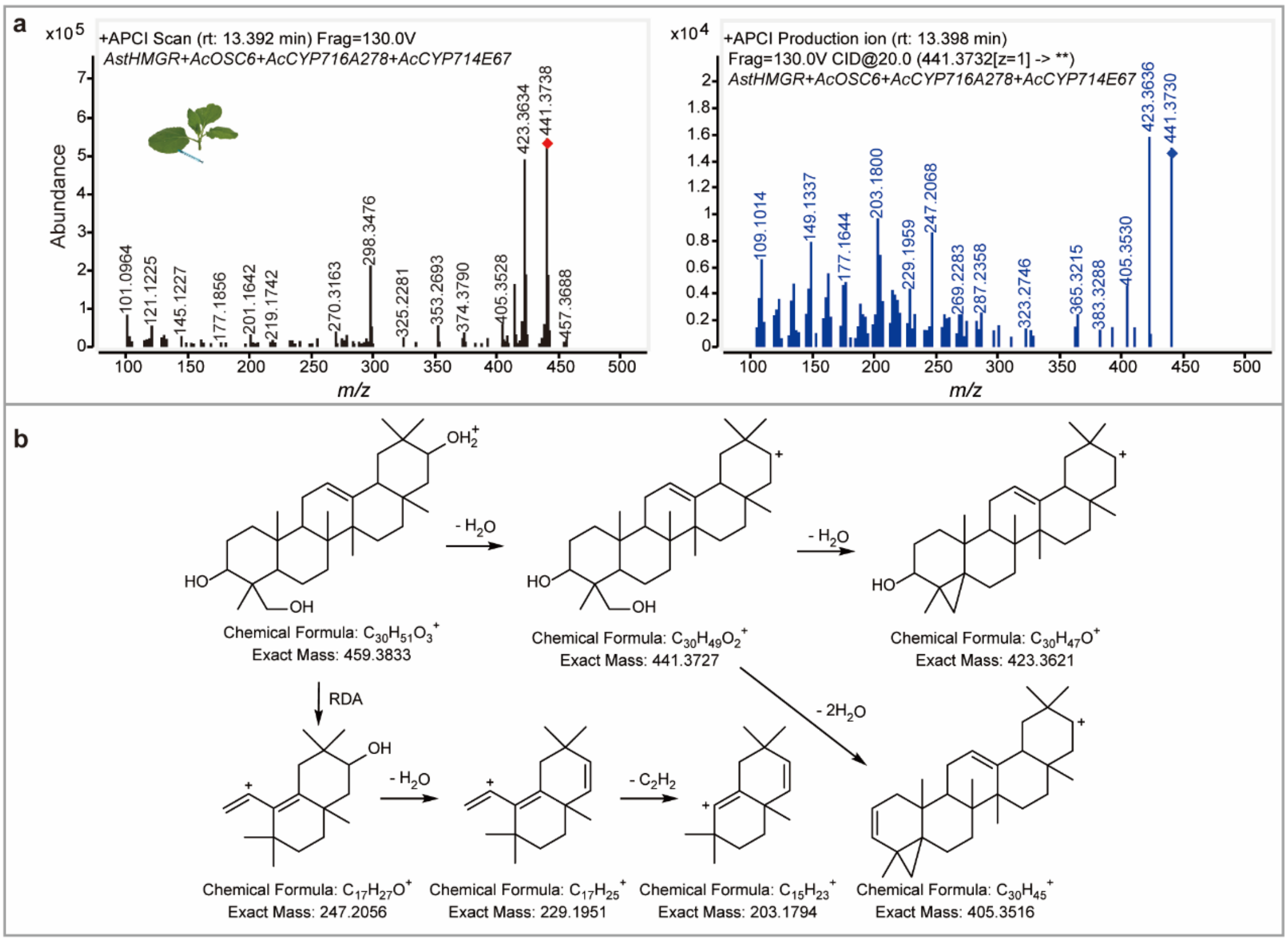

Supplementary Figure 31. Mass spectra (**a**) and proposed fragmentation pathways (**b**) of 21β,24-dihydroxy-β-amyrin (**16**) produced in infiltrated *N. benthamiana*. The syringe schematic was created by Luz Eugenia Velasquez and obtained from Vecteezy (Vector Art ID: 14175787, Vecteezy Free License, https://www.vecteezy.com/vector-art/14175787-syringe-medical-drug).

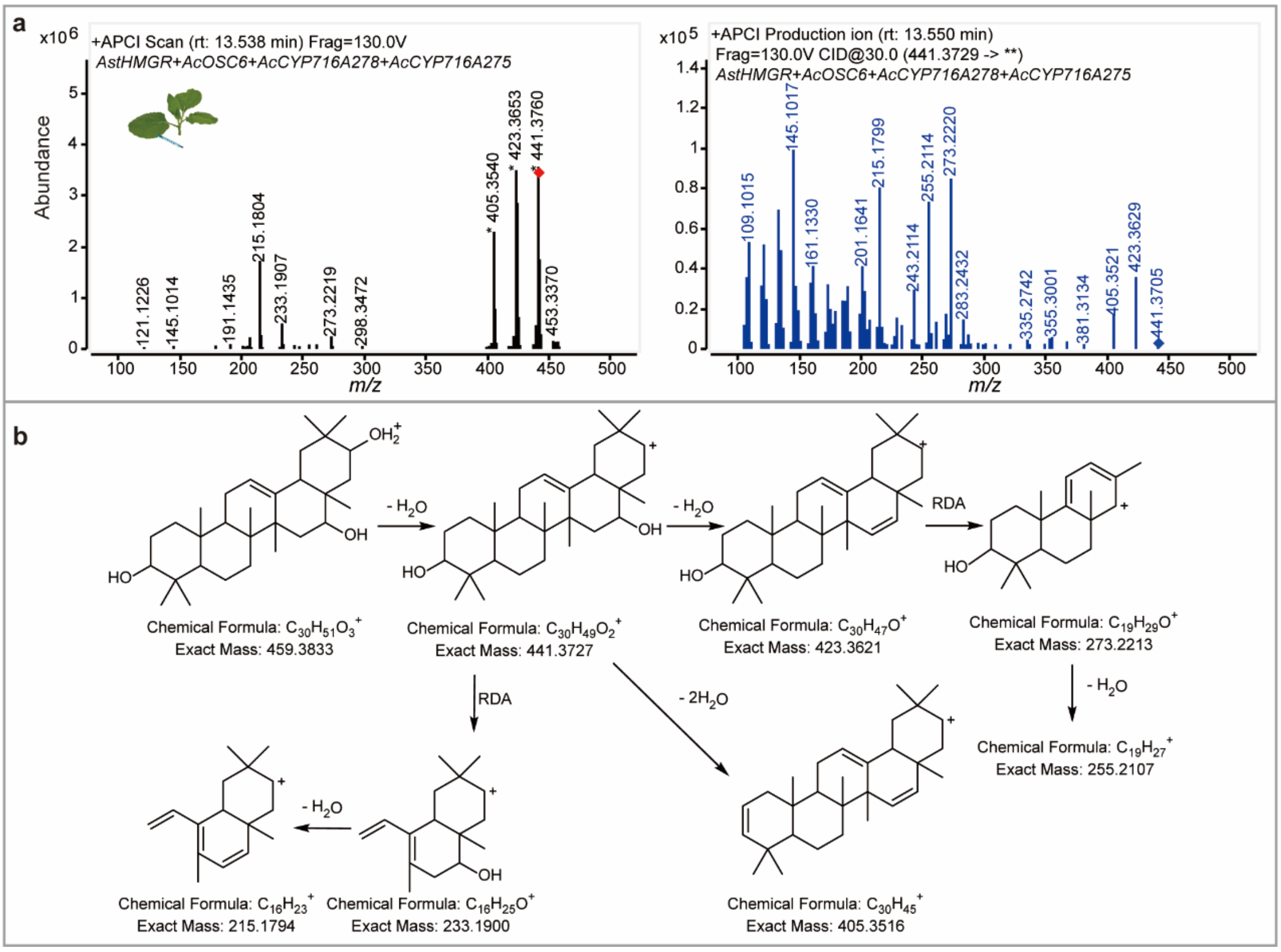

Supplementary Figure 32. Mass spectra (**a**) and proposed fragmentation pathways (**b**) of 16α,21β-dihydroxy-β-amyrin (**17**) produced in infiltrated *N. benthamiana*. The syringe schematic was created by Luz Eugenia Velasquez and obtained from Vecteezy (Vector Art ID: 14175787, Vecteezy Free License, https://www.vecteezy.com/vector-art/14175787-syringe-medical-drug).

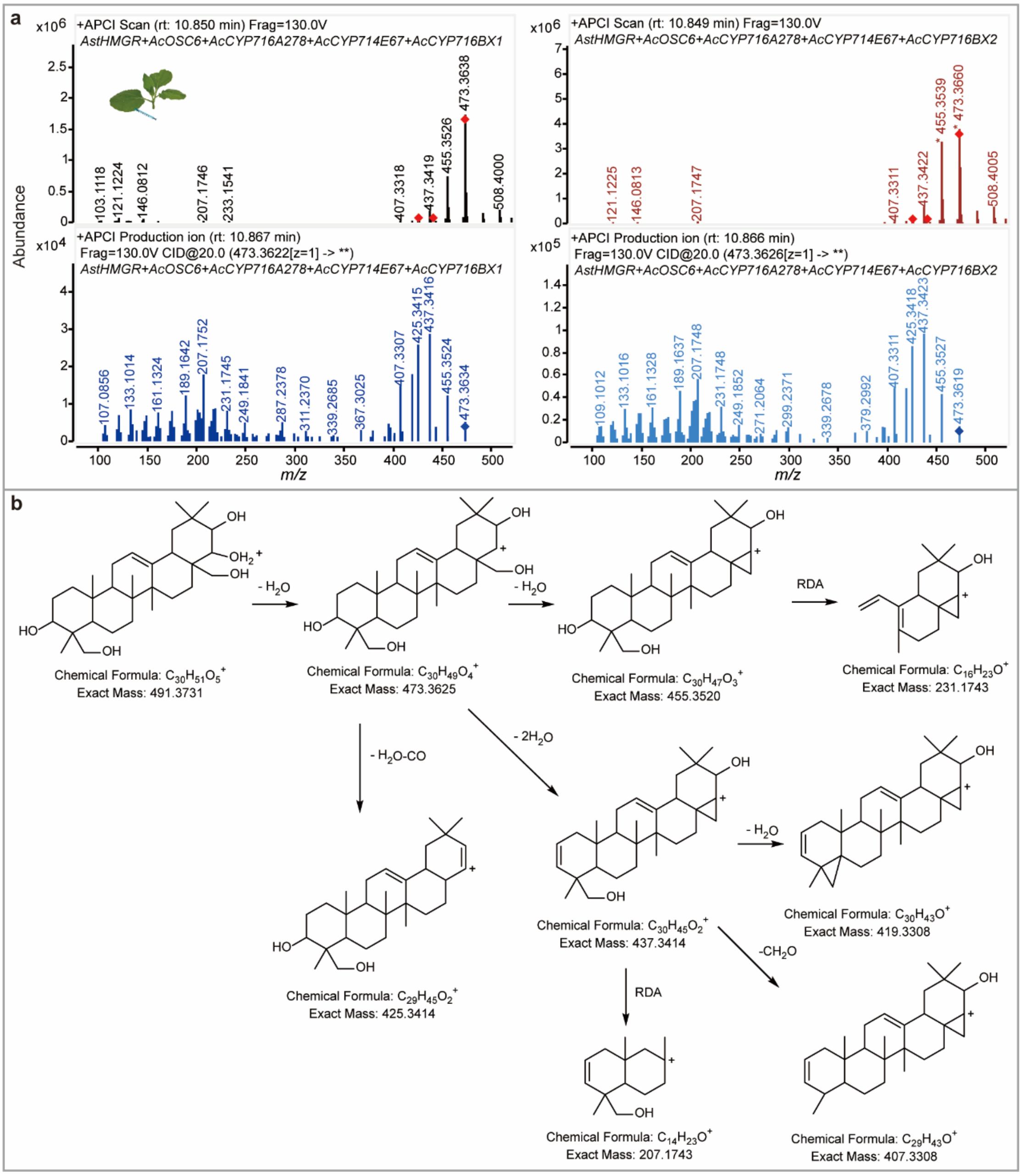

Supplementary Figure 33. Mass spectra (**a**) and proposed fragmentation pathways (**b**) of 21β,22α,24-trihydroxy-erythrodiol (**18**) produced in infiltrated *N. benthamiana*. The syringe schematic was created by Luz Eugenia Velasquez and obtained from Vecteezy (Vector Art ID: 14175787, Vecteezy Free License, https://www.vecteezy.com/vector-art/14175787-syringe-medical-drug).

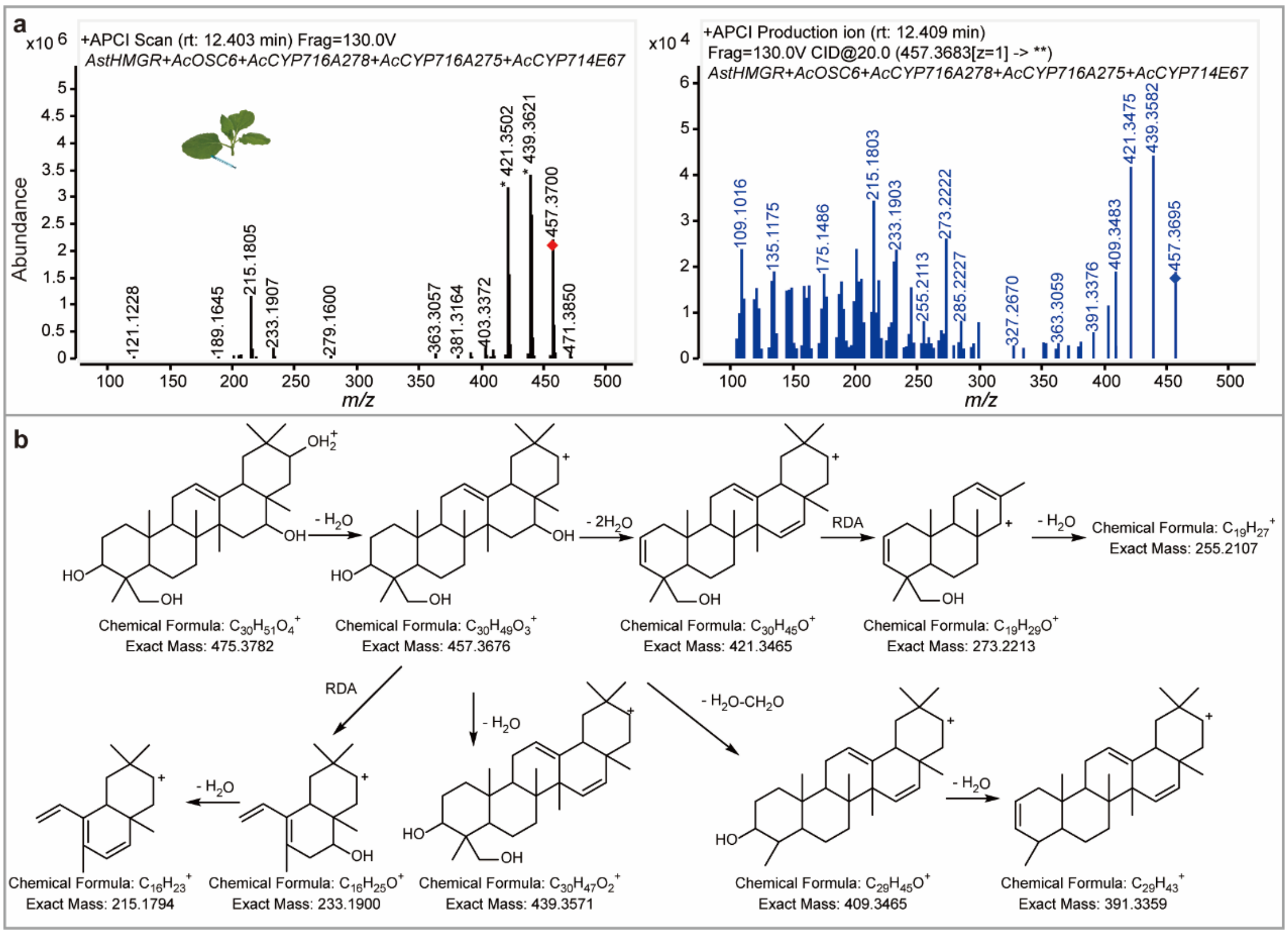

Supplementary Figure 34. Mass spectra (**a**) and proposed fragmentation pathways (**b**) of 16α,21β,24-trihydroxy-β-amyrin (**19**) produced in infiltrated *N. benthamiana*. The syringe schematic was created by Luz Eugenia Velasquez and obtained from Vecteezy (Vector Art ID: 14175787, Vecteezy Free License, https://www.vecteezy.com/vector-art/14175787-syringe-medical-drug).

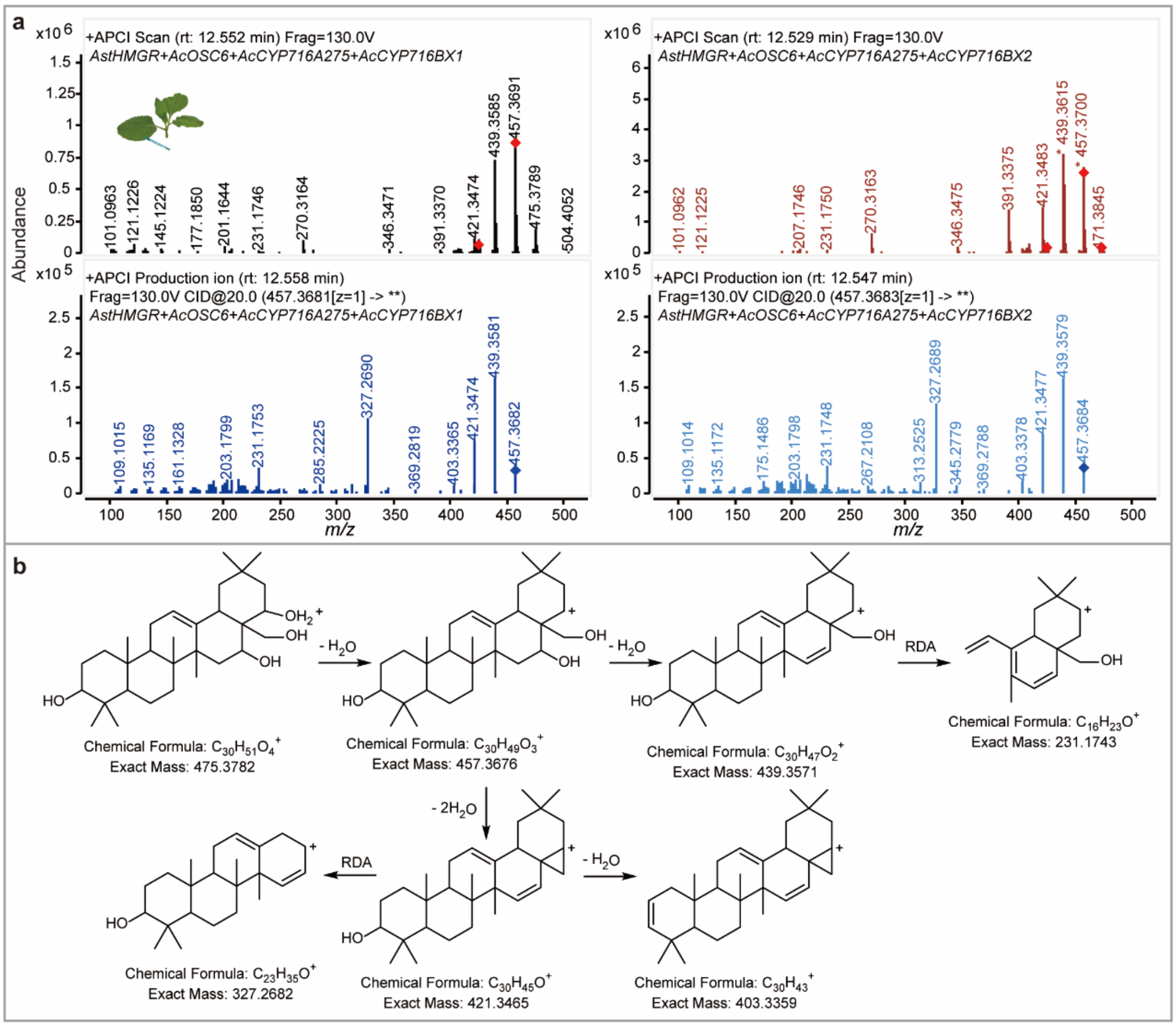

Supplementary Figure 35. Mass spectra (**a**) and proposed fragmentation pathways (**b**) of 16α,22α-dihydroxy-erythrodiol (**20**) produced in infiltrated *N. benthamiana*. The syringe schematic was created by Luz Eugenia Velasquez and obtained from Vecteezy (Vector Art ID: 14175787, Vecteezy Free License, https://www.vecteezy.com/vector-art/14175787-syringe-medical-drug).

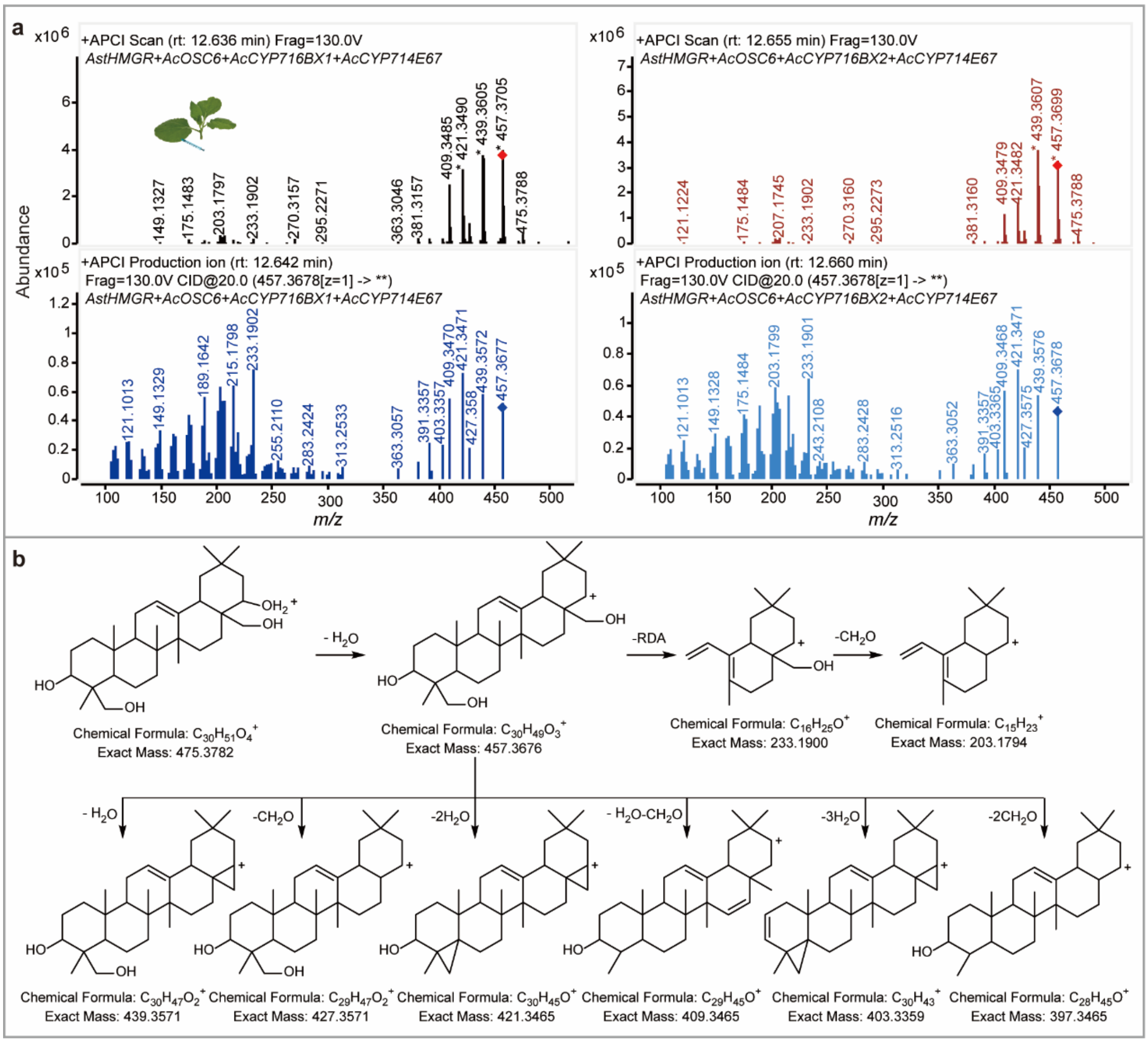

Supplementary Figure 36. Mass spectra (**a**) and proposed fragmentation pathways (**b**) of 22α,24-dihydroxy-erythrodiol (**21**) produced in infiltrated *N. benthamiana*. The syringe schematic was created by Luz Eugenia Velasquez and obtained from Vecteezy (Vector Art ID: 14175787, Vecteezy Free License, https://www.vecteezy.com/vector-art/14175787-syringe-medical-drug).

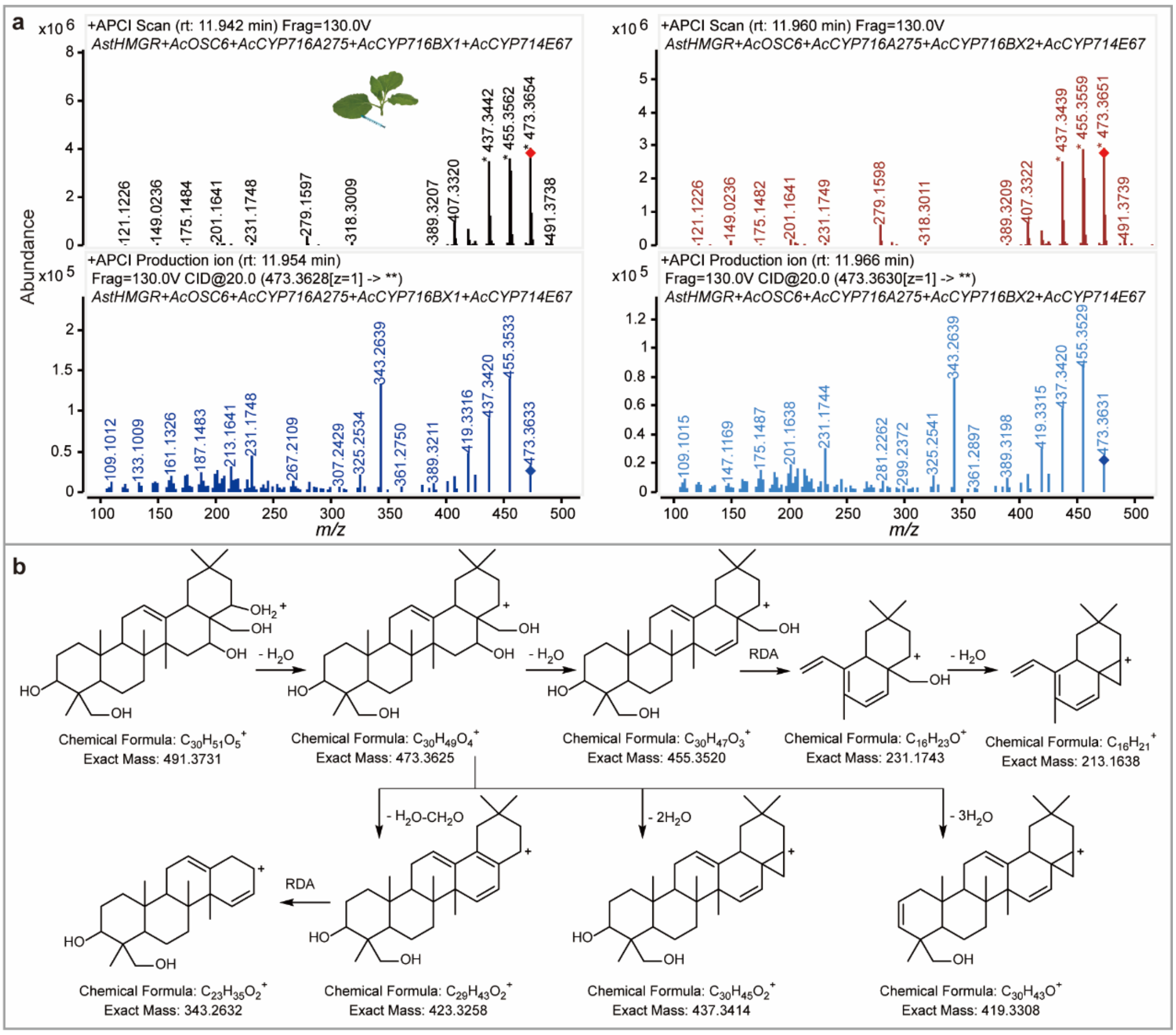

Supplementary Figure 37. Mass spectra (**a**) and proposed fragmentation pathways (**b**) of 16α,22α,24-trihydroxy-erythrodiol (**22**) produced in infiltrated *N. benthamiana*. The syringe schematic was created by Luz Eugenia Velasquez and obtained from Vecteezy (Vector Art ID: 14175787, Vecteezy Free License, https://www.vecteezy.com/vector-art/14175787-syringe-medical-drug).

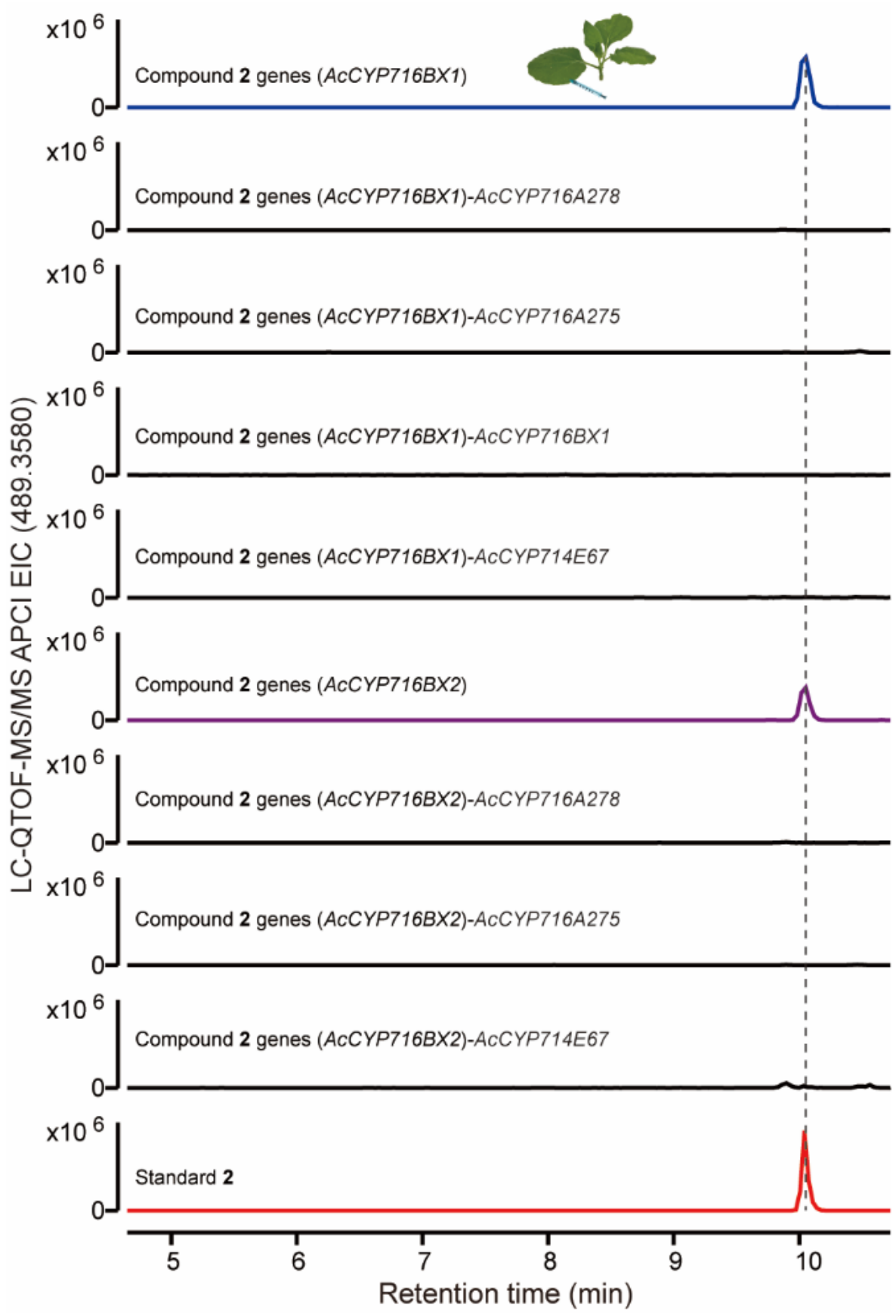

Supplementary Figure 38. Elimination of interference from endogenous *N. benthamiana* P450s via co-expression minus-one control experiments. The full biosynthetic module for **2** included *AstHMGR*, *AcOSC6*, *AcCYP716A278*, *AcCYP716BX1/2*, *AcCYP716A275*, and *AcCYP714E67*. Individual *P450* genes were systematically omitted from these modules to serve as negative controls and support that the observed hydroxylation activities were specific to the heterologously expressed target genes. The syringe schematic was created by Luz Eugenia Velasquez and obtained from Vecteezy (Vector Art ID: 14175787, Vecteezy Free License, https://www.vecteezy.com/vector-art/14175787-syringe-medical-drug).

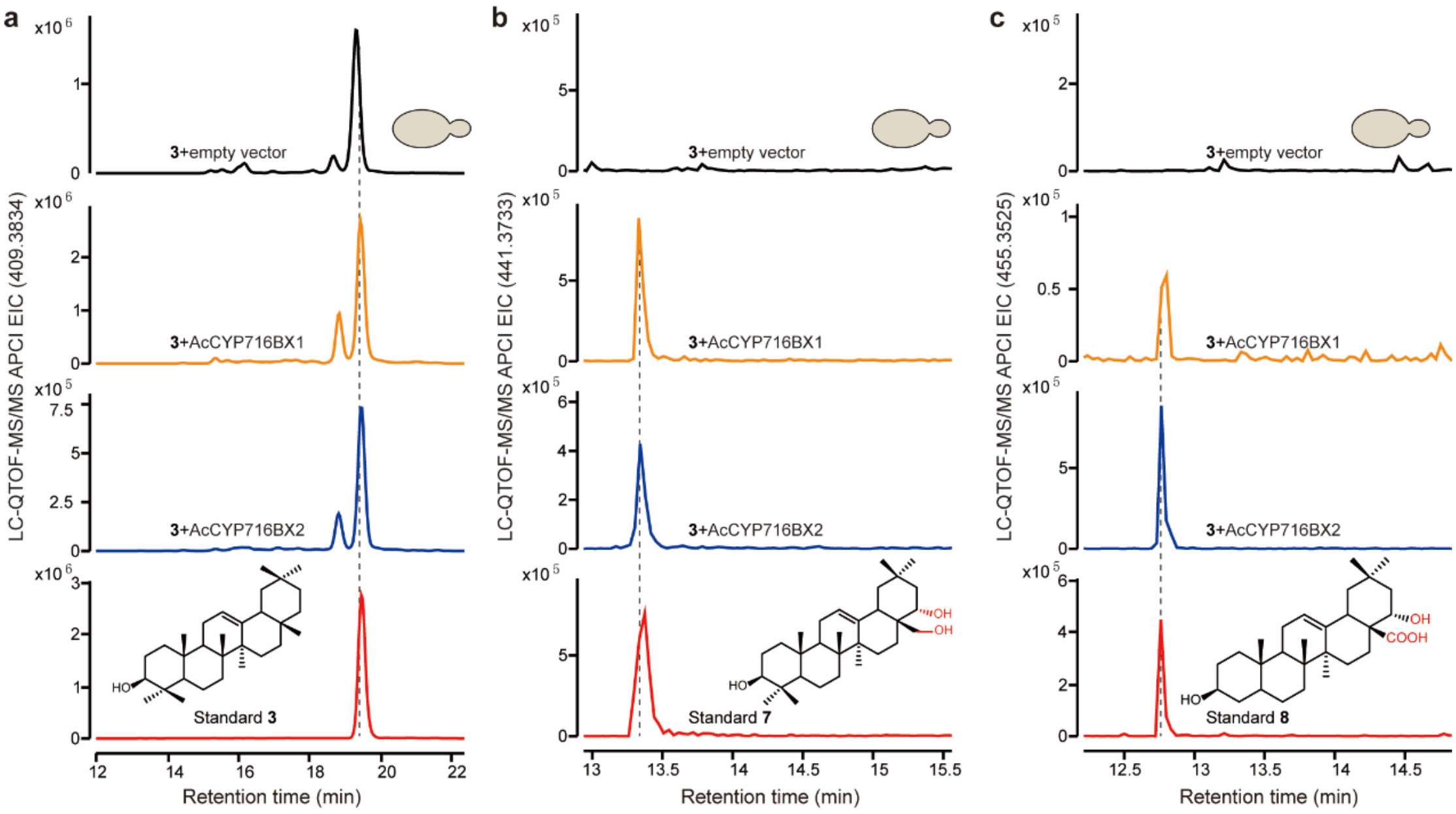

Supplementary Figure 39. Functional validation of AcCYP716BX1 and AcCYP716BX2 via yeast microsomal assays. (**a–c**) Overlay of LC-QTOF-MS/MS extracted ion chromatograms for (**a**) **3** (*m/z* 409.3834), (**b**) **7** (*m/z* 441.3733), and (**c**) **8** (*m/z* 455.3525). The data represent the catalytic conversion of substrate **3** into products **7** and **8** by the respective recombinant enzymes.

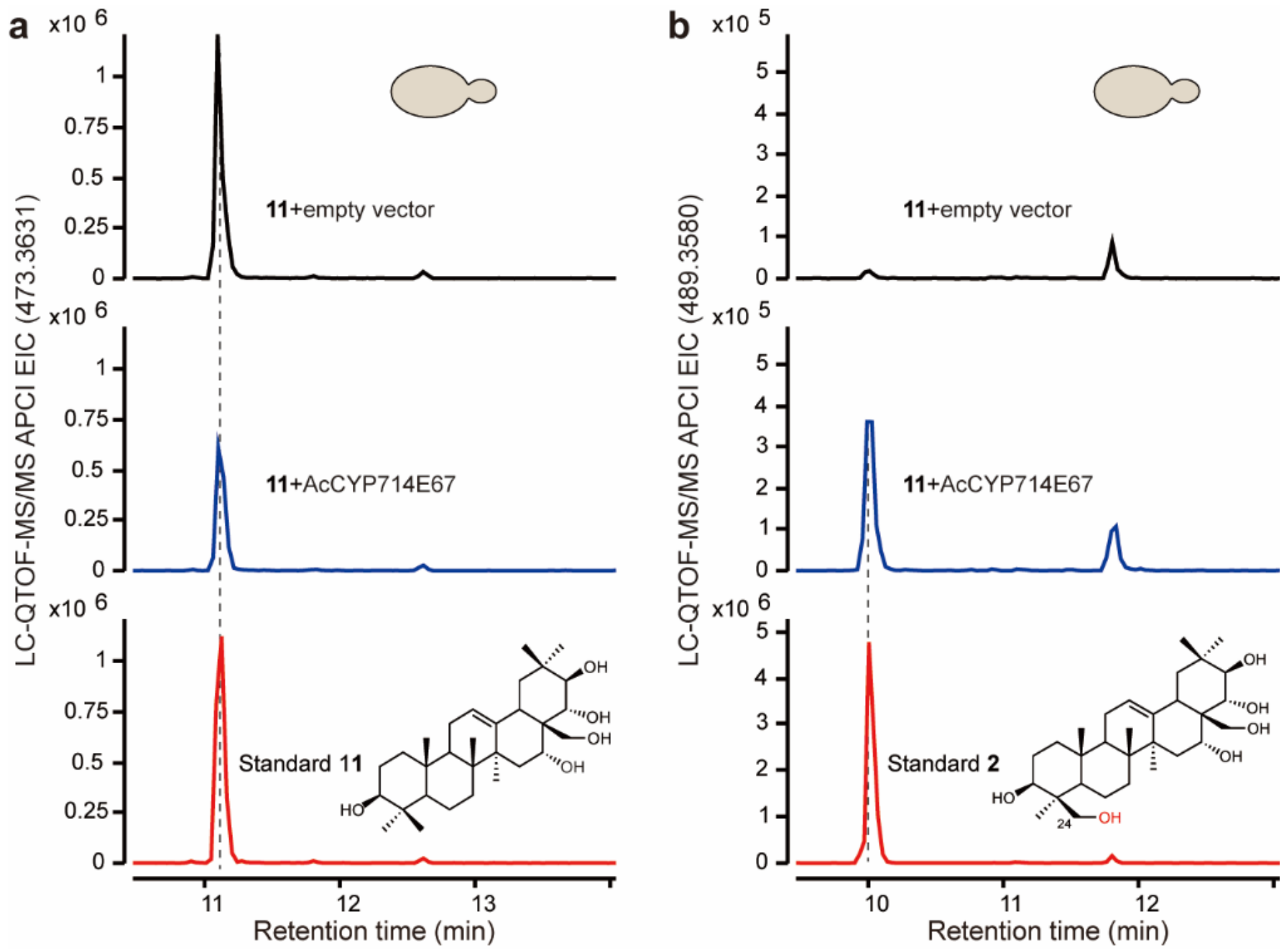

Supplementary Figure 40. Functional validation of AcCYP714E67 via yeast microsomal assays. (**a**–**b**) Overlay of LC-QTOF-MS/MS extracted ion chromatograms for (**a**) **11** (*m/z* 473.3631), and (**b**) **2** (*m/z* 489.3580).

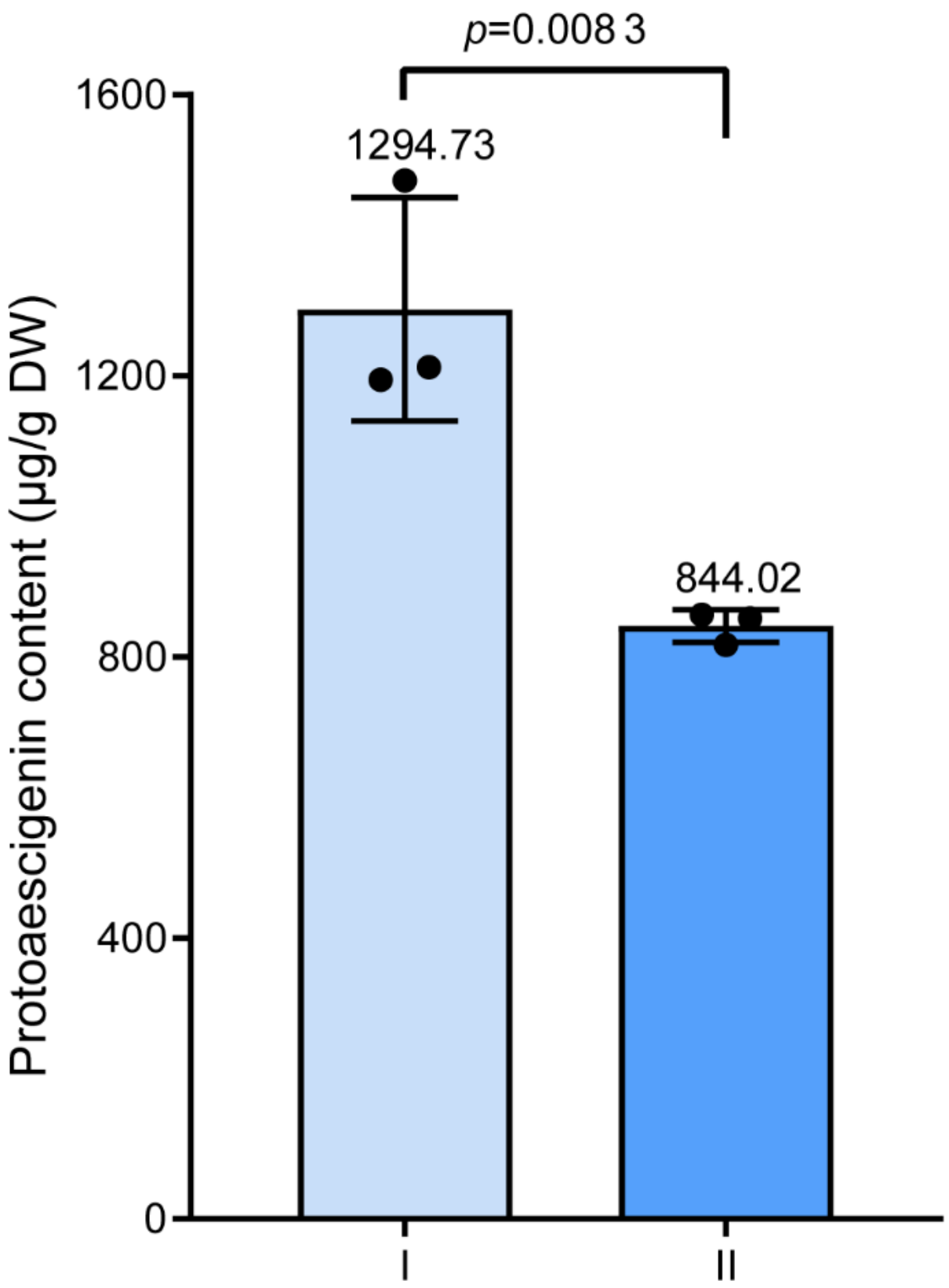

Supplementary Figure 41. Protoaescigenin (**2**) content in infiltrated *N. benthamiana*. I: Co-expression of *AstHMGR*, *AcOSC6*, *AcCYP716A278*, *AcCYP716BX1*, *AcCYP716A275*, and *AcCYP714E67*; II: Co-expression of *AstHMGR*, *AcOSC6*, *AcCYP716A278*, *AcCYP716BX2*, *AcCYP716A275*, and *AcCYP714E67*. Data are presented as mean ± *s.d.* (n = 3 independent biological replicates). Significant differences and the p-values were determined using a two-tailed Student's t-test.

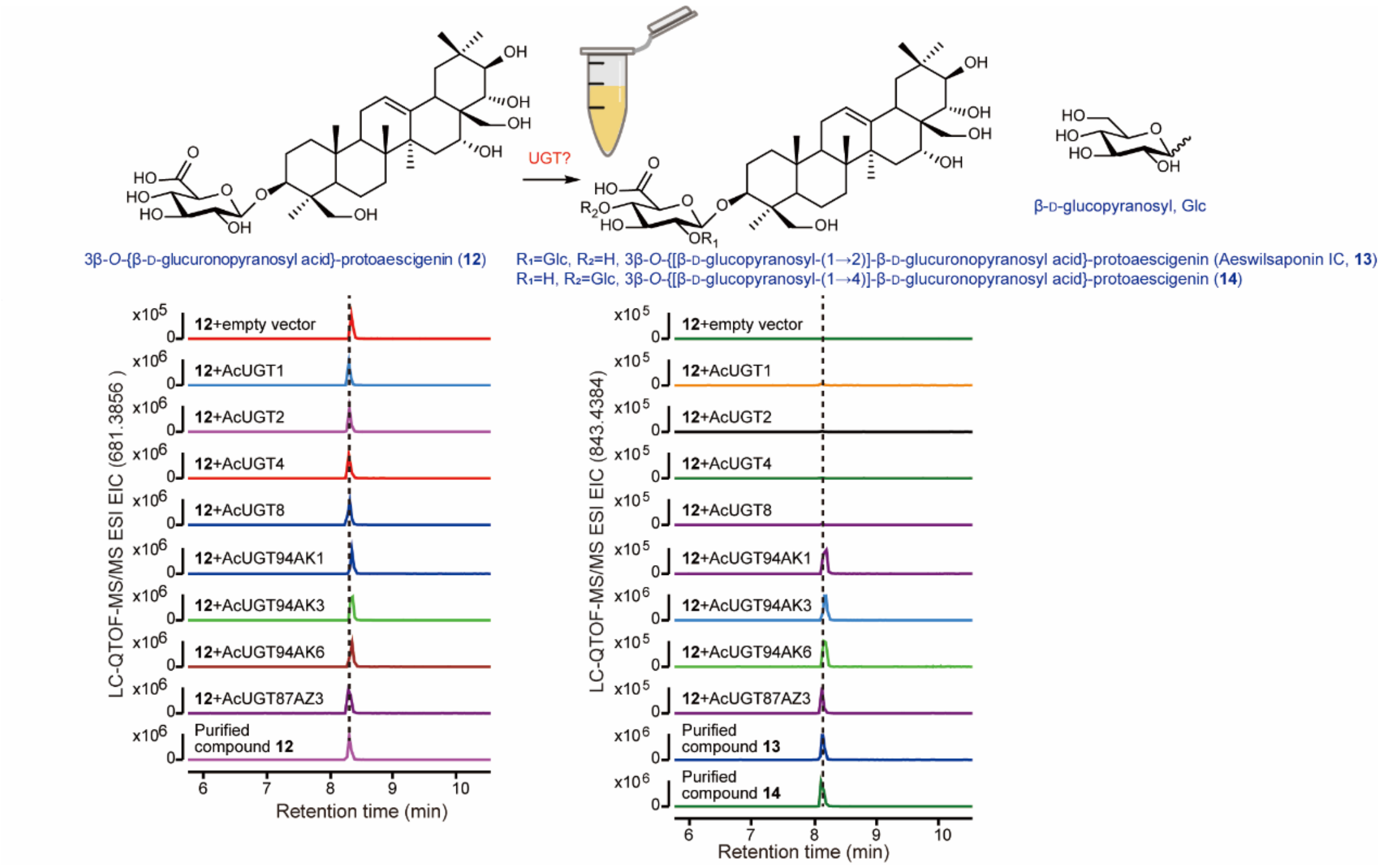

Supplementary Figure 42. *In vitro* functional analysis of candidate AcUGTs using **12** as substrate detected by the 15 min elution gradient. The glycosylated product of **12** at 2'-OH or 4'-OH position displayed the same retention time. Overlay of LC-QTOF-MS/MS extracted ion chromatograms showed that AcUGT94AK1, AcUGT94AK3, AcUGT94AK6, AcUGT87AZ3 generated a new glycosylated product, possessing the same MS^1 values and retention time with **13** and **14**. The centrifuge tube icon was created by Helicase 11 (CC-BY 4.0, https://creativecommons.org/licenses/by/4.0/).

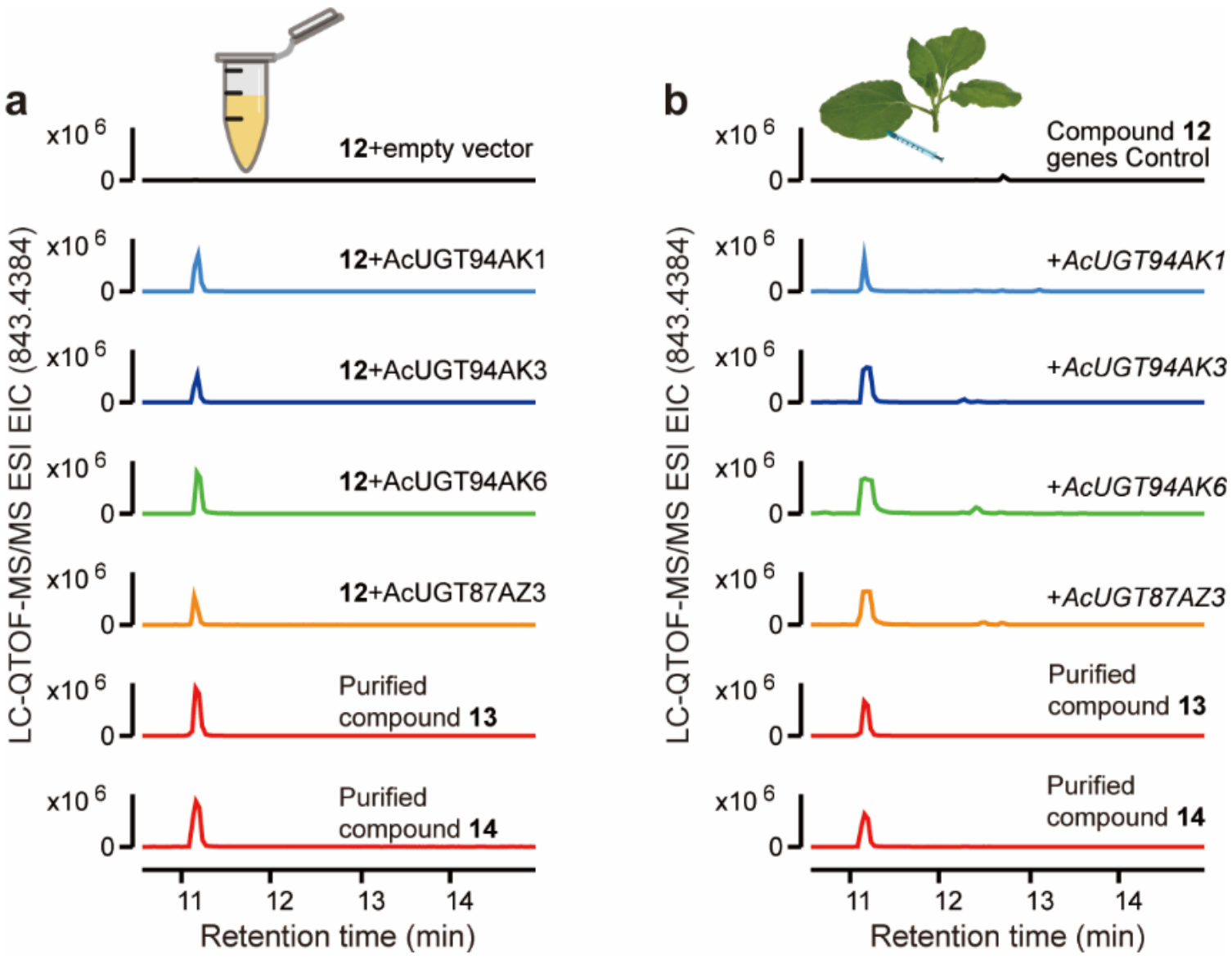

Supplementary Figure 43. Functional characterization of candidate AcUGTs using **12** as substrate detected by the 35 min elution gradient. (**a**) *In vitro* enzymatic assays of AcUGT94AK1, AcUGT94AK3, AcUGT94AK6, and AcUGT87AZ3. (**b**) *In vivo* functional analysis of the same AcUGTs in transiently expressed *N. benthamiana*. The centrifuge tube icon was created by Helicase 11 (CC-BY 4.0, https://creativecommons.org/licenses/by/4.0/); and the syringe schematic was by Luz Eugenia Velasquez via Vecteezy (Vector Art ID: 14175787, Vecteezy Free License, https://www.vecteezy.com/vector-art/14175787-syringe-medical-drug).

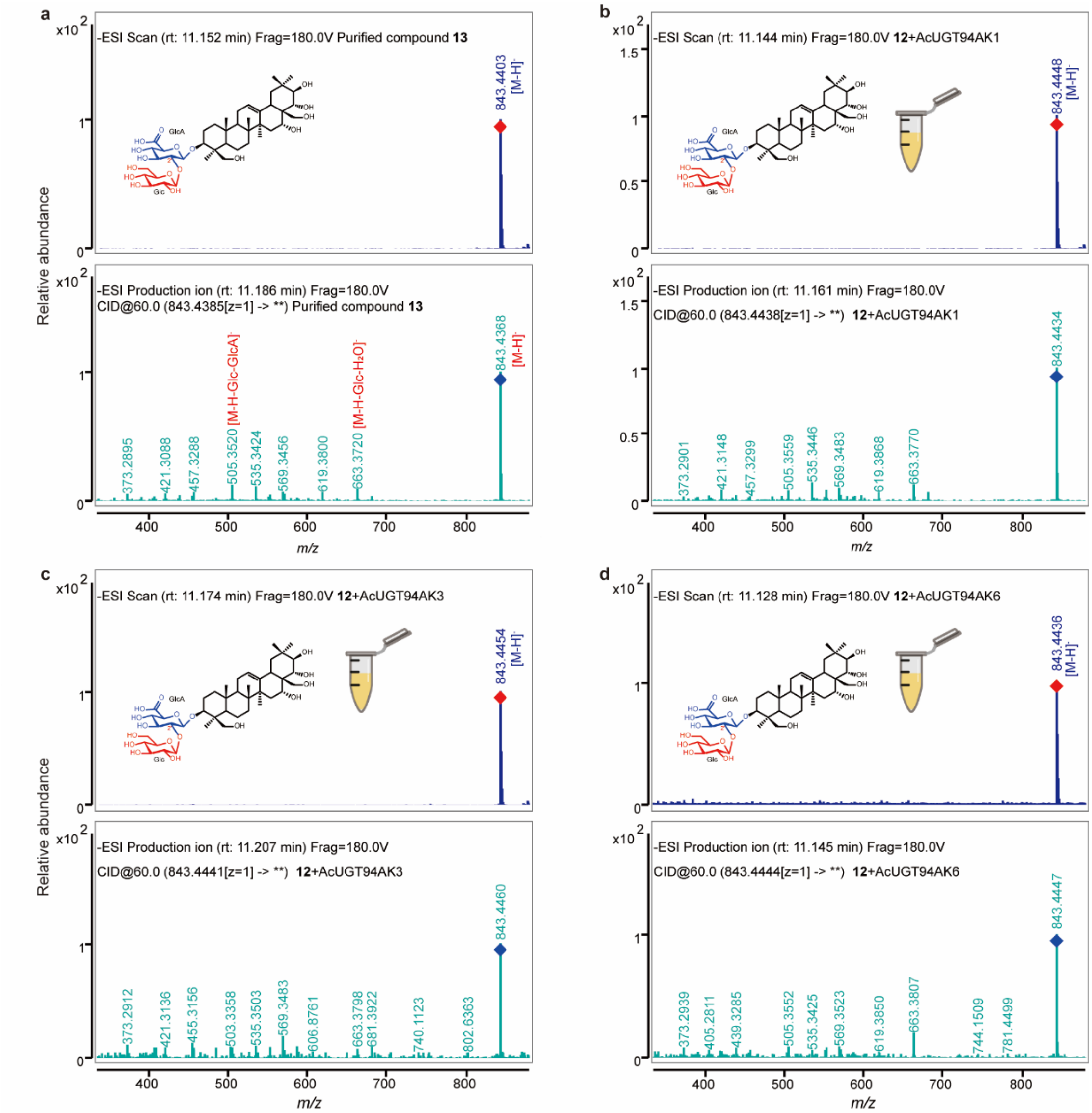

Supplementary Figure 44. The MS[1] and MS[2] spectra of 3β-*O*-{[β-D-glucopyranosyl-(1→2)]-β-D-glucuronopyranosyl acid}-protoaescigenin (Aeswilsaponin IC, **13**) generated *in vitro*. (**a**) The purified compound **13**. **(b**, **c**, **d**) The corresponding compound generated *in vitro* by AcUGT94AK1, AcUGT94AK3 and AcUGT94AK6 using **12** as substrate. The centrifuge tube icon was created by Helicase 11 (CC-BY 4.0, https://creativecommons.org/licenses/by/4.0/).

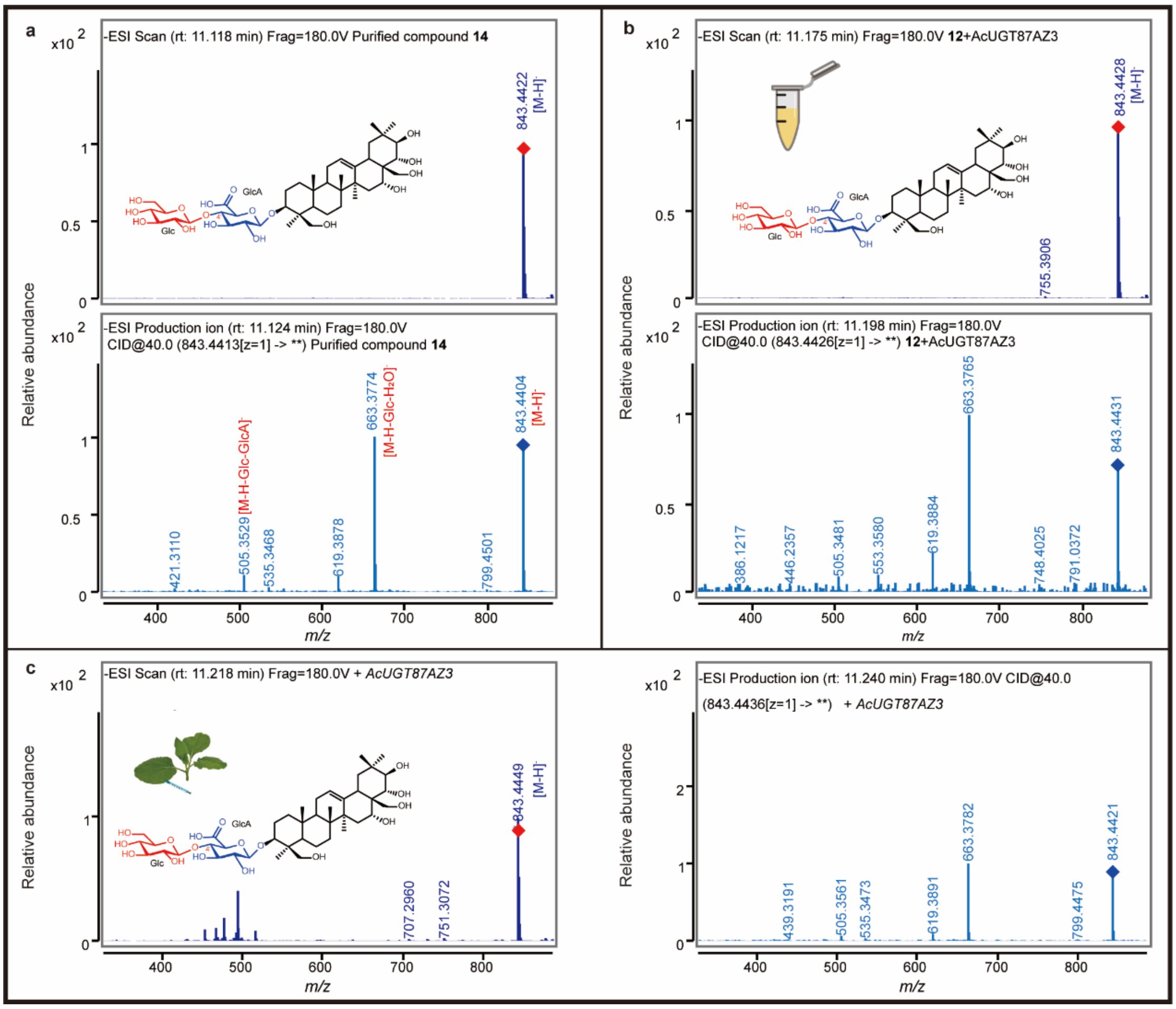

Supplementary Figure 45. The MS[1] and MS[2] spectra of 3β-*O*-{[β-D-glucopyranosyl-(1→4)]-β-D-glucuronopyranosyl acid}-protoaescigenin (**14**). (**a**) The purified compound **14**. (**b**) The corresponding compound generated *in vitro* by AcUGT87AZ3 using **12** as substrate. (**c**) The corresponding compound yielded in *N. benthamiana* co-expressing seven genes involved in **12** biosynthesis (*AstHMGR, AcOSC6, AcCYP716A278, AcCYP716BX1, AcCYP716A275, AcCYP714E67, AcCSL1*) with *AcUGT87AZ3*. The centrifuge tube icon was created by Helicase 11 (CC-BY 4.0, https://creativecommons.org/licenses/by/4.0/); and the syringe schematic was by Luz Eugenia Velasquez via Vecteezy (Vector Art ID: 14175787, Vecteezy Free License, https://www.vecteezy.com/vector-art/14175787-syringe-medical-drug).

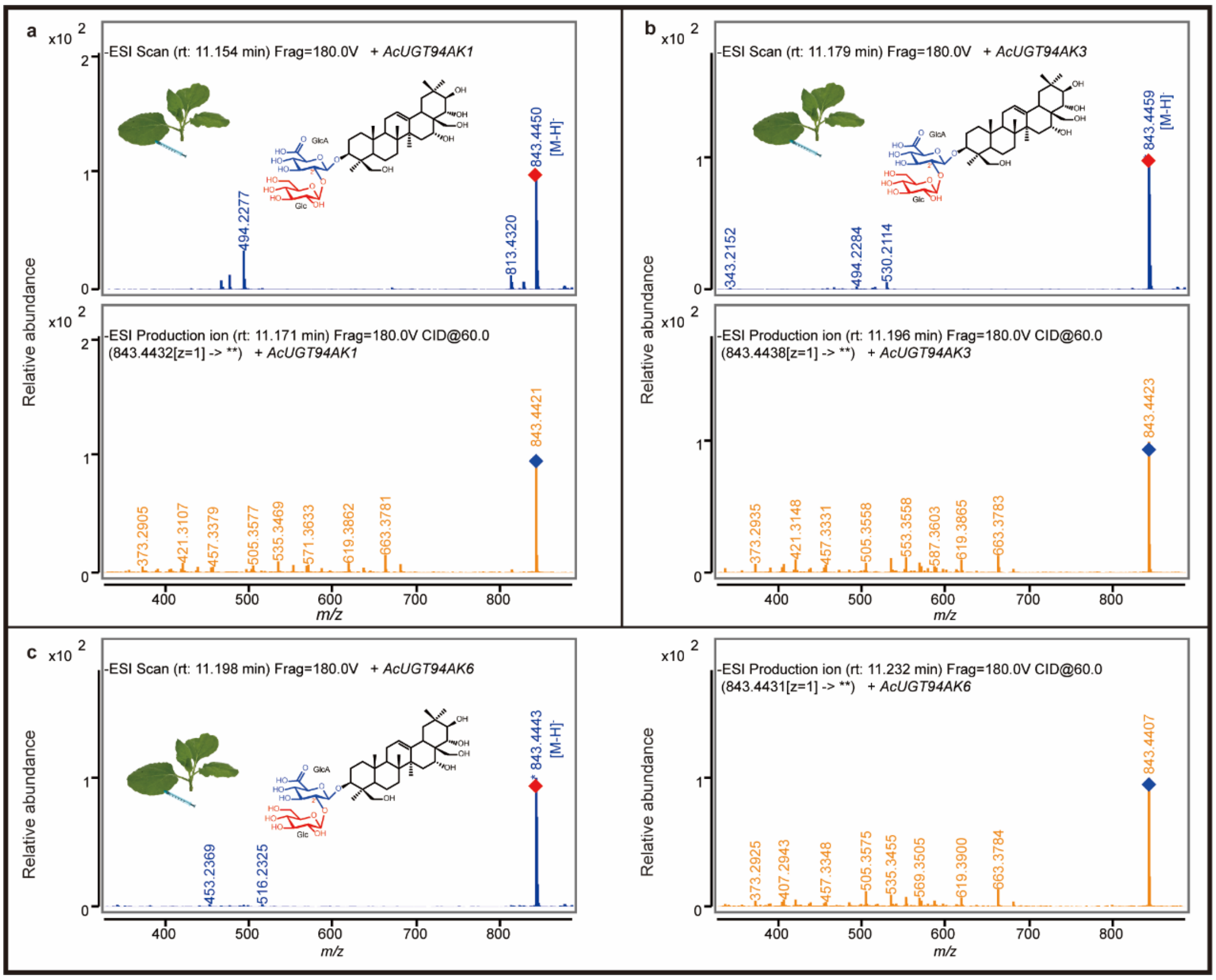

Supplementary Figure 46. The MS^1 and MS^2 spectra of **13** generated in infiltrated *N. benthamiana* co-expressing seven genes involved in **12** biosynthesis (*AstHMGR, AcOSC6, AcCYP716A278, AcCYP716BX1, AcCYP716A275, AcCYP714E67, AcCSL1*) with *UGT94AK1* (**a**), *UGT94AK3* (**b**), and *UGT94AK6* (**c**), respectively. The syringe schematic was created by Luz Eugenia Velasquez and obtained from Vecteezy (Vector Art ID: 14175787, Vecteezy Free License, https://www.vecteezy.com/vector-art/14175787-syringe-medical-drug).

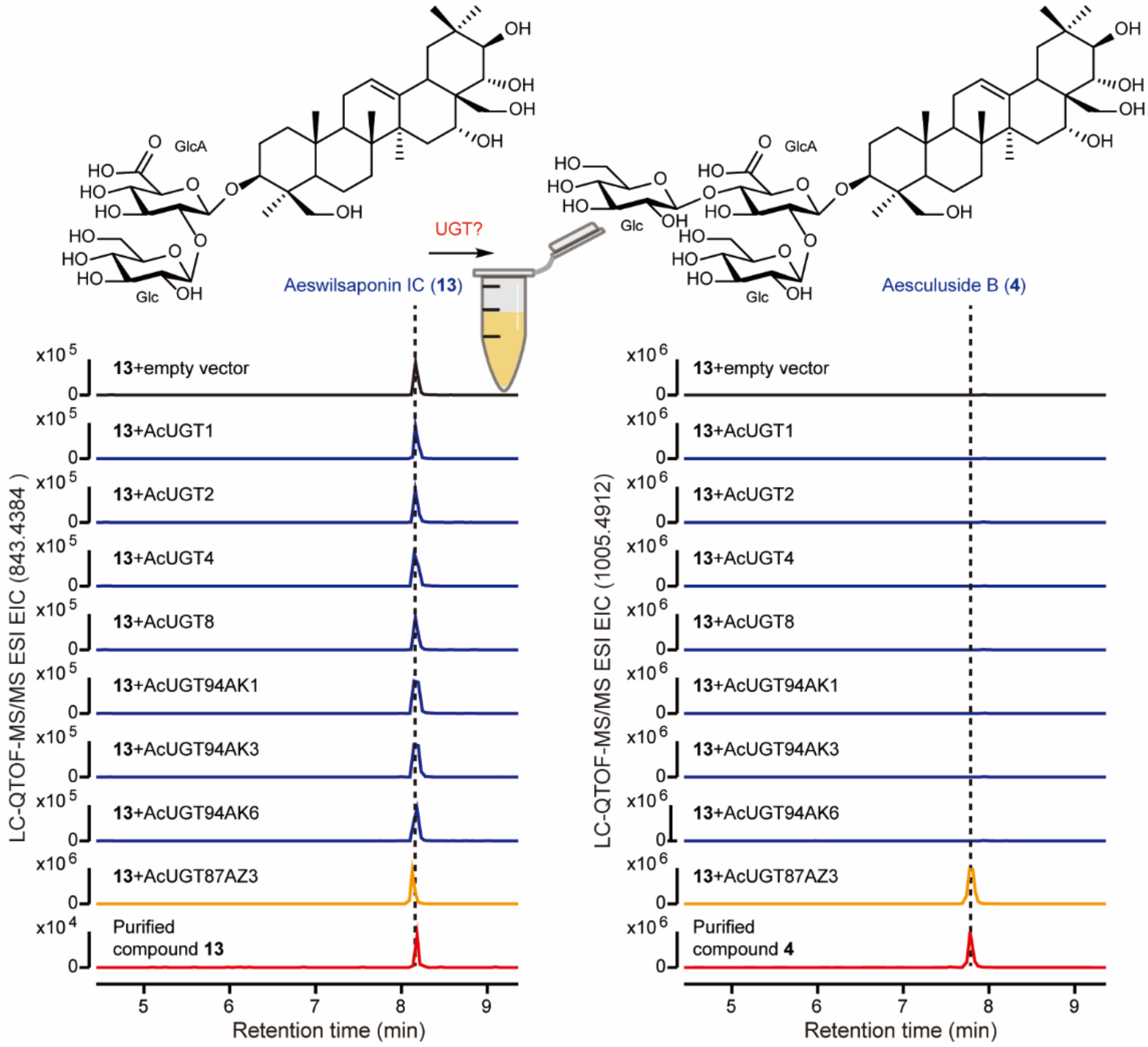

Supplementary Figure 47. *In vitro* functional analysis of candidate AcUGTs using **13** as substrate detected by the 15 min elution gradient. Overlay of LC-QTOF-MS/MS extracted ion chromatograms showed that only AcUGT87AZ3 generated compound **4**. The centrifuge tube icon was created by Helicase 11 (CC-BY 4.0, https://creativecommons.org/licenses/by/4.0/).

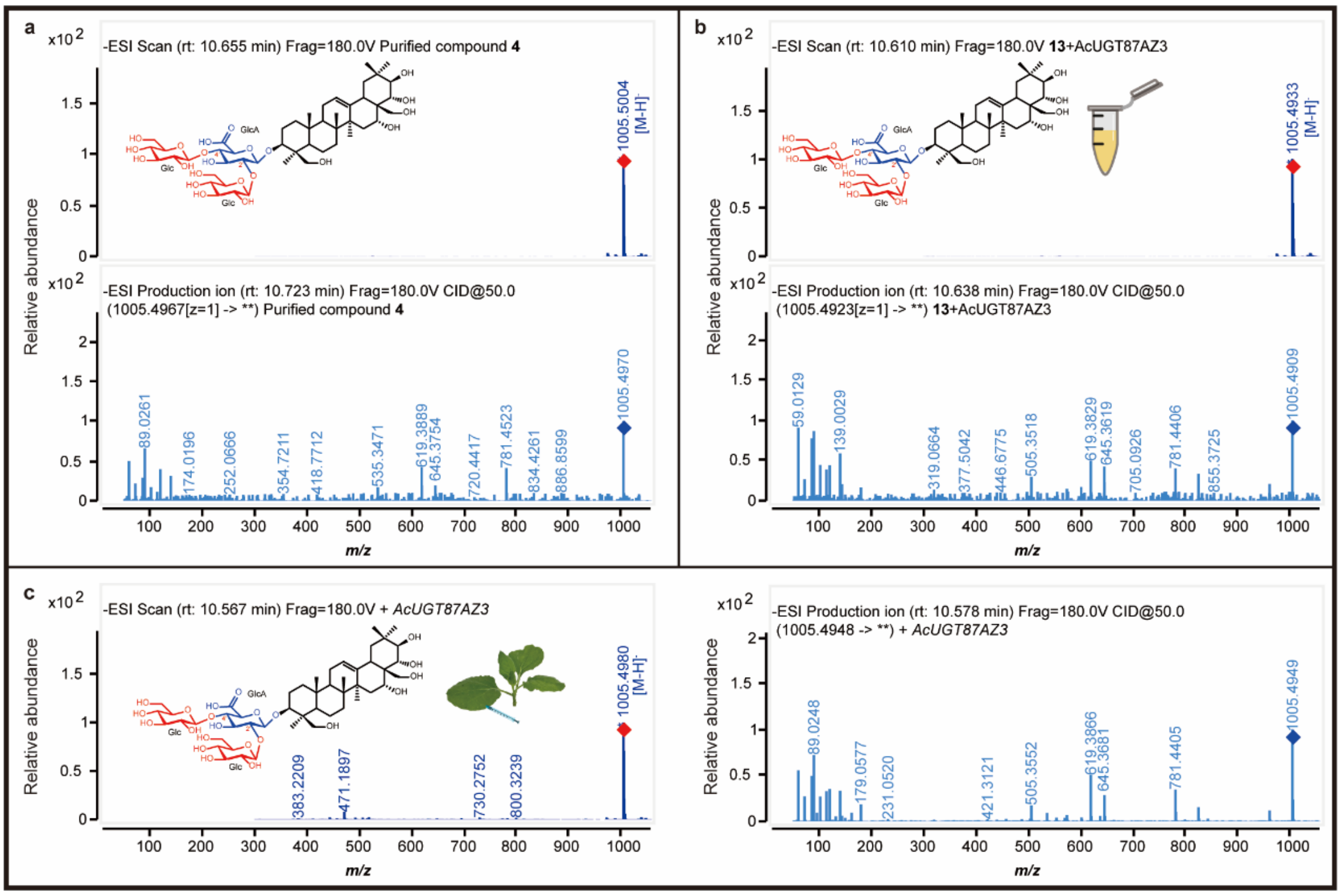

Supplementary Figure 48. The MS[1] and MS[2] spectra of 3β-*O*-{β-D-glucopyranosyl-(1→4)-[β-D-glucopyranosyl-(1→2)]-β-D-glucuronopyranosyl acid}-protoaescigenin (Aesculuside B, **4**). (**a**) The purified compound **4**. (**b**) The corresponding compound generated *in vitro* by AcUGT87AZ3 using **13** as substrate. (**c**) The corresponding product yielded in infiltrated *N. benthamiana* co-expressing eight genes involved in **13** biosynthesis (*AstHMGR, AcOSC6, AcCYP716A278, AcCYP716BX1, AcCYP716A275, AcCYP714E67, AcCSL1, AcUGT94AK1*) with *AcUGT87AZ3*. The centrifuge tube icon was created by Helicase 11 (CC-BY 4.0, https://creativecommons.org/licenses/by/4.0/); and the syringe schematic was by Luz Eugenia Velasquez via Vecteezy (Vector Art ID: 14175787, Vecteezy Free License, https://www.vecteezy.com/vector-art/14175787-syringe-medical-drug).

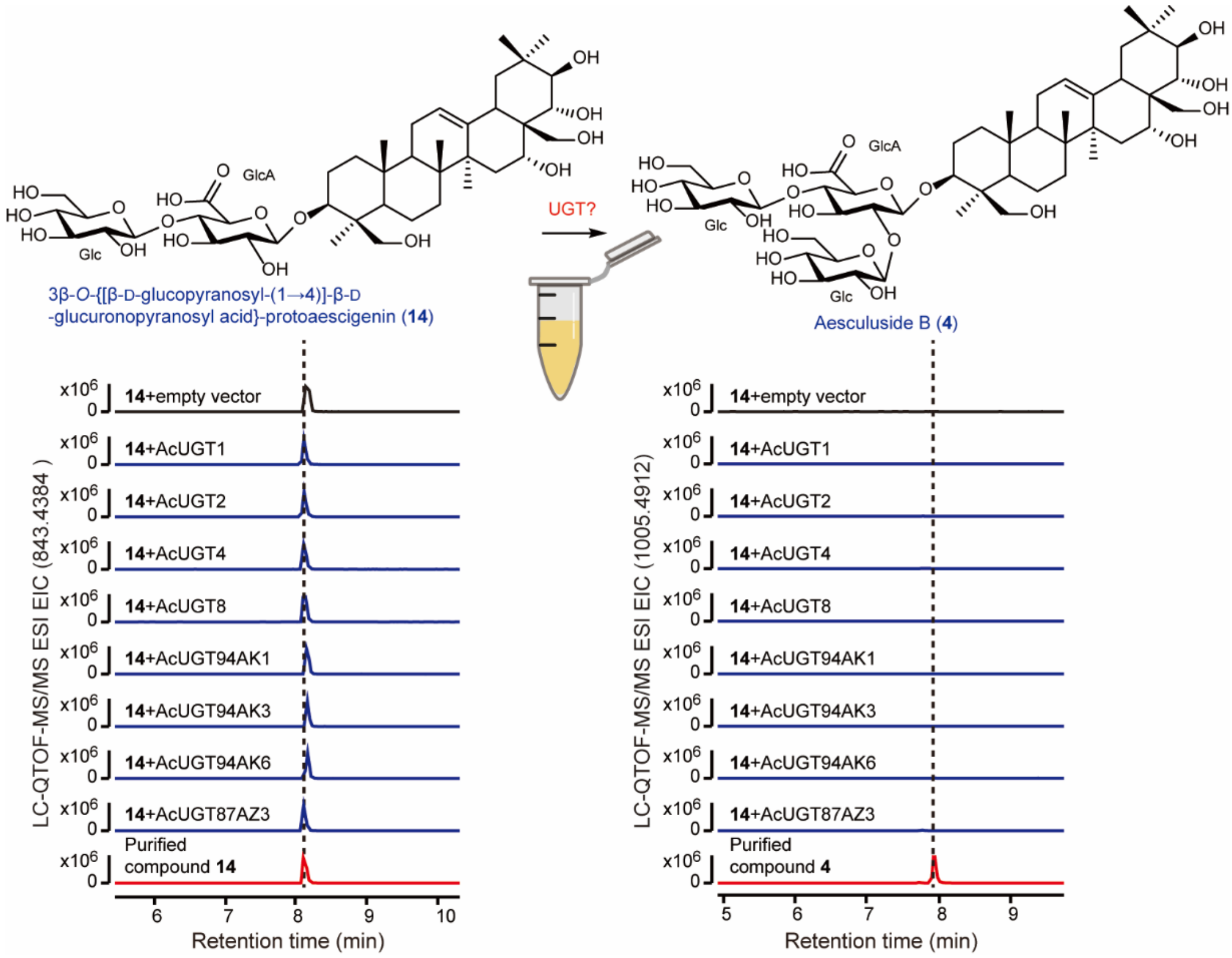

Supplementary Figure 49. *In vitro* functional analysis of candidate AcUGTs using **14** as substrate detected by the 15 min elution gradient. Overlay of LC-QTOF-MS/MS extracted ion chromatograms show that none of 8 tested AcUGTs can generate any new glycosylated products. The centrifuge tube icon was created by Helicase 11 (CC-BY 4.0, https://creativecommons.org/licenses/by/4.0/).

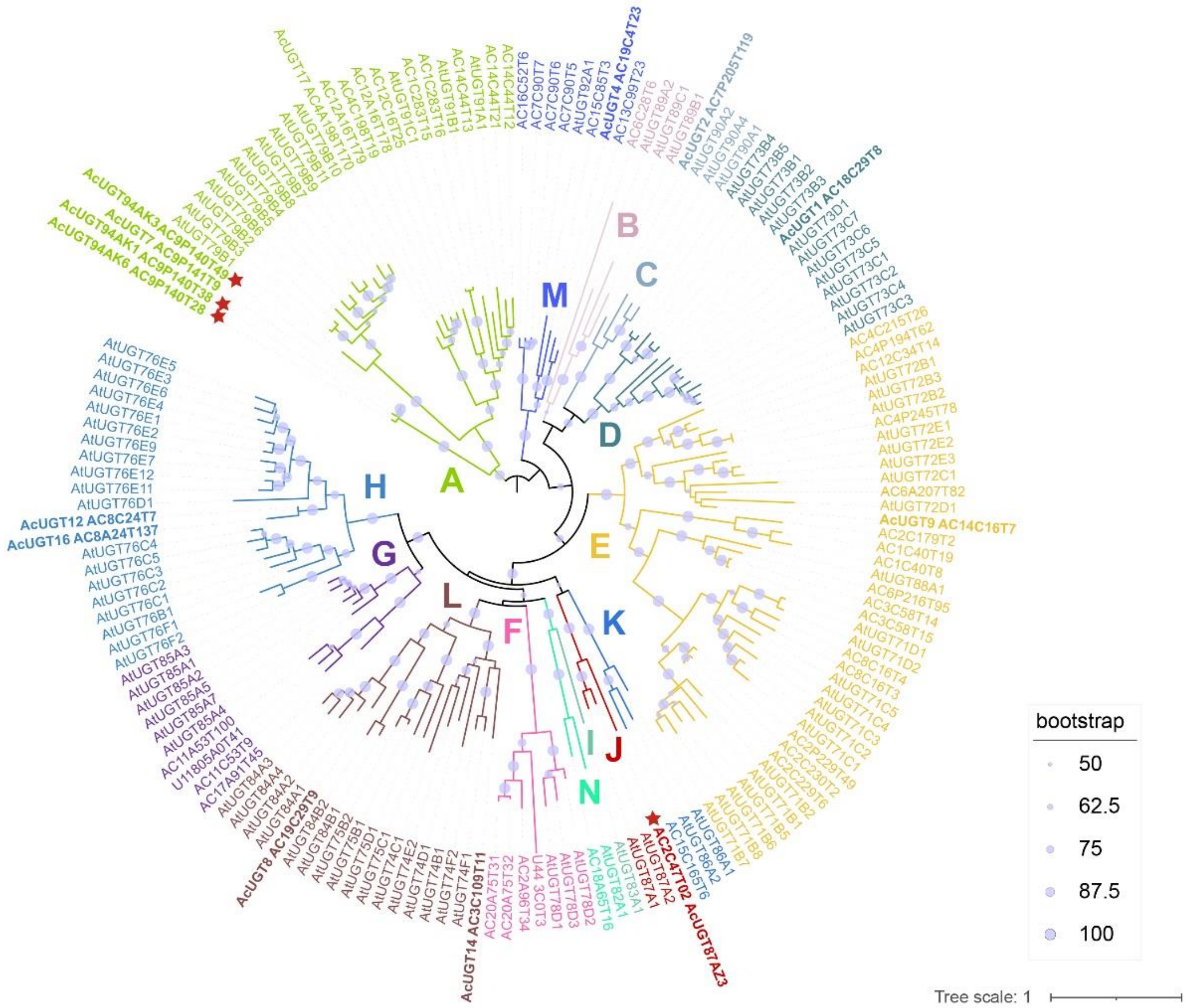

Supplementary Figure 50. Phylogenetic analysis of the AcUGT family. A total of 58 AcUGTs were mined through Pfam annotation (PF00201). The maximum-likelihood tree was constructed with IQ-TREE under 1000 bootstrap replicates. As mentioned in the main text, AcUGTs (highlighted in bold and marked with a star) represent the functional gene responsible for escin Ia biosynthesis. Fourteen clades are labelled according to the classification[1]. Distinct UGT clades are designated by letters. The scale bar corresponds to phylogenetic distance.

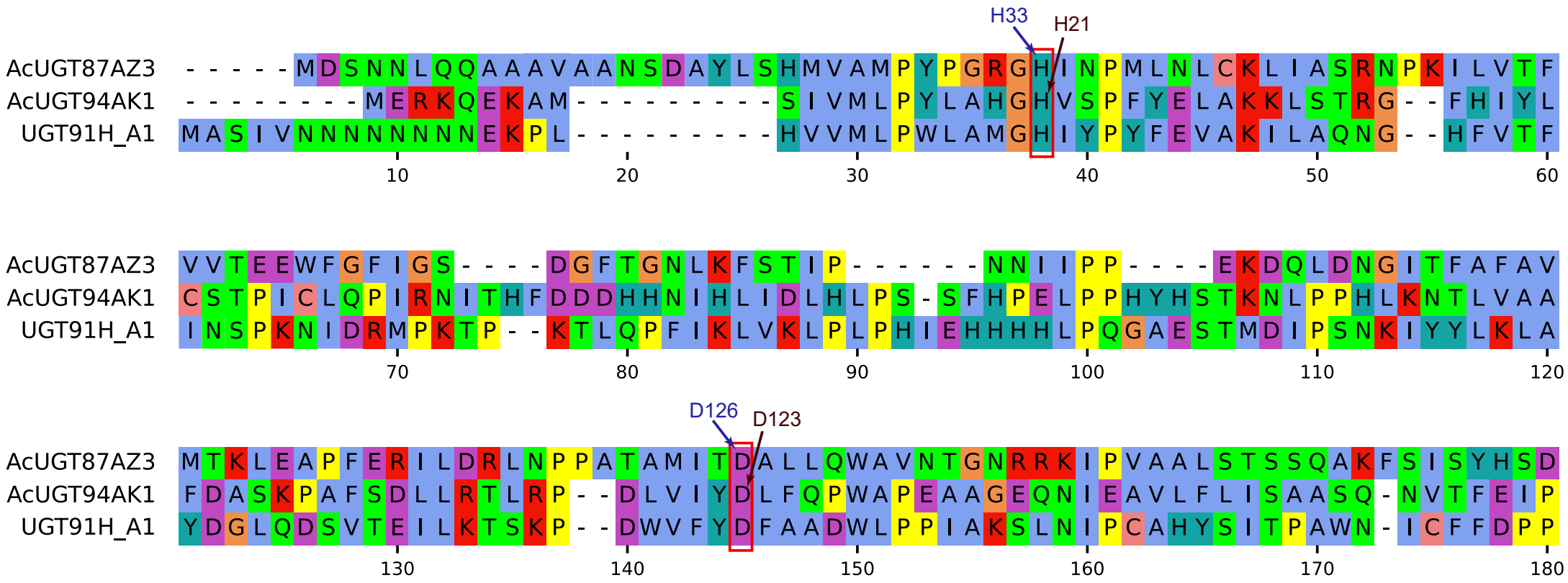

Supplementary Figure 51. Sequence alignment among AcUGT94AK1, AcUGT87AZ3 and UGT91H_A1. UGT91H_A1 was previously reported by Jian et al. (2024)[2]. Key residues of the core UGT HD dyad are highlighted by red rectangles.

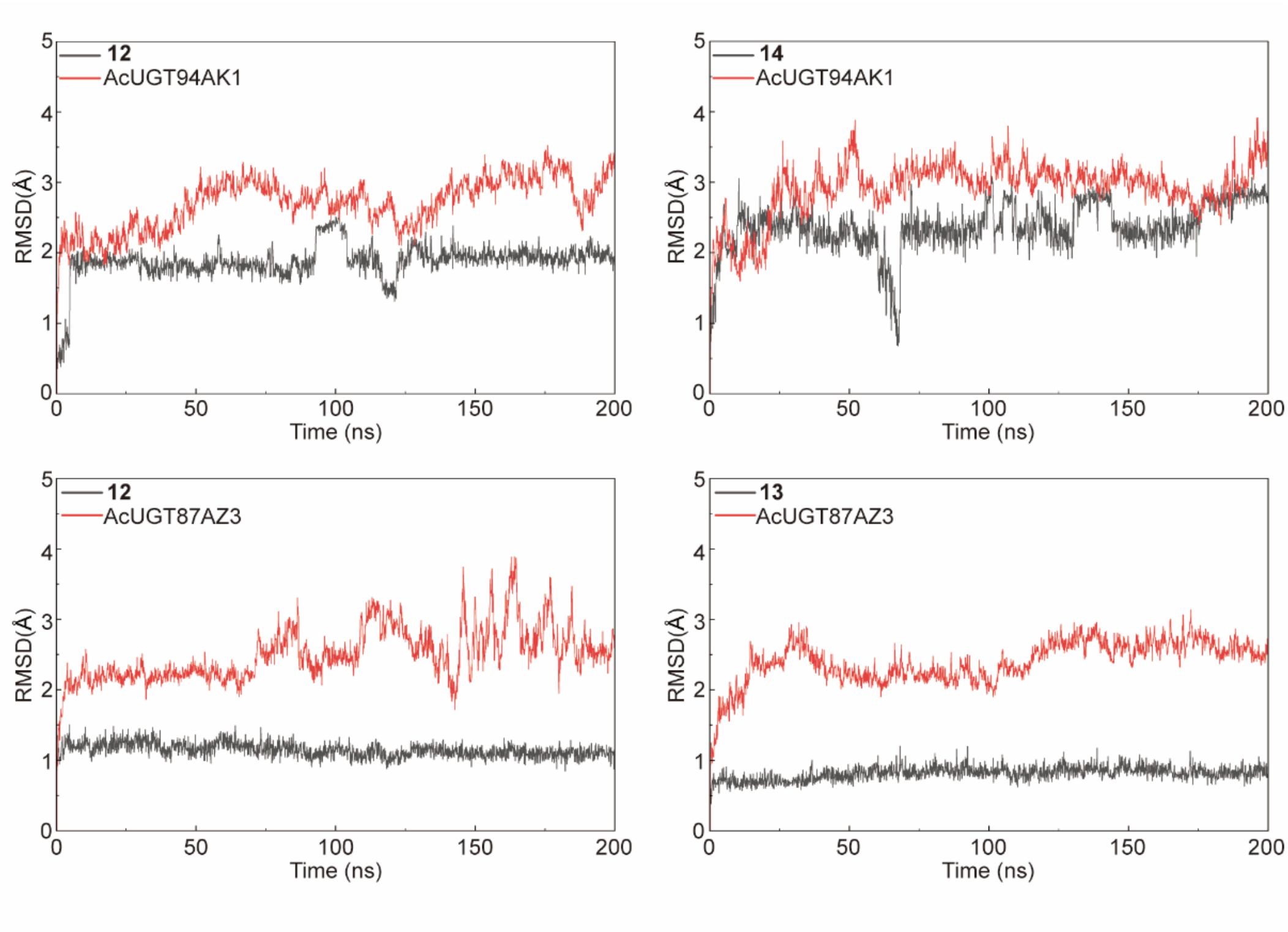

Supplementary Figure 52. Root-mean-square deviation (RMSD) profiles of AcUGT94AK1 and AcUGT87AZ3 in the complexes with various ligands during 200 ns MD simulations.

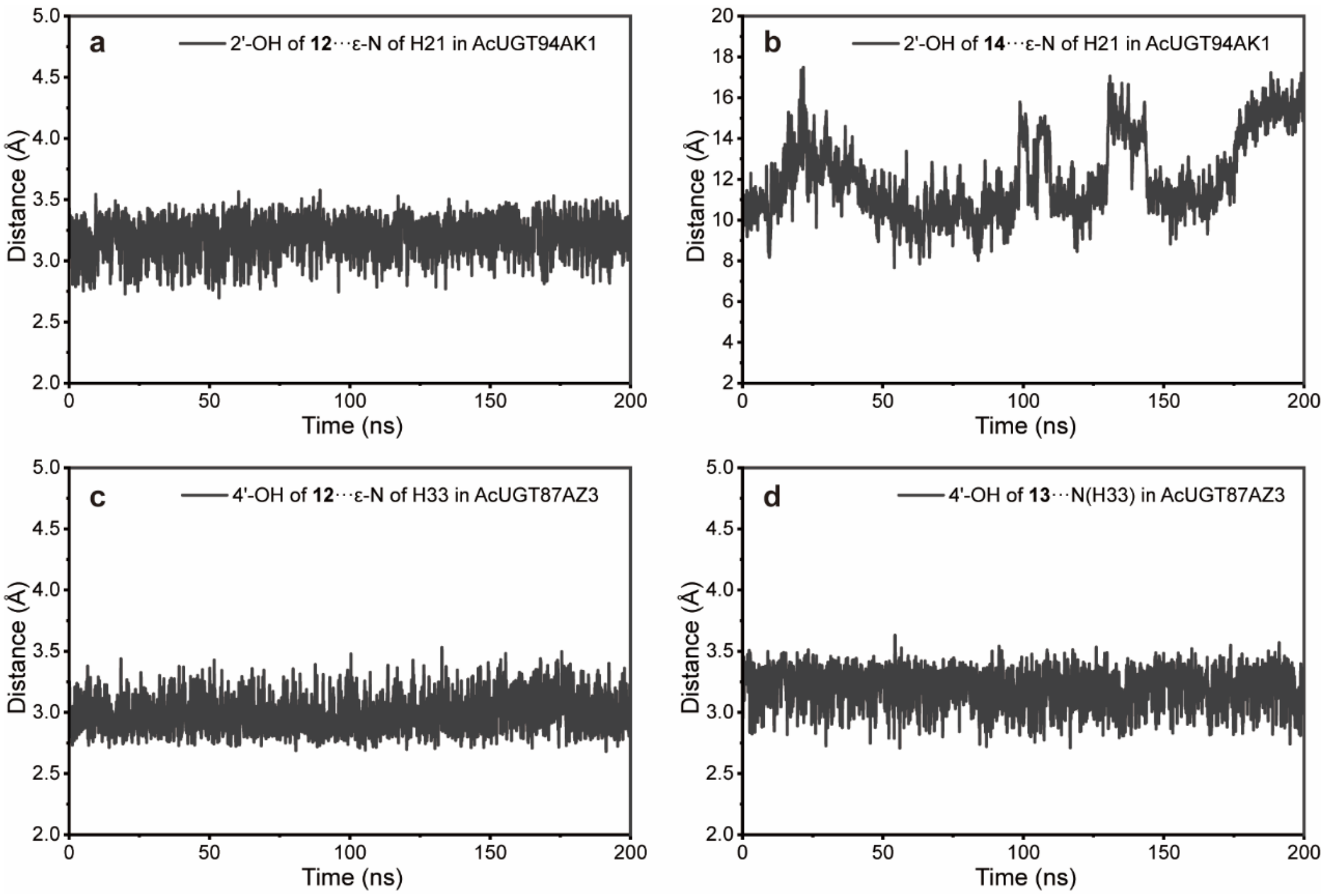

Supplementary Figure 53. The measured O–N distance between the oxygen atom of the acceptor molecule and the ε-nitrogen of histidine in the complexes of AcUGT94AK1-**12** (**a**), AcUGT94AK1-**14** (**b**), AcUGT87AZ3-**12** (**c**), and AcUGT87AZ3-**13** (**d**), respectively.

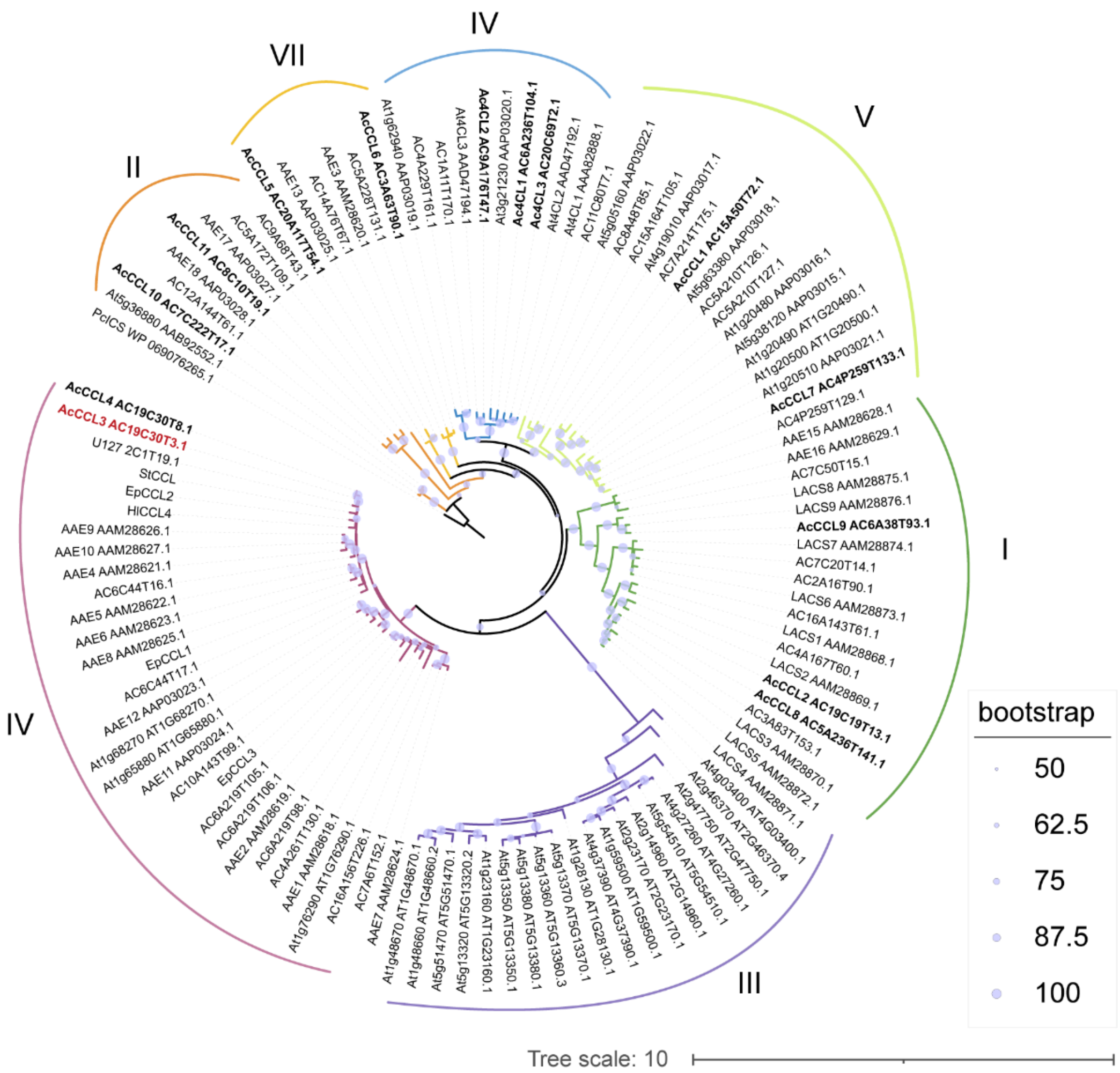

Supplementary Figure 54. Phylogenetic analysis of the AcCCL family. A total of 45 AcCCLs were mined through Pfam annotation (PF00501). The tree was constructed using the maximum likelihood method. The AcCCL genes were aligned with 63 CCL genes from previous reports[3-5], and isobutyryl CoA synthetase gene (PcICS, GenBank ID: WP_069076265.1) from *Pseudomonas chlororaphis*[6] was used as an outgroup. Seven clades are labelled according to the classification of Martin et al. (2024)[3]. AcCCL3 (highlighted in red bold) represent the functional gene responsible for escin Ia biosynthesis.

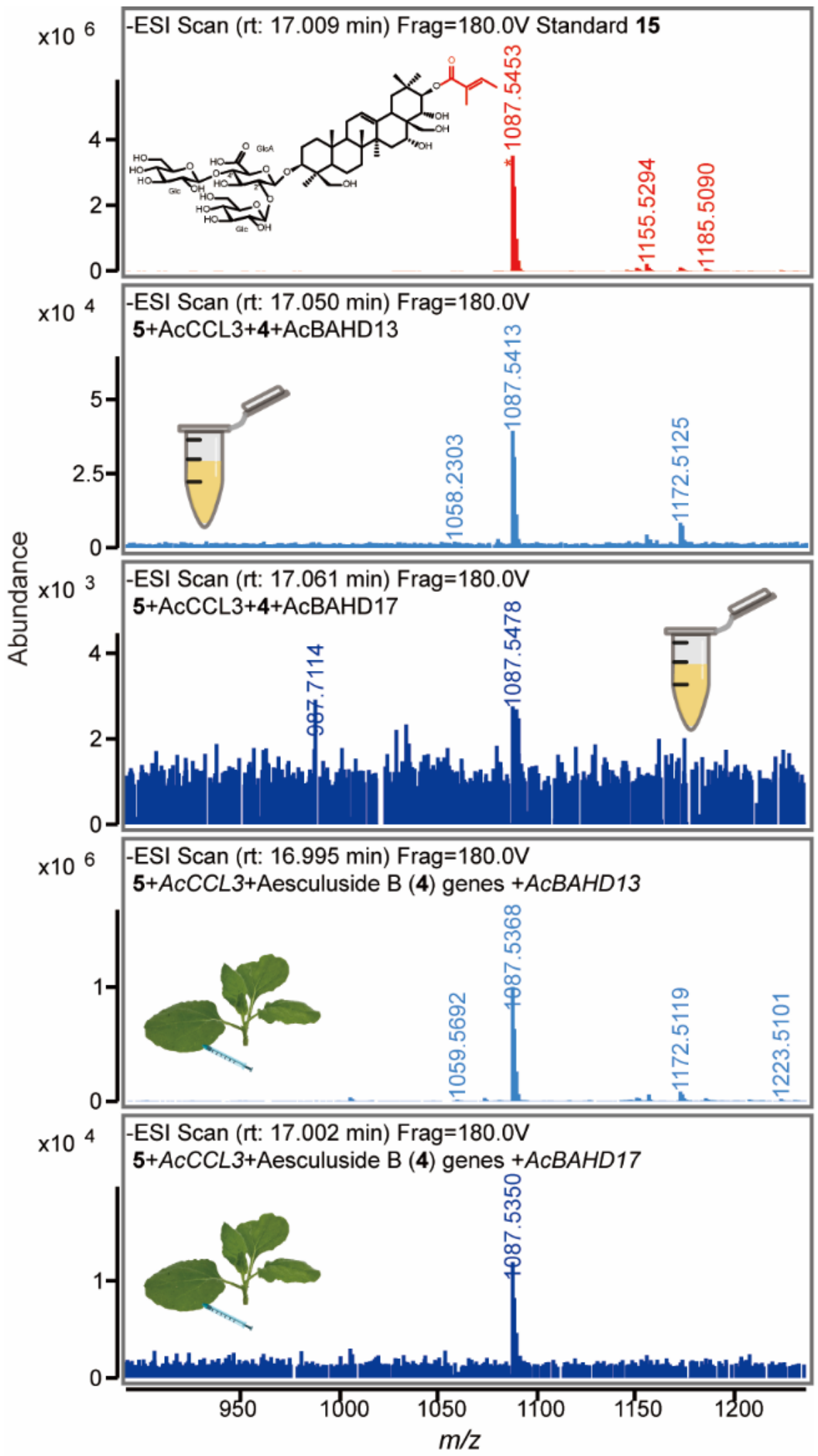

Supplementary Figure 55. The mass spectra of desacetylescin Ia (**15**): the standard **15**; the corresponding product generated *in vitro* by AcBAHD13 or AcBAHD17 with AcCCL3 using **4** as substrate; the corresponding product generated in infiltrated *N. benthamiana* co-expressing *AcBAHD13* or *AcBAHD17* with *AcCCL3* and nine genes involved in **4** biosynthesis (*AstHMGR, AcOSC6, AcCYP716A278, AcCYP716BX1, AcCYP716A275, AcCYP714E67, AcCSL1, AcUGT94AK1,* and *AcUGT87AZ3*). The centrifuge tube icon was created by Helicase 11 (CC-BY 4.0, https://creativecommons.org/licenses/by/4.0/); and the syringe schematic was by Luz Eugenia Velasquez via Vecteezy (Vector Art ID: 14175787, Vecteezy Free License, https://www.vecteezy.com/vector-art/14175787-syringe-medical-drug).

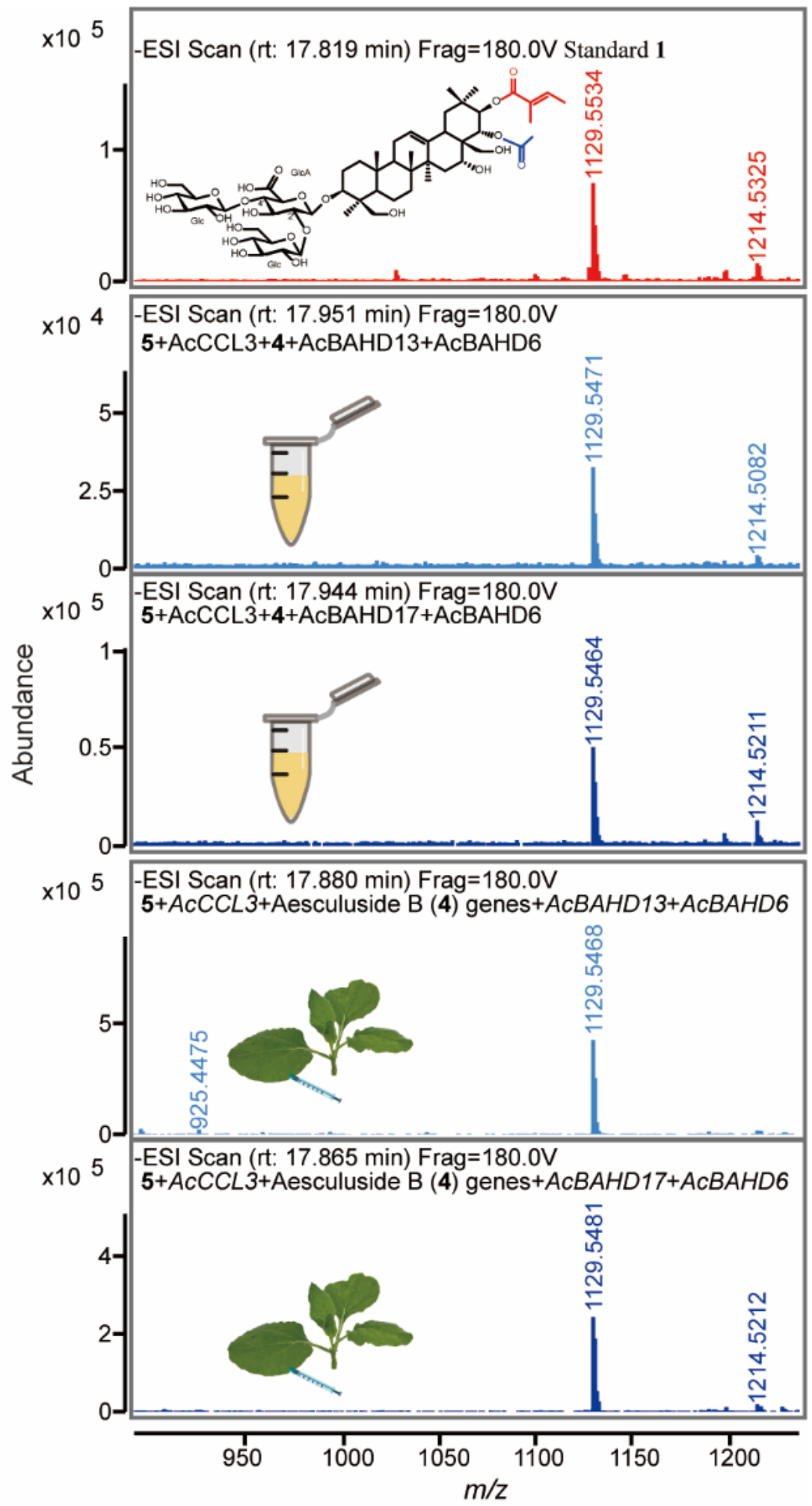

Supplementary Figure 56. The mass spectra of escin Ia (**1**): the standard **1**, the corresponding product generated *in vitro* by AcBAHD13 or AcBAHD17 along with AcCCL3, AcBAHD6, **4** and **5**, and the corresponding product generated in infiltrated *N. benthamiana* co-expressing *AcBAHD13* or *AcBAHD17* with **5**, *AcCCL3, AcBAHD6*, and nine genes involved in **4** biosynthesis (*AstHMGR, AcOSC6, AcCYP716A278, AcCYP716BX1, AcCYP716A275, AcCYP714E67, AcCSL1, AcUGT94AK1,* and *AcUGT87AZ3*). The centrifuge tube icon was created by Helicase 11 (CC-BY 4.0, https://creativecommons.org/licenses/by/4.0/); and the syringe schematic was by Luz Eugenia Velasquez via Vecteezy (Vector Art ID: 14175787, Vecteezy Free License, https://www.vecteezy.com/vector-art/14175787-syringe-medical-drug).

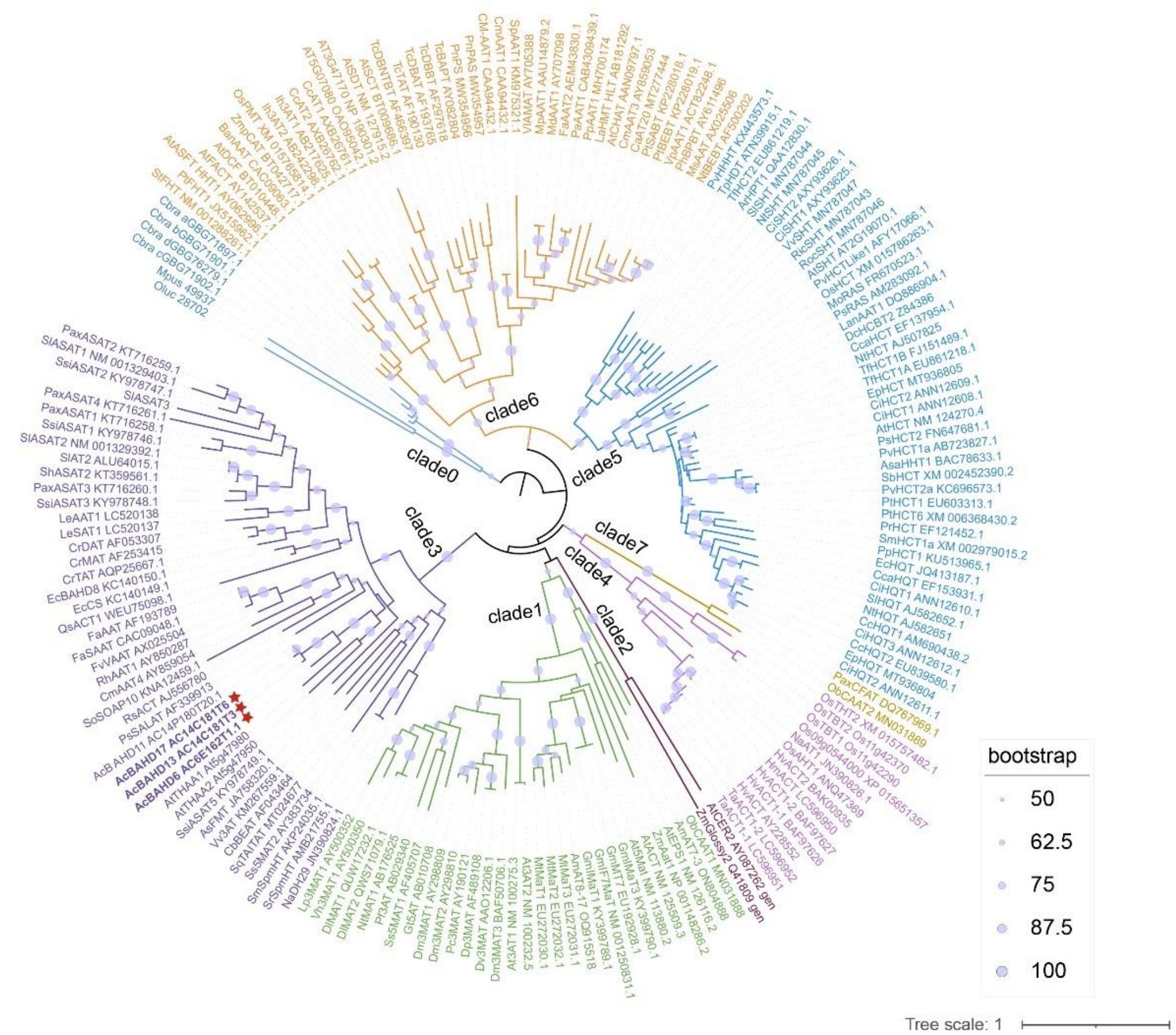

Supplementary Figure 57. Phylogenetic analysis of the AcBAHD6, AcBAHD11, AcBAHD13, and AcBAHD17 with known BAHDs. The tree was constructed using the maximum likelihood method. AcBAHDs (AcBAHD6, AcBAHD13, and AcBAHD17) involved in escin Ia biosynthesis are highlighted in red stars. Other BAHD sequences included in the phylogenetic analysis were obtained from the study by Moghe et al. (2023)[7]. Distinct clades are labeled with letters.

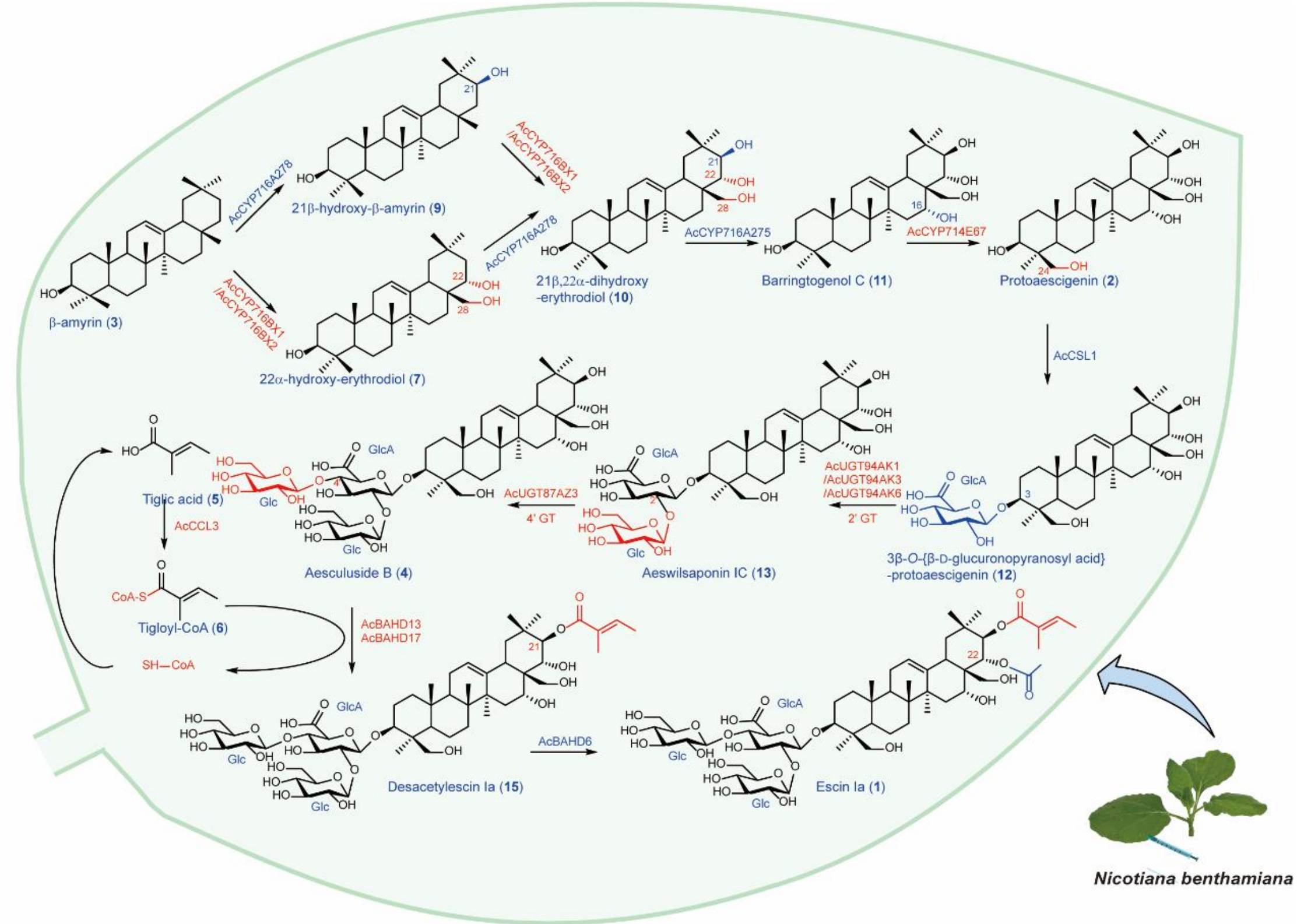

Supplementary Figure 58. Elucidation and reconstruction of escin Ia biosynthesis in *N. benthamiana*. Escin Ia production was successfully achieved in the infiltrated *N. benthamiana* leaves co-expressing 12 candidate biosynthetic genes (*AstHMGR, AcOSC6, AcCYP716A278, AcCYP716BX1/2, AcCYP716A275, AcCYP714E67, AcCSL1, AcUGT94AK1, AcUGT87AZ3, AcCCL3, AcBAHD13/AcBAHD17, and AcBAHD6*). The syringe schematic was created by Luz Eugenia Velasquez and obtained from Vecteezy (Vector Art ID: 14175787, Vecteezy Free License, https://www.vecteezy.com/vector-art/14175787-syringe-medical-drug).

a

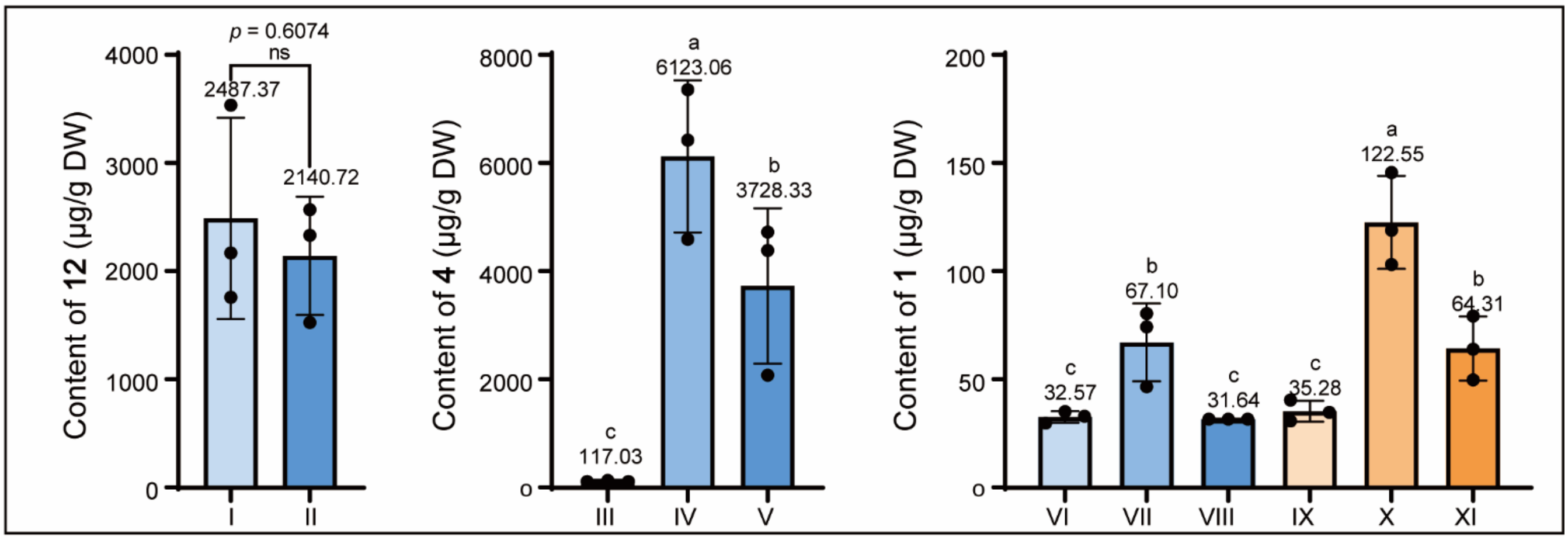

b

Name	Specific genes
I	*AstHMGR*, *AcOSC6*, *AcCYP716A278*, *AcCYP716BX1*, *AcCYP716A275*, *AcCYP714E67*, *AcCSL1*
II	*AstHMGR*, *AcOSC6*, *AcCYP716A278*, *AcCYP716BX2*, *AcCYP716A275*, *AcCYP714E67*, *AcCSL1*
III	*AstHMGR*, *AcOSC6*, *AcCYP716A278*, *AcCYP716BX1*, *AcCYP716A275*, *AcCYP714E67*, *AcCSL1*, *AcUGT94AK1*, *AcUGT87AZ3*
IV	*AstHMGR*, *AcOSC6*, *AcCYP716A278*, *AcCYP716BX1*, *AcCYP716A275*, *AcCYP714E67*, *AcCSL1*, *AcUGT94AK3*, *AcUGT87AZ4*
V	*AstHMGR*, *AcOSC6*, *AcCYP716A278*, *AcCYP716BX1*, *AcCYP716A275*, *AcCYP714E67*, *AcCSL1*, *AcUGT94AK6*, *AcUGT87AZ5*
VI	*AstHMGR*, *AcOSC6*, *AcCYP716A278*, *AcCYP716BX1*, *AcCYP716A275*, *AcCYP714E67*, *AcCSL1*, *AcUGT94AK1*, *AcUGT87AZ3*, *AcCCL3*, *AcBAHD6*, *AcBAHD13*
VII	*AstHMGR*, *AcOSC6*, *AcCYP716A278*, *AcCYP716BX1*, *AcCYP716A275*, *AcCYP714E67*, *AcCSL1*, *AcUGT94AK3*, *AcUGT87AZ3*, *AcCCL3*, *AcBAHD6*, *AcBAHD13*
VIII	*AstHMGR*, *AcOSC6*, *AcCYP716A278*, *AcCYP716BX1*, *AcCYP716A275*, *AcCYP714E67*, *AcCSL1*, *AcUGT94AK6*, *AcUGT87AZ3*, *AcCCL3*, *AcBAHD6*, *AcBAHD13*
IX	*AstHMGR*, *AcOSC6*, *AcCYP716A278*, *AcCYP716BX1*, *AcCYP716A275*, *AcCYP714E67*, *AcCSL1*, *AcUGT94AK1*, *AcUGT87AZ3*, *AcCCL3*, *AcBAHD6*, *AcBAHD17*
X	*AstHMGR*, *AcOSC6*, *AcCYP716A278*, *AcCYP716BX1*, *AcCYP716A275*, *AcCYP714E67*, *AcCSL1*, *AcUGT94AK3*, *AcUGT87AZ3*, *AcCCL3*, *AcBAHD6*, *AcBAHD17*
XI	*AstHMGR*, *AcOSC6*, *AcCYP716A278*, *AcCYP716BX1*, *AcCYP716A275*, *AcCYP714E67*, *AcCSL1*, *AcUGT94AK6*, *AcUGT87AZ3*, *AcCCL3*, *AcBAHD6*, *AcBAHD17*

Supplementary Figure 59. Quantification of **12**, **4**, and **1** in infiltrated *N. benthamiana* leaves. (**a**) Content of **12** in groups I and II, **4** in groups III–V, **1** in groups VI–XI. Data are presented as *mean* ± *s.d.* (*n* = 3 independent biological replicates). For **12**, Significant differences and the *p*-values were determined using a two-tailed Student's *t*-test. For **4** and **1**, different lowercase letters represent significant differences ($p < 0.05$) among group means, as determined by one-way analysis of variance (ANOVA) followed by Duncan's multiple range test. (**b**) Specific gene combinations transiently expressed in groups I–XI.

Supplementary Table 1. The expression patterns of candidate *AcP450s*, *AcUGTs* and *AcCCLs*, which were identified in the 'turquoise' module of WGCNA

Gene name	Flower	Pericarp	Seed	Leaf	Branch	Chr	Length (bp)
***AcCYP716BX2**	**81.29**	**391.95**	**5327.85**	**3.42**	**150.87**	**chr8**	**1470**
***AcCYP716A278**	**36.15**	**281.04**	**4997.01**	**0.85**	**95.08**	**chr15**	**1422**
***AcCYP716A275**	**31.75**	**257.9**	**3725.86**	**0.64**	**116.31**	**chr8**	**1356**
***AcCYP714E67**	**2.2**	**4.15**	**3229.12**	**0.12**	**11.24**	**chr13**	**1557**
***AcCYP716BX1**	**3.78**	**16.68**	**1677.3**	**0**	**0.3**	**chr15**	**1461**
AcCYP82D185	**4.91**	**36.54**	**528.24**	**74.52**	**3.63**	**chr4**	**1590**
AcCYP75B134	**164.54**	**231.56**	**519.85**	**222.3**	**40.34**	**chr5**	**1041**
AcCYP701A79	**14.16**	**3.38**	**96.72**	**8.38**	**13.86**	**chr4**	**1602**
AcCYP85A67	**0**	**0.33**	**88.29**	**0**	**0**	**chr1**	**1404**
AcCYP710A134	**9.23**	**6.43**	**51.36**	**0.61**	**0.14**	**chr17**	**1509**
AcCYP85A82	**18.5**	**7.9**	**50.32**	**0.95**	**0.15**	**chr2**	**1506**
AcCYP724B37	**0.22**	**1.33**	**42.15**	**0**	**0.05**	**chr12**	**1497**
AcCYP735A74	**0.67**	**0.08**	**33.72**	**0**	**0.48**	**chr10**	**1524**
***AcUGT94AK3**	**13.21**	**107.16**	**3030.09**	**2.31**	**3.48**	**chr9**	**1341**
AcUGT7	2.79	24.25	1129.65	0.63	1.06	chr9	**879**
***AcUGT94AK1**	**2.97**	**51.14**	**975.06**	**0.44**	**110.29**	**chr9**	**1344**
AcUGT8	**210**	**69.13**	**836.86**	**60.56**	**0.16**	**chr19**	**1419**
AcUGT14	1.94	12.98	451.64	0	4.78	chr3	1380
***AcUGT87AZ3**	**0.62**	**0.04**	**291.58**	**0**	**0**	**chr2**	**1380**
AcUGT4	**0.1**	**0.28**	**204.52**	**0.1**	**0.63**	**chr19**	**1473**
AcUGT15	0.05	0.13	124.21	0	0.06	chr8	1359
AcUGT16	4.16	2.73	111.81	0	0.07	chr8	1392
AcUGT1	**26.86**	**25.84**	**74.22**	**18.21**	**30.7**	**chr18**	**1518**
AcUGT9	2.74	9.93	73.74	0	0	chr14	1455
AcUGT17	0.67	1.3	65.51	0	0	chr4	1449

AcUGT2	**6.1**	**0.09**	**50.77**	**0.09**	**0**	**chr7**	**1365**
***AcUGT94AK6**	**0.16**	**0.76**	**44.46**	**0**	**0.22**	**chr9**	**1338**
AcUGT12	3.61	2.57	31.94	0.48	0.63	chr8	1362
***AcCCL3**	**12.57**	**70.62**	**360.25**	**3.65**	**2.12**	**chr19**	**1689**
AcCCL2	**29.88**	**28.43**	**91.15**	**27.36**	**12.67**	**chr19**	**1953**
AcCCL10	**17.44**	**21.8**	**73.02**	**17.9**	**19.26**	**chr7**	**2556**
AcCCL4	4.3	5.54	14.19	0.53	0.56	chr8	1458
AcCCL5	2.86	0.36	12.32	0.51	0.9	chr4	1632

Note: Candidate genes which were successfully expressed were marked as bold font; * represents genes demonstrated to involved in escin Ia biosynthesis.

Supplementary Table 2. Candidate *P450s* identified in biosynthetic genomic clusters (BGCs) and their expression patterns in different tissues.

Gene name	Flower	Pericarp	Seed	Leaf	Branch	Gene length (bp)	Note
AcCYP716A274	15.24	0.03	0.00	0.99	0.88	1437	AcCluster I
AcCYP716A278	**365.00**	**281.04**	**4997.01**	**0.85**	**95.08**	**1422**	**AcCluster I**
AcCYP716BX1	**3.78**	**16.68**	**1677.30**	**0.00**	**0.30**	**1461**	**AcCluster I**
AcCYP716BX3	0.00	0.00	11.99	0.00	0.00	1287	AcCluster I
AcCYP716BX6	0.02	0.00	0.09	0.00	0.00	1461	AcCluster I
AcCYP716A276	13.74	0.00	0.00	0.50	0.00	1449	AcCluster I
AcCYP716A277	0.20	1.29	0.09	0.49	0.67	1452	AcCluster I
AcCYP716A275	**31.75**	**257.90**	**3725.86**	**0.64**	**116.31**	**1356**	**AcCluster II**
AcCYP716BX2	**81.29**	**391.95**	**5327.85**	**3.42**	**150.87**	**1470**	**AcCluster II**
CYP716A-fragment2	0.00	0.00	0.00	0.00	0.00	342	AcCluster III
CYP92A-fragment	0.00	0.00	0.00	0.00	0.00	429	AcCluster IV
CYP92A-fragment4	0.00	0.00	0.00	0.00	0.00	801	AcCluster IV
AcCYP716E58	59.37	0.34	0.00	44.48	0.26	1425	AcCluster V
AcCYP716E59	57.61	0.03	0.03	5.44	0.00	1428	AcCluster V
AcCYP716E55	12.74	87.00	6.73	8.40	18.65	1449	AcCluster V
AcCYP716E56	5.54	6.35	6.31	15.85	9.74	1449	AcCluster V

Note: Candidate genes marked in bold represent those involved in escin Ia biosynthesis.

Supplementary Table 3. ^{1}H (600 MHz) and ^{13}C NMR (150 MHz) data for **7** and **8** in pyridine-d_5.

Position	**7**		**8**	
	δ_C, type	δ_H (J in Hz)	δ_C, type	δ_H (J in Hz)
1	39.5, CH_2	0.98, m	39.4, CH_2	0.98, m
		1.56, m		1.82, m
2	28.5, CH_2	1.82, m	28.5, CH_2	1.80, m
		1.84, m		1.83, m
3	78.4, CH	3.44, dd, (11.4, 4.8)	78.6, CH	3.45, dd, (10.8, 6.0)
4	39.8, C		39.9, C	
5	56.1, CH	1.69, br d, (12.0)	56.3, CH	0.86, br d, (10.8)
6	19.2, CH_2	1.38, br t, (12.0)	19.3, CH_2	1.38, m
		1.56, m		1.58, m
7	34.0, CH_2	1.28, m	33.6, CH_2	1.35, m
		1.52, m		1.52, m
8	40.6, C		40.4, C	
9	48.4, CH	1.66, dd, (10.8, 7.2)	48.6, CH	1.68, t, (9.6)
10	37.6, C		37.8, C	
11	24.3, CH_2	1.83, m	24.3, CH_2	1.92, m
		1.86, m		1.94, m
12	123.2, CH	5.24, br s	123.1, CH	5.50, br s
13	144.4, C		145.0, C	
14	43.4, C		43.1, C	
15	26.2, CH_2	1.12, m	28.5, CH_2	1.27, m
		1.99, m		2.45, br t, (12.6)
16	16.2, CH_2	1.94, m	17.6, CH_2	2.01, m
		2.27, m		2.71, br d, (13.2)
17	42.5, C		53.5, C	
18	43.7, CH	2.25, dd, (13.8, 3.6)	44.0, CH	2.35, br d, (10.8)

19	46.8, CH_2	1.17, br d, (11.4)	46.7, CH_2	1.29, m
		1.97, m		1.96, m
20	32.0, C		32.0, C	
21	43.7, CH_2	1.70, m	43.7, CH_2	1.79, m
		1.75, t, (11.4)		1.81, m
22	74.5, CH	4.41, dd, (11.4, 4.8)	72.1, CH	4.54, br t, (8.4)
23	29.2, CH_3	1.24, s	29.3, CH_3	1.24, s
24	17.0, CH_3	1.04, s	17.1, CH_3	1.03, s
25	16.2, CH_3	0.92, s	16.0, CH_3	0.87, s
26	17.3, CH_3	0.96, s	18.0, CH_3	1.07, s
27	26.8, CH_3	1.35, s	27.2, CH_3	1.32, s
28	69.6, CH_2	3.75, d, (10.8)	not observed	
		4.25, d, (10.8)		
29	34.0, CH_3	0.96, s	34.0, CH_3	0.99, s
30	25.4, CH_3	1.01, s	25.7, CH_3	1.10, s

Supplementary Table 4. ^{1}H (600 MHz) and ^{13}C NMR (150 MHz) data for **10** in pyridine-d_5.

Position	**10**	
	δ_C, type	δ_H (J in Hz)
1	39.5, CH_2	1.43, td, (13.2, 3.6)
		1.58, m
2	28.6, CH_2	1.84, m
		1.85, m
3	78.5, CH	3.44, m
4	39.8, C	
5	56.2, CH	0.86, br d, (11.4)
6	19.2, CH_2	1.38, m
		1.57, m
7	33.4, CH_2	1.26, m
		1.52, dd, (12.6, 3.6)
8	40.1, C	
9	48.4, CH	1.68, dd, (11.4, 7.2)
10	37.7, C	
11	24.3, CH_2	1.86, m
		1.89, m
12	123.6, CH	5.29, br s
13	144.2, C	
14	42.7, C	
15	26.2, CH_2	1.09, m
		1.96, m
16	18.3, CH_2	1.94, m
		2.36, m
17	43.9, C	
18	42.4, CH	2.53, dd, (13.8, 4.2)

19	47.2, CH_2	1.36, dd, (13.8, 4.2)
		1.82, t, (13.8)
20	36.9, C	
21	77.1, CH	4.36, d, (10.2)
22	77.3, CH	3.81, d, (10.2)
23	29.2, CH_3	1.24, s
24	17.0, CH_3	1.10, s
25	16.2, CH_3	1.05, s
26	17.3, CH_3	0.96, s
27	27.0, CH_3	1.30, s
28	68.4, CH_2	3.81, d, (10.2)
		4.17, d, (10.2)
29	30.8, CH_3	1.26, s
30	19.6, CH_3	1.25, s

Supplementary Table 5. Sequence similarity of four AcUGT enzymes

Protein Name	AcUGT94AK1	AcUGT94AK3	AcUGT94AK6	AcUGT87AZ3
AcUGT94AK1	100.00%			
AcUGT94AK3	93.50%	100.00%		
AcUGT94AK6	90.52%	92.53%	100.00%	
AcUGT87AZ3	40.21%	41.62%	43.19%	100.00%

Supplementary Table 6. Designed primers for cloning *AcP450s* into the pEAQ-HT vector.

Names	Primers (F)	Primers (R)
AcCYP716BX1	aaattcgcgaccggtacgcgtcgacATGGCACCTATTCTCCTTGT	tgaaaccagagttaaaggcctcgagTCAAGTACTTTGTTGGTGGGG
AcCYP716BX2	aaattcgcgaccggtacgcgtcgacATGGCACCCCTTTTCCTTGT	tgaaaccagagttaaaggcctcgagTCAGGTACATTGTTCATGATGAGATTGAA
AcCYP714E67	aaattcgcgaccggtacgcgtcgacATGGAGGGATTCGAGTTTGA	tgaaaccagagttaaaggcctcgagCTATAACTTTCTCACCAAAAGATCGAGT
AcCYP82D185	aaattcgcgaccggtacgcgtcgacATGAATTTCCTTCTTCAATCTCTAAACC	tgaaaccagagttaaaggcctcgagTTATTGGTAGAGATGAAGGGGC
AcCYP75B134	aaattcgcgaccggtacgcgtcgacATGTCTGCTCTGGTTCTCTAC	tgaaaccagagttaaaggcctcgagTCAGTTGGAGACTCTATAAGCTTTTG
AcCYP701A79	aaattcgcgaccggtacgcgtcgacATGGACATTTTTGCGATTTGTTG	tgaaaccagagttaaaggcctcgagCTATTCATTTCTAGGCCTTATGATCAC
AcCYP85A67	aaattcgcgaccggtacgcgtcgacATGGCGGTCTTGTTGTTGAC	tgaaaccagagttaaaggcctcgagTTAGTACTTCGACACCCTTATATGCA
AcCYP710A134	taaattcgcgaccggtacgcgtcgacATGGGTTCTCTTTTATCGTCATCT	tgaaaccagagttaaaggcctcgagTCATTCCGACGAAAGGCTG
AcCYP85A82	aaattcgcgaccggtacgcgtcgacATGCTTTTATTACTGCTTAGAGTAGG	tgaaaccagagttaaaggcctcgagTCAGCAGTAAGAAGAGACCCTAATA
AcCYP724B37	aaattcgcgaccggtacgcgtcgacATGGCTGCACTATCAGGT	tgaaaccagagttaaaggcctcgagTTAAGCATGATCAGCCATTAATGG
AcCYP735A74	aaattcgcgaccggtacgcgtcgacATGGCTATGGTGGTGCAGA	tgaaaccagagttaaaggcctcgagTCACTGATCTAGGGCCCTC

Supplementary Table 7. Designed primers for cloning *AcP450s* into the pESC-HIS vector.

Names	Primers (F)	Primers (R)
AcCYP716BX1	aggagaaaaaaccccggatccATGGCACCTATTCTCCTTGTTCTT	caacttctgttccatgtcgacTCAAGTACTTTGTTGGTGGGGTT
AcCYP716BX2	aggagaaaaaaccccggatccATGGCACCCCTTTTCCTTGT	caacttctgttccatgtcgacTCAGGTACATTGTTCATGATGAGATTG
AcCYP714E67	aggagaaaaaaccccggatccATGGAGGGATTCGAGTTTGAGTAT	caacttctgttccatgtcgacCTATAACTTTCTCACCAAAAGATCGAG

Supplementary Table 8. Designed primers for cloning *AcUGTs*, *AcCCLs* and *AcBAHDs* into pET-series vectors .

Vector name	Gene name	Primers (F)	Primers (R)
pET-32a(+)	***AcUGT94AK3**	gccatggctgatatcggatccATGGAAAGCAAGGAAGAAAAGGC	tgcggccgcaagcttgtcgacTTACAGTACATGAGTTTTCTTTGATTCTTC
	AcUGT7	gccatggctgatatcggatccATGAAGATTCCGGCTGAGACG	tgcggccgcaagcttgtcgacTTACAGTACATGAGTTTTCTTTGATTCT
	***AcUGT94AK1**	gccatggctgatatcggatccATGGAAAGAAAGCAAGAAAAGGC	tgcggccgcaagcttgtcgacTTACAGTACATGAGTTTTCTTTGATTCTTC
	AcUGT8	gccatggctgatatcggatccATGGGTTCTCAATCTCTTGTTCATG	tgcggccgcaagcttgtcgacCTAGCAAGTGACCTCCACGCTT
	AcUGT14	gccatggctgatatcggatccATGGAAAAACCCGGCAGC	tgcggccgcaagcttgtcgacTCAGGAGCTGCAGCGTAGTTG
	***AcUGT87AZ3**	gccatggctgatatcggatccATGGATTCCAACAATTTGCAGC	tgcggccgcaagcttgtcgacTTAGGCTTGTGTAATGTCCTTGATG
	AcUGT4	gccatggctgatatcggatccATGGCTATGGCTGAACCAGAAA	tgcggccgcaagcttgtcgacTCATGGTTTAGTACTTGCTGCATTG
	AcUGT15	gccatggctgatatcggatccATGGAGATACAAGGCGGAGGA	tgcggccgcaagcttgtcgacCTAGGAAGACAAAATCAGGATGGC
	AcUGT16	gccatggctgatatcggatccATGGAGGAGGAGGAGCAAGG	tgcggccgcaagcttgtcgacTCAAGCGAAGTGGACCTTCTTT
	AcUGT1	gccatggctgatatcggatccATGGCCGCCACCCAAATC	tgcggccgcaagcttgtcgacTTAATAGTGCTTGCTCTGCCTATGC
	AcUGT9	gccatggctgatatcggatccATGAAAATCCAAAAACCACATGTAGT	tgcggccgcaagcttgtcgacTCAAGAACCTTGAGCCTTTGCG
	AcUGT17	gccatggctgatatcggatccATGGCGGATGAACAAGTATTGC	tgcggccgcaagcttgtcgacTTACTCGCTGCAAGGATGATTCT
	AcUGT2	gccatggctgatatcggatccATGTCAAAGGGTCACACCATCC	tgcggccgcaagcttgtcgacCTACTTGCCATGTGCTATGACATTT
	***AcUGT94AK6**	gccatggctgatatcggatccATGGAAAGCAAGGAA	tgcggccgcaagcttgtcgacTTACAGTACATGAGTTTTCTTTG

		GAAAAGGC	ATTCTTC
	AcUGT12	gccatggctgatatcggatccATGGTGGTGGAGCAAGGAG	tgcggccgcaagcttgtcgacTTAAAATGATTTGATAAGCTCTAAAAAGTTGTTC
pET-28a(+)	***AcCCL2***	cagcaaatgggtcgcggatccATGGCGAGTACTGCGACG	tgcggccgcaagcttgtcgacTCATGCCTTGCCTCCTTTTGC
	****AcCCL3***	cagcaaatgggtcgcggatccATGGAAGAGCTAAAACCAAACC	tgcggccgcaagcttgtcgacTTAGATTCCATTGGACTTGGAGG
	AcCCL10	cagcaaatgggtcgcggatccATGGCTCGCTCCCCACATG	tgcggccgcaagcttgtcgacTCAAGAATCAGCAAGTGCGATAAG
	AcBAHD11	cagcaaatgggtcgcggatccATGGAAATCCGTATCATTTCTAAAGA	tgcggccgcaagcttgtcgacTTAGAACATAGAAGAAGAAGCATAAGCA
	****AcBAHD13***	cagcaaatgggtcgcggatccATGGAAATCCGTATCATTTCTAAAG	tgcggccgcaagcttgtcgacTTAGAACATAGAAGAAGAAGCAT
	****AcBAHD17***	cagcaaatgggtcgcggatccATGGAAATCCGTATCATTTCTAAAGA	tgcggccgcaagcttgtcgacTTAGAACATAGAAGAAGAAGCAAAAGC

Note: Candidate genes which were successfully expressed were marked as bold font; * represents genes demonstrated to involved in escin Ia biosynthesis.

Supplementary Table 9. Designed primers for cloning *AcCSL1*, *AcUGTs*, *AcCCL3* and *AcBAHDs* into pEAQ-HT vector.

Name	Primers (F)	Primers (R)
AcCSL1	aaattcgcgaccggtacgcgtcgacATGGGTGATCTTCATTCCCG	tgaaaccagagttaaaggcctcgagTTAATTTGTTTTGCTCTTGCTTGAAATTA
AcUGT94AK1	ctgcccaaattcgcgaccggtATGGAAAGAAAGCAAGAAAAGGC	accagagttaaaggcctcgagTTACAGTACATGAGTTTTCTTTGATTCTTC
AcUGT94AK3	ctgcccaaattcgcgaccggtATGGAAAGCAAGGAAGAAAAGGC	accagagttaaaggcctcgagTTACAGTACATGAGTTTTCTTTGATTCTTC
AcUGT94AK6	ctgcccaaattcgcgaccggtATGGAAAGCAAGGAAGAAAAGGC	accagagttaaaggcctcgagTTACAGTACATGAGTTTTCTTTGATTCTTC
AcUGT87AZ3	ctgcccaaattcgcgaccggtATGGATTCCAACAATTTGCAGC	accagagttaaaggcctcgagTTAGGCTTGTGTAATGTCCTTGATG
AcCCL3	ctgcccaaattcgcgaccggtATGGAAGAGCTAAAACCAAACC	accagagttaaaggcctcgagTTAGATTCCATTGGACTTGGAGG
AcBAHD6	ctgcccaaattcgcgaccggtATGGAAAAAAGCAGCATCATTTC	accagagttaaaggcctcgagTCATGTGGTTGGATTAAAGGAAG
AcBAHD13	ctgcccaaattcgcgaccggtATGGAAATCCGTATCATTTCTAAAG	accagagttaaaggcctcgagTTAGAACATAGAAGAAGAAGCAT
AcBAHD17	ctgcccaaattcgcgaccggtATGGAAATCCGTATCATTTCTAAAGA	accagagttaaaggcctcgagTTAGAACATAGAAGAAGAAGCAAAAGC

Supplementary Table 10. Parameters of the Multiple Reaction Monitoring (MRM) for **2**, **12**, **4**, and **1**.

Compounds	Ion source	Ion mode	Precursor ion (*m/z*)	Product Ion (*m/z*)	Fragmentor (V)	Collision energy voltage (V)
**2**	APCI	positive	489.4	453.3/471.7	140/150	15/10
**12**	AJS ESI	Negative	681.4	421.3/505.4	150	44/46
**4**	AJS ESI	Negative	1005.5	645.4/781.5	150	44/42
**1**	AJS ESI	Positive	1131.5	969.5/789.5	170	12/15

Supplementary Table 11. Linear ranges, calibration curves and correlation coefficients (R^2) for **2**, **12**, **4**, and **1** obtained by LC-QQQ-MS.

Compounds	Linear range (ug/mL)	Calibration curve	R^2
**2**	0.3125-20	y = 509.92x - 228.66	0.9990
**12**	0.2604-20	y = 253.23x + 124.12	0.9986
**4**	0.2604-20	y = 625.15x + 192.92	0.9993
**1**	0.2604-20	y = 1679.4x + 41.375	0.9993

Supplementary Table 12. Specific parameters of LC-MS/MS analysis of enzymatic products.

Detection targets	Column	Mobile phases	Gradient programs	Mass spectrometer type/ scan type/ polarity
Enzymatic assays of AcP450s	Agilent Infinity Lab Poroshell 120 EC-C18 column (2.7 µm, 2.1×150mm)	A, water containing 0.1% formic acid;<br>B, methanol	0 min, 50% B;<br>6 min, 60% B;<br>12 min, 90%;<br>15-20 min, 100% B, 21-25 min, 50% B	LC-QTOF-MS/MS;<br>Auto MS/MS mode;<br>Positive mode
Quantification of protoaescegenin (**2**)	Agilent Infinity Lab Poroshell 120 EC-C18 column (2.7 µm, 2.1×150mm)	A, water containing 0.1% formic acid;<br>B, methanol	0 min, 50% B;<br>6 min, 60% B;<br>12 min, 90% B;<br>15-20 min, 100% B, 21-25 min, 50% B	LC-QQQ-MS/MS;<br>MRM mode;<br>Positive mode
Preliminary Functional Evaluation of AcUGTs	Waters Acquity UPLC® HSS T3 column (1.8 µm, 2.1×100 mm)	A, water containing 0.1% formic acid;<br>B, acetonitrile	0-6 min, 20% B<br>12-14 min, 100% B;<br>15 min, 20% B	LC-QTOF-MS/MS;<br>Auto MS/MS mode;<br>Negative mode
Further confirmation of the AcUGTs and AcBAHDs function	Waters Acquity UPLC® HSS T3 column (1.8 µm, 2.1×100 mm)	A, water containing 0.1% formic acid;<br>B, acetonitrile	0 min, 5% B;<br>20 min, 50% B;<br>28 min, 70% B;<br>30-32 min, 100% B;<br>32.1-35 min, 5% B	LC-QTOF-MS/MS;<br>Auto MS/MS mode;<br>Negative mode.
The third optimized elution gradient to distinguish **13** and **14**	Agilent Infinity Lab Poroshell 120 EC-C18 column (2.7 µm, 2.1×150mm)	A, water;<br>B, acetonitrile	0 min, 20% B;<br>6 min, 26% B;<br>7 min, 100% B	LC-QTOF-MS/MS;<br>MS mode;<br>Negative mode.
Enzymatic assays of AcCCLs	Ultimate® XB-C18 column (3 µm, 2.1×100 mm)	A, water containing 0.1% formic acid;<br>B, acetonitrile	0-5 min, 5% B;<br>10 min, 50% B;<br>13-15 min, 100% B<br>15.5-18.5 min, 5% B	LC-QTOF-MS/MS;<br>Auto MS/MS mode;<br>Negative mode
Quantification of escin Ia (**1**)	Waters Acquity UPLC® HSS T3 column (1.8 µm, 2.1×100 mm)	A, water containing 0.1% formic acid;<br>B, acetonitrile	0 min, 5% B;<br>20 min, 50% B;<br>28 min, 70% B;<br>30-32 min, 100% B;<br>32.1-35 min, 5% B	LC-QQQ-MS/MS;<br>MRM mode;<br>Positive mode
Quantification of **12** and **4**	Ultimate® XB-C18 column (3 µm, 2.1×100 mm)	A, water containing 0.1% formic acid;<br>B, acetonitrile	0 min, 5% B;<br>20 min, 50% B;<br>28 min, 70% B;<br>30-32 min, 100% B;<br>32.1-35 min, 5% B	LC-QQQ-MS/MS;<br>MRM mode;<br>Negative mode

Supplementary Table 13. Mass errors (ppm) of key compounds which were either authentic standards or purified components.

ID	Compound name	Detection method	Characteristic ion	detected value	theoretical value	ppm
**1**	Escin Ia	LC-QTOF ESI $[M-H]^-$	$[C_{55}H_{85}O_{24}]^-$	1129.5473	1129.5436	3.2756
**2**	Protoaescigenin	LC-QTOF APCI $[M+H-H_2O]^+$	$[C_{30}H_{49}O_5]^+$	489.3589	489.3580	1.8391
**3**	β-amyrin	LC-QTOF APCI $[M+H-H_2O]^+$	$[C_{30}H_{49}]^+$	409.3835	409.3834	0.2443
**4**	3β-*O*-{β-D-glucopyranosyl-(1→4)-[β-D-glucopyranosyl-(1→2)]-β-D-glucuronopyranosyl acid}-protoaescigenin (aesculuside B)	LC-QTOF ESI $[M-H]^-$	$[C_{48}H_{77}O_{22}]^-$	1005.5004	1005.4912	9.1497
**6**	Tigloyl-CoA	LC-QTOF ESI $[M-2H]^{2-}$	$[C_{26}H_{40}N_7O_{17}P_3S]^{2-}$	423.5722	423.5713	2.1248
**7**	22α-hydroxy-erythrodiol	LC-QTOF APCI $[M+H-H_2O]^+$	$[C_{30}H_{49}O_2]^+$	441.3732	441.3733	-0.2266
**8**	22α-hydroxy-oleanic acid	LC-QTOF APCI $[M+H-H_2O]^+$	$[C_{30}H_{47}O_3]^+$	455.3556	455.3525	6.8079
**9**	21β-hydroxy-β-amyrin	LC-QTOF APCI $[M+H-H_2O]^+$	$[C_{30}H_{49}O]^+$	425.3782	425.3783	-0.2351
**10**	21β,22α-dihydroxy-erythrodiol	LC-QTOF APCI $[M+H-H_2O]^+$	$[C_{30}H_{49}O_3]^+$	457.3693	457.3682	2.4051
**11**	Barringtogenol C	LC-QTOF APCI $[M+H-H_2O]^+$	$[C_{30}H_{49}O_4]^+$	473.3630	473.3631	-0.2113
**12**	3β-*O*-{β-D-glucuronopyranosyl acid}-protoaescigenin	LC-QTOF ESI $[M-H]^-$	$[C_{36}H_{57}O_{12}]^-$	681.3891	681.3856	5.1366
**13**	3β-*O*-{[β-D-glucopyranosyl-(1→2)]-β-D-glucuronopyranosyl acid}-protoaescigenin (aeswilsaponin IC)	LC-QTOF ESI $[M-H]^-$	$[C_{42}H_{67}O_{17}]^-$	843.4403	843.4384	2.2527
**14**	3β-*O*-{[β-D-glucopyranosyl-(1→4)]-β-D-glucuronopyranosyl acid}-protoaescigenin	LC-QTOF ESI $[M-H]^-$	$[C_{42}H_{67}O_{17}]^-$	843.4422	843.4384	4.5053
**15**	Desacetylescin Ia	LC-QTOF ESI $[M-H]^-$	$[C_{53}H_{83}O_{23}]^-$	1087.5453	1087.5331	11.2179

References

1. Wilson, A. E.&Tian, L. Phylogenomic analysis of UDP-dependent glycosyltransferases provides insights into the evolutionary landscape of glycosylation in plant metabolism. *The Plant Journal*. **100**, 1273-1288 (2019).
2. Jian, X.et al. Engineering the substrate specificity of UDP-glycosyltransferases for synthesizing triterpenoid glycosides with a linear trisaccharide as aided by ancestral sequence reconstruction. *Angewandte Chemie International Edition*. **63**, e202409867 (2024).
3. Martin, L. B. B.et al. Complete biosynthesis of the potent vaccine adjuvant QS-21. *Nat. Chem. Biol.*, (2024).
4. Shockey, J. M., Fulda, M. S.&Browse, J. Arabidopsis contains a large superfamily of acyl-activating enzymes. Phylogenetic and biochemical analysis reveals a new class of acyl-coenzyme A synthetases. *Plant Physiol.* **132**, 1065-1076 (2003).
5. Callari, R., Fischer, D., Heider, H.&Weber, N. Biosynthesis of angelyl-CoA in *Saccharomyces cerevisiae*. *Microb. Cell. Fact.* **17**, 1-14 (2018).
6. Sofeo, N.et al. Altering the substrate specificity of acetyl-CoA synthetase by rational mutagenesis of the carboxylate binding pocket. *ACS Synth. Biol.* **8**, 1325-1336 (2019).
7. Moghe, G.et al. BAHD company: the ever-expanding roles of the BAHD acyltransferase gene family in plants. *Annu. Rev. Plant Biol.* **74**, 165-194 (2023).
