# Supplementary material for: Complete biosynthesis of the clinical agent escin Ia
Source: Nat Commun. 2026 Aug 18;17:9891. doi: 10.1038/s41467-026-76851-3 (PMC13582942; doi:10.1038/s41467-026-76851-3)
Supplement: Supplementary file 2 [file 41467_2026_76851_MOESM2_ESM.pdf]

nature portfolio

Corresponding author(s): Wei Sun

Last updated by author(s): Jul 22, 2026

Reporting Summary

Nature Portfolio wishes to improve the reproducibility of the work that we publish. This form provides structure for consistency and transparency in reporting. For further information on Nature Portfolio policies, see our Editorial Policies and the Editorial Policy Checklist.

Statistics

For all statistical analyses, confirm that the following items are present in the figure legend, table legend, main text, or Methods section.

n/a	Confirmed	
☐	☒	The exact sample size (*n*) for each experimental group/condition, given as a discrete number and unit of measurement
☐	☒	A statement on whether measurements were taken from distinct samples or whether the same sample was measured repeatedly
☐	☒	The statistical test(s) used AND whether they are one- or two-sided<br>*Only common tests should be described solely by name; describe more complex techniques in the Methods section.*
☒	☐	A description of all covariates tested
☐	☒	A description of any assumptions or corrections, such as tests of normality and adjustment for multiple comparisons
☐	☒	A full description of the statistical parameters including central tendency (e.g. means) or other basic estimates (e.g. regression coefficient) AND variation (e.g. standard deviation) or associated estimates of uncertainty (e.g. confidence intervals)
☐	☒	For null hypothesis testing, the test statistic (e.g. *F*, *t*, *r*) with confidence intervals, effect sizes, degrees of freedom and *P* value noted<br>*Give P values as exact values whenever suitable.*
☒	☐	For Bayesian analysis, information on the choice of priors and Markov chain Monte Carlo settings
☒	☐	For hierarchical and complex designs, identification of the appropriate level for tests and full reporting of outcomes
☒	☐	Estimates of effect sizes (e.g. Cohen's *d*, Pearson's *r*), indicating how they were calculated

Our web collection on statistics for biologists contains articles on many of the points above.

Software and code

Policy information about availability of computer code

Data collection	LC-MS/MS data were collected using Agilent MassHunter Data Acquisition 10.0 software. All NMR data were evaluated employing Bruker topspin-4.4.0 software.
Data analysis	To better screen candidate genes, we further updated the annotation of the A. chinensis genome by integrating both short-read and long-read transcriptomic data. Specifically, short-read RNA-seq data were aligned, assembled, and merged using HISAT2 (v2.1.0)80 and StringTie (v2.0), while full-length transcriptome data were aligned with the isoseq3 (v3.4.0) package81. For phylogenetic analysis, MAFFT (version 7.526), MAFFT (version 7.526), IQ-TREE (version 2.3.6), and ITOL v7 were used. Gene duplication modes were determined through the DupGen_finder pipeline (https://github.com/qiao-xin/DupGen_finder).Gene expression heatmaps were generated using TBtools-II v2.331. LC-MS raw files and exact m/z values were analyzed using Agilent MassHunter Qualitative Analysis 10.0 software. Molecular docking and molecular dynamics simulations were generated using AlphaFold3, AutoDock4.2.6, AMBER22, GAFF2, and TIP3P.

For manuscripts utilizing custom algorithms or software that are central to the research but not yet described in published literature, software must be made available to editors and reviewers. We strongly encourage code deposition in a community repository (e.g. GitHub). See the Nature Portfolio guidelines for submitting code & software for further information.

Data

Policy information about availability of data

All manuscripts must include a data availability statement. This statement should provide the following information, where applicable:

- Accession codes, unique identifiers, or web links for publicly available datasets
- A description of any restrictions on data availability
- For clinical datasets or third party data, please ensure that the statement adheres to our policy

The nucleotide sequences of the functionally characterized genes in this study have been deposited in the GenBase database at Beijing Institute of Genomics, Chinese Academy of Sciences (https://ngdc.cncb.ac.cn/genbase), under the following accession codes: AcCYP716BX1 (C_AA118697.1), AcCYP716BX2 (C_AA118699.1), AcCYP714E67 (C_AA118693.1), AcUGT94AK1 (C_AA118701.1), AcUGT94AK3 (C_AA118702.1), AcUGT94AK6 (C_AA118700.1), AcUGT87AZ3 (C_AA118703.1), AcCCL3 (C_AA118698.1), AcBAHD13 (C_AA118694.1), and AcBAHD17 (C_AA118695.1). The updated gene annotation of A. chinensis has been deposited in the Figshare database (https://doi.org/10.6084/m9.figshare.21350865.v2). The raw analytical data, including all LC-MS datasets and NMR spectra, have been deposited in the Figshare database (https://doi.org/10.6084/m9.figshare.32613411). Both datasets are publicly and fully accessible. Source data are provided with this paper.

Research involving human participants, their data, or biological material

Policy information about studies with human participants or human data. See also policy information about sex, gender (identity/presentation), and sexual orientation and race, ethnicity and racism.

Reporting on sex and gender	This study does not involve human participants or data; therefore, sex- and gender-based analyses are not applicable.
Reporting on race, ethnicity, or other socially relevant groupings	Not applicable. This study does not involve human subjects, demographic data, or human social groupings.
Population characteristics	Not applicable. No human participants or population datasets were utilized in this study.
Recruitment	Not applicable. This study does not involve the recruitment of human participants.
Ethics oversight	Not applicable. This study is focused entirely on plant secondary metabolism and does not require human research ethics oversight.

Note that full information on the approval of the study protocol must also be provided in the manuscript.

Field-specific reporting

Please select the one below that is the best fit for your research. If you are not sure, read the appropriate sections before making your selection.

☒ Life sciences ☐ Behavioural & social sciences ☐ Ecological, evolutionary & environmental sciences

For a reference copy of the document with all sections, see nature.com/documents/nr-reporting-summary-flat.pdf

Life sciences study design

All studies must disclose on these points even when the disclosure is negative.

Sample size	No statistical methods were used to predetermine sample size. The sample size (n = 3 distinct biological replicates) was chosen based on historical data and standard protocols in plant metabolic engineering and enzymatic assays, which provide sufficient statistical power to confirm pathway reconstruction and ensure reproducibility.
Data exclusions	No data were excluded from the analyses. All experimental replicates and successful measurements were fully included in the manuscript.
Replication	All attempts at replication were successful. Every stage of escin Ia biosynthesis, enzymatic assay, and Nicotiana benthamiana co-expression analysis was performed with at least three independent biological replicates to robustly verify the reproducibility of the experimental findings.
Randomization	Randomization was not relevant to this study because the experimental groups consisted of distinct, well-defined gene combinations or specific enzyme-substrate reaction mixtures. Cultivated Nicotiana benthamiana plants of similar age and growth conditions were used uniformly across all infiltration groups to control for environmental covariates.
Blinding	Blinding was not relevant to this study because sample identities (gene combinations, enzymatic products) were objective and defined by strict biochemical and molecular descriptors. Data collection and quantification via automated LC-MS, MS/MS spectra, and DTIM-MS left no room for subjective investigator bias.

Reporting for specific materials, systems and methods

We require information from authors about some types of materials, experimental systems and methods used in many studies. Here, indicate whether each material, system or method listed is relevant to your study. If you are not sure if a list item applies to your research, read the appropriate section before selecting a response.

Materials & experimental systems

n/a	Involved in the study
☒	☐ Antibodies
☒	☐ Eukaryotic cell lines
☒	☐ Palaeontology and archaeology
☒	☐ Animals and other organisms
☒	☐ Clinical data
☒	☐ Dual use research of concern
☐	☒ Plants

Methods

n/a	Involved in the study
☒	☐ ChIP-seq
☒	☐ Flow cytometry
☒	☐ MRI-based neuroimaging

Dual use research of concern

Policy information about dual use research of concern

Hazards

Could the accidental, deliberate or reckless misuse of agents or technologies generated in the work, or the application of information presented in the manuscript, pose a threat to:

No	Yes
☒	☐ Public health
☒	☐ National security
☒	☐ Crops and/or livestock
☒	☐ Ecosystems
☒	☐ Any other significant area

Experiments of concern

Does the work involve any of these experiments of concern:

No	Yes
☒	☐ Demonstrate how to render a vaccine ineffective
☒	☐ Confer resistance to therapeutically useful antibiotics or antiviral agents
☒	☐ Enhance the virulence of a pathogen or render a nonpathogen virulent
☒	☐ Increase transmissibility of a pathogen
☒	☐ Alter the host range of a pathogen
☒	☐ Enable evasion of diagnostic/detection modalities
☒	☐ Enable the weaponization of a biological agent or toxin
☒	☐ Any other potentially harmful combination of experiments and agents

Plants

Seed stocks	Nicotiana benthamiana seeds used in this study were obtained from our laboratory collection (originally maintained at the Institute of Chinese Materia Medica, China Academy of Chinese Medical Sciences). No wild plant specimens were collected from the field; therefore, field collection descriptors and sampling procedures are not applicable.
Novel plant genotypes	No stable novel plant genotypes or transgenic lines were generated or used in this study. Gene expression and pathway reconstruction experiments (co-expression of biosynthetic genes) were carried out strictly via transient expression using Agrobacterium tumefaciens-mediated infiltration (agro-infiltration) on the leaves of 4- to 5-week-old wild-type Nicotiana benthamiana plants.
Authentication	The identity of the Nicotiana benthamiana strain was authenticated by its characteristic morphological features, including growth habit, leaf morphology, and flowering traits under standard greenhouse conditions (25°C, 16 h light/8 h dark cycle). Plants were monitored continuously to ensure they were healthy, uniform, and free of viral or bacterial symptoms prior to agro-infiltration.
