# Supplementary material for: Complete biosynthesis of the clinical agent escin Ia
Source: Nat Commun. 2026 Aug 18;17:9891. doi: 10.1038/s41467-026-76851-3 (PMC13582942; doi:10.1038/s41467-026-76851-3)
Supplement: Supplementary file 3 [file 41467_2026_76851_MOESM3_ESM.pdf]

nature portfolio

Peer Review File

Complete Biosynthesis of the Clinical Agent Escin Ia

Corresponding Author: Dr Wei Sun

This file contains all reviewer reports in order by version, followed by all author rebuttals in order by version.

Version 0:

Reviewer comments:

Reviewer #1

(Remarks to the Author)
This research on the total biosynthesis of clinical drug Escin Ia represents a significant advance in the field. It not only systematically characterizes the function of CYP450, UGT, AT and CCLs involved in Escin Ia biosynthesis but also establishes a foundation for the sustainable production of complex triterpenoid saponins. Despite its outstanding scientific achievements, the authors need to further improve on the following points to enhance the manuscript quality.

Major point:
1.In line 101, the author explicitly labels 21β-hydroxy-β-amyrin as (9). However, the author states, "LC-MS analysis identified the catalytic product as Desacetylescin Ia (9) in line 191. Please revise it.
2.Add the "Ac" prefix to the UGT nomenclature in Figure 3
3.It would be beneficial to include a discussion regarding future research directions for Escin Ia biosynthesis in the Conclusion section.
4.The specific details, yields, or analytical proof for the *Escin* Ia produced in N. benthamiana are lacking in the provided method.
5.In line 355, the authors mentioned that the reaction system was supplemented with "0.2 mM crotonyl-CoA. However, the core focus of the present study is the acylation involving tigloyl-CoA (which is catalyzed by AcCCL3).
6.The authors acknowledge the limitation in determining the exact hydroxylation sequence due to a lack of commercial standards. I recommend a more in-depth discussion of the likely order of C-22a, C-24, and C-28 hydroxylations based on the isolated intermediates (such as compounds 7, 8, 10, 11), which would enhance the mechanistic insight into pathway regulation.

Minor point：
Line 22: “Aa a result” should be “As a result”.
Line 35: “potion” should be “position”.
Line 47: “AcOCS6” should be “AcOSC6”.
Line 49: “was consisted of” should be “consisted of” or “was composed of”.
Line 121: “the enzymes… remains undefined” should be “remain undefined”.
Line 179: “succussed” should be “succeeded”.
Line 194: “were belong to” should be “belonged to”.
Line 200: “catalysis the formation” should be “catalyzes the formation”.
Line 234: “funtioning” should be “functioning”.
Line 255: “variou” should be “various”.
Line 267: “involved forming” should be “involved in forming”.
Line 273: “clustere” should be “clustered”.
Line 276: “clade 3 o BAHDs” should be “clade 3 of BAHDs”.
Line 284: “noval” should be “novel”.
Line 370: “conFigured” should be “configured”.

(Remarks on code availability)

Reviewer #2

(Remarks to the Author)
This manuscript reports the complete elucidation of the biosynthetic pathway of escin Ia, a clinically important barrigenol-type triterpenoid saponin from Aesculus chinensis. Building on prior genomic analyses, the authors identify and functionally characterize ten previously unknown enzymes encompassing cytochrome P450-mediated polyhydroxylation, stepwise glycosylation, tiglic acid activation and BAHD-dependent acylation. The study combines co-expression analysis, phylogenetics, in vitro enzymology, transient expression in Nicotiana benthamiana, metabolite purification and extensive structural validation. The authors further demonstrate heterologous reconstruction of the full pathway in planta, thereby establishing a framework for sustainable production. Overall, the work is technically ambitious and addresses an important problem in plant specialized metabolism, with clear relevance to both fundamental biosynthetic logic and applied metabolic engineering.

Major comments

1. The central claim of “complete biosynthesis” is largely convincing; however, the manuscript would benefit from a clearer and more rigorous discussion of pathway order and enzyme promiscuity. Several cytochrome P450s, particularly AcCYP716BX1/2 and AcCYP714E67, are inferred to act in a defined sequence based primarily on transient co-expression and accumulation of intermediates. The authors do not convincingly exclude alternative orders of hydroxylation or partial functional redundancy with endogenous N. benthamiana enzymes. A more explicit justification of reaction order, ideally supported by targeted feeding experiments or time-resolved analyses, would strengthen the mechanistic conclusions rather than leaving them implicit.

2. While the identification of C-22α hydroxylation of β-amyrin is indeed noteworthy, the assertion that “no CYP450s responsible for C-22α hydroxylation of the β-amyrin skeleton has been reported until now” is incorrect. CYP716A2 has previously been demonstrated to catalyze C-22α hydroxylation in Arabidopsis, and this prior work should be explicitly acknowledged and discussed to accurately position the present findings within the existing literature.

3. The glycosylation section is one of the strongest components of the manuscript, particularly the use of DTIM-MS to distinguish regioisomeric products. However, the conclusion that 2′-O-glucosylation is strictly required before 4′-O-glucosylation is based entirely on negative in vitro results. While this hierarchical model is plausible, it would be more convincing if supported by kinetic data or a structural rationale. At present, the conclusion appears stronger than the data justify. In addition, the reported drift time difference between the two regioisomeric products is very small. The authors should address whether higher-resolution ion mobility instrumentation or an orthogonal analytical approach could be used to further validate these assignments.

4. The heterologous reconstruction of escin Ia in N. benthamiana is an important validation of pathway completeness, but the reported yields are low and the manuscript does not sufficiently address this limitation. Given the applied motivation emphasized throughout the abstract and discussion, the authors should comment more directly on potential metabolic bottlenecks, compound toxicity, or flux constraints. Even a qualitative comparison with yields reported for other complex triterpenoid saponins would help place these results in a more realistic applied context.

5. Finally, the Discussion would benefit from a dedicated paragraph addressing the possible biological roles of escin Ia and related intermediates in the plant itself. Given the extensive biosynthetic investment described, some consideration of ecological or physiological function would add valuable biological context.

Minor comments

1. Throughout the manuscript, terminology alternates between “tiglylation” and “tigloylation.” This should be standardized to avoid confusion, particularly for readers outside the immediate field.

2. The Discussion occasionally reiterates background material already covered in the Introduction, especially regarding general triterpenoid biosynthesis and UGT family classification. Reducing this redundancy would sharpen the focus on the genuinely novel aspects of the work.

3. There are a number of minor grammatical and typographical issues, including inconsistent spacing, missing articles and occasional tense errors, which should be corrected during revision. While none affect the scientific content, they detract from the overall polish expected at this level.

4. The Data Availability section clearly lists accession numbers and repositories, but it would be helpful to explicitly state whether raw LC-MS and NMR data are available, and if so, where. Given the structural claims made throughout the manuscript, transparency here is important.

5. There is an issue with the following sentence in the Abstract: “AcCCL3 6 catalyzes tiglic acid to form tigloyl-CoA, and AcBAHD13/17 catalyze C-21β tigloylation of aesculuside B to yield desacetylescin Ia”. This sentence should be corrected for clarity.

6. In the Introduction, the statement that saponins are “also known as triterpene glycosides” is misleading, as it excludes steroidal saponins. This should be rephrased.

7. In several places, the authors refer to the discovery of CSL enzymes involved in glucuronic acid attachment to triterpenoid aglycones but do not cite the original studies that first reported these activities. Appropriate primary references should be included.

8. The manuscript frequently refers to the “turquoise module” identified by WGCNA, but no information is provided about other modules. A brief explanation would improve clarity for readers unfamiliar with the analysis.

9. In the Supplementary Dataset, several MS/MS spectra appear to be of poor quality. The authors should consider rerunning these analyses using different collision energies or higher analyte loading to improve spectral quality and confidence in fragment assignments.

(Remarks on code availability)

Reviewer #3

(Remarks to the Author)
The authors of this paper investigate the biosynthesis of Escin Ia, a pharmaceutically important triterpene saponin, and elucidate key pathway genes required. To do this they utilised a transcriptomics and weighted co-expression gene network analysis utilising 5 previously determined genes that are involved in the biosynthesis. From this they identified a module that was seed specific (matching the location of biosynthesis) and identified 43 potential biosynthesis genes. Subsequently they identify two AcCYP716 genes that can convert B-amyrin to 21B,22a-dihydroxyerythrodiol. They also identified AcCYP714E67 responsible for protoaescigenin conversion. Following this, they investigated the addition of the hexose groups to the aglycone and identified AcUGT87AZ3 which adds 4’ glucose and AcUGT94AK1/3/6 which adds the 2’ glucose. As the final step they then identified conclusively that BAHD13/17 can catalyse the transfer of the tigloyl group to descetylescin Ia to for Escin Ia and identified the enzyme that converts tiglic acid to tigloyl-CoA which may help with future engineering efforts for production of Escin Ia. This is an exciting and thorough piece of work with new CYPs and UGT enzyme activity described for previously uncharacterised clades and provides potential access to a very important compound.

In my view the authors clearly state their approach to elucidating the Escin Ia pathway and determine the biosynthesis of this molecule with heterologous production in N. benthamiana and in vitro (where possible). They utilise NMR techniques to conclusively confirm the production of the correct molecule and compare to authentic/purified standards throughout the manuscript.

The methods and results are clear; however, it would be helpful to the reader to provide the numbers of replicates used in each experiment within the

figure legends. This data is available in the supplement, and it seems biosynthesis steps were carried out with 3 replicates, which is appropriate, but these details should be in the main body.

Also, while the writing is clear throughout most of the manuscript, the discussion would benefit from some polishing. I have noted a few specific errors below, but the whole section should be carefully revised to improve the grammar and to remove typographical errors.

• Please include the number of replicates carried out in biosynthesis for in vitro and N. benthamiana infiltration experiments in the main figure legend
• Genome mining, phylogenetic analysis, gene expression analysis and gene duplications identifying- please include the substitution model used and how it was selected (e.g. ModelFinder)
• The bars indicating expression in figure 3A and figure 5A do not seem to be aligned with the genes. I am unsure if this is a formatting error or if this indicates something different – please clarify.
• It would be clearer if the Y axis for all EIC traces were on the same scale when in the same plot (e.g. within Figure 3D some show x10^4 and others x10^5)
• Supplemental table 2 & 4 note: “Candidate genes succussed to expressing protein”- should this be successfully expressed?
• Line widths and text boldness varies across figure 3C-H, it would be visually more appealing if they matched
• Figure S42 should include CYP716BX2 in the figure legend
• Line 234: “functioning”
• Line 255: “variou positions”
• Line 273 “ clustere”
• Line 276: “clade 3 o BAHDs”
• Repeated information in Line 231 and line 233: “study is the first to report that a member of the CYP714E subfamily functions as a C-24 hydroxylase of oleanane skeleton” and “There is no report on CYP714E19 funtioning as a oleanane C-24 hydroxylase until now”. Please delete one of these.

(Remarks on code availability)

Reviewer #4

(Remarks to the Author)
I co-reviewed this manuscript with one of the reviewers who provided the listed reports. This is part of the Nature Communications initiative to facilitate training in peer review and to provide appropriate recognition for Early Career Researchers who co-review manuscripts.

(Remarks on code availability)

Version 1:

Reviewer comments:

Reviewer #1

(Remarks to the Author)
The authors have adequately addressed all my comments and the revisions are well done. I recommend acceptance of the manuscript in its current form.

Reviewer #2

(Remarks to the Author)
I thank the authors for their thorough and thoughtful revision. They have done an excellent job addressing all of my comments.

The reframing of the protoaescigenin pathway as a promiscuity-driven metabolic grid, supported by the combinatorial co-expression matrix, is convincing, and the leave-one-out omission controls together with the independent yeast microsomal reconstructions now firmly exclude any contribution from endogenous N. benthamiana enzymes. The prior characterization of CYP716A2-mediated C-22α hydroxylation is now properly acknowledged, and the novelty of AcCYP716BX1/2 is accurately positioned within the existing literature. The glycosylation section has been particularly strengthened: the AlphaFold3 modeling and MD simulations provide a clear structural rationale for the 2-O- before 4-O- sequence, and the orthogonal chromatographic and energy-resolved MS/MS validation resolves my earlier concern about the narrow drift-time difference. The expanded analysis of metabolic flux and bottlenecks, the comparison with other heterologously produced triterpenoid saponins, and the new discussion of the ecological roles of escin Ia all add valuable context. The minor points have likewise been fully resolved.

The manuscript is now of the highest quality. I congratulate the authors on this fantastic body of work and on completing the elucidation of escin Ia biosynthesis, an elegant and impactful contribution to plant specialized metabolism and metabolic engineering. I am pleased to recommend it for publication.

Reviewer #3

(Remarks to the Author)
The main advance reported in this manuscript is the elucidation of the biosynthetic pathway for Escin Ia. I am content with the revisions made by the authors in response to the previous reviews and have no further comments.

Reviewer #4

(Remarks to the Author)
I co-reviewed this manuscript with one of the reviewers who provided the listed reports. This is part of the Nature Communications initiative to facilitate training in peer review and to provide appropriate recognition for Early Career Researchers who co-review manuscripts.

Open Access This Peer Review File is licensed under a Creative Commons Attribution 4.0 International License, which permits use, sharing, adaptation, distribution and reproduction in any medium or format, as long as you give appropriate credit to the original author(s) and the source, provide a link to the Creative Commons license, and indicate if changes were made.
In cases where reviewers are anonymous, credit should be given to 'Anonymous Referee' and the source.
The images or other third party material in this Peer Review File are included in the article's Creative Commons license, unless indicated otherwise in a credit line to the material. If material is not included in the article's Creative Commons license and your intended use is not permitted by statutory regulation or exceeds the permitted use, you will need to obtain permission directly from the copyright holder.
To view a copy of this license, visit https://creativecommons.org/licenses/by/4.0/

Response to Reviewers

Reviewer #1 (Remarks to the Author):

This research on the total biosynthesis of clinical drug Escin Ia represents a significant advance in the field. It not only systematically characterizes the function of CYP450, UGT, AT and CCLs involved in Escin Ia biosynthesis but also establishes a foundation for the sustainable production of complex triterpenoid saponins. Despite its outstanding scientific achievements, the authors need to further improve on the following points to enhance the manuscript quality.

Response: We sincerely appreciate your valuable comments and constructive suggestions on our manuscript. In response to your insightful comments, we have completed the revisions to the manuscript and provided a point-by-point response to each of your concerns.

Major point:

1.In line 101, the author explicitly labels 21β-hydroxy-β-amyrin as (9). However, the author states, "LC-MS analysis identified the catalytic product as Desacetylescin Ia (9) in line 191. Please revise it.

Response: Thanks for your suggestion. 21β-hydroxy-β-amyrin should be labeled as (**9**), however, Desacetylescin Ia should be labeled as (**15**). We have revised it as: "Desacetylescin Ia (**15**)" in line 247.

2.Add the "Ac" prefix to the UGT nomenclature in Figure 3

Response: Thanks for your suggestion. We have added "Ac" prefix to the UGT nomenclature in Figure 3.

3.It would be beneficial to include a discussion regarding future research directions for Escin Ia biosynthesis in the Conclusion section.

Response: We thank the reviewer for this forward-looking suggestion. In accordance with your advice, we have expanded the Conclusion section to discuss future research directions for escin Ia (**1**) biosynthesis. The revised text (lines 380–383) is as follows: "Building upon these findings, future research will focus on enhancing the biosynthetic efficiency of **1** through metabolic engineering, establishing a scaling production platform by transferring this pathway into microbial chassis such as S. cerevisiae, and utilizing this newfound enzymatic repertoire to create next-generation analogues of **1** via combinatorial biosynthesis."

4.The specific details, yields, or analytical proof for the Escin Ia produced in N. benthamiana are lacking in the provided method.

Response: We thank the reviewer for pointing this out. To provide clear and sufficient methodological details regarding the analytical proof and quantification of escin Ia (**1**), we have updated the heading of the relevant section to **"Qualification and quantification analysis of products by LC-MS"** in Methods for improved clarity.

Furthermore, comprehensive details regarding the quantification approach and standard curves have been explicitly added to the revised manuscript (lines 480–482): "The quantitation analysis was conducted by external calibration of the corresponding standards, and the Multiple Reaction Monitoring (MRM) parameters, the linear ranges, calibration curves, and correlation coefficients (R^2) for **2**, **12**, **4**, and **1** are listed in Tables S10–S11."

5.In line 355, the authors mentioned that the reaction system was supplemented with "0.2 mM crotonyl-CoA. However, the core focus of the present study is the acylation involving tigloyl-CoA (which is catalyzed by AcCCL3).

Response: We sincerely thank the reviewer for catching this discrepancy and apologize for the inadvertent oversight. As the reviewer correctly pointed out, the core of this assay involves tigloyl-CoA (generated *in situ* from tiglic acid via AcCCL3 catalysis) for the C-21 acylation, as well as acetyl-CoA for the C-22 acetylation mediated by AcBAHD6.

Here, we removed the erroneous statement from the original manuscript and thoroughly revised the description of the reaction conditions (lines 461–463) to precisely reflect our experimental setup: "Subsequently, 10 μg of AcBAHD13/17 protein and 0.2 mM aesculuside B were supplemented; if AcBAHD6 was also included, 0.2 mM acetyl-CoA was added, followed by continued incubation for a total of 16 h."

6.The authors acknowledge the limitation in determining the exact hydroxylation sequence due to a lack of commercial standards. I recommend a more in-depth discussion of the likely order of C-22a, C-24, and C-28 hydroxylations based on the isolated intermediates (such as compounds 7, 8, 10, 11), which would enhance the mechanistic insight into pathway regulation.

Response: We sincerely thank the reviewer for this constructive suggestion. We fully agree that a detailed discussion of the hydroxylation sequence based on the isolated intermediates significantly enhances the mechanistic depth of our study.

Following your advice, we have substantially expanded the Discussion section regarding the likely order of the C-22α, C-24, and C-28 hydroxylations—along with the C-16α and C-21β

positions of β-amyrin—has been incorporated into lines 297–307: "In recent years, there has been an increasing number of complete elucidations of the metabolic pathways of plant-derived natural products with clinical applications[58, 59]. A growing body of research has discovered that target products often do not arise through a simple linear metabolic process[60]. This study provides a novel framework for elucidating the complex polyhydroxylation mechanisms of barrigenol-type triterpenoids. Intriguingly, based on the profiles of the isolated intermediates (such as **7**, **8** and **11**) and the results of multiplexed transient expression assays, the core biosynthetic steps from β-amyrin (**3**) to protoaescigenin (**2**) do not strictly follow a single, channeled pathway but instead operate as a highly plastic metabolic grid. Driven by the promiscuous nature of the AcCYP716 and AcCYP714 families, the sequential hydroxylations at C-16α, C-21β, C-22α, C-24, and C-28 proceed via complex, interconnected grid routes. Evolving such non-linear, resilient catalytic networks for specialized metabolism represents a classic metabolic hedging strategy in plants. The core ecological significance of this architecture lies in maximizing chemical diversity and metabolic robustness, thereby enhancing the evolutionary fitness and environmental adaptation of the species[61, 62]."

Minor point:

Line 22: "Aa a result" should be "As a result".

Response: Thanks for your suggestion. "Aa a result" has been changed to "As a result".

Line 35: "potion" should be "position".

Response: Thanks for your suggestion. "potion" has been changed to "position".

Line 47: "AcOCS6" should be "AcOSC6".

Response: Thanks for your suggestion. "AcOCS6" has been changed to "AcOSC6".

Line 49: "was consisted of" should be "consisted of" or "was composed of".

Response: Thanks for your suggestion. "was consisted of" has been changed to "consisted of".

Line 121: "the enzymes... remains undefined" should be "remain undefined".

Response: Thanks for your suggestion. "remains undefined" has been changed to "remain

undefined”.

Line 179: “succussed” should be “succeeded”.

Response: Thanks for your suggestion. “succussed” has been changed to “succeeded”.

Line 194: “were belong to” should be “belonged to”.

Response: Thanks for your suggestion. “were belong to” has been changed to “belonged to”.

Line 200: “catalysis the formation” should be “catalyzes the formation”.

Response: Thanks for your suggestion. “catalysis the formation” has been changed to “which catalyzes the formation”.

Line 234: “funtioning” should be “functioning”.

Response: We are sorry, but the sentence containing “functioning” was redundant with the preceding content and has been removed.

Line 255: “variou” should be “various”.

Response: Thanks for your suggestion. “variou” has been changed to “various”.

Line 267: “involved forming” should be “involved in forming”.

Response: Thanks for your suggestion. “involved forming” has been changed to “involved in forming”.

Line 273: “clustere” should be “clustered”.

Response: Thanks for your suggestion. “clustere” has been changed to “clustered”.

Line 276: “clade 3 o BAHDs” should be “clade 3 of BAHDs”.

Response: Thanks for your suggestion. “clade 3 o BAHDs” has been changed to “clade 3 of BAHDs”.

Line 284: “noval” should be “novel”.

Response: Thanks for your suggestion. “noval” has been changed to “novel”.

Line 370: “conFigured” should be “configured”.

Response: Thanks for your suggestion. “conFigured” has been changed to “configured”.

Reviewer #2 (Remarks to the Author):

This manuscript reports the complete elucidation of the biosynthetic pathway of escin Ia, a clinically important barrigenol-type triterpenoid saponin from Aesculus chinensis. Building on prior genomic analyses, the authors identify and functionally characterize ten previously unknown enzymes encompassing cytochrome P450-mediated polyhydroxylation, stepwise glycosylation, tiglic acid activation and BAHD-dependent acylation. The study combines co-expression analysis, phylogenetics, in vitro enzymology, transient expression in Nicotiana benthamiana, metabolite purification and extensive structural validation. The authors further demonstrate heterologous reconstruction of the full pathway in planta, thereby establishing a framework for sustainable production. Overall, the work is technically ambitious and addresses an important problem in plant specialized metabolism, with clear relevance to both fundamental biosynthetic logic and applied metabolic engineering.

Response: We sincerely appreciate your valuable comments and constructive suggestions on our manuscript. In response to your insightful comments, we have completed the revisions to the manuscript and provided a point-by-point response to each of your concerns.

Major comments

1. The central claim of “complete biosynthesis” is largely convincing; however, the manuscript would benefit from a clearer and more rigorous discussion of pathway order and enzyme promiscuity. Several cytochrome P450s, particularly AcCYP716BX1/2 and AcCYP714E67, are inferred to act in a defined sequence based primarily on transient co-expression and accumulation of intermediates. The authors do not convincingly exclude alternative orders of hydroxylation or partial functional redundancy with endogenous N. benthamiana enzymes. A more explicit justification of reaction order, ideally supported by targeted feeding experiments or time-resolved analyses, would strengthen the mechanistic conclusions rather than leaving them implicit.

Response: We sincerely thank the reviewer for this insightful and constructive opinion. We completely agree that the catalytic promiscuity of Cytochrome P450s frequently creates an interconnected metabolic network rather than a strict, linear pathway for the biosynthesis of protoaescigenin (**2**). Given that the intermediate standards were neither commercially available nor accessible in sufficient quantities for traditional sequential feeding assays, we addressed this challenge by designing a comprehensive combinatorial co-expression matrix in *N. benthamiana* to systematically map the metabolic landscape governing protoaescigenin (**2**) biosynthesis.

In addition, we successfully excluded any endogenous plant background interference through both heterologous individual-gene omission (“minus-one”) controls and independent *in vitro* yeast

microsomal reconstructions.

The corresponding revisions have been integrated into the manuscript as detailed below:

Part 1: Addressing Pathway Grid and Substrate Promiscuity

We have thoroughly revised the section **"Biosynthesis of the protoaescigenin (2) scaffold"** (lines 114–125) to clarify the promiscuity landscape: "To further decipher the cooperative biosynthetic cascade from β-amyrin (**3**) to protoaescigenin (**2**), we performed multiplexed transient expression matrices using various gene combinations in *N. benthamiana*. LC-MS analysis revealed no detectable 24-hydroxy-β-amyrin, suggesting that *AcCYP714E67* lacks hydroxylation activity toward β-amyrin at the C-24 position (Figure S29). Additionally, it also failed to catalyze the C-24 hydroxylation of 16α-hydroxy-β-amyrin (**23**). However, with the exception of compounds **3** and **23**, AcCYP714E67 was capable of hydroxylating other tested intermediates at the C-24 position. In contrast, AcCYP716A275, AcCYP716A278, and AcCYP716BX1/2 displayed broader substrate promiscuity, as they were capable of catalyzing regioselective hydroxylations on all tested β-amyrin derivatives with varying degrees of prior hydroxylation (Figure S30). Various gene combinations yielded seven novel intermediate products (**16-22**). Based on the mass spectrometric patterns and diagnostic fragments summarized in Supplementary Figs. 49–71, the chemical structures of **16-22** were putatively identified (Figures S31–37). Collectively, the substrate promiscuity of AcCYP716A275, AcCYP716A278, AcCYP716BX1/2 and AcCYP714E67, results in a complex, grid-like metabolic network for the biosynthetic pathway from β-amyrin (**3**) to protoaescigenin (**2**)."

We have also expanded the **Discussion** section (lines 297–307) to highlight the ecological and evolutionary significance of this metabolic network: "In recent years, there has been an increasing number of complete elucidations of the metabolic pathways of plant-derived natural products with clinical applications[58, 59]. A growing body of research has discovered that target products often do not arise through a simple linear metabolic process[60]. This study provides a novel framework for elucidating the complex polyhydroxylation mechanisms of barrigenol-type triterpenoids. Intriguingly, based on the profiles of the isolated intermediates (such as **7**, **8** and **11**) and the results of multiplexed transient expression assays, the core biosynthetic steps from β-amyrin (**3**) to protoaescigenin (**2**) do not strictly follow a single, channeled pathway but instead operate as a highly plastic metabolic grid. Driven by the promiscuous nature of the AcCYP716 and AcCYP714 families, the sequential hydroxylations at C-16α, C-21β, C-22α, C-24, and C-28 proceed via complex, interconnected grid routes. Evolving such non-linear,

resilient catalytic networks for specialized metabolism represents a classic metabolic hedging strategy in plants. The core ecological significance of this architecture lies in maximizing chemical diversity and metabolic robustness, thereby enhancing the evolutionary fitness and environmental adaptation of the species[61, 62]."

Part 2: Excluding Endogenous Host Interference

To exclude potential functional redundancy with endogenous *N. benthamiana* enzymes, we have highlighted our leave-one-out control strategy and yeast validation in lines 126–135: "To unambiguously eliminate the possibility of functional redundancy contributed by endogenous *N. benthamiana* enzymes, we performed stringent 'leave-one-out' combinatorial omission controls in *N. benthamiana* leaves. LC-MS analysis indicated that **2** was exclusively produced in the two gene pools containing *AstHMGR*, *AcOSC6*, *AcCYP716A278*, *AcCYP716BX1/2*, *AcCYP716A275*, and *AcCYP714E67*. Notably, the omission of any single *CYP450* gene from either pool completely abrogated the production of **2**, confirming that no host-plant CYP450s can complement or bypass the required steps (Figure S38). Furthermore, the independent functions of the newly identified enzymes (AcCYP716BX1, AcCYP716BX2, and AcCYP714E67) were robustly validated using *in vitro Saccharomyces cerevisiae* microsomal assays, yielding results consistent with those observed in *N. benthamiana* (Figures S39–S40). Collectively, transient co-expression of AcCYP716BX1 or AcCYP716BX2 with associated gene modules in *N. benthamiana* leaves produced **2** at a yield of 1294.73 μg/g (dry weight, DW) and 844.02 μg/g (DW), respectively (Figure S41)."

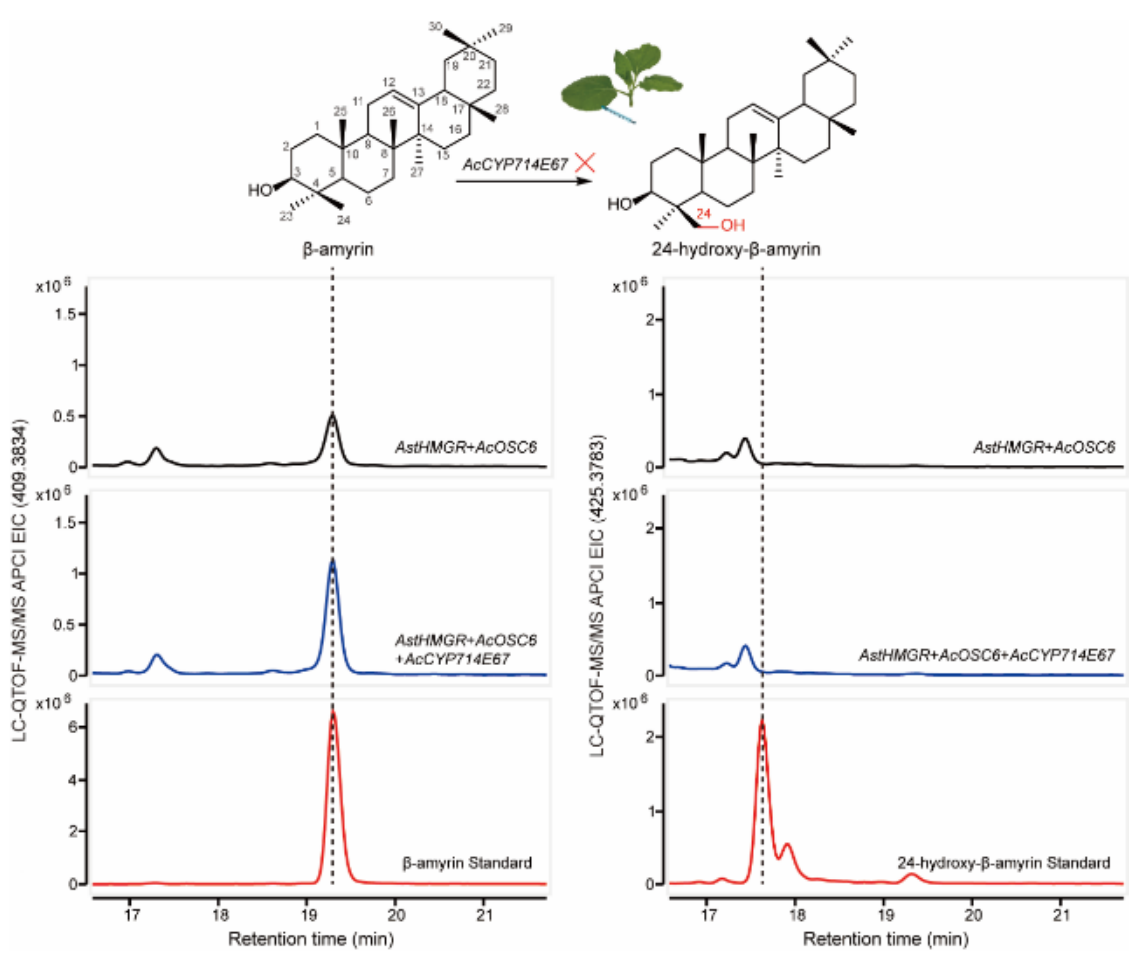

Figure S29. Co-infiltration of *AstHMGR*, *AcOSC6* and *AcCYP714E67* into *N. benthamiana*. This co-injection only yielded β-amyrin and no 24-hydroxy-β-amyrin was detected. These results indicate *AcCYP714E67* cannot catalyze the hydroxylation of β-amyrin at the C-24 position.

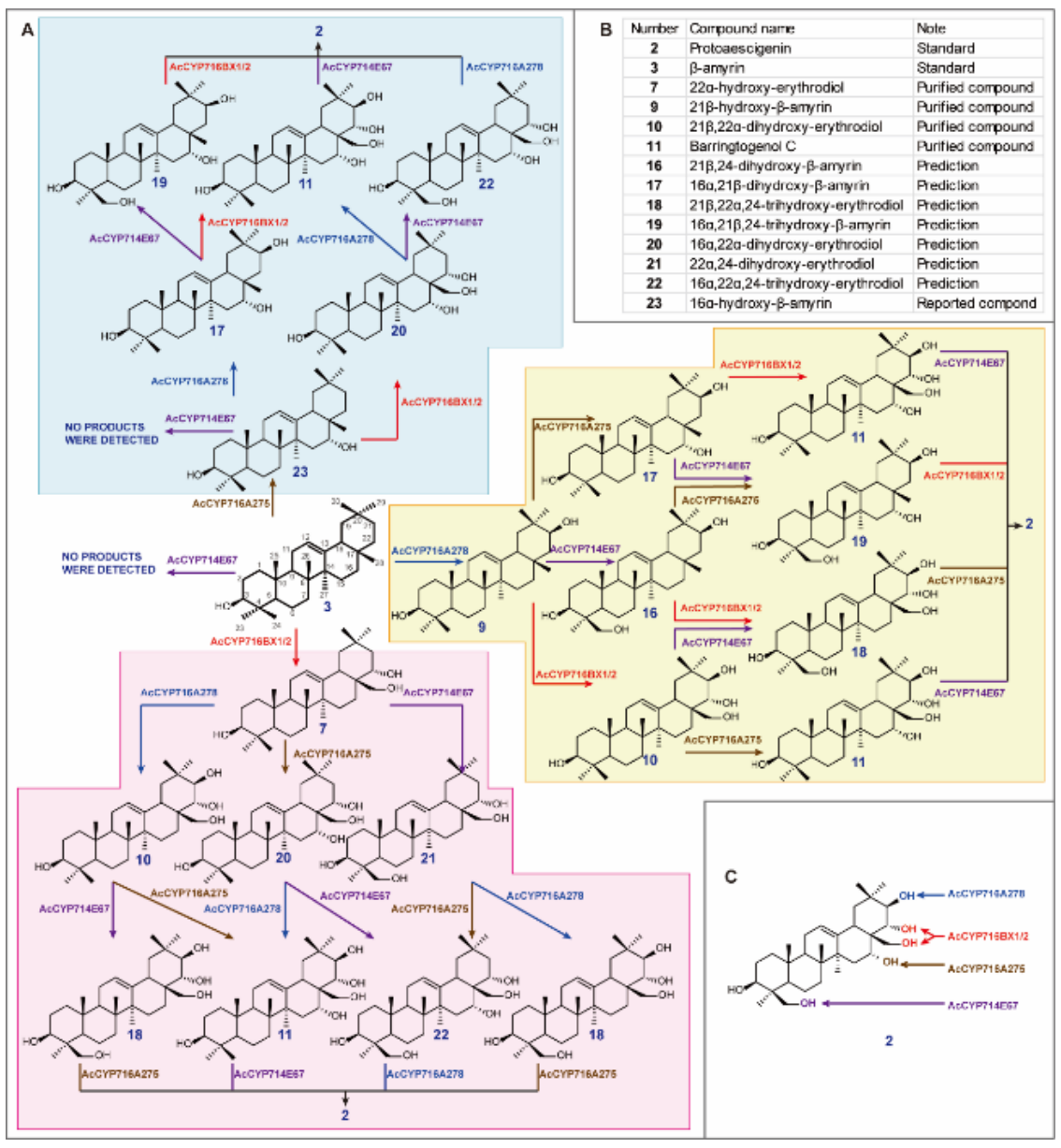

Figure S30. Complex metabolic network for the biosynthesis of protoaescigenin (**2**) from β-amyrin (**3**). (A) Schematic diagram of the metabolic network; (B) Related intermediates; (C) Schematic diagram of hydroxylation sites catalyzed by CYP450s.

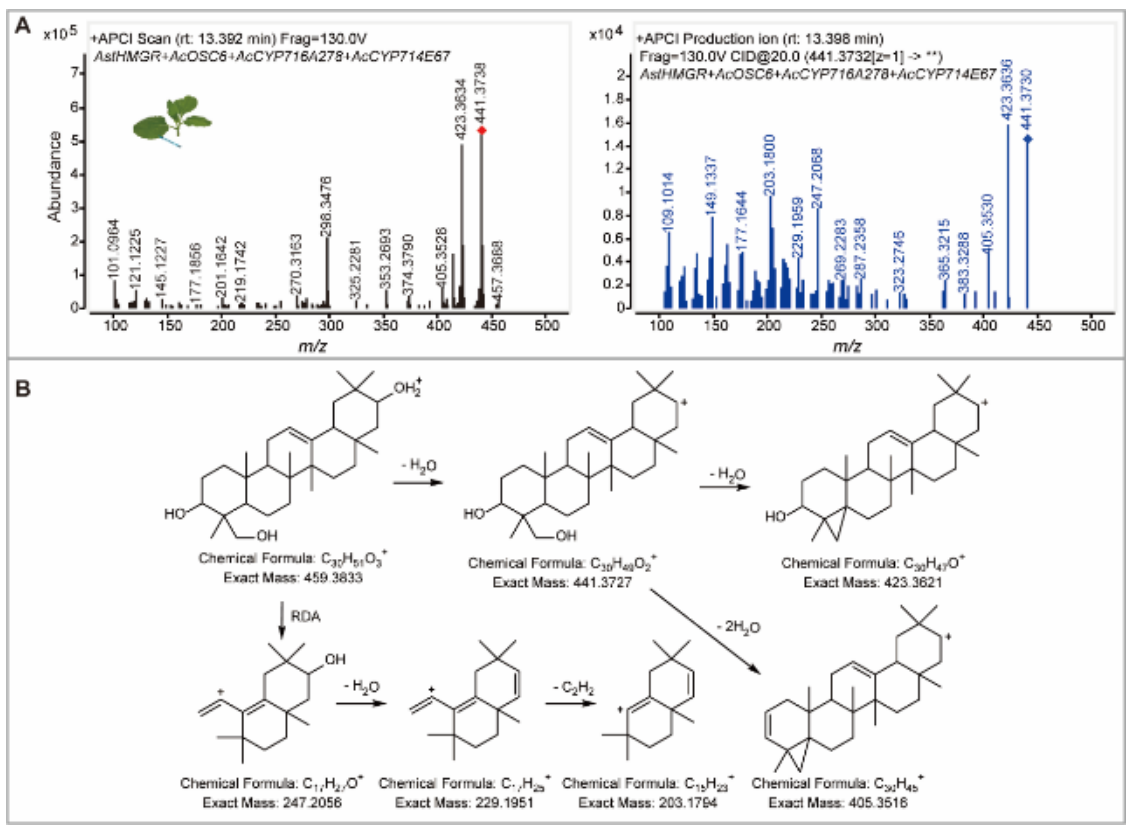

Figure S31. Mass spectra (A) and proposed fragmentation pathways (B) of 21β,24-dihydroxy-β-amyrin (**16**) produced in infiltrated *N. benthamiana*.

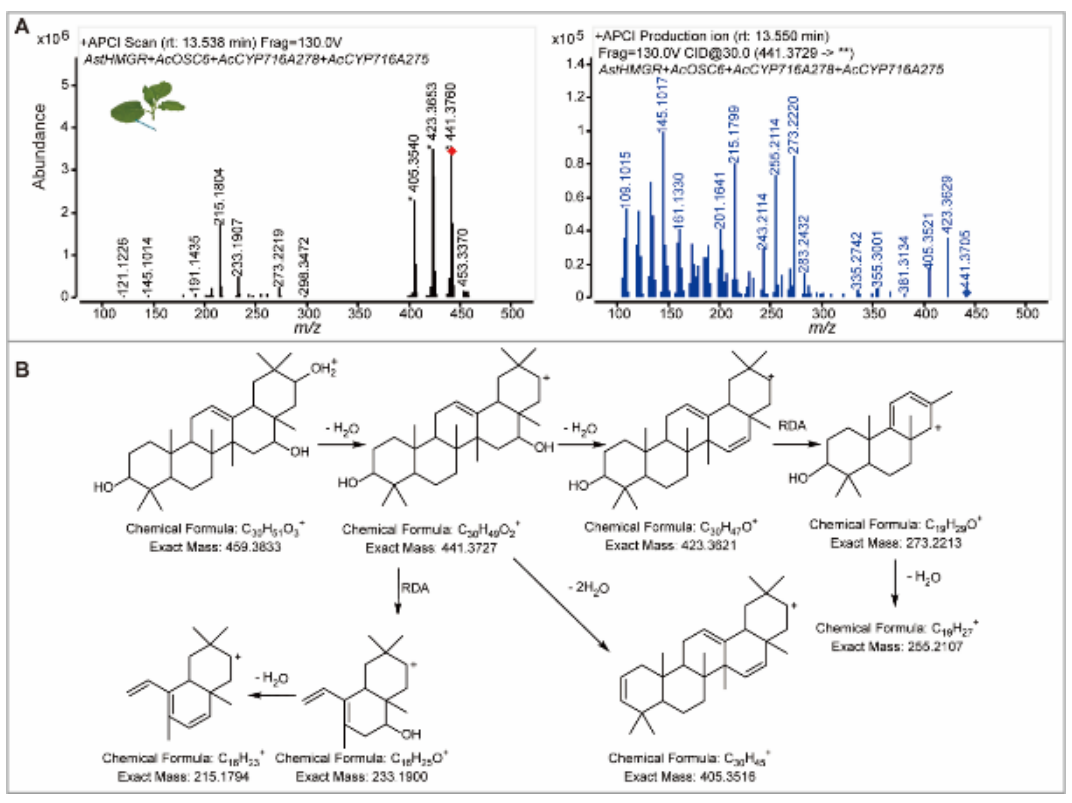

Figure S32. Mass spectra (A) and proposed fragmentation pathways (B) of 16α,21β-dihydroxy-β-amyrin (**17**) produced in infiltrated *N. benthamiana*.

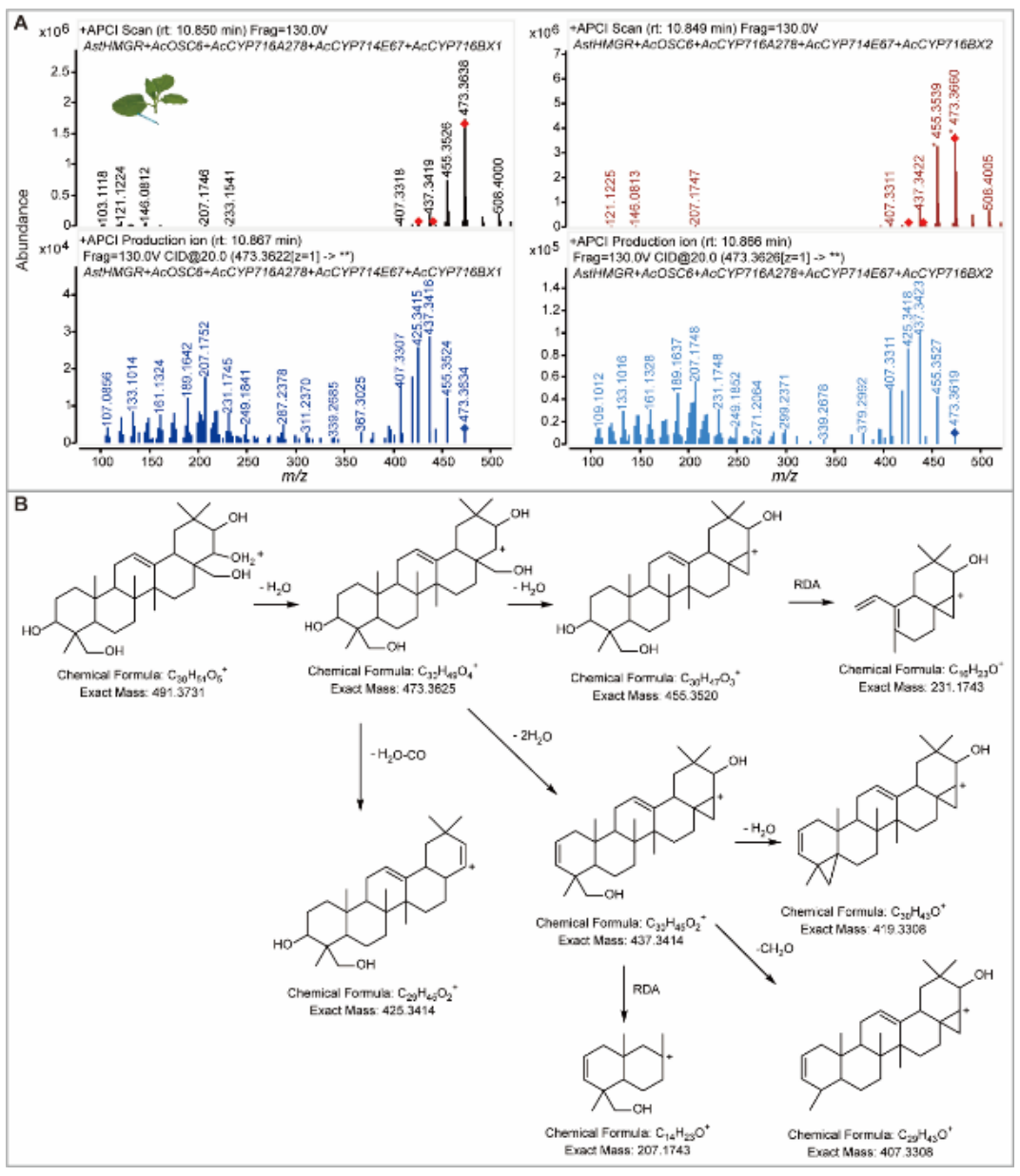

Figure S33. Mass spectra (A) and proposed fragmentation pathways (B) of 21β,22α,24-trihydroxy-erythrodiol (**18**) produced in infiltrated *N. benthamiana*.

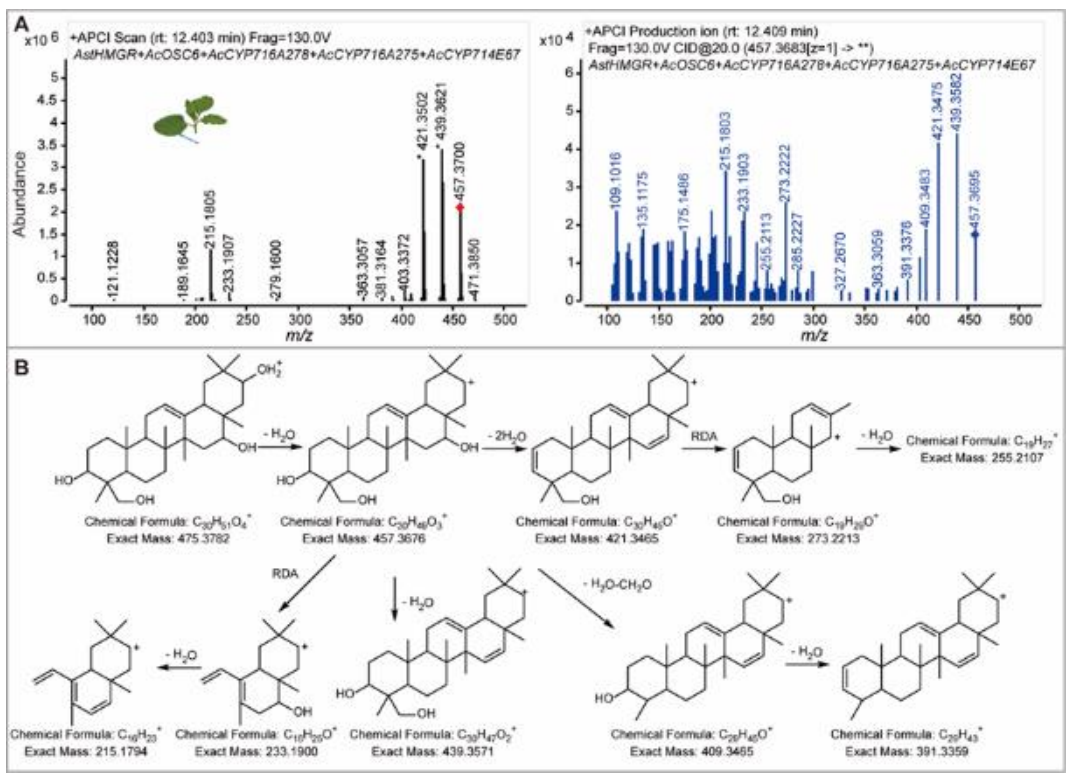

Figure S34. Mass spectra (A) and proposed fragmentation pathways (B) of 16α,21β,24-trihydroxy-β-amyrin (**19**) produced in infiltrated *N. benthamiana*.

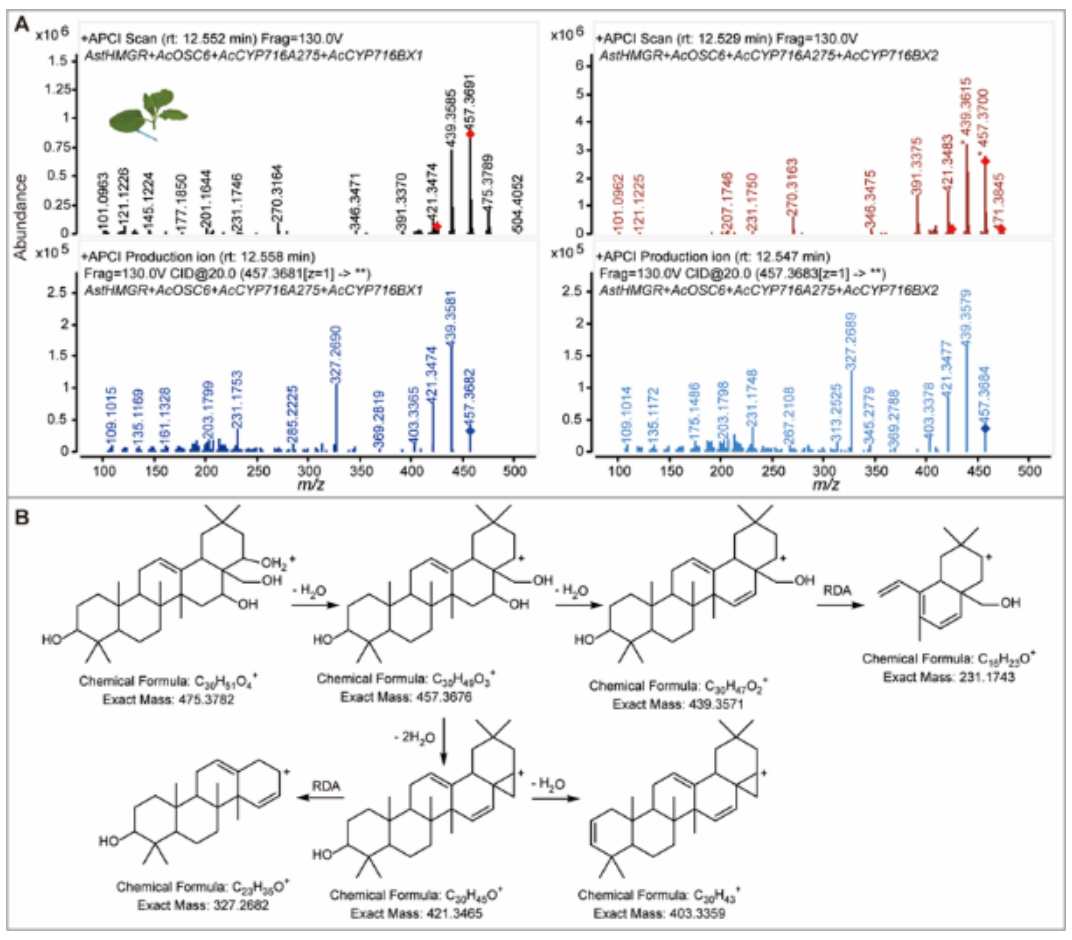

Figure S35. Mass spectra (A) and proposed fragmentation pathways (B) of 16α,22α-dihydroxy-erythrodiol (**20**) produced in infiltrated *N. benthamiana*.

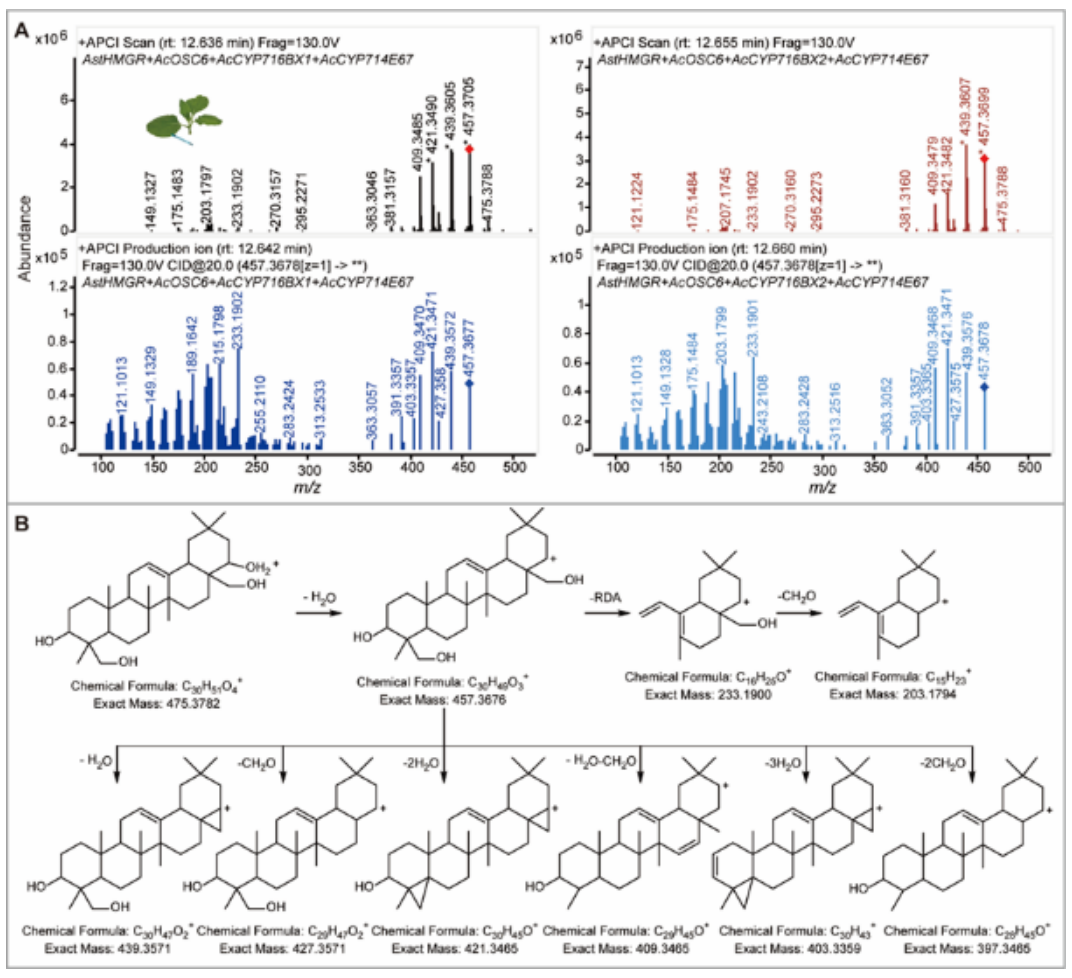

Figure S36. Mass spectra (A) and proposed fragmentation pathways (B) of 22α,24-dihydroxy-erythrodiol (**21**) produced in infiltrated *N. benthamiana*.

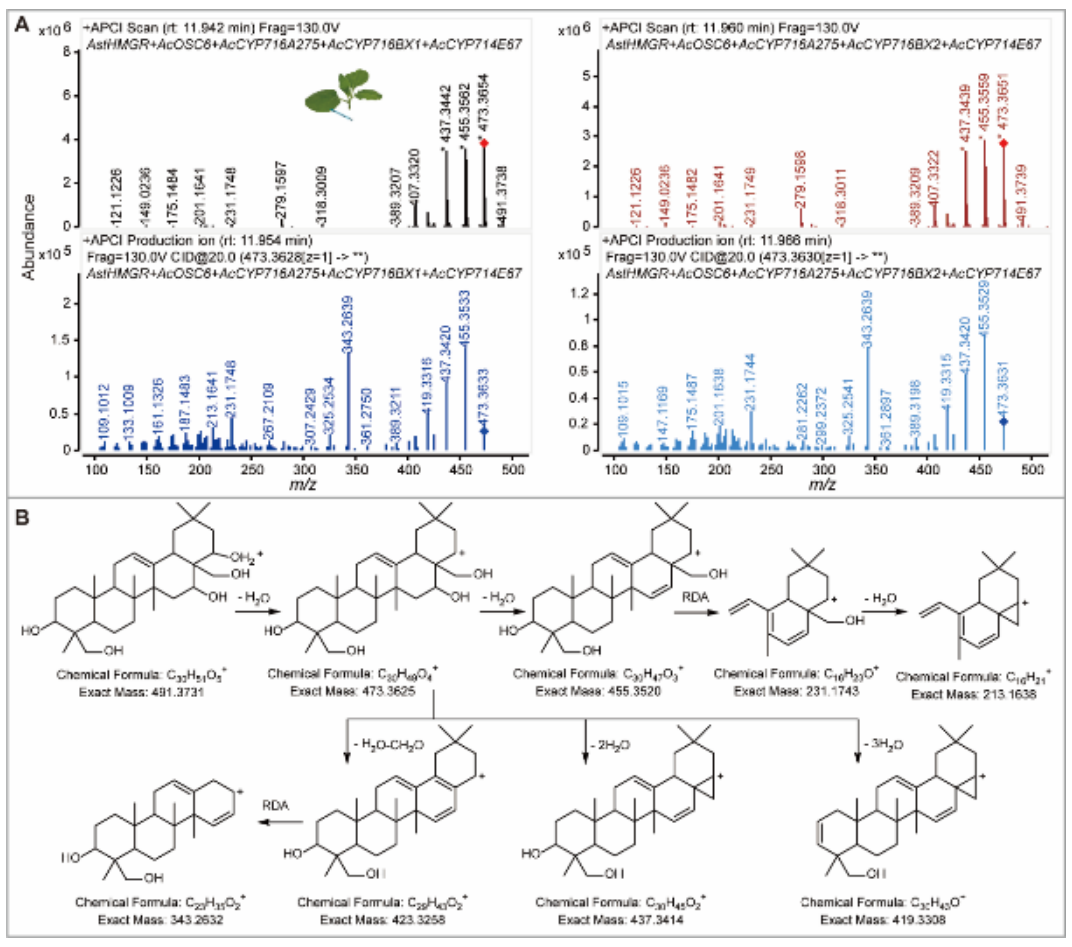

Figure S37. Mass spectra (A) and proposed fragmentation pathways (B) of 16α,22α,24-trihydroxy-erythrodiol (**22**) produced in infiltrated *N. benthamiana*.

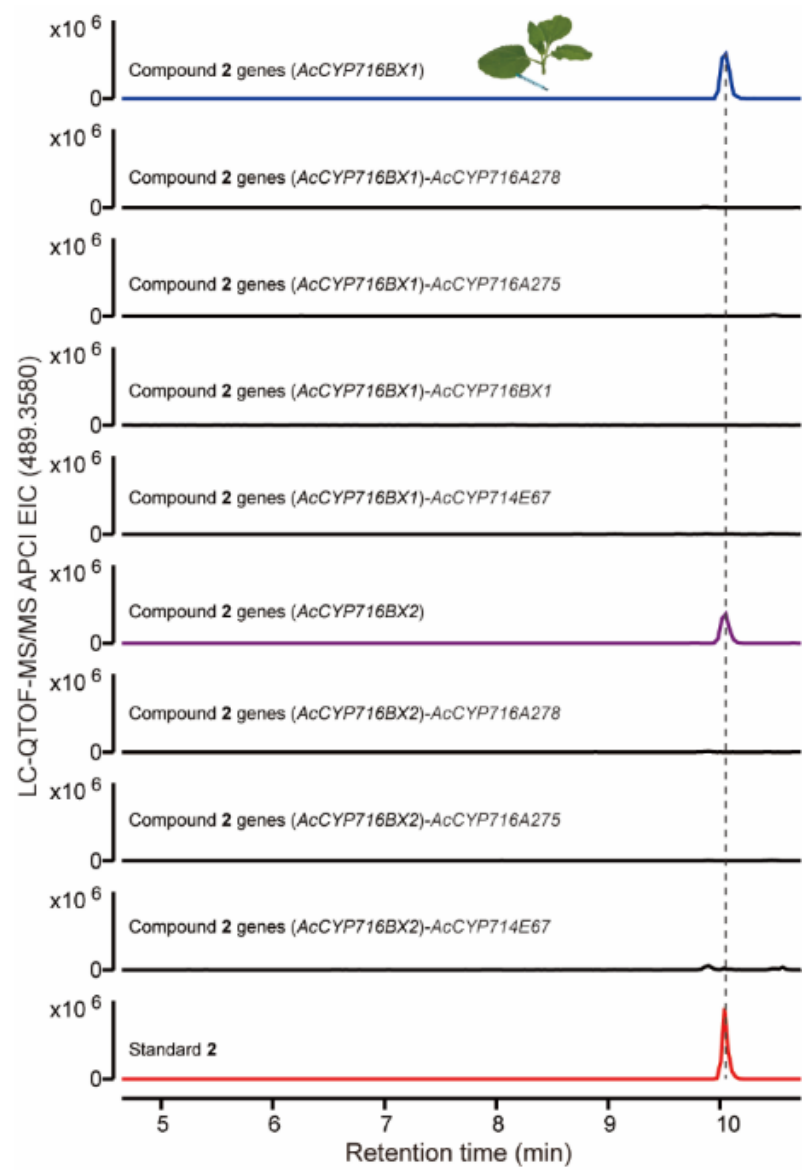

Figure S38. Elimination of interference from endogenous *N. benthamiana* CYP450s via co-expression minus-one control experiments. The full biosynthetic module for **2** included *AstHMGR*, *AcOSC6*, *AcCYP716A278*, *AcCYP716BX1/2*, *AcCYP716A275*, and *AcCYP714E67*. Individual *CYP450* genes were systematically omitted from these modules to serve as negative controls and confirm that the observed hydroxylation activities were specific to the heterologously expressed target genes.

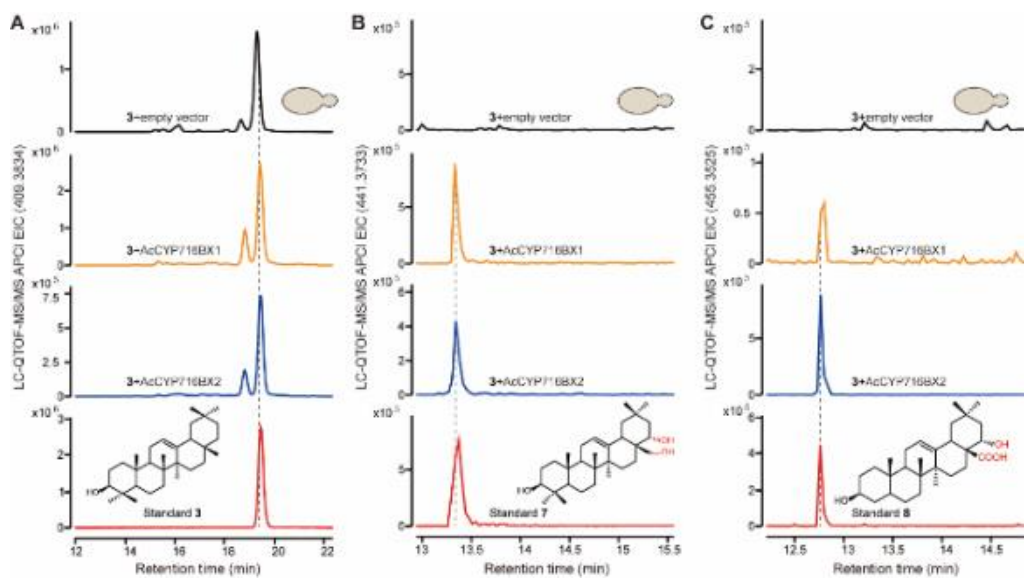

Figure S39. Functional validation of AcCYP716BX1 and AcCYP716BX2 via yeast microsomal assays. (A–C) Overlay of LC-QTOF-MS/MS extracted ion chromatograms for (A) **3** (*m/z* 409.3834), (B) **7** (*m/z* 441.3733), and (C) **8** (*m/z* 455.3525). The data represent the catalytic conversion of substrate 3 into products 7 and 8 by the respective recombinant enzymes.

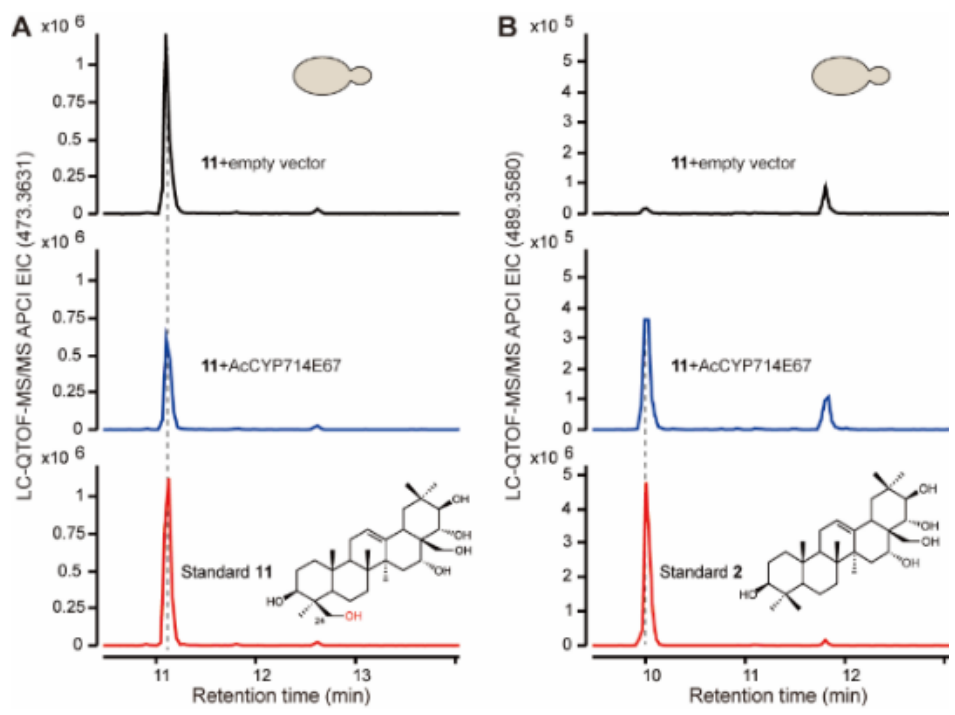

Figure S40. Functional validation of AcCYP714E67 via yeast microsomal assays. (A–B) Overlay of LC-QTOF-MS/MS extracted ion chromatograms for (A) **11** (*m/z* 473.3631), and (B) **2** (*m/z* 489.3580).

2. While the identification of C-22α hydroxylation of β-amyrin is indeed noteworthy, the assertion that "no CYP450s responsible for C-22α hydroxylation of the β-amyrin skeleton has been reported until now" is incorrect. CYP716A2 has previously been demonstrated to catalyze C-22α hydroxylation in Arabidopsis, and this prior work should be explicitly acknowledged and discussed to accurately position the present findings within the existing literature.

Response: We sincerely thank the reviewer for correcting our oversight. We apologize for the inaccurate statement in the original draft. In accordance with your valuable comment, we have thoroughly revised the text (lines 285–290) to accurately contextualize our discoveries within the established landscape of triterpenoid functionalization: "Previously, *Arabidopsis* CYP716A2 has been demonstrated to catalyze C-22α or C-28 monohydroxylation of α-amyrin and β-amyrin[50], while CYP72A61v2[51] and CYP72A566[52] are known to catalyze the C-22β hydroxylation of the β-amyrin skeleton. However, no CYP450s capable of dual C-22α and C-28 hydroxylation of the β-amyrin skeleton to form a dihydroxylated product have been reported until now. Thus, the characterization of AcCYP716BX1 and AcCYP716BX2 uncovers a distinct evolutionary and catalytic trajectory for multi-position tailoring in triterpenoid pathways."

3. The glycosylation section is one of the strongest components of the manuscript, particularly the use of DTIM-MS to distinguish regioisomeric products. However, the conclusion that 2′-O-glucosylation is strictly required before 4′-O-glucosylation is based entirely on negative in vitro results. While this hierarchical model is plausible, it would be more convincing if supported by kinetic data or a structural rationale. At present, the conclusion appears stronger than the data justify. In addition, the reported drift time difference between the two regioisomeric products is very small. The authors should address whether higher-resolution ion mobility instrumentation or an orthogonal analytical approach could be used to further validate these assignments.

Response: We sincerely appreciate the reviewer's positive evaluation of our glycosylation work and the highly constructive suggestions.

(1) To establish a rigorous structural rationale for the observed hierarchical glycosylation sequence (2′-*O*-glucosylation preceding 4′-*O*-glucosylation), we performed AlphaFold3-based structural modeling coupled with comprehensive molecular dynamics (MD) simulations. These computational insights unveiled a clear structural gating mechanism, demonstrating that the initial binding of compound **14** within the donor-acceptor pocket is thermodynamically and geometrically unfavorable, thereby explaining the strict order of tailoring steps.

(2) As the reviewer rightly pointed out, the drift time difference between the two regioisomers

in DTIM-MS was relatively narrow. To unambiguously cross-validate these assignments, we successfully achieved complete baseline chromatographic separation of the two isomers by developing a third, optimized LC gradient. In addition, we identified distinct energy-resolved tandem mass spectrometry (MS/MS) fragmentation stability profiles for each product. The manuscript has been meticulously revised as follows (with target lines updated):

Part 1: Structural Rationale via MD Simulations (lines 177–199)

"To elucidate the molecular mechanism underlying the glycosylation sequence, molecular dynamics (MD) simulations were conducted to compare the catalytic reactivity of candidate AcUGTs. Plant UGTs typically utilize S_N2-like direct displacement to catalyze glycosyl transfer[33]. A histidine (His) and an aspartic acid (Asp) near the acceptor binding pocket at the N-terminal domain of UGT have been proven to play crucial roles in the recognition and specificity of the substrate[33, 34]. Given that AcUGT94AK1, AcUGT94AK3, and AcUGT94AK6 share over 90% sequence similarity (Table S5), AcUGT94AK1 was selected as a representative model for the MD simulations. Structural models of AcUGT94AK1 and AcUGT87AZ3 were generated using AlphaFold3, and sequence alignment identified H21/D123 and H33/D126 as their respective catalytic dyads (Figure S51). Complexes of AcUGT94AK1 with **12**/**14** and AcUGT87AZ3 with **12**/**13** were subjected to docking followed by MD simulations. Although classical MD simulations cannot directly estimate reaction barriers as QM/MM methods do, productive binding conformations defined by the proximity and stability between the acceptor hydroxyl group and the catalytic His residue are widely used in parallel to evaluate UGT catalytic competence and regioselectivity[35-39]. Notably, in the AcUGT94AK1-**14** complex, ligand **14** exhibited pronounced fluctuations and progressively drifted toward the solvent-exposed region, indicating failure to maintain a productive binding mode within the acceptor pocket, consistent with an unfavorable binding free energy (ΔG_{bind} = +5.98 ± 8.23 kcal/mol) (Figure 3I and 3J, Figure S52). Consequently, the O–N distance between the C2′–OH oxygen of **14** and the His ε-nitrogen in the AcUGT94AK1-**14** complex remained above 10 Å throughout the simulation (Figure S53). These MD simulation results are consistent with the *in vitro* validations, collectively demonstrating that AcUGT94AK1 cannot catalyze the 2'-*O*-glucosylation of **14**. In contrast, the AcUGT94AK1-**12** complex adopted a stable binding state with favorable affinity (ΔG_{bind} = −9.46 ± 6.78 kcal/mol), in which the O-N distance between the C2′-OH oxygen and the catalytic His ε-nitrogen rapidly shortened and stabilized at less than 3.5 Å. Similarly, both AcUGT87AZ3-**12** and AcUGT87AZ3-**13** complexes maintained stable conformations with catalytic O-N distances consistently below 3.5 Å, indicating productive catalytic geometries (Figure 3I and 3J, Figure S52 and S53). Together, these MD

simulations reveal that AcUGT94AK1 exhibits more stringent substrate specificity than AcUGT87AZ3, selectively catalyzing the 2′-O-glycosylation of **12**, but not **14**. Therefore, this difference in substrate specificity elucidates why 2′-O-glycosylation precedes 4′-O-glycosylation in the biosynthesis of **4**."

Part 2: Orthogonal Analytical Validation (lines 148–162)

"Using 3β-*O*-{β-D-glucuronopyranosyl acid}-protoaescigenin (**12**) as the substrate, enzymatic assays revealed that four of the eight tested AcUGTs generated a new peak with an identical *m/z* value of 843.4384 and retention time of 8.2 min, matching those of the two isomers: 3β-*O*-{[β-D-glucopyranosyl-(1→2)]-β-D-glucuronopyranosyl acid}-protoaescigenin (aeswilsaponin IC, **13**) and 3β-*O*-{β-D-glucopyranosyl-(1→4)-β-D-glucuronopyranosyl acid}-protoaescigenin (**14**) (Figure S42). While initial attempts to separate these isomers using an extended 35 min elution gradient were unsuccessful (Figure S43), they were unambiguously differentiated by MS/MS fragmentation stability, a third optimized elution gradient along with Drift Tube Ion Mobility Mass Spectrometry (DTIM-MS). Specifically, **13** showed a shorter drift time than **14** (by about 0.7 ms). In terms of fragmentation stability, **13** required higher collision energy (CE) than **14**; at CE = 60, the relative intensity of the precursor ion $[M-H]^-$ for **13** remained higher than that of its product ion $[M-H-Glc]^-$. In contrast, for **14**, the precursor ion intensity was already lower than the product ion even at a lower CE of 40. Furthermore, the retention times of **13** and **14** were completely resolved under the third elution gradient. Based on these distinct differences in drift time, fragmentation, and retention time, we confirmed that AcUGT94AK1, AcUGT94AK3, and AcUGT94AK6 catalyzed the regioselective 2'-*O*-glucosylation to form **13**, whereas AcUGT87AZ3 mediated 4'-*O*-glucosylation to yield **14** (Figure 3C-D and Figures S44–45). Functional validation through *N. benthamiana* transient co-expression assays (*AstHMGR*, *AcOSC6*, *AcCYP716A278/275/BX1*, *AcCYP714E67, AcCSL1*) with individual AcUGTs confirmed this regioselective glycosylation activity *in vivo* (Figure 3E–F and Figures S45–46)."

Table S5. Sequence similarity of four AcUGT enzymes

Protein Name	AcUGT94AK1	AcUGT94AK3	AcUGT94AK6	AcUGT87AZ3
AcUGT94AK1	100.00%			
AcUGT94AK3	93.50%	100.00%		
AcUGT94AK6	90.52%	92.53%	100.00%	
AcUGT87AZ3	40.21%	41.62%	43.19%	100.00%

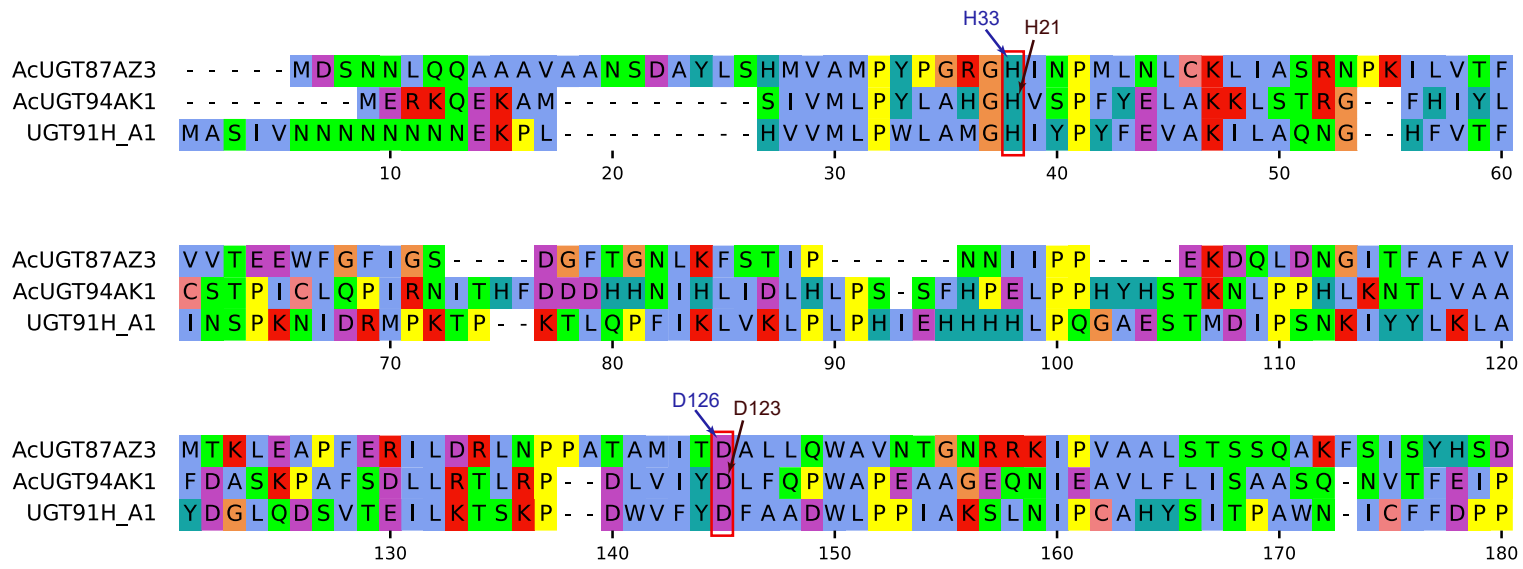

Figure S51. Sequence alignment among AcUGT94AK1, AcUGT87AZ3 and UGT91H_A1. UGT91H_A1 was previously reported by Jian et al. (2024)[2]. Key residues of the core UGT HD dyad are highlighted by red rectangles.

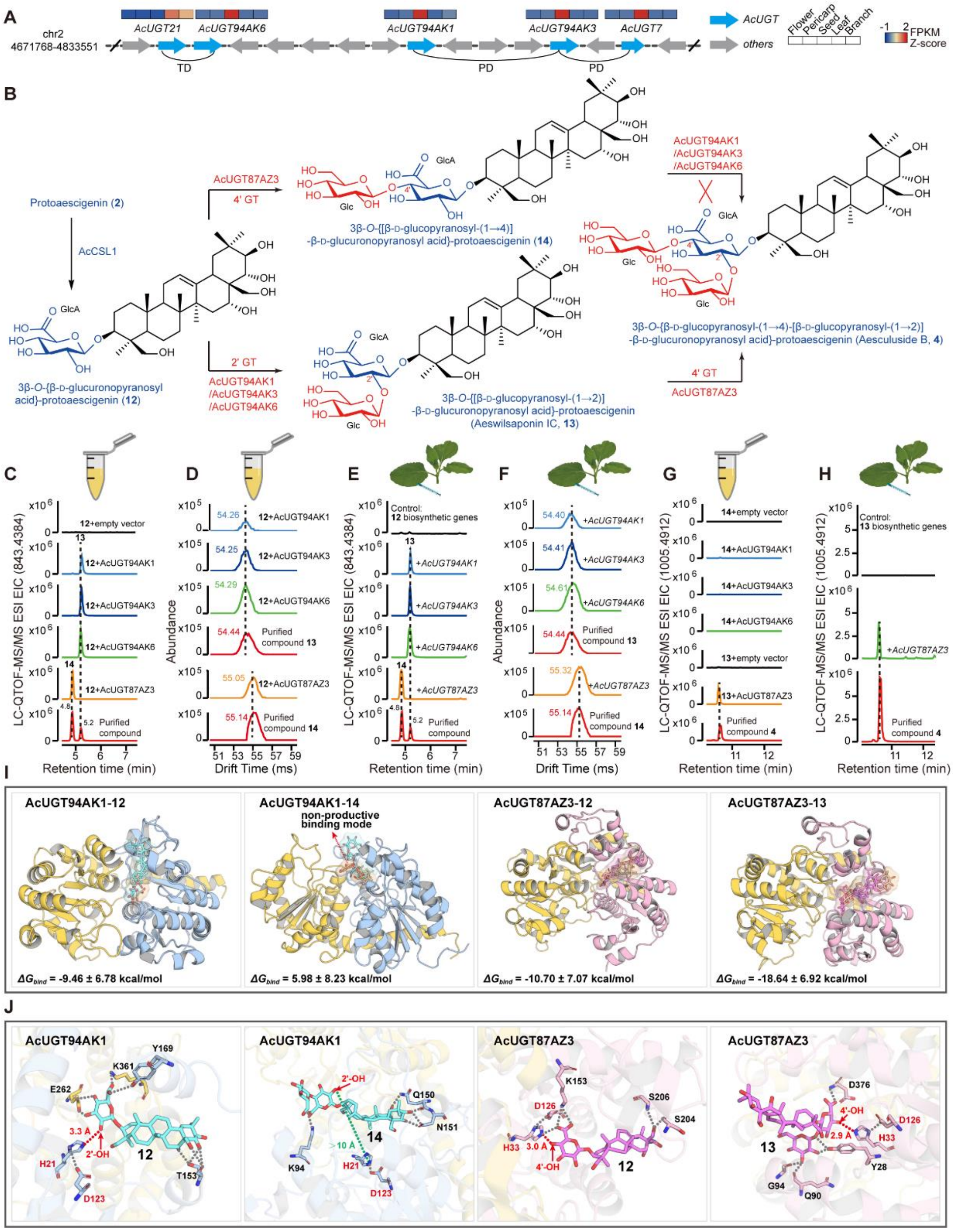

Figure 3. Genetic, biochemical, and structural basis of the branched C-3 sugar chain assembly in escin Ia (1) biosynthesis. (**A**) Genomic localization and expression profile of *AcUGTs*. *AcUGT94AK1*, *AcUGT94AK3*, *AcUGT94AK6*, and two other *AcUGTs* form a cluster on chromosome 2 and exhibit seed-specific expression. TD: tandem duplication; PD: proximal duplication. (**B**) Biosynthetic pathway from **2** to **4**. The "×" symbol indicates that no catalytic activity was detected. (**C**) *In vitro* assays showed that **12** was catalyzed by AcUGT94AK1/3/6 and AcUGT87AZ3 to generate a new product, which was identified as **13** (*m/z* 843.4384) and **14** (*m/z* 843.4384), respectively. (**D**) Isomers **13** and **14** were further distinguished by DTIM-MS analysis. This analysis confirmed that, *in vitro*, AcUGT94AK1/3/6 catalyzed the glycosylation of **12** at the 2'-OH position, while AcUGT87AZ3 catalyzed the glycosylation of **12** at the 4'-OH position. (**E**, **F**) In infiltrated *N. benthamiana*, co-expression of genes involved in **12** biosynthesis (*AstHMGR, AcOSC6, AcCYP716A278, AcCYP716BX1, AcCYP716A275, AcCYP714E67, AcCSL1*) with either *AcUGT94AK1*, *AcUGT94AK3* or *AcUGT94AK6* resulted in the production of **13**, while co-expression of these seven genes with *AcUGT87AZ3* led to the production of **14**. (**G**) *In vitro* assays revealed that AcUGT94AK1, AcUGT94AK3 or AcUGT94AK6 did not catalyze **14** to produce **4**, while AcUGT87AZ3 catalyzed **13** to generate **4**. (**H**) Co-expression of eight genes involved in compound **13** biosynthesis (*AstHMGR, AcOSC6, AcCYP716A278, AcCYP716BX1,*

AcCYP716A275, AcCYP714E67, AcCSL1, AcUGT94AK1) with *AcUGT87AZ3* resulted in the production of **4** in infiltrated *N. benthamiana***.** (**I**) Overlay of the initial docking poses and MD-equilibrated conformations for the AcUGT94AK1-**12,** AcUGT94AK1-**14,** AcUGT87AZ3-**12** and AcUGT87AZ3-**13** complexes with calculated binding energies. In AcUGT94AK1 and AcUGT87AZ3, the C-terminal and N-terminal domains are colored yellow and blue/pink, respectively. ΔG_{bind} values were calculated using the MM/GBSA method based on 200 ns MD trajectories. (**J**) Close-up views of the catalytic center. Red and green dotted lines indicate the distances between the acceptor hydroxyl group and the catalytic histidine residue. The biosynthesis steps of **4** were carried out with three biological replicates, both *in vitro* and *in vivo*.

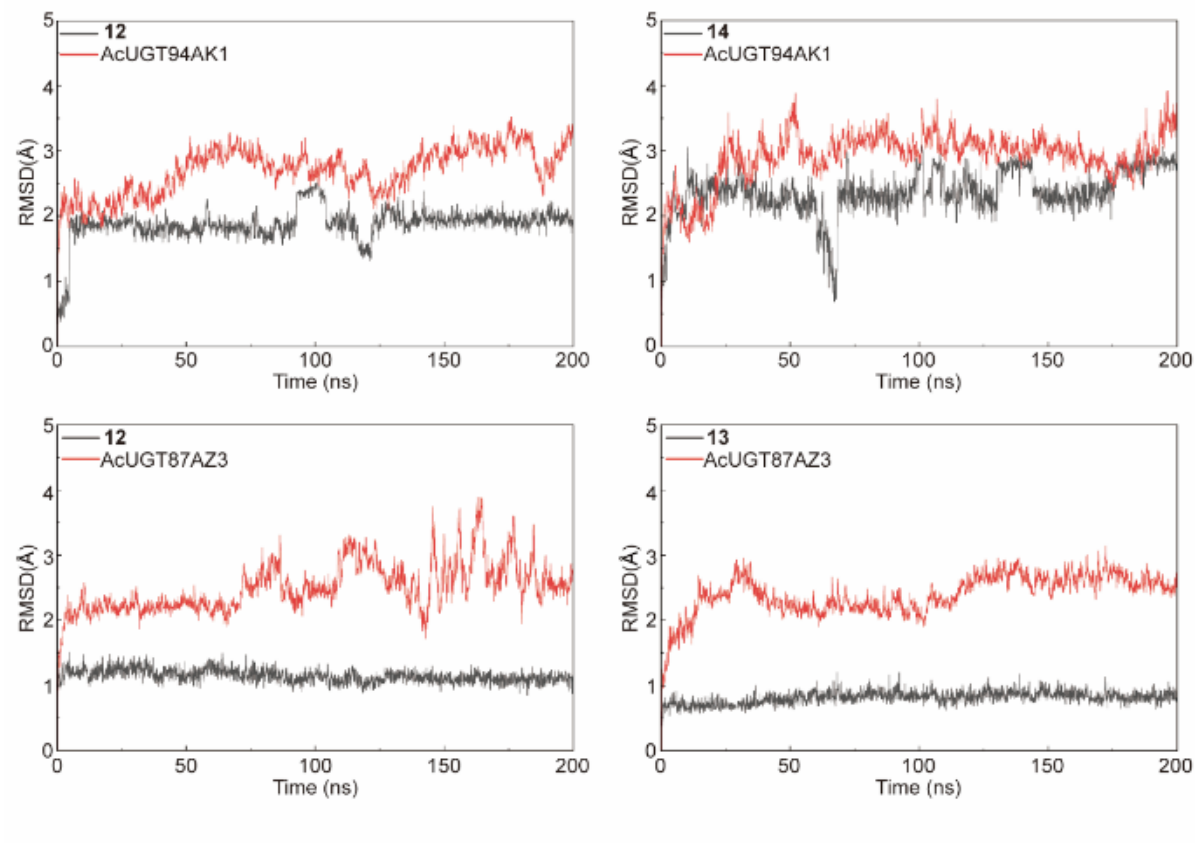

Figure S52. Root-mean-square deviation (RMSD) profiles of AcUGT94AK1 and AcUGT87AZ3 in the complexes with various ligands during 200 ns MD simulations.

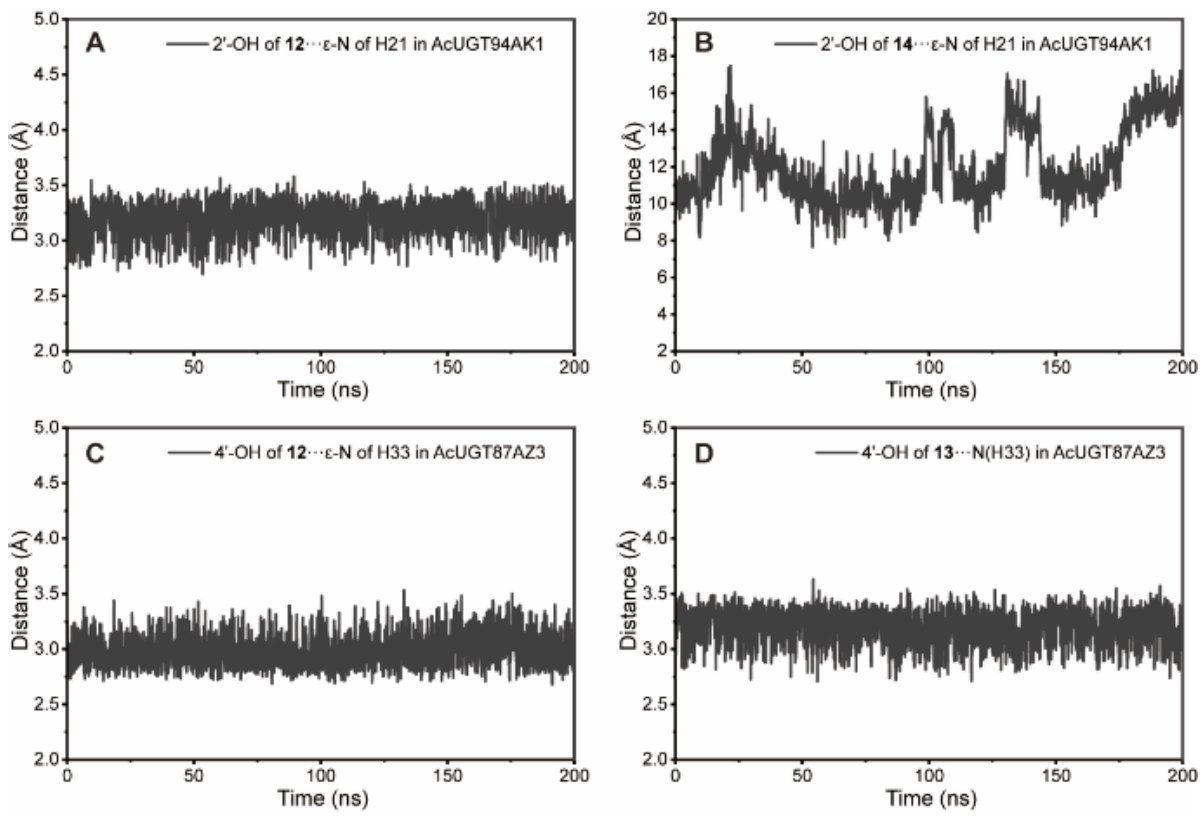

Figure S53. The O–N distance between the C2′–OH oxygen of the acceptor molecules and the His ε-nitrogen in the complexes of AcUGT94AK1-**12** (A), AcUGT94AK1-**14** (B), AcUGT87AZ3-**12** (C), and AcUGT87AZ3-**13** (D), respectively.

4. The heterologous reconstruction of escin Ia in N. benthamiana is an important validation of pathway completeness, but the reported yields are low and the manuscript does not sufficiently address this limitation. Given the applied motivation emphasized throughout the abstract and discussion, the authors should comment more directly on potential metabolic bottlenecks, compound toxicity, or flux constraints. Even a qualitative comparison with yields reported for other complex triterpenoid saponins would help place these results in a more realistic applied context.

Response: We sincerely thank the reviewer for this insightful comment. We agree that a critical evaluation of metabolic flux and potential bottlenecks is essential for placing our heterologous reconstruction efforts in a realistic applied context.

To address this, we have expanded our analysis of metabolic flux within *N. benthamiana*. As requested, we provide a qualitative and quantitative comparison with other triterpenoid saponins produced in *N. benthamiana* and have performed a diagnostic analysis of the metabolic bottlenecks. The relevant data and discussion have been incorporated into the revised manuscript.

The relevant **results** have been descried in lines 259-266: "Furthermore, we evaluated the levels of **1** and key intermediate metabolites across various gene combinations in *N. benthamiana*. Initially, the content of **12** was higher when expressing *AcCYP716BX1* compared to *AcCYP716BX2* (2,487.37 vs. 2,140.72 µg/g, respectively). Using the *AcCYP716BX1*-based combination to further stack *AcUGT*s, we found that the yield of **4** peaked at 6,123.06 µg/g in the group IV (harboring *AcUGT94AK3*), which was significantly higher than that in group V (*AcUGT94AK6*) and III (*AcUGT94AK1*) ($P < 0.05$). Among the **1**-producing groups, the accumulation of **1** reached a maximum of 122.55 µg/g in group X (containing *AcUGT94AK3* and *AcBAHD17*), significantly outperforming all other combinations ($P < 0.05$). This was followed by groups VII, XI, IX, VI, and VIII, yielding 67.10, 64.31, 35.28, 32.57, and 31.64 µg/g, respectively (Figure S59)."

The relevant **discussion** has been descried in lines 341-358: "The production of **1** (122.55 µg/g DW) achieved in this study is competitive compared to the heterologous synthesis of other triterpenoids in *N. benthamiana*, such as QS-7 (7.9 mg/g DW)[20], QS-21 (8.6 µg/g DW)[42], and asiaticoside (97.4 µg/g DW)[68],. However, metabolic flux analysis reveals a significant bottleneck at the terminal acylation steps. Indeed, the levels of the aglycone and various glycosylated intermediates, including **2**, **4**, and **12**, consistently reached mg/g scales. Particularly, intermediate **4** accumulated to a maximum of 6,123.06 µg/g. In comparision, the yield of the final product (**1**) remained relatively low. Overcoming this disparity will require multi-faceted metabolic engineering strategies. At the enzymatic level, this includes increasing the supply of acetyl-CoA and tigloyl-CoA

donors, enhancing the catalytic efficiency of BAHDs through rational design or directed evolution. At the system level, pathway optimization, flux rewiring, transcriptional regulation, and spatial organization hold great promise for further elevating the production of compound 1, as evidenced by several successful precedents. For instance, systematic optimization of the *N. benthamiana* expression system enhanced rotenoid content by 5.0- to 38.6-fold, reaching 0.2–0.6 mg/g DW[69]. Similarly, "rewiring" diterpene biosynthesis from the plastidial MEP pathway to the high-flux cytosolic MVA pathway increased the content of forskolin and related compounds approximately 10-fold[70]. Furthermore, through the systematic reconstruction of the upstream isoflavone pathway, coupled with the utilization of transcription factors to upregulate target genes while suppressing competing branches, researchers achieved yields of 7.04 g/kg DW for daidzein and 11.85 g/kg DW for genistein[71]. Regarding spatial organization, MCD1 (miR1919-targeted cell death-factor-1) from Solanaceae plants was recently shown to act as a metabolic organizer, assembling three sequential enzymes into a complex to promote the biosynthesis of debneyol[72]. Combining these sophisticated strategies in our established *N. benthamiana* platform will pave the way for the scalable production of **1**."

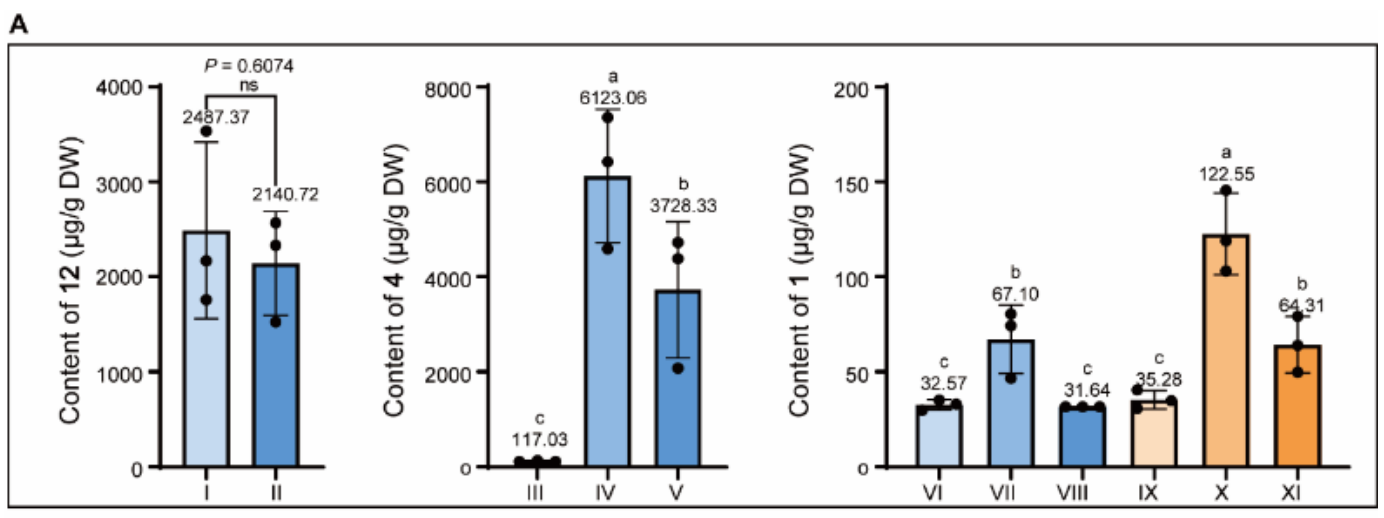

B

Name	Specific genes
I	*AstHMGR, AcOSC6, AcCYP716A278, AcCYP716BX1, AcCYP716A275, AcCYP714E67, AcCSL1*
II	*AstHMGR, AcOSC6, AcCYP716A278, AcCYP716BX2, AcCYP716A275, AcCYP714E67, AcCSL1*
III	*AstHMGR, AcOSC6, AcCYP716A278, AcCYP716BX1, AcCYP716A275, AcCYP714E67, AcCSL1, AcUGT94AK1, AcUGT87AZ3*
IV	*AstHMGR, AcOSC6, AcCYP716A278, AcCYP716BX1, AcCYP716A275, AcCYP714E67, AcCSL1, AcUGT94AK3, AcUGT87AZ4*
V	*AstHMGR, AcOSC6, AcCYP716A278, AcCYP716BX1, AcCYP716A275, AcCYP714E67, AcCSL1, AcUGT94AK6, AcUGT87AZ5*
VI	*AstHMGR, AcOSC6, AcCYP716A278, AcCYP716BX1, AcCYP716A275, AcCYP714E67, AcCSL1, AcUGT94AK1, AcUGT87AZ3, AcCCL3, AcBAHD6, AcBAHD13*
VII	*AstHMGR, AcOSC6, AcCYP716A278, AcCYP716BX1, AcCYP716A275, AcCYP714E67, AcCSL1, AcUGT94AK3, AcUGT87AZ3, AcCCL3, AcBAHD6, AcBAHD13*
VIII	*AstHMGR, AcOSC6, AcCYP716A278, AcCYP716BX1, AcCYP716A275, AcCYP714E67, AcCSL1, AcUGT94AK6, AcUGT87AZ3, AcCCL3, AcBAHD6, AcBAHD13*
IX	*AstHMGR, AcOSC6, AcCYP716A278, AcCYP716BX1, AcCYP716A275, AcCYP714E67, AcCSL1, AcUGT94AK1, AcUGT87AZ3, AcCCL3, AcBAHD6, AcBAHD17*
X	*AstHMGR, AcOSC6, AcCYP716A278, AcCYP716BX1, AcCYP716A275, AcCYP714E67, AcCSL1, AcUGT94AK3, AcUGT87AZ3, AcCCL3, AcBAHD6, AcBAHD17*
XI	*AstHMGR, AcOSC6, AcCYP716A278, AcCYP716BX1, AcCYP716A275, AcCYP714E67, AcCSL1, AcUGT94AK6, AcUGT87AZ3, AcCCL3, AcBAHD6, AcBAHD17*

Figure S59. Quantification of **12**, **4**, and **1** in infiltrated *N. benthamiana*. (A) content of **12** in groups I and II, **4** in groups III–V, **1** in groups VI–XI. Data are presented as the means ± SD of three replicates. Bars represent standard errors of the means. For **12**, *P* value was determined using a two-tailed Student's *t*-tests. For **4** and **1**, different letters represent significant differences ($P < 0.05$) among means, as determined by one-way analysis of variance (ANOVA) followed by Duncan's multiple range test. (B) Specific gene combinations used in groups I–XI.

5. Finally, the Discussion would benefit from a dedicated paragraph addressing the possible biological roles of escin Ia and related intermediates in the plant itself. Given the extensive biosynthetic investment described, some consideration of ecological or physiological function would add valuable biological context.

Response: We sincerely appreciate the reviewer's insightful suggestion to contextualize the biological and ecological significance of escin Ia (**1**) and its biosynthetic intermediates. We agree that discussing the physiological trade-offs of this high-cost biosynthetic pathway provides essential context for the evolutionary adaptations of *Aesculus* species.

As requested, we have added a dedicated paragraph to the Discussion section (lines 359–369) to address these ecological roles: "Beyond their well-characterized pharmacological activities, triterpenoids serve as indispensable defensive metabolites evolved by plants to counteract pathogens, herbivores, and phytophagous insects[73, 74]. The biosynthesis of **1** represents a substantial metabolic investment, requiring significant carbon flux through at least 15 specialized catalytic steps. This metabolic commitment underscores the vital contribution of **1** to the ecological fitness of *Aesculus* species. For instance, aescins exhibit potent antifungal activity against *Leptosphaeria maculans*, *Microdochium nivale* and five other important fungal pathogens via a synergistic dual-mode mechanism: directly disrupting fungal membrane sterols and priming the plant immune system via salicylic acid signaling and oxidative bursts[73, 74]. Similarly, the localized accumulation of aescin in *A. pavia* leaves provides a robust barrier against leafminer infestation[76]. Furthermore, the distinct bitterness of **1** in seeds functions as a formidable deterrent to both generalist herbivores and insect pests, illustrating a specialized chemical strategy to safeguard reproductive tissues[73, 77]. Taken together, we propose that **1** and its metabolic intermediates orchestrate a multi-layered chemical defense system, conferring comprehensive protection upon *Aesculus* species against a diverse array of biotic stressors."

Minor comments

1. Throughout the manuscript, terminology alternates between "tigloylation" and "tigloylation." This should be standardized to avoid confusion, particularly for readers outside the immediate field.

Response: We appreciate the reviewer's observation. We have standardized the terminology to "tigloylation" throughout the revised manuscript to ensure consistency for all readers.

2. The Discussion occasionally reiterates background material already covered in the Introduction, especially regarding general triterpenoid biosynthesis and UGT family classification. Reducing this redundancy would sharpen the focus on the genuinely novel aspects of the work.

Response: Thank you for your valuable suggestions. We agree that the original Discussion contained redundant background information. In the revised manuscript, we have streamlined these sections by removing general summaries of triterpenoid biosynthesis and UGT classification. This revision allows the Discussion to focus more directly on the novel findings of our work.

The first paragraph of the Discussion has been revised in lines 277-290 as follows: "Advancements in sequencing technologies, improved assembly techniques, and reduced sequencing costs have significantly facilitated the elucidation of enzymatic activities involved in triterpenoid biosynthesis[47]. Co-expression analyses, coupled with the identification of BGCs, enable the rapid discovery of enzyme-encoding genes associated with these pathways[48, 49]. Notably, we identified a WGD event unique to the genus *Aesculus*, which contributed to the lineage-specific accumulation of aescins[26]. Within these clusters, *AcCYP716BX1* and its paralog *AcCYP716BX2*, both originating from a WGD event, function as isoenzymes that catalyze the oxidation of β-amyrin at the C-22 and C-28 positions to yield 22α-hydroxy-erythrodiol or 22α-hydroxy-oleanolic acid. Their functional redundancy supports the hypothesis that the *Aesculus*-specific WGD was a pivotal driver for the high-level accumulation of **1** in seeds. Several studies have demonstrated that members from the CYP716A subfamily can catalyze the sequential three-step oxidation at the C-28 position of the β-amyrin skeleton. Previously, *Arabidopsis* CYP716A2 has been demonstrated to catalyze C-22α or C-28 monohydroxylation of α-amyrin and β-amyrin[50], while CYP72A61v2[51] and CYP72A566[52] are known to catalyze the C-22β hydroxylation of the β-amyrin skeleton. However, no CYP450s capable of dual C-22α and C-28 hydroxylation of the β-amyrin skeleton to form a dihydroxylated product have been reported until now. Thus, the characterization of AcCYP716BX1 and AcCYP716BX2 uncovers a distinct evolutionary and catalytic trajectory for multi-position tailoring in triterpenoid pathways."

To reduce redundancy, paragraphs 3, 4, and 5 of the Discussion have been consolidated into one single paragraph as follows (lines 308-330): "Glycosylation is a crucial modification that enhances the stability, water solubility, and pharmacological activities of triterpenoids[63, 64]. A defining feature of **1** is its branched trisaccharide chain, composed of a glucuronic acid core with glucose units attached at the 2'-*O* and 4'-*O* positions. Elucidating the enzymes involved in this modification is of significant interest. Previously, our lab identified AcCSL1 as a 3-*O*-glucuronosyltransferase[26]. In this study, we verified that AcUGT94AK1/3/6 from group A functions as the 2'-*O*-glucosyltransferase in the biosynthesis of **1**. Interestingly, our findings demonstrate that UGTs from different groups (UGT94: group A; UGT73: group D) can exhibit convergent catalytic activities. Phylogenetic analysis suggests a common ancestry between group A and D, supporting our

hypothesis that 2'-*O*-glycosyltransferase activity was inherited from a shared ancestor and subsequently underwent independent evolution across species, resulting in significant sequence divergence while retaining ancestral function. While many UGTs have been demonstrated to catalyze triterpene glycosylation at various positions[63], 4'-*O-glucosylation* remains poorly understood. To date, only AsTG1 has been reported to catalyze this specific reaction, which is a glycosyl hydrolase (GH) rather than a UGT[63]. Thus, no UGTs have been proven to catalyze the 4'-*O*-glucosylation of triterpenoids until now. Although members of the UGT87A subfamily in *Sapindus mukorossi* were recently identified in a BGC and displayed activity toward oleanolic acid, their specific regioselectivity remained elusive[27]. Here, we report for the first time that AcUGT87AZ3 catalyzes the addition of the final 1,4- D-glucose moiety to the glucuronic acid linked at the 3β-*O* position of the barrigenol-type triterpenoid. This identifies AcUGT87AZ3 as only the second functionally characterized member of the UGT87 family in secondary metabolism, following UGT87Y1, which catalyzes the glucosylation of mandelonitrile to form prunasin[65]. These findings expand the known catalytic functions of the UGT87 family and even the J group to which it belongs. Furthermore, MD simulations and biochemical assays provided critical insights into the assembly order of the branched trisaccharide chain, 2'-*O*-glycosylation precedes 4'-*O*-glycosylation. AcUGT94AK1/3/6 exhibited higher substrate specificity, exclusively catalyzing the 2'-*O*-glucosylation of the monoglycoside. In contrast, AcUGT87AZ3 displayed broader flexibility, catalyzing 4'-*O*-glucosylation on both the monoglycoside and the diglycoside intermediates. Collectively, this study provides the first experimental evidence that AcUGT94AK1/3/6 and AcUGT87AZ3 are involved in forming the branched (1→2 and 1→4) trisaccharide chain of **1**, offering valuable insights into the complex glycosylation patterns of triterpenoids."

3. There are a number of minor grammatical and typographical issues, including inconsistent spacing, missing articles and occasional tense errors, which should be corrected during revision. While none affect the scientific content, they detract from the overall polish expected at this level.

Response: We apologize for the grammatical and typographical errors. We have carefully proofread the entire manuscript and corrected the grammatical, typographical, spacing, and tense errors you pointed out.

4. The Data Availability section clearly lists accession numbers and repositories, but it would be helpful to explicitly state whether raw LC-MS and NMR data are available, and if so, where. Given the structural claims made throughout the manuscript, transparency here is important.

Response: Thanks for your suggestion. We fully agree with the reviewer that transparency

regarding raw analytical data is essential for validating the structural claims made in this study. Accordingly, we have updated the "Data Availability" section to explicitly include the locations of these datasets in lines 534-541: "The nucleotide sequences of the functionally characterized genes in this study have been submitted to the Beijing Institute of Genomics (BIG), Chinese Academy of Sciences and assigned the following accession numbers: *AcCYP716BX1* (C_AA118697.1), *AcCYP716BX2* (C_AA118699.1), *AcCYP714E67* (C_AA118693.1), *AcUGT94AK1* (C_AA118701.1), *AcUGT94AK3* (C_AA118702.1), *AcUGT94AK6* (C_AA118700.1), *AcUGT87AZ3* (C_AA118703.1), *AcCCL3* (C_AA118698.1), *AcBAHD13* (C_AA118694.1), and *AcBAHD17* (C_AA118695.1). The updated genome and gene structures of *A. chinensis* are available at Figshare [https://doi.org/10.6084/m9.figshare.21350865]. Furthermore, the raw analytical data, including all LC-MS datasets and NMR spectra, have been deposited in the Figshare repository via the journal's integrated submission channel and will be fully accessible upon article publication. Source data are provided with this paper."

5. There is an issue with the following sentence in the Abstract: "AcCCL3 6 catalyzes tiglic acid to form tigloyl-CoA, and AcBAHD13/17 catalyze C-21β tigloylation of aesculuside B to yield desacetylescin Ia". This sentence should be corrected for clarity.

Response: Thank you for pointing this out. We apologize for the lack of clarity. The sentence has been corrected as follows: "AcCCL3 catalyzes the conversion of tiglic acid to form tigloyl-CoA, and AcBAHD13/17 catalyze the C-21β tigloylation of aesculuside B to yield desacetylescin Ia."

6. In the Introduction, the statement that saponins are "also known as triterpene glycosides" is misleading, as it excludes steroidal saponins. This should be rephrased.

Response: We thank the reviewer for this correction. The term "saponin" is indeed broader than "triterpene glycoside". We have rephrased the sentence to: "Triterpene glycosides are naturally occurring organic compounds ubiquitously distributed in plants[1]. These triterpenoids have achieved…."

7. In several places, the authors refer to the discovery of CSL enzymes involved in glucuronic acid attachment to triterpenoid aglycones but do not cite the original studies that first reported these activities. Appropriate primary references should be included.

Response: We thank the reviewer for this important reminder. We agree that the non-canonical function of cellulose synthase-like (CSL) enzymes as triterpenoid glucuronosyltransferases is a significant discovery that warrants proper citation of the primary literature. Accordingly, we have added the relevant description in "Introduction" section (lines 50-51) as follows: "CSL proteins have been demonstrated to catalyse 3-*O*-glucuronosylation of triterpenoid aglycones in several plant species, including licorice[28], spinach[29], and horse chestnut[26]."

8. The manuscript frequently refers to the "turquoise module" identified by WGCNA, but no information is provided about other modules. A brief explanation would improve clarity for readers unfamiliar with the analysis.

Response: We appreciate the suggestion to clarify our WGCNA approach. While the WGCNA analysis yielded several modules, the "turquoise" module was the only one that aligned with the seed-specific accumulation of escin Ia. Genes in other modules showed expression patterns related to other tissues (such as leaves or branches) and lacked correlation with the biosynthesis of **1**. We have revised the text to explain the module selection as follows (lines 68-71): "A seed-specific 'turquoise' module was identified as the primary candidate for escin Ia (**1**) biosynthesis, as its expression profile most closely mirrored the accumulation of **1** in seeds. Conversely, other identified modules showed peak expression in non-seed tissues and lacked significant correlation with the biosynthetic pathway. The 'turquoise' module includes AcOSC6, AcCSL1, 14 CYP450s, 14 UGTs, 5 CCLs, and 13 BAHDs."

9. In the Supplementary Dataset, several MS/MS spectra appear to be of poor quality. The authors should consider rerunning these analyses using different collision energies or higher analyte loading to improve spectral quality and confidence in fragment assignments.

Response: We thank the reviewer for this constructive suggestion. To improve the spectral quality and ensure the confidence of our fragment assignments, we have optimized the collision energies (CE). Accordingly, the MS/MS spectra for compounds **13**, **14**, and **4** have been updated in the revised Supplementary Materials (**Figures S44–S46** and **S48**).

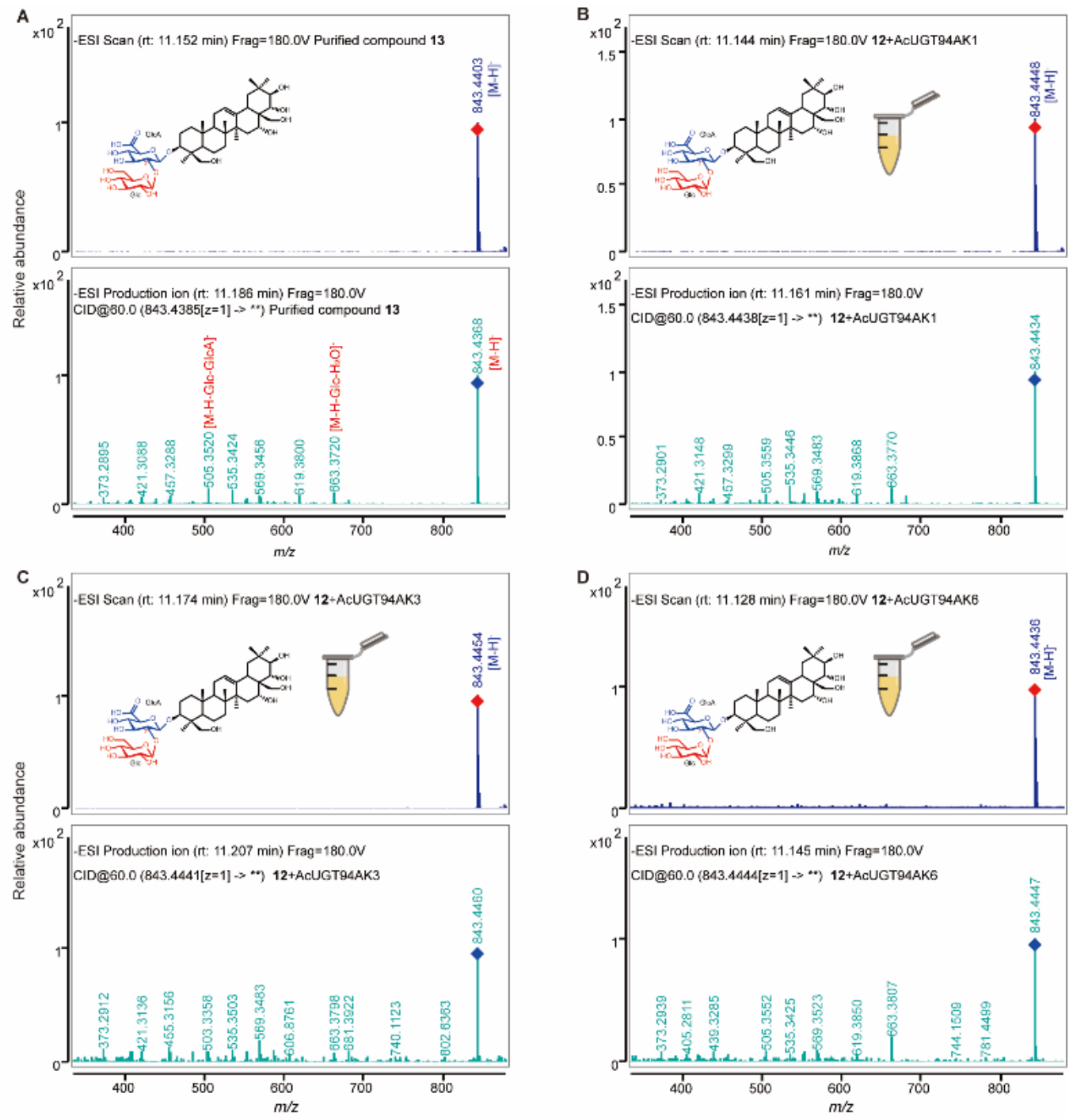

Figure S44. The MS[1] and MS[2] spectra of 3β-*O*-{[β-D-glucopyranosyl-(1→2)]-β-D-glucuronopyranosyl acid}-protoaescigenin (Aeswilsaponin IC, **13**) generated *in vitro*. (A) The purified compound **13**. **(**B, C, D) The corresponding compound generated *in vitro* by AcUGT94AK1, AcUGT94AK3 and AcUGT94AK6 using **12** as substrate.

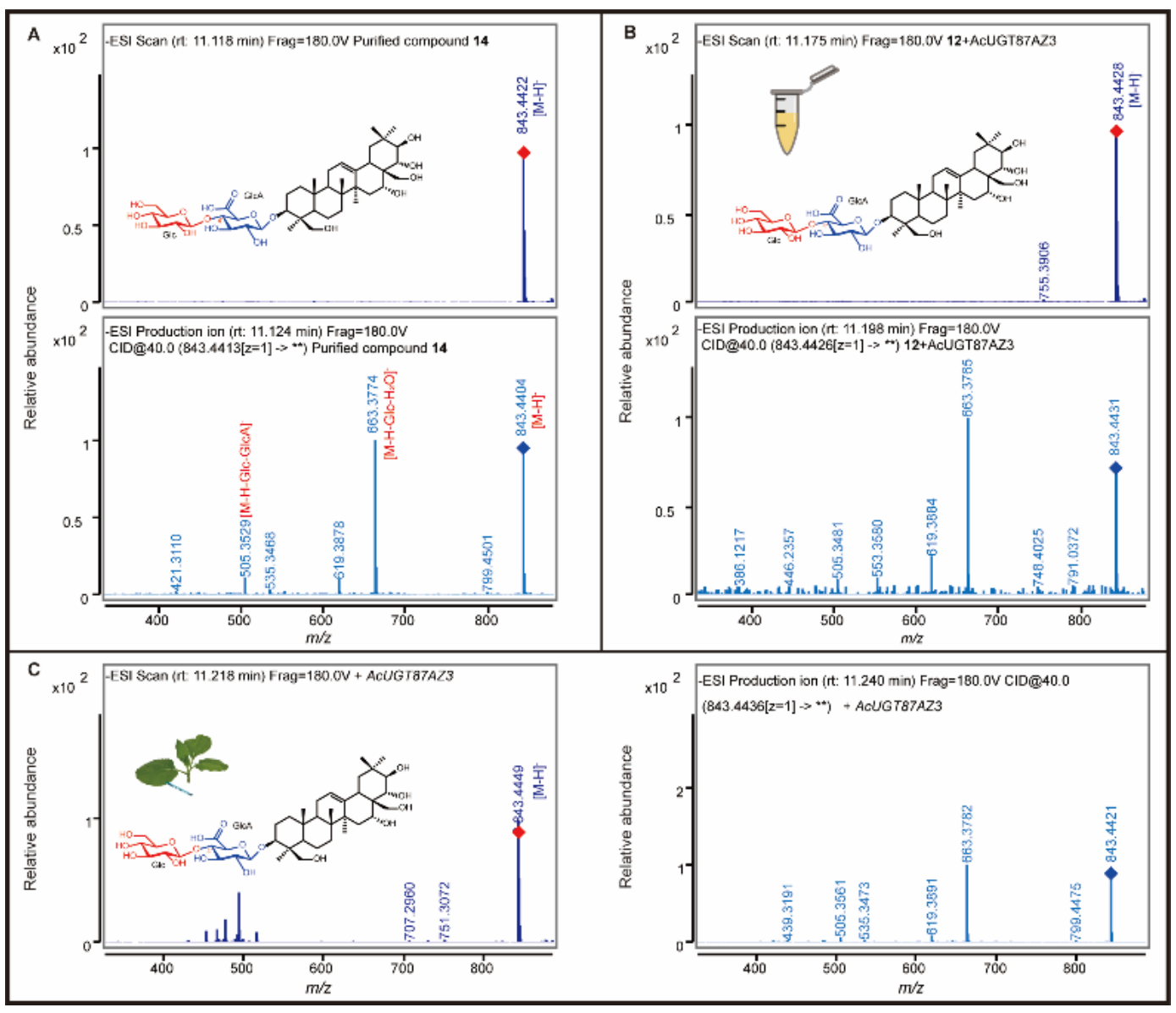

Figure S45. The MS[1] and MS[2] spectra of 3β-*O*-{[β-D-glucopyranosyl-(1→4)]-β-D-glucuronopyranosyl acid}-protoaescigenin (**14**). (A) The purified compound **14**. (B) The corresponding compound generated *in vitro* by AcUGT87AZ3 using **12** as substrate. (C) The corresponding compound yielded in *N. benthamiana* co-expressing seven genes involved in **12** biosynthesis (*AstHMGR, AcOSC6, AcCYP716A278, AcCYP716BX1, AcCYP716A275, AcCYP714E67, AcCSL1*) with *AcUGT87AZ3*.

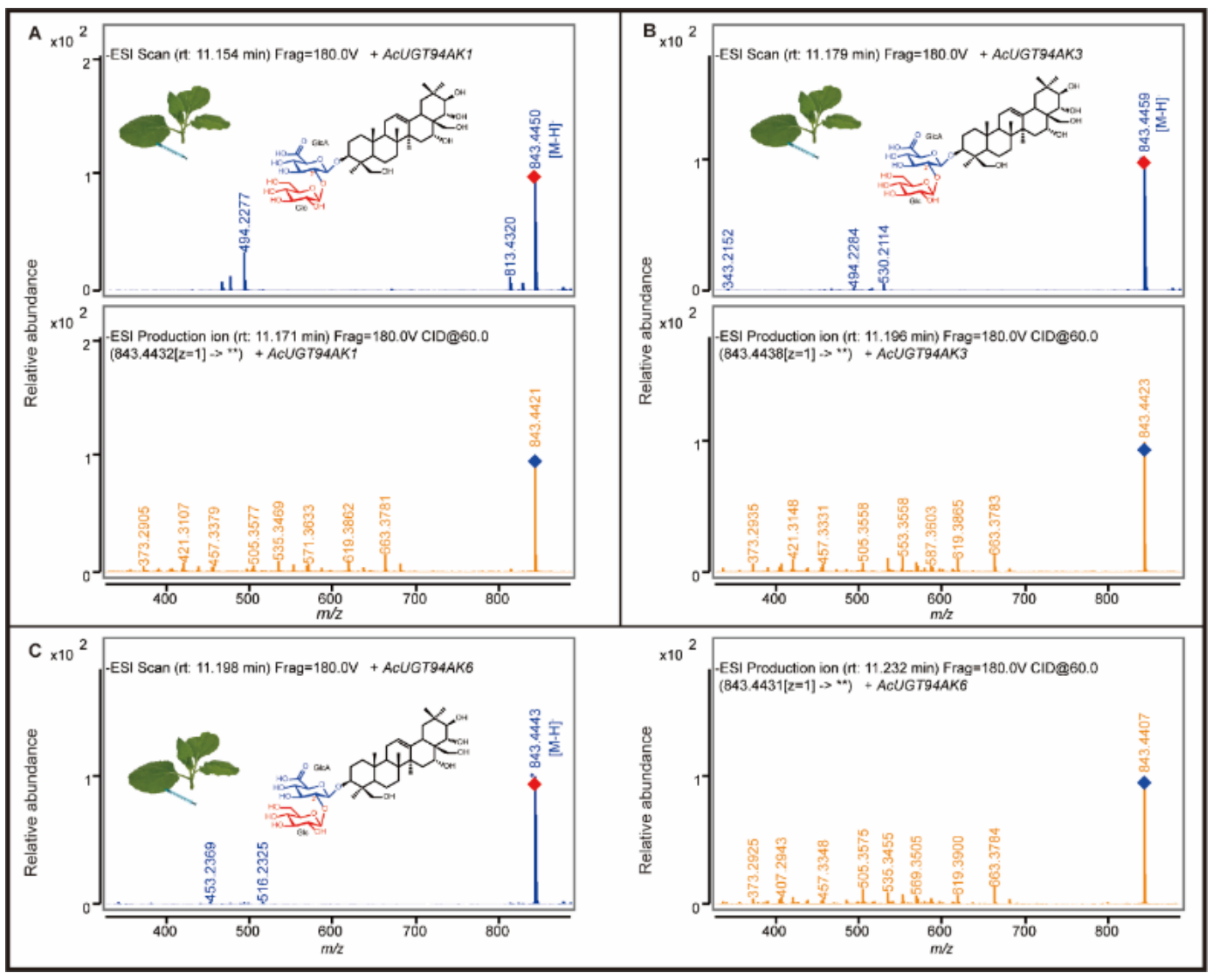

Figure S46. The MS^1 and MS^2 spectra of **13** generated in infiltrated *N. benthamiana* co-expressing seven genes involved in **12** biosynthesis (*AstHMGR, AcOSC6, AcCYP716A278, AcCYP716BX1, AcCYP716A275, AcCYP714E67, AcCSL1*) with *UGT94AK1* (A), *UGT94AK3* (B), and *UGT94AK6* (C), respectively.

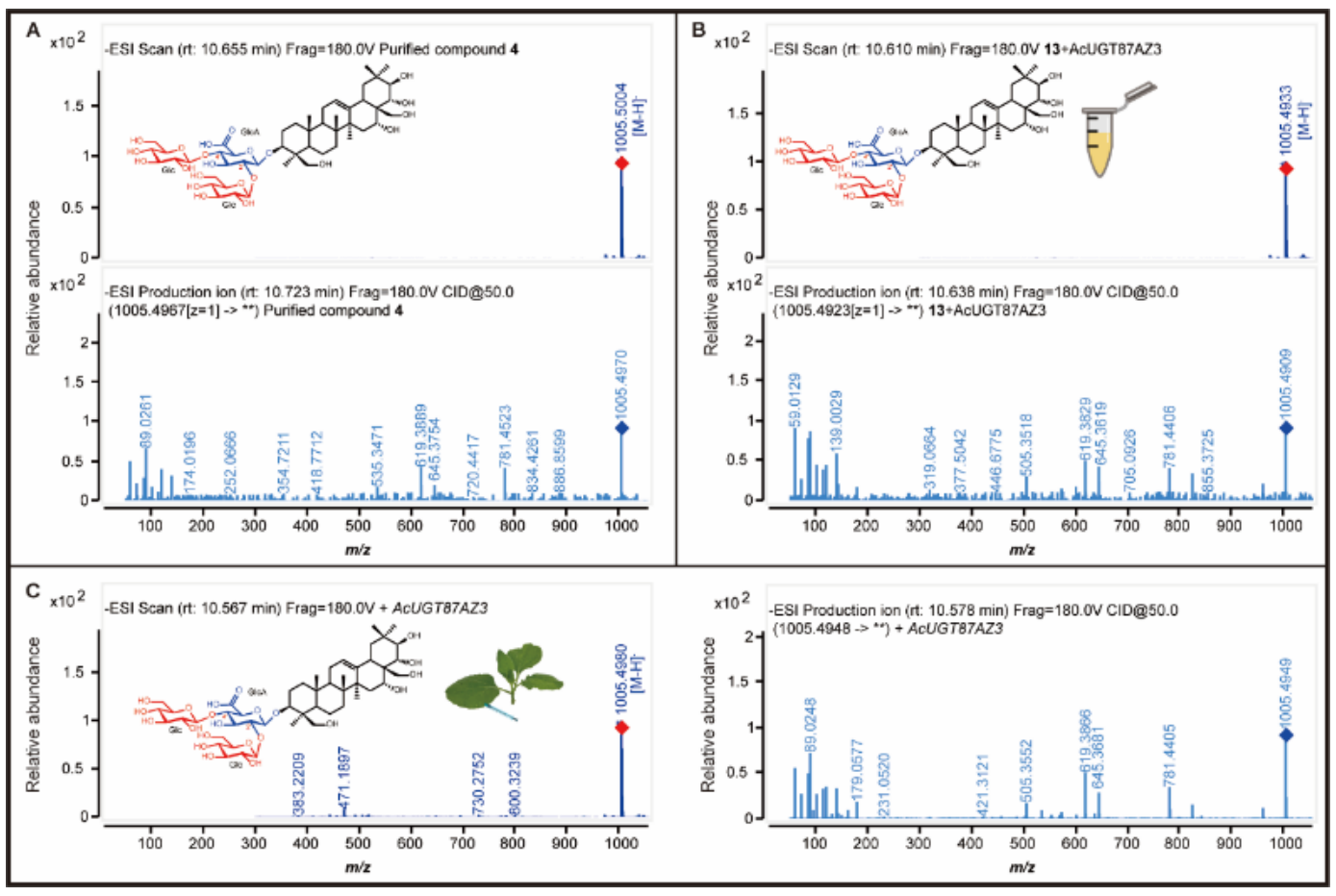

Figure S48. The MS^1 and MS^2 spectra of 3β-*O*-{β-D-glucopyranosyl-(1→4)-[β-D-glucopyranosyl-(1→2)]-β-D-glucuronopyranosyl acid}-protoaescigenin (Aesculuside B, **4**). (A) The purified compound **4**. (B) The corresponding compound generated *in vitro* by AcUGT87AZ3 using **13** as substrate. (C) The corresponding product yielded in infiltrated *N. benthamiana* co-expressing eight genes involved in **13** biosynthesis (*AstHMGR, AcOSC6, AcCYP716A278, AcCYP716BX1, AcCYP716A275, AcCYP714E67, AcCSL1, AcUGT94AK1*) with *AcUGT87AZ3*.

Reviewer #3 (Remarks to the Author):

The authors of this paper investigate the biosynthesis of Escin Ia, a pharmaceutically important triterpene saponin, and elucidate key pathway genes required. To do this they utilised a transcriptomics and weighted co-expression gene network analysis utilising 5 previously determined genes that are involved in the biosynthesis. From this they identified a module that was seed specific (matching the location of biosynthesis) and identified 43 potential biosynthesis genes. Subsequently they identify two AcCYP716 genes that can convert B-amyrin to 21B,22a-dihydroxyerythrodiol. They also identified AcCYP714E67 responsible for protoaescigenin conversion. Following this, they investigated the addition of the hexose groups to the aglycone and identified AcUGT87AZ3 which adds 4' glucose and AcUGT94AK1/3/6 which adds the 2' glucose. As the final step they then identified conclusively that BAHD13/17 can catalyse the transfer of the tigloyl group to descetylescin Ia to for Escin Ia and identified the enzyme that converts tiglic acid to tigloyl-CoA which may help with future engineering efforts for production of Escin Ia. This is an exciting and thorough piece of work with new CYPs and UGT enzyme activity described for previously uncharacterised clades and provides potential access to a very important compound.

Response: We sincerely appreciate your valuable comments and constructive suggestions on our manuscript. In response to your insightful comments, we have completed the revisions to the manuscript and provided a point-by-point response to each of your concerns.

In my view the authors clearly state their approach to elucidating the Escin Ia pathway and determine the biosynthesis of this molecule with heterologous production in N. benthamiana and in vitro (where possible). They utilise NMR techniques to conclusively confirm the production of the correct molecule and compare to authentic/purified standards throughout the manuscript.

The methods and results are clear; however, it would be helpful to the reader to provide the numbers of replicates used in each experiment within the figure legends. This data is available in the supplement, and it seems biosynthesis steps were carried out with 3 replicates, which is appropriate, but these details should be in the main body.

Response: Thank you for this constructive suggestion. We have updated the legends for **Figures 2–5** to explicitly state the number of biological replicates performed for all *in vitro* and *N. benthamiana* infiltration experiments.

Also, while the writing is clear throughout most of the manuscript, the discussion would benefit from some polishing. I have noted a few specific errors below, but the whole section should be carefully revised to improve the grammar and to remove typographical errors.

Response: We appreciate this constructive suggestion. We have carefully polished the Discussion section to correct grammatical and typographical errors. The revised text has been improved for clarity and readability, with changes marked in the revised manuscript.

• Please include the number of replicates carried out in biosynthesis for in vitro and N. benthamiana infiltration experiments in the main figure legend.

Response: Thank you for this constructive suggestion. We have updated the legends for **Figures 2–5** to explicitly state the number of biological replicates performed for all *in vitro* and *N. benthamiana* infiltration experiments.

• Genome mining, phylogenetic analysis, gene expression analysis and gene duplications identifying- please include the substitution model used and how it was selected (e.g. ModelFinder)

Response: We thank the reviewer for this valuable suggestion. In our study, the maximum likelihood phylogenetic tree was constructed using IQ-TREE, which has an integrated ModelFinder tool. The ModelFinder automatically evaluated and selected the best-fit amino acid substitution model for our dataset based on the Bayesian Information Criterion (BIC). According to the analysis, the best-fit models were identified as Q.plant+R7 for UGTs, LG+R6 for CCLs, and Q.plant+R5 for BAHDs.

We have incorporated this important detail into the revised Methods section (lines 401-404) as follows: "Phylogenetic trees were constructed with IQ-TREE (version 2.3.6) using the maximum likelihood method, with the ultrafast bootstrap approximation set to 1000 replicates. The best-fit amino acid substitution models were automatically selected by ModelFinder based on the Bayesian Information Criterion (BIC), resulting in the use of Q.plant+R7 for UGTs, LG+R6 for CCLs, and Q.plant+R5 for BAHDs. The resulting trees were visualized and annotated using ITOL v7."

• The bars indicating expression in figure 3A and figure 5A do not seem to be aligned with the genes. I am unsure if this is a formatting error or if this indicates something different – please clarify.

Response: Thank you very much for your valuable suggestion. The expression heatmaps in Figure 3A and Figure 5A show the expression patterns of each candidate gene in flower, pericarp, seed, leaf, and branch. To facilitate better understanding, we have optimized the layout of Figure 3A and Figure 5A. The revised Figure 3 and Figure 5 are displayed above and below, respectively.

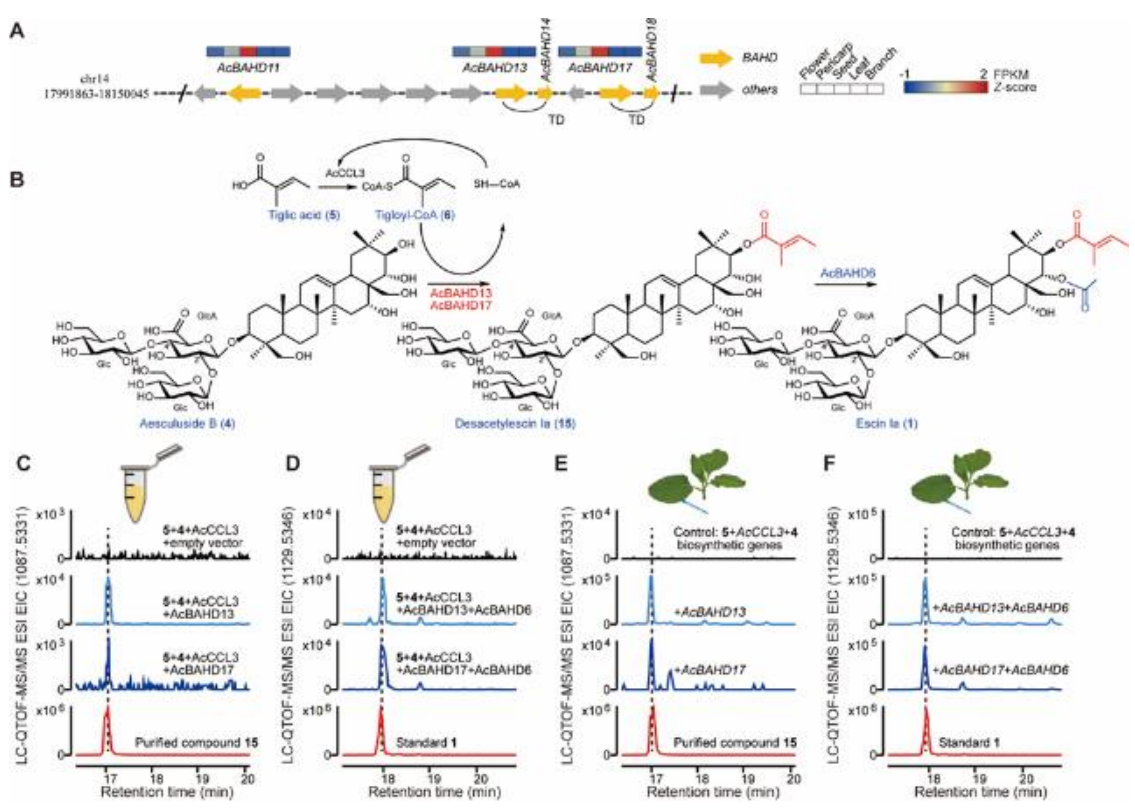

Figure 5. Elucidation of the formation of acyl side chains in escin Ia (1) biosynthesis. (**A**) Genomic organization of AcBAHDs on chromosome 14. *AcBAHD13* and *AcBAHD17*, along with three other *AcBAHDs*, form a biosynthetic gene cluster. TD: tandem duplication. (**B**) Biosynthetic pathway from **4** to **1**. (**C**) *In vitro* assays showed that AcBAHD13 or AcBAHD17, in the presence of AcCCL3 and tiglic acid (**5**), catalyzed the tigloylation of **4** to form **15**. (**D**) Further *in vitro* reconstitution showing that the addition of AcBAHD6 to the reaction mixture (AcBAHD13/17, AcCCL3, and **5**) enables the conversion of **4** to **1**. (**E**) Co-expression of either *AcBAHD13* or *AcBAHD17* with *AcCCL3*, tiglic acid (**5**) and nine genes involved in aesculuside B (**4**) biosynthesis (*AstHMGR, AcOSC6, AcCYP716A278, AcCYP716BX1, AcCYP716A275, AcCYP714E67, AcCSL1, AcUGT94AK1,* and *AcUGT87AZ3*) generated **15**. (**F**) Co-expression of *AcBAHD6* with the system of (**E**) led to the production of **1** in infiltrated *N. benthamiana*. The biosynthesis steps of **1** were carried out with three biological replicates, both *in vitro* and *in vivo*.

• It would be clearer if the Y axis for all EIC traces were on the same scale when in the same plot (e.g. within Figure 3D some show x10^4 and others x10^5)

Response: We sincerely thank the reviewer for this constructive suggestion. Accordingly, we have re-scaled all EIC traces in Figures 3D and 3F to a uniform Y-axis scale in the revised manuscript. However, Figures 5C–F are an exception. Because the peak areas of the catalytic products from different genes and the authenticated standards differ by more than an order of magnitude, we have selectively adjusted the scales for certain high-intensity standards or low-intensity samples. This ensures that their peak morphologies and retention times remain clearly visible for comparison.

• Supplemental table 2 & 4 note: “Candidate genes successed to expressing protein”- should this be successfully expressed?

Response: We apologize for this oversight. The term “successed” has been corrected to “successfully”. Accordingly, the notes of table S2 and S4 have been changed to “Candidate genes which were successfully expressed were marked as bold font”.

• Line widths and text boldness varies across figure 3C-H, it would be visually more appealing if they matched

Response: Thank you for pointing this out. We have now standardized the line widths and text boldness across all panels of Figures 3C-H to make them visually consistent. The figure has been revised accordingly.

• Figure S42 should include CYP716BX2 in the figure legend

Response: We thank the reviewer for carefully reading our manuscript and pointing out this error. Accordingly, the figure legend has been revised as: “...co-expressing 12 candidate test genes (*AstHMGR, AcOSC6, AcCYP716A278, AcCYP716BX1/2*...”

• Line 234: “functioning”

Response: We apologize for the omission. The sentence containing “functioning” was redundant with the preceding content and has been removed.

• Line 255: “variou positions”

Response: We apologize for this spelling mistake. "variou positions" has been changed to “various positions”.

• Line 273 “ clustere”

Response: We are sorry, this is a spelling mistake. " clustere " has been changed to “clustered”.

• Line 276: “clade 3 o BAHDs”

Response: We are sorry, this is a spelling mistake. " clade 3 o BAHDs " has been changed to “clade 3 of BAHDs”.

• Repeated information in Line 231 and line 233: "study is the first to report that a member of the CYP714E subfamily functions as a C-24 hydroxylase of oleanane skeleton" and "There is no report on CYP714E19 funtioning as a oleanane C-24 hydroxylase until now". Please delete one of these.

Response: We thank the reviewer for carefully reading our manuscript and pointing out this error. We have deleted "*There is no report on CYP714E19 funtioning as a oleanane C-24 hydroxylase until now*".

Reviewer #4 (Remarks to the Author):

I co-reviewed this manuscript with one of the reviewers who provided the listed reports. This is part of the Nature Communications initiative to facilitate training in peer review and to provide appropriate recognition for Early Career Researchers who co-review manuscripts.
